# Chemical optimization of the exercise mimetic SLU-PP-332 enables insight into estrogen-related receptor signaling

**DOI:** 10.1016/j.ijbiomac.2026.151450

**Published:** 2026-03-16

**Authors:** Hazem Essam Okda, Puhan Zhao, Matt Hayes, Colten Duvall, Elsa Quillin, Hong Fang, Bassem M. Mohammed, Lamees Hegazy, Thomas P. Burris, Bahaa Elgendy

**Affiliations:** a Center for Clinical Pharmacology, Department of Anaesthesiology, Washington University in St. Louis, St. Louis, MO, 63110, United States; b Department of Cellular and Systems Pharmacology, University of Florida College of Pharmacy, Gainesville, FL, 32610, United States; c University of Florida Genetics Institute, University of Florida School of Medicine, Gainesville, FL, 32610, United States; d Department of Pharmaceutical and Administrative Sciences, University of Health Sciences and Pharmacy, St. Louis, MO, 63110, United States; e Edward A. Doisy Department of Biochemistry and Molecular Biology, Saint Louis University School of Medicine, St. Louis, MO, 63104, United States

**Keywords:** Estrogen-related receptors (ERRs), ERR agonists, SLU-PP-332, Exercise mimetics, Structure–activity relationship (SAR), Transcriptional regulation, Small-molecule modulators

## Abstract

Estrogen-related receptors (ERRs) are master regulators of mitochondrial metabolism and exercise-responsive transcription, yet only a limited number of synthetic agonists with suitable potency and drug-like properties have been reported. **SLU-PP-332** is a well-established exercise mimetic and widely used chemical probe for ERR activation; however, the structural features governing its potency, efficacy, selectivity, and drug-like properties have not been systematically elucidated. Here, we report the first comprehensive structure–activity relationship (SAR) analysis of the **SLU-PP-332** scaffold, integrating chemical synthesis, cell-based functional assays, downstream gene-expression profiling, and computational modeling. Through iterative modification of core pharmacophoric elements, we identify key structural determinants that control ERRα and ERRγ agonism, transcriptional efficacy, ligand efficiency, and physicochemical properties. While **SLU-PP-332** remains a strong benchmark for ERR activation, several analogues achieve comparable or context-dependent transcriptional responses while exhibiting improved ligand efficiency, solubility, or metabolic stability. Computational docking and molecular dynamics simulations reveal how subtle structural modifications influence ERR engagement and signaling outcomes. Together, this work defines design principles for tuning ERR agonism and provides a foundational SAR roadmap for the rational development of next-generation ERR agonists and exercise-mimetic therapeutics.

## 1. Introduction

Estrogen-related orphan receptors (ERRs) with their three subtypes, ERRα, ERRβ, and ERRγ, were identified as the first orphan nuclear receptors [1,2]. No endogenous ligands have been identified for the three isoforms till now. ERRα and ERRβ were identified in 1988 by Giguère and his co-workers [3], while ERRγ was identified in 1999 by Chen et al. [4,5]. The three ERR isoforms possess significant sequence homology with the estrogen receptors (ERs).

ERRs are prominently expressed in highly metabolically active tissues such as skeletal muscle, liver, brown adipose tissue, heart, and brain, and they are known as transcriptional regulators of energy metabolism [6,7]. Several studies have demonstrated that ERRα plays a vital role in regulating metabolism and energy homeostasis by interacting with multiple transcriptional cofactors including the peroxisome proliferator-activated receptor γ (PPARγ) coactivator 1 protein (i.e., PGC-1α and PGC-1β) [8,9].

**Fig. 1.**
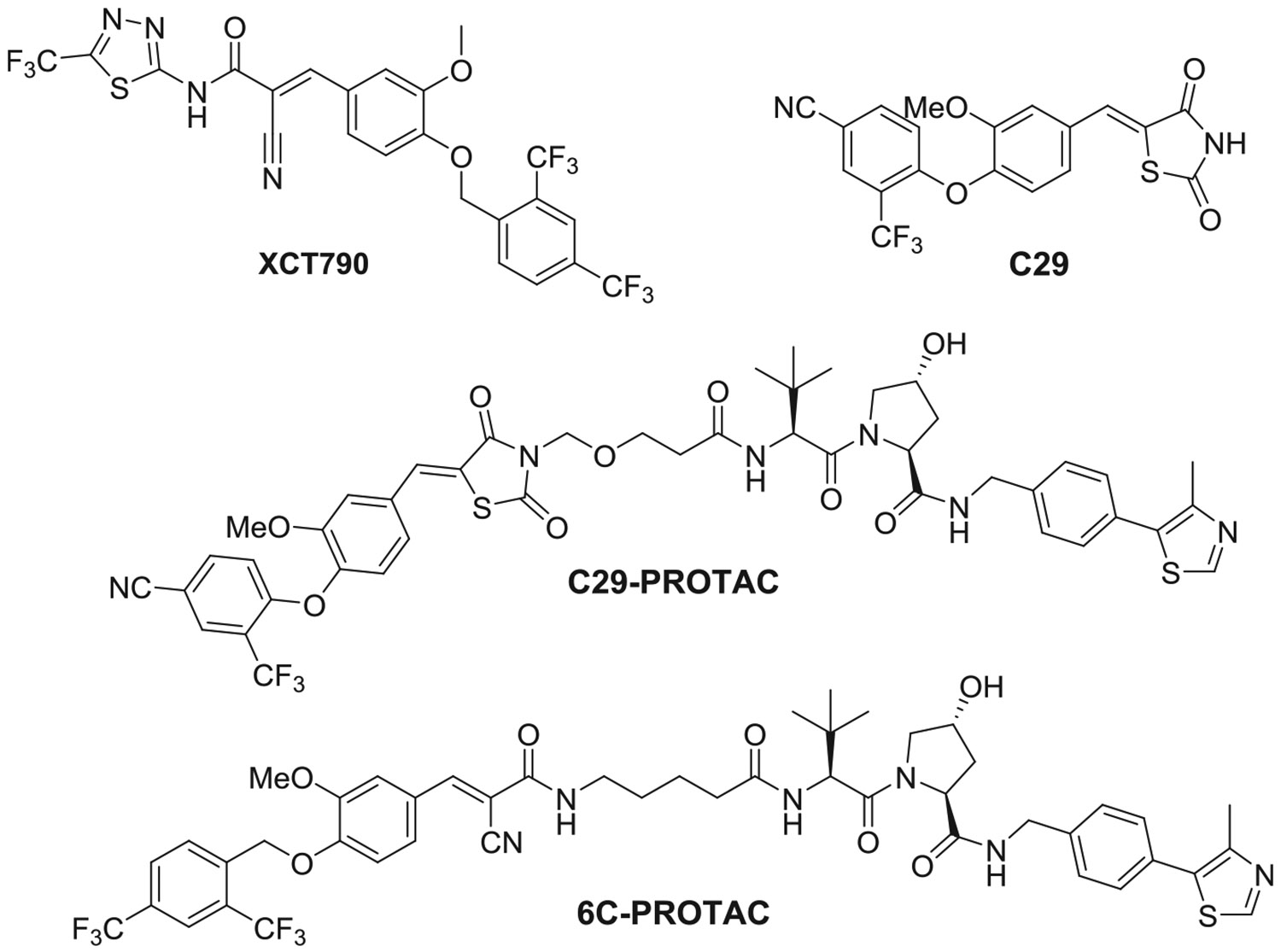
Chemical structures of ERRα inverse agonists and PROTACs.

Pharmacological inhibition of ERRα was shown to be a promising therapeutic strategy for treating breast cancer both in vitro and in vivo. In the last two decades, various novel classes of ERRα inverse agonists that could suppress the ERRα’s transcriptional functions have been identified (Fig. 1) [10–12]. Some synthetic ERRα inverse agonists inhibited tumor expansion by disrupting ERRs’ interaction with coactivators. Compound **XCT790** was the first reported and widely investigated ERRα inverse agonist with an IC_50_ = 0.37 μM in a GAL4-ERRα cell-based transfection assay [13]. Recently, Crews et al. reported the development of a PROTAC (proteolysis-targeting chimera) derived from compound **C29**, which was designed to induce the degradation of ERRα (Fig. 1) [14]. Building on this work, Ding and colleagues subsequently identified a novel ERRα inverse agonist, **6C**, also based on **C29**. This compound demonstrated potent and selective ERRα degradation, achieving >80% reduction of the protein at a relatively low concentration of 30 nM [15].

**Fig. 2.**
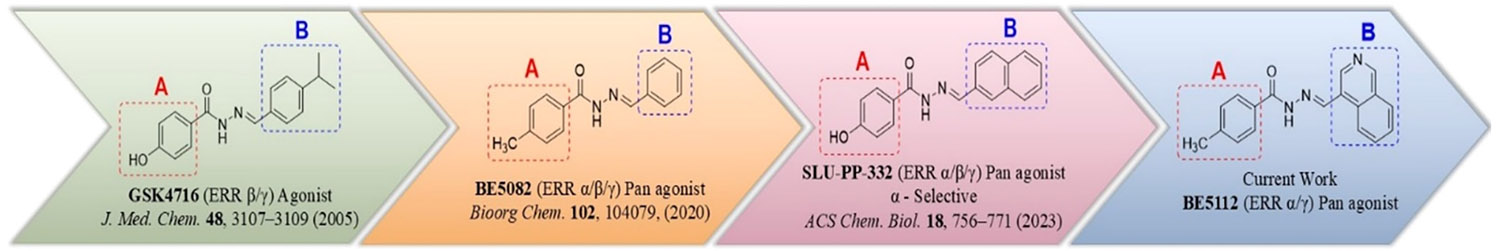
Development of *N*-acylhydrazones as ERR pan agonists.

Despite the successful development of ERRα inverse agonists, the development of novel ERRα agonists through high-throughput screening and structure-guided design has been unsuccessful for a long time. The primary obstacle in designing agonists was the size of the ERRα binding pocket, which is the smallest among all nuclear receptors [16]. In 2005, **GSK4716** (Fig. 2) was identified as the first agonist of ERRβ and ERRγ with no activity toward ERRα and showed remarkable selectivity over the classical estrogen receptors [17].

Recently, our lab has developed a new series of ERRα/β/γ panagonists through structural modification of the **GSK4716** scaffold (Fig. 2) [18]. These pan agonists were able to induce new conformational changes in the cavity of ERRα that led to the observed activity toward this subtype. We identified the structural requirements for ERRα activity, and molecular modeling showed that the new agonists have favorable binding modes in the ligand binding pocket (LBP) of ERRα. Interestingly, Phe328, which partially occupies the binding pocket of ERRα, is slightly displaced to open a space for the ligand to bind and form π-π interactions with the phenyl ring on ring B of **BE5082**. The identified ligands enhanced the expression of ERR target genes, PGC-1α, PGC-1β, CPT1α, and PDK4 in mouse myoblast cell line C2C12 [18]. Compounds that demonstrated the most promising activity toward ERRα did not exhibit any toxicity when tested at concentrations of 1 and 10 μM using the FITC Annexin V/Dead Cell Apoptosis Kit with FITC annexin V and propidium iodide -based assay [18].

Recently, we have identified another synthetic agonist, **SLU-PP-332**, with 4–5-fold selectivity for ERRα over ERRγ (Fig. 2) [19]. **SLU-PP-332** was designed based on the **GSK4716** and possesses naphthalene-2-yl moiety in lieu of the isopropyl moiety in **GSK4716**. The naphthyl moiety is more hydrophobic and planar and could bind and make π-π stack interactions with the Phe328 in the ligand binding domain (LBD) of ERRα.

**SLU-PP-332** was found to activate an acute aerobic exercise program and improve muscle and metabolic functions in vivo [19,20]. Specifically, **SLU-PP-332** upregulates the DDIT4 gene and enhances the mitochondrial function and cellular respiration. Moreover, **SLU-PP-332** has been identified as an exercise mimetic, which can play a crucial role in modulating age-related mitochondrial dysfunction and inflammation. These findings have identified new pathways that can be explored as potential drug targets to prevent the progression of age-related kidney diseases using ERR pan agonists [21].

Several nuclear receptors have been studied as potential targets for developing compounds to treat metabolic diseases. Among them, LXR, FXR, PPARα, PPARδ, PPARγ, and REV-ERB have been evaluated. However, among the nuclear receptors only the activation of REV-ERB [22] and PPARδ [23] through pharmacological means has been shown to have exercise-mimetic activity.

Notably, **SLU-PP-332** exhibits superior efficacy compared to known PPARδ and REV-ERB ligands in inducing acute aerobic exercise–related responses, a property that appears unique to ERRα activation. In mice, **SLU-PP-332** increased type IIa oxidative muscle fibers, enhanced exercise endurance, and selectively activated an ERRα-dependent aerobic exercise gene program. These findings highlight ERRα as a promising therapeutic target for developing exercise mimetics to treat metabolic disorders and improve muscle function in aging populations.

Building on the original discovery of **SLU-PP-332** as a potent exercise mimetic and chemical probe for ERR activation, the present study focuses on its chemical optimization to define structure–activity relationships that govern ERR signaling, ligand efficiency, and drug-like properties.

## 2. Results and discussion

### 2.1. Chemistry

**Scheme 1.**
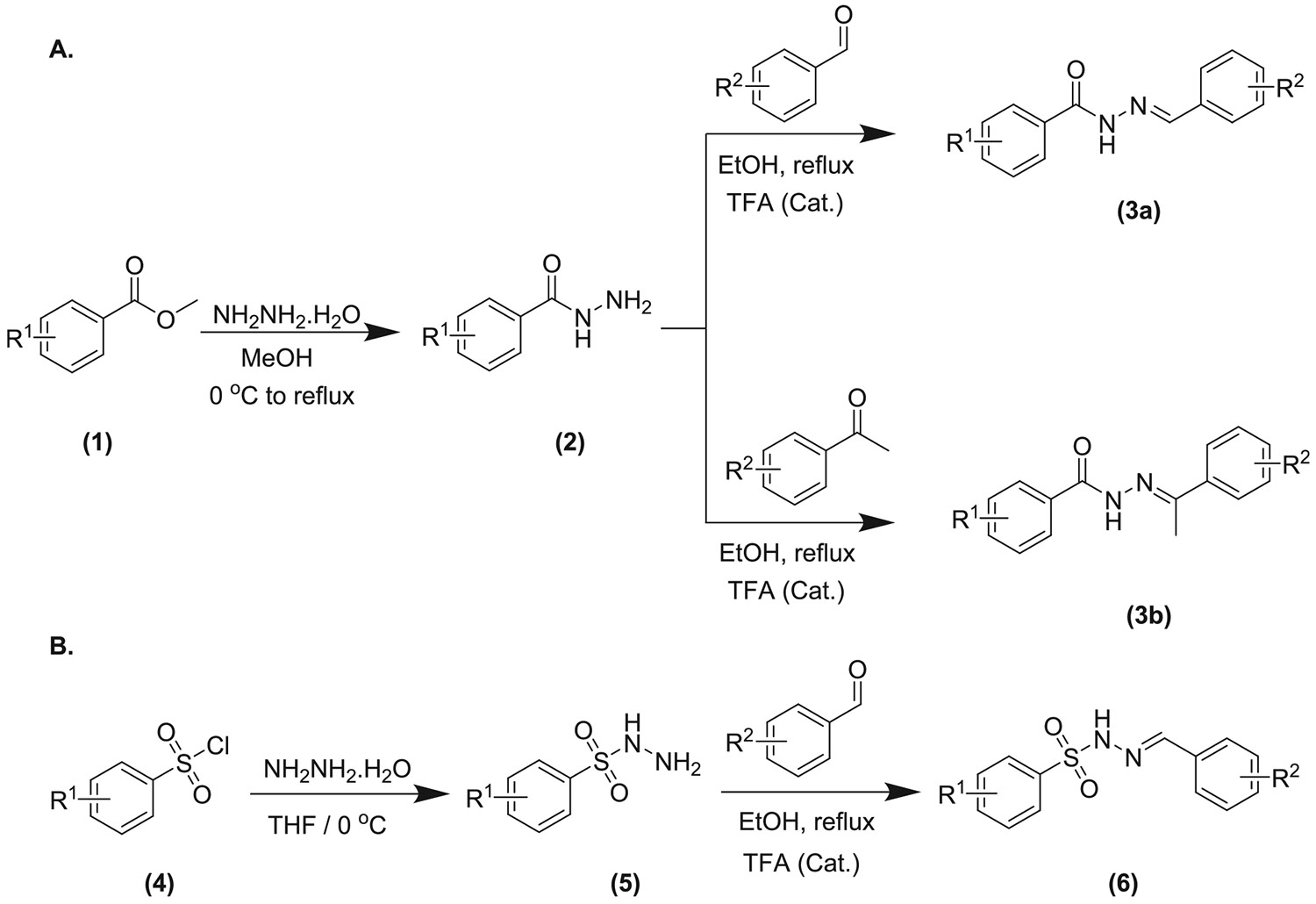
Synthesis of *N*-acyl hydrazones (A) and *N*-sulfonyl hydrazones (B).

The synthesis of all compounds was conducted according to the procedure outlined in Scheme 1. The preparation of acyl hydrazides **(2)** was achieved by reacting the corresponding methyl esters **(1)** with hydrazine in methanol under reflux conditions. The acyl hydrazides were obtained in quantitative yields, and their identities were confirmed by comparing their melting points and mass spectra with literature values. Subsequent condensation of the acyl hydrazides with aldehydes or ketones in ethanol, using a catalytic amount of trifluoroacetic acid, afforded the corresponding *N*-acyl hydrazones (Scheme 1A).

Similarly, the sulfonyl hydrazide intermediates **(5)** were generated by treating arylsulfonyl chlorides **(4)** with hydrazine monohydrate in THF at 0 °C. These intermediates were then condensed with the corresponding aldehydes under refluxing ethanol in the presence of catalytic trifluoroacetic acid to furnish the sulfonyl hydrazone derivatives **(6)** (Scheme 1B).

All synthesized compounds were thoroughly characterized using ^1^H, ^13^C NMR spectroscopy, and mass spectrometry.

### 2.2. Structure–activity relationship (SAR) study of hydrazone-based derivatives

The newly synthesized ligands were screened in a co-transfection cell-based assay using an ERRE reporter construct and expression plasmids pcDNA3.1-ERRα or pcDNA3.1-ERRγ. Luciferase activity was measured using One-Glo luciferase reporter assay system (Promega). The values represent the means with 95% confidence intervals from transfected wells (96-well plate). The EC_50_ values for most compounds described were determined using a seven-point dose-response titration. For compounds exhibiting high potency, where a sigmoidal dose-response curve could not be accurately generated with seven points, an 11-point dose-response titration was employed to obtain precise EC_50_ values.

**Fig. 3.**
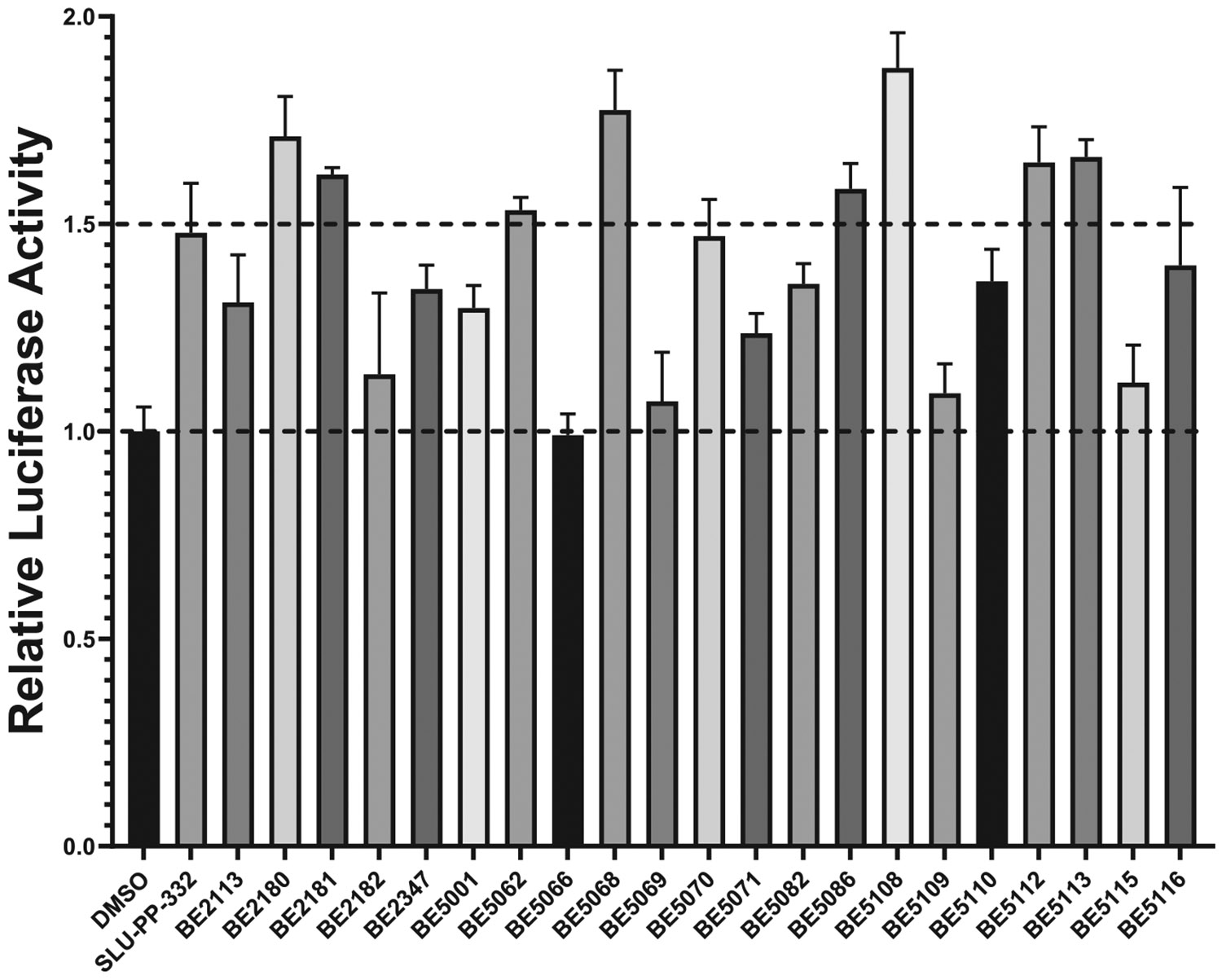
Comparison of the efficacy of putative ERR agonists in an ERR-luciferase co-transfection reporter assay. In vitro evaluation of synthesized analogs of **SLU-PP-332** and analogs were tested at 3.0 μM relative to vehicle (dimethyl sulfoxide, DMSO) treatment and normalized to cell number. The y-axis is relative luciferase activity which has been normalized to DMSO control (no drug). Standard error (SE) is represented using error bars.

The synthesized ligands were initially tested in a cell-based assay against ERRα at a concentration of 3.0 μM. To determine their activity, the fold activation of each compound was compared to DMSO, and their relative activation was compared to **SLU-PP-332**. The experimental results shown in Fig. 3 indicate that several synthetic ligands exhibited significantly higher activity than DMSO and comparable or superior activity to **SLU-PP-332**. For example, at 3.0 μM, compounds **BE2180** and **BE5112** demonstrated greater activity than **SLU-PP-332**.

Molecular modeling has become an increasingly important tool for designing new ligands for nuclear receptors (NRs). Molecular docking, which requires an accurate receptor structure, can provide valuable insights into how ligands bind to receptors. To better understand how the protein interacts with active ligands, we used the X-ray co-crystal structure of the ERRα LBD complexed with **DS45500853** and a PGC-1α coactivator peptide (PDB ID: 7E2E) [24] as the receptor conformation for docking.

**Fig. 4.**
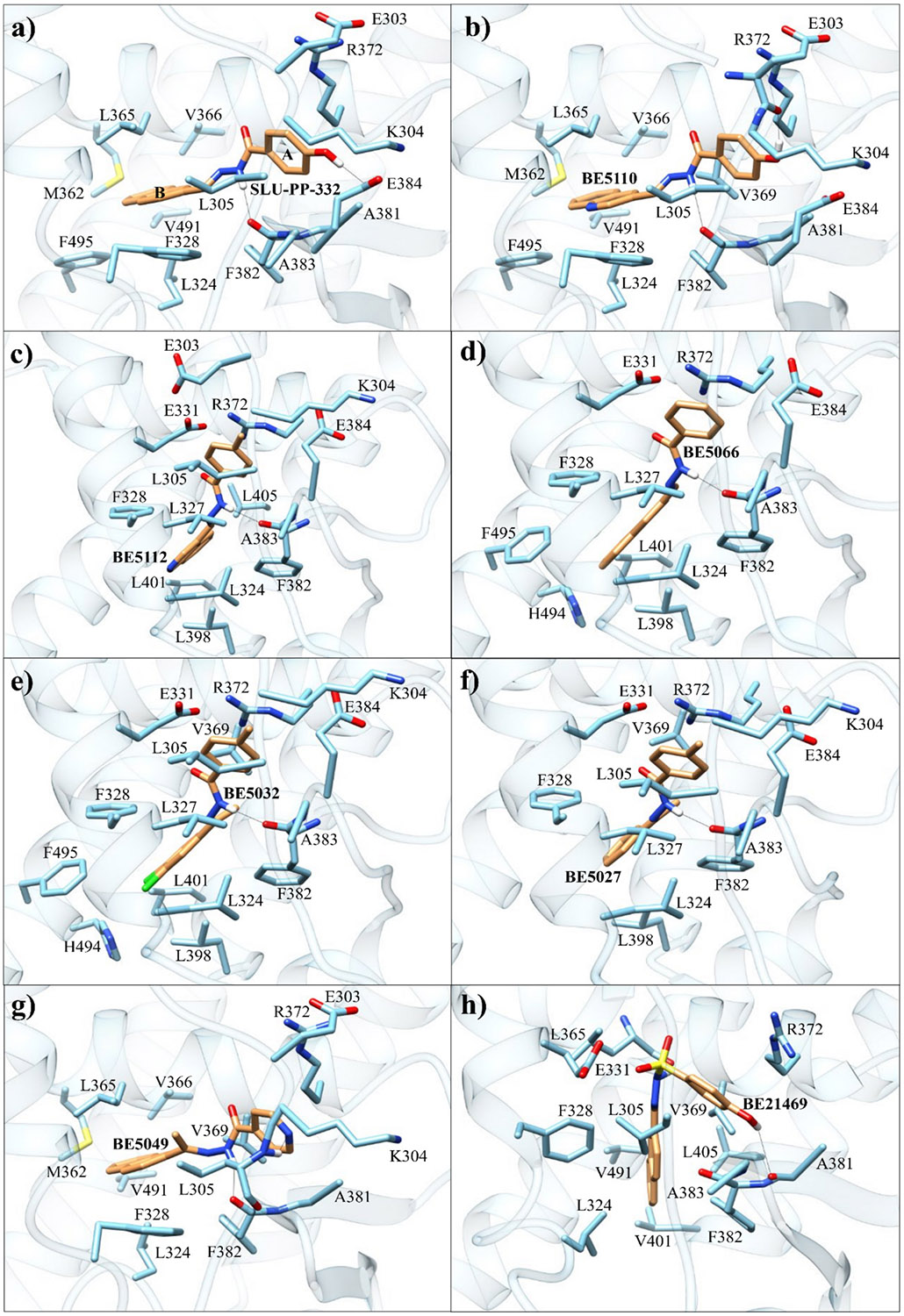
Docking poses of ligands bound to ERRα: a) **SLU-PP-332**, b) **BE5110**, c) **BE5112**, d) **BE5066**, e) **BE5032**, f) **BE5027**, g) **BE5049**, and h) **BE21469**.

Docking analysis of **SLU-PP-332** in the LBD of ERRα suggests that the hydroxyl group of ring A can form a hydrogen bond with Glu384 and the NH can form another hydrogen bond with Phe382 (Fig. 4a). Additionally, there was a π-π stacking interaction between the naphthalene moiety (ring B) and Phe328. The presence of the naphthalene group likely enhances the ligand’s affinity toward ERRα by interacting with Phe328. A previous study has demonstrated that the naphthalene group can dynamically interact with both Phe328 and Phe382 [25], which likely enhances the binding affinity of **SLU-PP-332** toward ERRα. Such interactions are absent in ERRβ and ERRγ, where Phe328 is replaced by alanine. Moreover, residues in the allosteric site, Phe382 and Gly402, in ERRα, are replaced by the residues of Tyr301/Tyr321 in ERRβ and Tyr326/Asn346 in ERRγ, which sterically hinder the accommodation of the naphthalene group. Consistent with these observations, **SLU-PP-332** had a better docking score than the native ligand **DS45500853** with ERRα (docking score = −11.706 for **SLU-PP-332** vs −8.233 kcal/mol for **DS45500853**).

We hypothesized that by modifying ring B, we could potentially cause a change in the way the ligand interacts with the LBD. This, in turn, could lead to an improvement in the potency and efficacy of the compound, as well as changes in selectivity toward ERRα. To test this hypothesis, we decided to replace naphthalene with bioisosteres (e.g., quinolines and isoquinolines) and positional isomers (e.g., 1-naphthyl group) to examine the effect of these modifications on molecular interactions and potency toward ERRα/γ.

We began exploring the SAR of ring B while keeping ring A fixed as a 4-hydroxy phenyl group (Table 1). Replacing the naphthalene moiety in **SLU-PP-332** with a quinoline-2-yl moiety yielded compound **BE2180**. **BE2180** functioned as a pan agonist and showed activity comparable to **SLU-PP-332** toward ERRα (EC_50_ = 0.31 μM (**BE2180**) vs 0.22 μM (**SLU-PP-332**)) while being approximately 2-fold more potent toward ERRγ (EC_50_ = 0.331 μM (**BE2180**) vs 0.59 μM (**SLU-PP-332**)). Although **BE2180** exhibited more than 2-fold decrease in efficacy at ERRα relative to **SLU-PP-332**, it maintained a clear potency advantage at ERRγ.

**Fig. 5.**
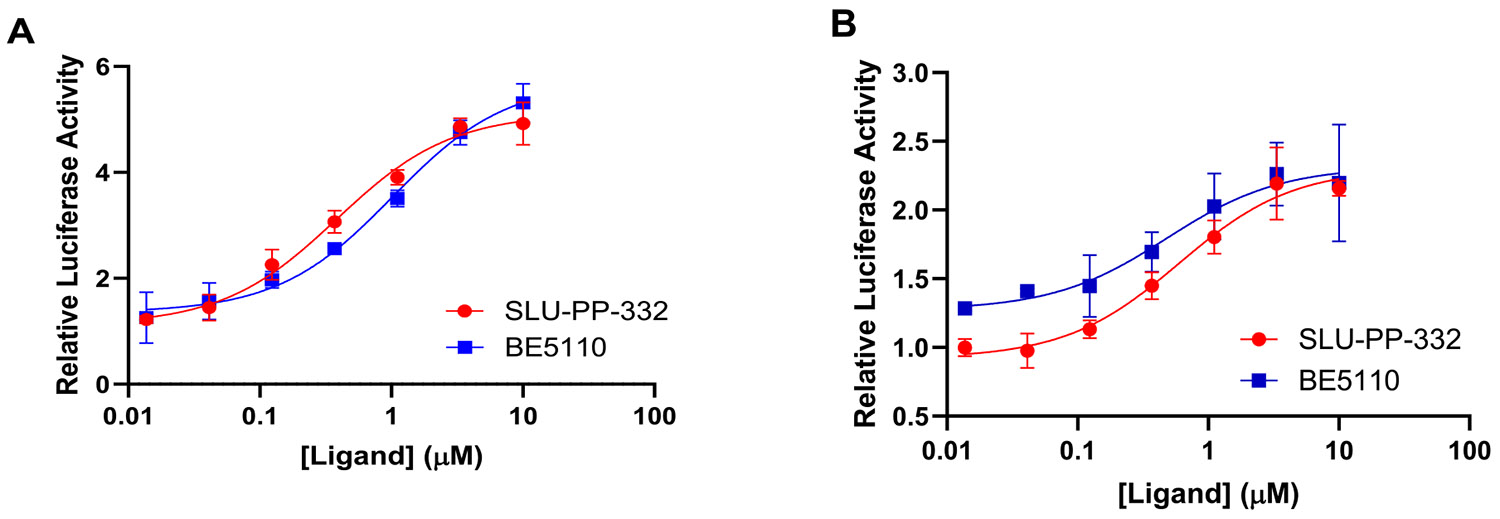
Full-length ERRα (A) and ERRγ (B) transfection assay results in HEK293 cells comparing the agonist activity of **BE5110** with **SLU-PP-332**.

Introduction of an isoquinolin-3-yl group on ring B yielded **BE21465**, which was completely inactive, whereas introduction of a quinoline-3-yl group on ring B yielded **BE5110**. Although **BE5110** was over four-fold less potent than **SLU-PP-332** at ERRα, it exhibited enhanced efficacy (Fig. 5). Notably, **BE5110** demonstrated improved activity toward ERRγ (EC_50_ = 0.48 μM) but with reduced efficacy relative to **SLU-PP-332** (Table 1). Molecular docking of **BE5110** in ERRα (Fig. 4b) revealed that the compound formed two hydrogen bonds: one retained with the backbone of Phe382, as seen with **SLU-PP-332**, and a second with the backbone of Glu303, replacing the interaction with the carboxylate of Glu384. However, loss of π-π stacking interactions by the quinoline ring corresponded with a lower docking score (DS = −9.333 kcal/mol) compared to **SLU-PP-332**.

Repositioning of the nitrogen atom from the quinoline-3-yl in **BE5110** to quinoline-4-yl position produced **BE5116**, a modification that proved detrimental, as the compound was inactive against both ERRα and ERRγ.

Replacing the naphthyl group of **SLU-PP-332** with a quinoxaline-2-yl moiety resulted in **BE5070**, which showed promising selectivity toward ERRα (EC_50_ α/γ = 0.7). This substitution significantly enhanced potency toward both ERRα (EC_50_ = 0.187 μM) and ERRγ (EC_50_ = 0.269 μM). Although **BE5070** displayed approximately half the efficacy of **SLU-PP-332**, it retained the same Ligand Efficiency (LE = 0.42).

Unexpectedly, replacement of ring B in **SLU-PP-332** with the positional isomer naphth-1-yl moiety produced **BE5068**, which was equipotent toward both ERR isoforms (EC_50_ = 0.36 μM). Introducing the naphth-1-yl bioisostere quinoline-4-yl yielded **BE5069**, which showed comparable ERRα activity (EC_50_ = 0.308 μM) but slightly reduced ERRγ potency (EC_50_ = 0.532 μM). Despite its favorable ERRα potency, **BE5069** exhibited more than 2-fold decrease in efficacy relative to **SLU-PP-332** (Table 1).

Finally, replacing the naphth-2-yl group in **SLU-PP-332** with other naphth-1-yl bioisosteres such as isoquinoline-4-yl (**BE5113**), quinoline-5-yl (**BE5071**), and isoquinoline-5-yl (**BE5117**), resulted in a complete loss of activity against both ERR isoforms (Table 1).

**Fig. 6.**
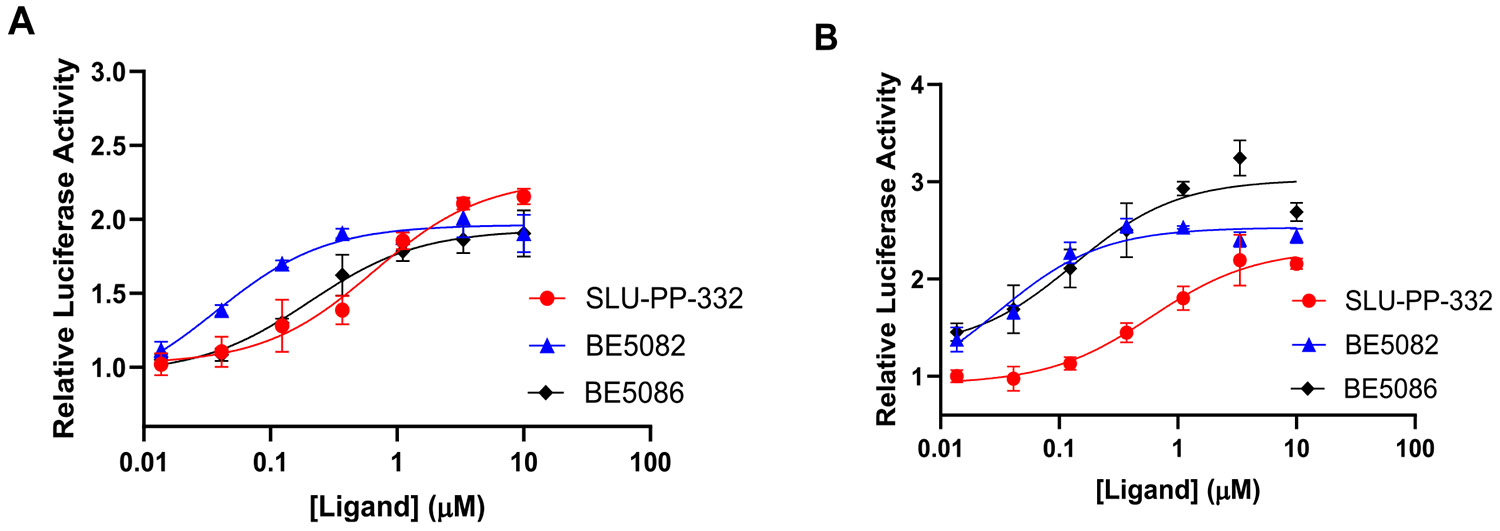
Full-length ERRα (A) and ERRγ (B) transfection assay results in HEK293 cells comparing the agonist activity of **BE5082**, **BE5086**, and **BE5112** with **SLU-PP-332**.

**BE5082** was previously reported to exhibit high potency toward both ERRα (EC_50_ = 0.209 μM) and ERRγ (EC_50_ = 0.194 μM) along with moderate selectivity for ERRα (EC_50_ α/γ = 1.07) [18]. When evaluated in the current full-length cell-based luciferase reporter assay, **BE5082** demonstrated significantly enhanced potency (ERRα: EC_50_ = 0.036 μM; ERRγ: EC_50_ = 0.031 μM) (Table 2, Fig. 6). The apparent increase in potency likely reflects differences in assay format and experimental conditions. Given this improved profile, we next explored the impact of introducing a 4-methyl substituent on ring A and systematically probed the influence of naphth-2-yl, its bioisosteres, and positional isomers on ring B.

To initiate the SAR exploration, the phenyl substituent on ring B of **BE5082** was replaced with a naphth-2-yl moiety, a defining structural feature of **SLU-PP-332**, yielding **BE2181** (Table 2). **BE2181** displayed reduced potency and efficacy compared to **BE5082**, while maintaining comparable potency to **SLU-PP-332** at ERRα and lower potency at ERRγ. Efficacy was reduced across both isoforms.

Replacing the phenyl group (ring B) of **BE5082** with a quinoline-2-yl moiety yielded **BE2182**. This modification resulted in an approximately 10-fold decrease in ERRα potency (EC**50 =** 0.324 μM) relative to **BE5082**, although activity remained comparable to **SLU-PP-332**. In addition, **BE2182** showed more than 25-fold reduction in potency toward ERRγ (EC_50_ = 0.78 μM) compared to **BE5082** (Table 2). Shifting the nitrogen from the quinoline-2-yl to the quinoline-3-yl position yielded **BE5109**, which was inactive at both ERR isoforms. Introducing an isoquinolin-3-yl group yielded **BE21466**, which was likewise completely inactive. Further repositioning of the nitrogen to quinoline-6-yl afforded **BE5115**, which exhibited modest ERRα activity (EC_50_ = 0.531 μM) but substantially reduced ERRγ activity, resulting in high selectivity for ERRα (SI = 0.3).

Substitution of the phenyl ring in **BE5082** with a quinoxaline-2-yl group produced **BE5001**, which showed more than 12-fold decrease in ERRα potency (EC_50_ = 0.45 μM) relative to **BE5082** and a 1.5-fold reduction in efficacy compared to **SLU-PP-332**. **BE5001** retained moderate ERRγ activity (EC_50_ = 0.76 μM) and showed improved selectivity for ERRα (SI = 0.6).

Introducing a naphth-1-yl group on ring B produced **BE5086**, a compound markedly more potent at both ERR isoforms. **BE5086** was equipotent with **SLU-PP-332** at ERRα (EC_50_ = 0.2 μM; E_max_ = 75.6%) and displayed robust ERRγ activity (EC_50_ = 0.13 μM), with good selectivity favoring ERRγ (SI = 1.5) (Table 2, Fig. 6). To our delight, introducing an isoquinoline-4-yl moiety as ring B led to the discovery of **BE5112**, which exhibited remarkable potency (EC_50_ = 0.098 μM) and superior efficacy toward ERRα compared to **SLU-PP-332** (Table 5, Fig. 8). **BE5112** also demonstrated greater potency and efficacy toward ERRγ (EC_50_ = 0.110 μM) than **GSK4716**. Consistent with these findings, **BE5086** and **BE5112** were the most potent agonists to both ERR isoforms in the series and displayed the highest LE values, second only to **BE5082**.

Molecular docking of **BE5112** (Fig. 4c) indicated that replacing the ring A hydroxyl group of **SLU-PP-332** with a methyl group abolished the hydrogen bond with Glu384. However, this modification repositioned ring A to form an anion-π interaction with Arg372 while maintaining the hydrogen bond with the backbone of Phe382. As a result, **BE5112** exhibited a docking score of −10.549 kcal/mol, which was less favorable than **SLU-PP-332** but better than **BE5110**.

Further modifications at ring B were less productive. Incorporating a quinoline-4-yl substituent generated **BE2113**, which showed reduced potency toward both ERRα (EC_50_ = 1.64 μM) and ERRγ (EC_50_ = 1.23 μM) relative to **BE5112**. Introduction of a quinoline-5-yl moiety provided **BE2347**, which exhibited weaker activity toward both isoforms (ERRα EC_50_ = 0.604 μM; ERRγ EC_50_ = 0.693 μM) compared to **BE5112** and **BE5082** (Table 2). Finally, replacing the isoquinoline-4-yl group in **BE5112** with an isoquinoline-5-yl moiety to yield **BE5118** resulted in a complete loss of activity at both receptors, despite the structural simplicity of this modification.

**Fig. 7.**
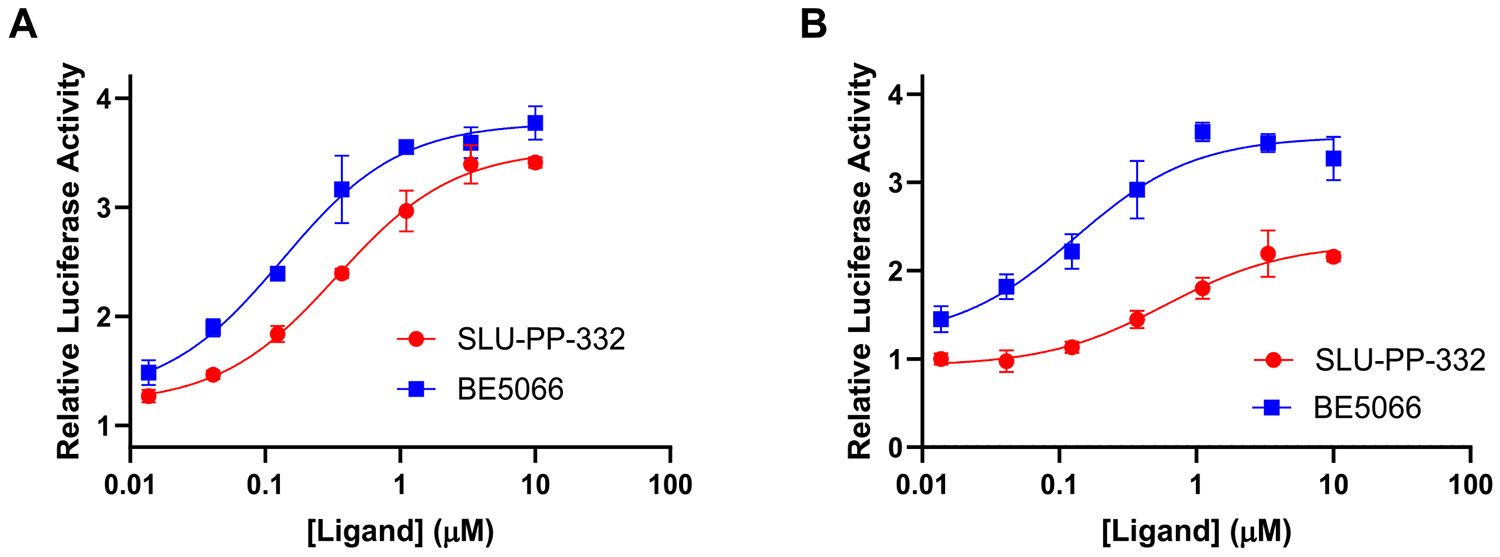
Full-length ERRα (A) and ERRγ (B) transfection assay results in HEK293 cells comparing the agonist activity of **BE5066** with **SLU-PP-332**.

**Fig. 8.**
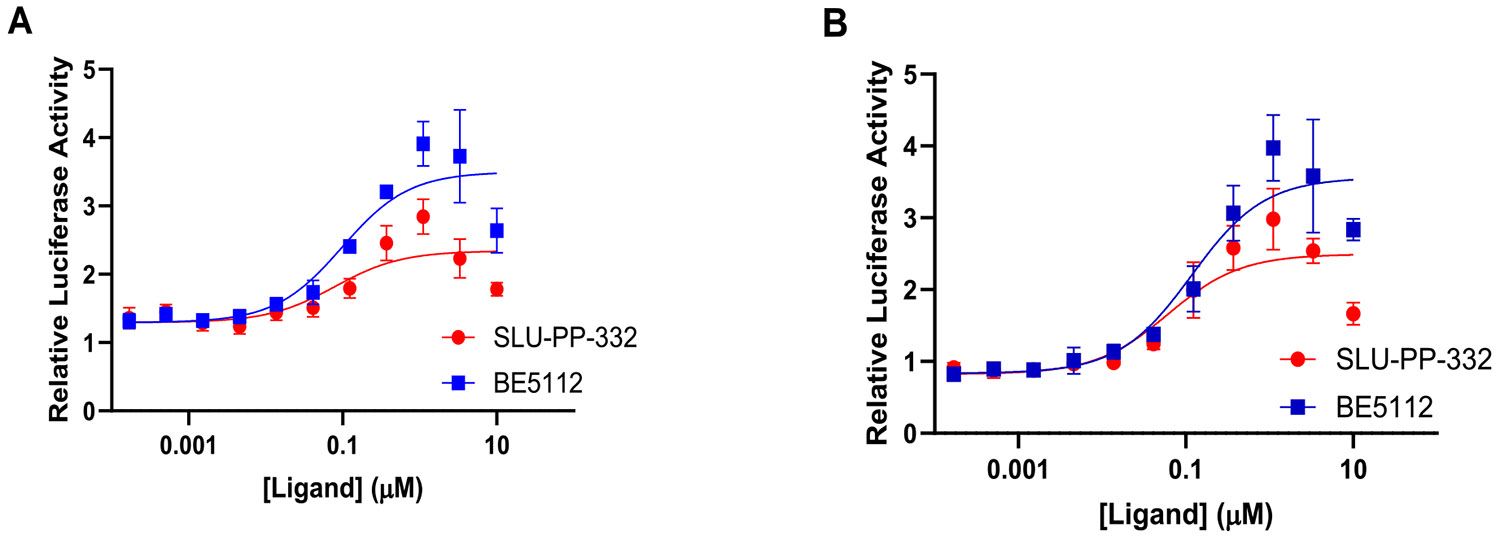
Full-length ERRα (A) and ERRγ (B) transfection assay results in HEK293 cells comparing the agonist activity of **BE5112** with **SLU-PP-332**.

Finally, we investigated the SAR of ring B while maintaining an unsubstituted phenyl group on ring A (Table 3). Introduction of a naphth-2-yl moiety produced **BE5066**, which was more potent than **SLU-PP-332** against both ERRα (EC_50_ = 0.133 μM) and ERRγ (EC_50_ = 0.129 μM) and exhibited significantly higher efficacy toward ERRγ (Table 3, Fig. 7). Similar to **SLU-PP-332**, docking analysis (Fig. 4d) revealed the ─NH group of **BE5066** formed a hydrogen bonding interaction with the backbone of Phe382. Although dynamic binding behavior cannot be captured by docking, the naphthalene system is expected to adopt a similar binding mode to **SLU-PP-332**. Additionally, **BE5066** engages in an anion-π interaction with Arg372, resulting in a docking score (−11.138 kcal/mol) comparable to that of **SLU-PP-332**.

Replacing the naphth-2-yl group of **BE5066** with quinoline-2-yl, isoquinoline-3-yl, or quinoline-3-yl yielded **BE5062**, **BE21467** and **BE5108**, respectively (Table 3). Unfortunately, all three compounds were inactive in both ERR isoforms. However, repositioning the nitrogen atom from the quinoline-3-yl position of **BE5108** to the quinoline-6-yl position restored activity, giving **BE5114**, which showed strong potency toward ERRα (EC_50_ = 0.091 μM) and ERRγ (EC_50_ = 0.14 μM). **BE5114** also displayed improved selectivity for ERRα (SI = 0.7) (Table 3).

Incorporation of a quinoxaline-2-yl group on ring B afforded **BE5064**, which displayed high potency and moderate efficacy toward ERRα (EC_50_ = 0.128 μM, *E_max_* = 42%). **BE5064** also maintained good ERRγ potency (EC_50_ = 0.29 μM) but with very low efficacy (*E_max_* = 13.4%).

Replacing naphth-2-yl moiety of **BE5066** with a naphth-1-yl group yielded **BE5067**, which demonstrated strong potency toward both ERRα (EC_50_ = 0.227 μM) and ERRγ (EC_50_ = 0.262 μM). However, **BE5067** showed reduced efficacy at ERRα compared to **SLU-PP-332**, while maintaining high efficacy at ERRγ. Introduction of a quinoline-5-yl moiety at ring B generated **BE5065**, which exhibited moderate ERRα potency (EC_50_ = 0.732 μM) and approximately 1.25-fold higher efficacy than **SLU-PP-332**. **BE5065** showed weak ERRγ activity (EC_50_ = 1.72 μM), indicating potential for ERRα selectivity (SI = 0.4).

Finally, replacement of the naphth-1-yl group in **BE5067** with naphth-1-yl bioisosteres, isoquinoline-4-yl (**BE5111**), quinoline-4-yl (**BE5063**), and isoquinoline-5-yl (**BE5119**), was not tolerated, and all three compounds were inactive toward both ERR isoforms.

Inspired by the historical discovery of pronethalol from dichloroisoprenaline, we replaced the naphthalene ring system of **SLU-PP-332** with 3,4-dichlorophenyl group to generate **BE5136**, aiming to examine how this modification affects the pharmacology of **SLU-PP-332**. This substitution was intentionally designed to probe induced-fit interactions in the binding site without significantly altering physicochemical properties, as **BE5136** and **SLU-PP-332** possess nearly identical parameters (cLogP, tPSA, LogS, and pKa). Interestingly, **BE5136** lost preferential ERRα selectivity observed for **SLU-PP-332**, and instead exhibited slightly greater potency toward ERRγ than ERRα (Table 4). This shift on isoform preference highlights the essential contribution of the naphthalene ring system to maintaining ERRα selectivity, independent of overall physicochemical properties.

We next applied this substitution strategy to **BE2181** and **BE5066**, generating **BE5137** and **BE5138**, respectively (Table 4). **BE2181**, which contains a naphthyl ring B, displayed clear ERRα selectivity driven by reduced ERRγ potency (Table 2), whereas **BE5066** showed nearly equipotent activity at both isoforms (Table 3). Replacement of the naphthalene ring with a 3,4-dichlorophenyl group in **BE5137** resulted in diminished ERRα potency and a pronounced shift toward ERRγ selectivity (Table 4). In contrast, **BE5138** retained the near-equipotent profile of its parent compound, **BE5066**, indicating that this substitution did not confer isoform selectivity in this context. Overall, these results demonstrate that replacement of the naphthalene system with a 3,4-dichlorophenyl group disrupts ERRα-favored interactions and either attenuates or reverses ERRα selectivity, underscoring the importance of optimal naphthalene engagement within the binding pocket for ERRα-selective activation.

Interestingly, **BE5032**, which features two methyl substituents at R^1^ and R^2^ retained higher potency and efficacy toward ERRα (EC_50_ = 0.34 μM, *E*_max_ = 110%) compared to ERRγ (EC_50_ = 0.45 μM, *E*_max_ = 75%) (Table 4). **BE5032** exhibited a docking score of −11.281 kcal/mol, which was comparable to those of **BE5112** and **SLU-PP-332**, and adopted a similar binding pose. Specifically, it preserved the hydrogen bond between the hydrazone NH and the backbone of Phe382, as well as the anion–π interaction between ring A and Arg372 (Fig. 4e).

To further investigate the SAR at the hydrazone double bond, we introduced a methyl substituent in place of the hydrogen atom. This modification was applied to the most promising ligands from previous iterations to assess whether the binding pocket could accommodate greater steric bulk, enhance binding affinity, or if it inherently favors smaller substituents. We also sought to determine whether modulating sterics and electronics at this position could influence isoform selectivity between ERRα and ERRγ.

**Fig. 9.**
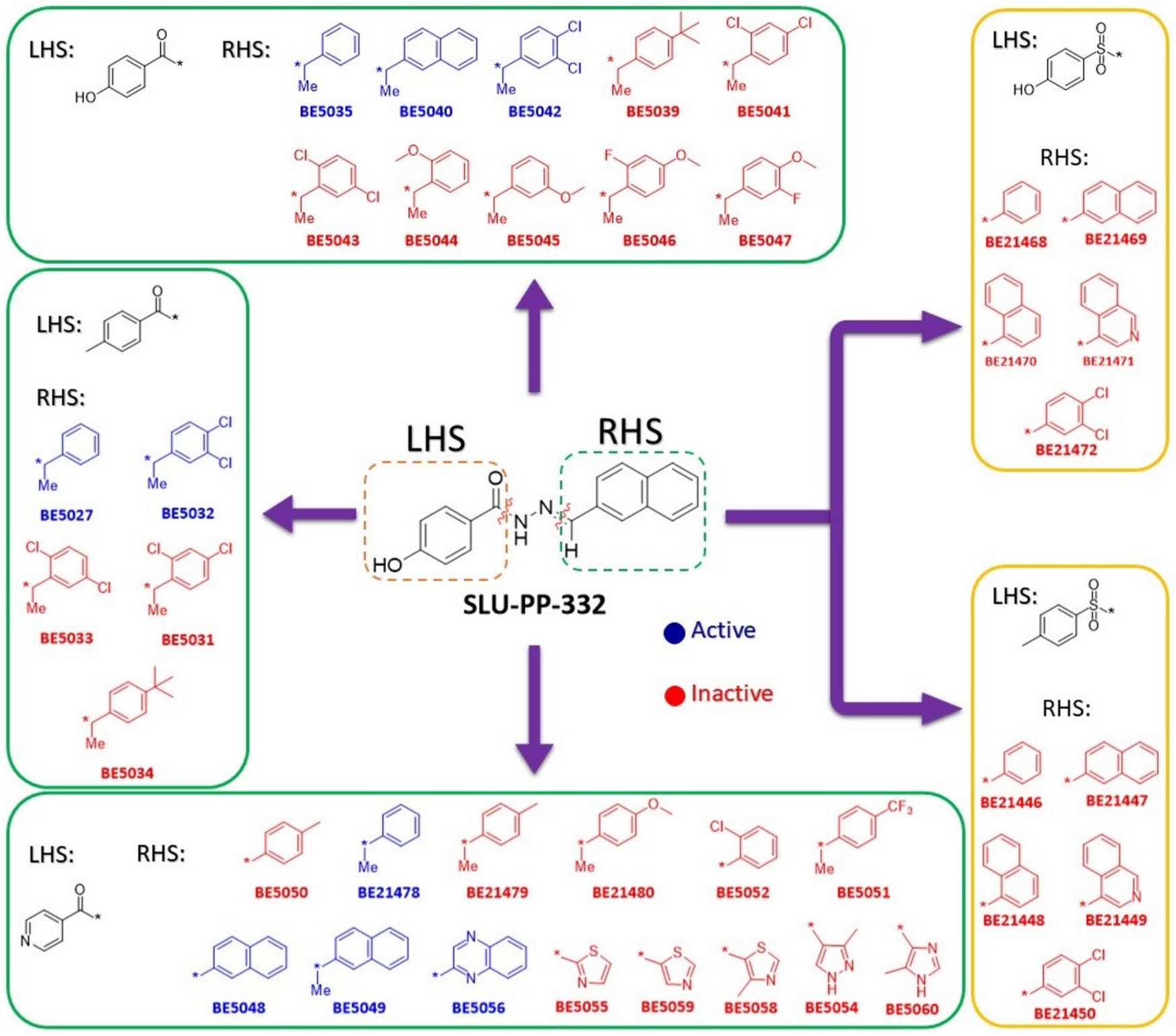
Summary of key structure–activity relationship (SAR) trends derived from Tables S1–S5, highlighting the influence of ring A, ring B, the hydrazone linker, and functional group modifications on ERRα and ERRγ activity (Blue modifications: Active; Red modifications: Inactive).

Overall, the methyl substitution was generally detrimental to activity at both ERR isoforms (Tables S1–S3, Fig. 9). However, in a subset of compounds, this change produced highly potent ligands. Notably, **BE5027**, **BE5035**, **BE5049**, and **BE21478** demonstrated strong potency toward both ERRα and ERRγ (Table 5). The methyl-substituted analogues exhibited higher cLogP values, indicating increased lipophilicity, which may enhance membrane permeability and contribute to improved activity. **BE5027** showed a lower docking score (DS = −9.853 kcal/mol) compared to **SLU-PP-332**. Although **BE5027** retained the anion-π interaction with Arg372 and the H-bond with Phe382 (Fig. 4f), replacing the naphthalene moiety with a phenyl ring likely weakened potential π-π interactions with Phe328 and Phe382.

Notably, introduction of a methyl substituent at the hydrazone position (**BE5040**) dramatically increased the potency toward both isoforms (EC_50_ (μM) = 0.002 (ERRα) and 0.004 (ERRγ)) relative to **SLU-PP-332** (Table 5). This observation highlights the sensitivity of the hydrazone moiety to subtle substituent changes (e.g., ─Me vs. ─H) and underscores the importance of fine-tuning this region to optimize potency, selectivity, and physicochemical properties.

To further probe the role of hydrogen bonding at ring A, the phenolic group of **SLU-PP-332** was replaced with a pyridine ring (Tables 5, S3, and Fig. 9). Substituting a hydroxyl group with a pyridine nitrogen can significantly alter ligand orientation within the binding site by changing hydrogen bonding capabilities and basicity. While the pyridine nitrogen may enable alternative interactions and potentially enhance selectivity, the loss of the phenolic hydrogen bond, often critical for high-affinity binding, can compromise potency.

Pyridine also reduces lipophilicity and alters metabolic properties, which may influence solubility, metabolic stability, and overall pharmacokinetic behavior. Exploration of SAR at ring B within this pyridine-containing scaffold revealed that most aromatic and heterocyclic substitutions led to diminished or lost activity at both ERR isoforms (Table S3, Fig. 9). This trend suggests that the phenol-to-pyridine substitution imposes stricter structural requirements for productive binding, consistent with the reduced tolerance for variation at ring B. This loss likely reflects the diminished hydrogen-bonding capacity following phenol-to-pyridine substitution, reinforcing the importance of these interactions for ERR binding.

Despite this overall trend, a limited subset of compounds (**BE5048**, **BE5049**, **BE5056**, and **BE21478**) retained high potency (Table 5). These results indicate that appropriate combinations of ring B substitution and hydrazone modification can partially compensate for the loss of the phenolic hydroxyl group, emphasizing the importance of precise substitution patterns for maintaining potency and selectivity in both ERRα and ERRγ agonists.

Docking studies of **BE5049** revealed a binding mechanism similar to that of **SLU-PP-332** (Fig. 4g). In addition to the dynamic binding behavior of the naphthalene moiety observed with **SLU-PP-332** and the conserved hydrogen bond with Phe382, **BE5049** formed an additional hydrogen bond with the backbone of Leu305. This interaction may contribute to its favorable docking score (−11.643 kcal/mol), which is comparable to that of **SLU-PP-332** and consistent with its retained in vitro activity.

To assess the importance of the carbonyl group within our pharmacophore, we synthesized benzenesulfonohydrazide derivatives (Scheme 1B) based on the most potent benzohydrazides identified in earlier iterations (Tables S4 and S5, Fig. 9). However, replacing the carbonyl group with an SO_2_ moiety proved detrimental, resulting in a complete loss of activity across all analogues. The carbonyl group is essential for maintaining the correct electronic environment, supporting strong hydrogen-bonding interactions, and preserving the proper molecular geometry required for optimal engagement of benzohydrazide derivatives with the receptor. In contrast, substitution with an SO_2_ group disrupts these critical interactions, leading to the observed loss of activity.

The SO_2_ group is significantly more electron-withdrawing than a carbonyl, significantly altering the electronic distribution of the molecule. This perturbation can destabilize the finely tuned electronic interactions necessary for high-affinity receptor binding. Although SO_2_ can participate in hydrogen bonding, the interactions it forms are generally weaker and less geometrically favorable than those formed by carbonyl groups, reducing compatibility with key binding-site residues. Additionally, the SO_2_ group introduces a bulkier, more tetrahedral geometry compared to the planar carbonyl, which can generate steric clashes or prevent proper positioning within the LBP, further impairing activity.

Molecular docking of **BE21469** (Fig. 4h) yielded a moderately favorable score of −10.856 kcal/mol, though less favorable than that of **SLU-PP-332**. **BE21469** adopted a binding pose distinct from other ligands in this series. The conserved hydrogen bond between the hydrazone NH and the backbone of Phe382, observed in active analogues, is replaced by an interaction with the backbone of Leu365. Similarly, the hydroxyl-Glu382 hydrogen bond present in **SLU-PP-332** is replaced by an interaction between the hydroxyl group and the backbone of Ala381. These altered interactions, combined with the tetrahedral geometry and reduced flexibility of the ─SO_2_ moiety, as well as the steric constraints imposed by the naphthalene ring, may limit conformational adaptability and hinder the dynamic binding behavior characteristic of **SLU-PP-332**.

It is also important to emphasize that docking may reflects a static binding model. Although **BE21469** can be accommodated within the LBP and is predicted to engage key residues, the electron-withdrawing nature and polarity of the ─SO_2_ group could perturb local charge distribution and weaken intermolecular interactions. As a result, **BE21469** may exhibit reduced binding stability under experimental or dynamic simulation conditions, consistent with its observed lack of activity.

In summary, across the benzohydrazide chemotype, ring B primarily governs potency and efficacy, whereas ring A and the hydrazone linkage modulate selectivity and drug-like properties. Swapping **SLU-PP-332**’s naphth-2-yl for closely related fused heteroarenes shows a sharp positional dependence: isoquinoline-4-yl is strongly favored (**BE5112**; high potency/efficacy at both ERRα/γ), whereas isoquinoline-5-yl and quinoline-5-yl analogues generally reduce or abolish activity. Quinoline-2-yl (**BE2180**) largely preserves ERRα potency but reduces efficacy and shifts toward ERRγ potency, while quinoline-3-yl and quinoline-4-yl substitutions tend to be less potent or inactive unless accompanied by complementary structural features.

Within the 4-methyl ring A series (**BE5082** lineage), reintroducing extended aromaticity on ring B (e.g., naphth-1-yl in **BE5086**) restores potency and may bias activity toward ERRγ. The isoquinoline-4-yl analogue (**BE5112**) again emerges as the optimal balance of ERRα/γ agonism. Replacement of the carbonyl group with a sulfonyl (–SO_2_–) moiety eliminates activity across the series, reinforcing the essential role of the carbonyl in maintaining proper geometry and hydrogen-bonding interactions. Introducing a 3,4-dichlorophenyl in place of naphthalene reverses selectivity toward ERRγ (e.g., **BE5137**), underscoring the role of the extended π-system in ERRα-biased activation. Finally, swapping phenol with pyridine on ring A is largely deleterious (loss of key H-bonding), with a few high-potency exceptions that succeed only when ring B and the hydrazone environment are co-optimized (e.g., **BE5049**).

Adding a methyl substituent at the hydrazone double bond (–CH=N–) is generally unfavorable and often reduces activity; however, in specific molecular contexts, it enables exceptional potency. In those contexts (e.g., **BE5027**, **BE5049**, **BE21478**), the methyl likely improves fit and hydrophobic packing (and sometimes permeability), yielding low-nanomolar EC_50_ values. However, this same modification can reverse selectivity or reduce efficacy if ring B’s π-surface or ring A electronics do not sufficiently support the productive “sandwich-like” pose necessary for coactivator-competent conformations. Thus, the methyl substituent should be viewed as a context-dependent optimization element, reserved for ring B frameworks with extended or fused aromatics that sustain π-stacking with Phe328/Phe382 and paired with ring A substitutions capable of retaining at least one strong backbone or polar contact.

### 2.3. In vitro ADME and ligand efficiency

The in vitro ADME profiles of selected ERR modulators were evaluated to assess their developability and to identify structure–property relationships (Table 6). Overall, the series displayed a range of lipophilicities (cLogP 3.2–4.7), solubilities (0.2–66 μM), and metabolic half-lives (t½ 6.6–47.7 min), illustrating clear relationships between structural modifications and pharmacokinetic behavior.

The reference agonist **SLU-PP-332** exhibited extremely low kinetic solubility (0.2 μM) consistent with its higher cLogP (4.05). Its moderate microsomal stability (t_1/2_ = 31.3 min; Cl_int_ = 22 μL/min/mg) and low LE values (0.42 and 0.31 for ERRα and ERRγ) highlight the need for structural optimization to improve physicochemical balance and metabolic resilience. Substitution with a quinoline-4-yl group, as in **BE2180**, reduced lipophilicity (cLogP 3.22) and markedly improved both solubility (11.2 μM) and microsomal stability (t_1/2_ = 47.7 min; Cl_int_ = 15 μL/min/mg). Although potency remained moderate (EC_50_ = 0.31 and 0.331 μM), the compound achieved well-balanced LE values (0.41–0.40), making it a favorable reference for optimizing drug-like properties within the series.

Among the most potent analogues, **BE5027** and **BE5049** emerged as high-efficiency ligands. **BE5027** demonstrated low-nanomolar potency (EC_50_ = 0.001 and 0.007 μM) with outstanding LE (0.57–0.58) and excellent solubility (66 μM). However, it suffered from rapid metabolic clearance (t_1/2_ = 11.7 min; Cl_int_ = 59 μL/min/mg), indicating a clear potency–stability trade-off. In contrast, **BE5049** maintained comparable potency (EC_50_ = 0.010 and 0.007 μM) and strong LE (0.49–0.50) while exhibiting moderate solubility (7.1 μM) and improved microsomal stability (t_1/2_ = 39.3 min; Cl_int_ = 18 μL/min/mg), representing the most balanced profile in the series.

**BE5040** exhibited high potency toward both ERRα and ERRγ, together with intermediate aqueous solubility (3.0 μM) and moderate metabolic stability (t_1/2_ = 28.5 min). In a similar manner, the closely related analogues **BE5066** and **BE5086** demonstrated good potency and ligand efficiency (LE = 0.43–0.46). **BE5066** showed moderate solubility (2.3 μM), whereas **BE5086** displayed substantially higher solubility (46.4 μM). Despite these differences, both compounds exhibited short microsomal half-lives and relatively high lipophilicity (cLogP = 4.39 and 4.73, respectively). Notably, the primary structural distinction between **BE5086** and the other analogues is the presence of an α-naphthyl substituent at ring B, rather than the β-naphthyl motif found in **BE5040** and **BE5066**, which may contribute to the improved solubility observed for **BE5086**.

The previously reported lead **BE5082** retained its strong potency (EC_50_ = 0.036 and 0.031 μM) and high LE (0.55–0.58) in the current study. Its moderate solubility (3.1 μM) and stability (t_1/2_ = 18.1 min; Cl_int_ = 38 μL/min/mg) reinforce its potential as a potent yet metabolically sensitive scaffold suitable for further optimization through polarity tuning or steric shielding.

Importantly, **BE5112**, exhibited robust potency and good LE (0.43), accompanied by a notably high solubility (21.7 μM). However, its extremely short microsomal half-life (t_1/2_ = 6.6 min; Cl_int_ = 105 μL/min/mg) suggests rapid oxidative metabolism, likely due to unshielded benzylic or heteroaryl positions. Despite this, **BE5112** stands out as a valuable lead for structural modifications aimed at reducing clearance while maintaining its favorable solubility and potency balance.

Finally, **BE5138** displayed good potency (EC_50_ = 0.1 and 0.12 μM) with high LE values (0.49–0.50), but was limited by poor aqueous solubility (1.1 μM) and moderate metabolic stability (t_1/2_ = 22.0 min; Cl_int_ = 31 μL/min/mg). Despite its favorable efficiency, the low solubility highlights an opportunity for polarity-enhancing substitutions to improve exposure.

Overall, these results highlight clear structure–property relationships across the series. Compounds **BE5049** and **BE5027** exhibit the best potency and LE but require stabilization to improve metabolic profiles. **BE5112** offers an excellent solubility benchmark, whereas **BE2180** provides a favorable DMPK profile at the cost of potency. Together, these compounds outline complementary optimization paths, improving metabolic stability for the most potent analogs, and enhancing potency for the most stable ones to guide future lead refinement.

### 2.4. Computational studies

#### 2.4.1. Binding free energy

Molecular Dynamics (MD) simulations were performed for five selected ligands, **BE5112**, **BE5138**, **BE5040**, **BE5049**, and **SLU-PP-332**, binding to ERRα. Effective binding energy Δ*G_bind_* and its energy components were calculated for **BE5112**, **BE5138**, and **SLU-PP-332** complexes using a total of 4000 snapshots from a 400 ns long MD simulation. Here, energies of Van der Waal Δ*G_VDW_* interactions and electrostatic interactions Δ*G_EEL_*, solvation energy Δ*G_solv_*, and total free binding energy Δ*G_bind_* are reported with standard errors (SE) in Table 7. Among the three ligands, **SLU-PP-332** displays the most favorable effective binding energy to ERRα (Δ*G_bind_* = −35.43 ± 0.08 kcal/mol), driven primarily by a substantially larger negative Δ*G_EEL_* (−28.43 ± 0.13 kcal/mol) relative to the other compounds. **BE5112** exhibits the least favorable Δ*G_bind_* of −33.96 ± 0.05 kcal/mol, whereas **BE5138** was modestly more favorable (≈−0.4 kcal/mol) than **BE5112**. The maximum difference in Δ*G_bind_* across the three ligands is quite small (≈1.5 kcal/mol). Notably, **BE5112** produces the largest Δ*G_VDW_* (−44.18 ± 0.05 kcal/mol) among the set.

#### 2.4.2. Protein-ligand contact analysis

**Fig. 10.**
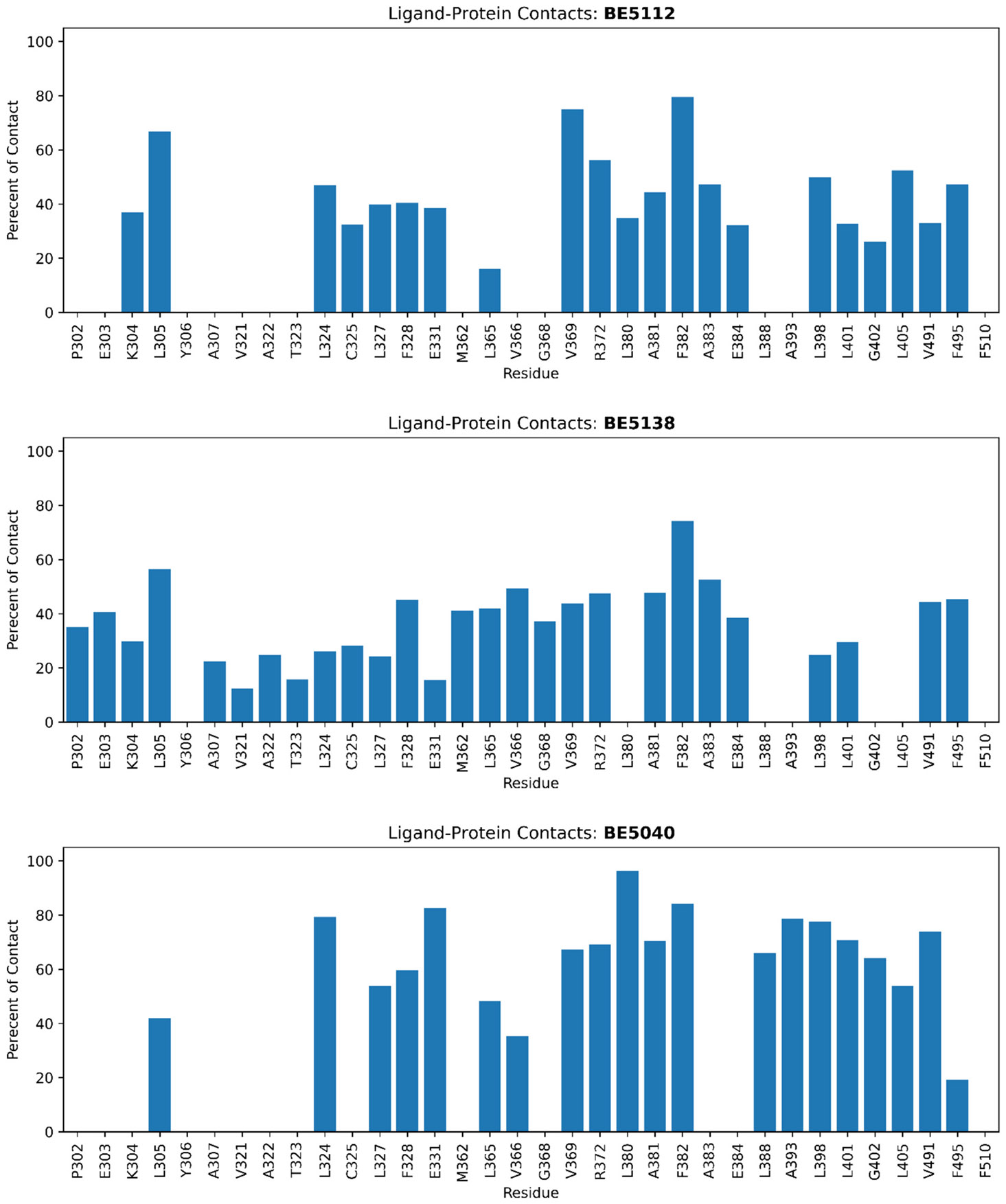

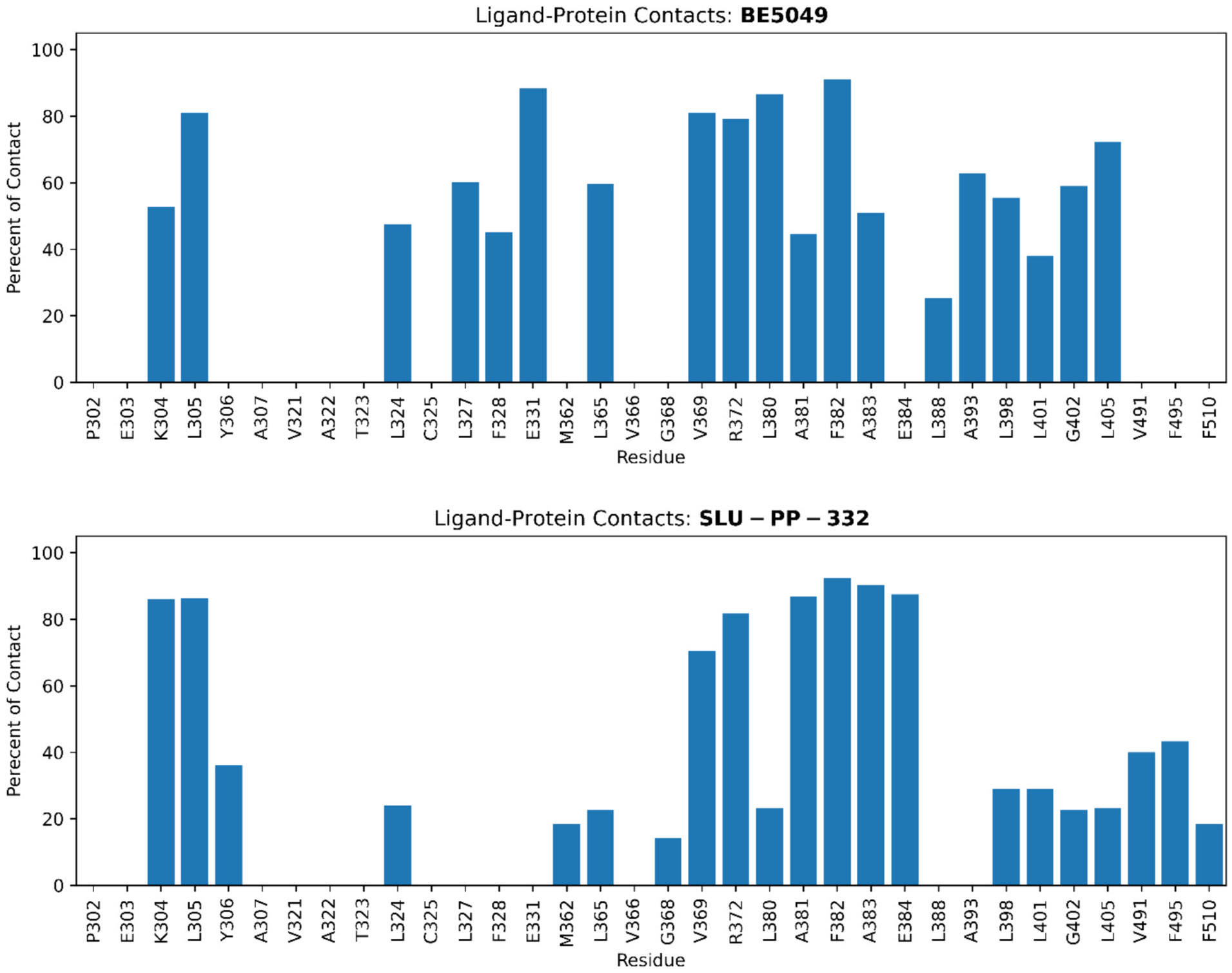
Ligand-protein contact frequencies of **BE5112**, **BE5138**, **BE5040**, **BE5049**, and **SLU-PP-332** in complex with ERRα from MD simulations.

**Fig. 11.**
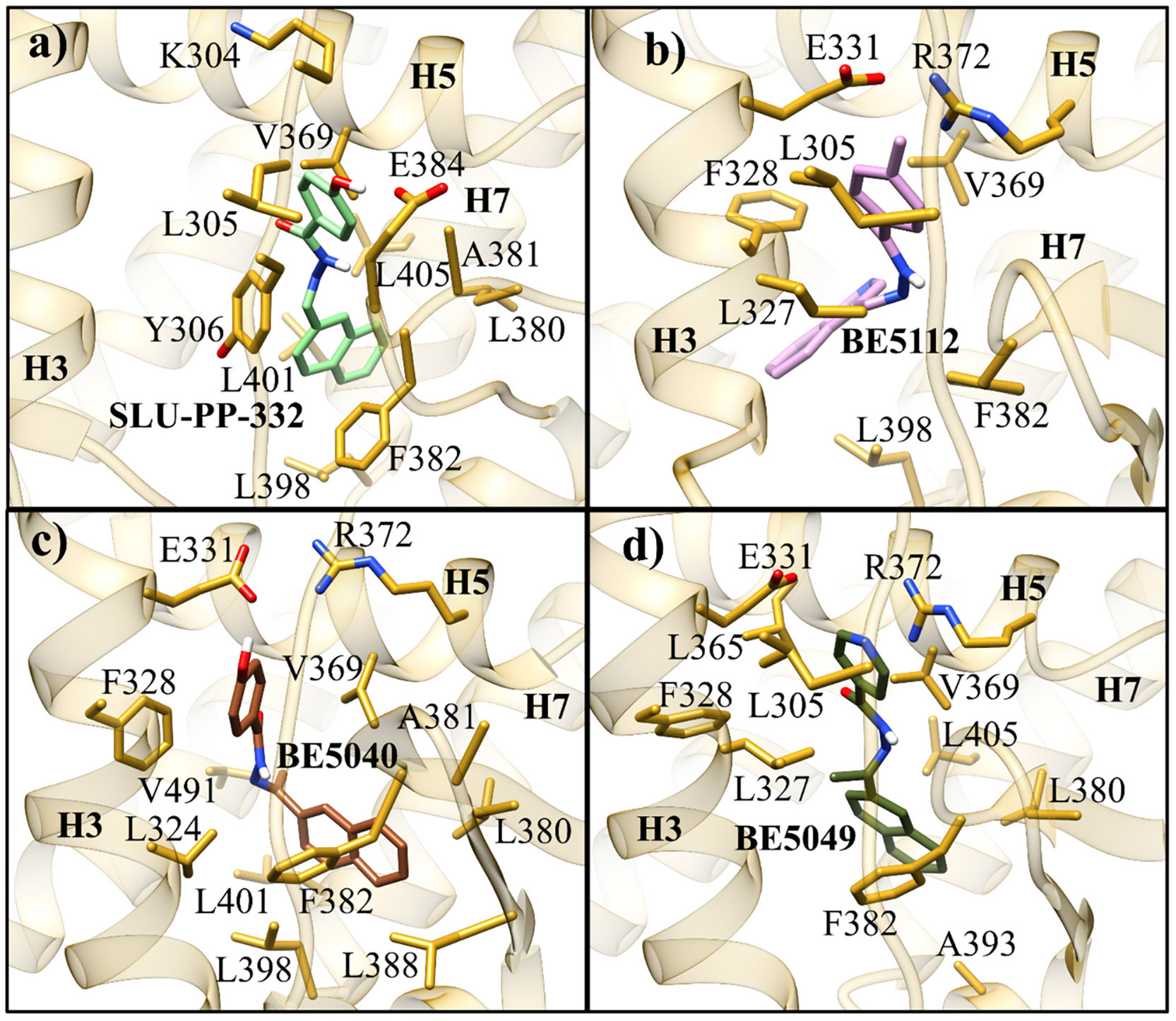
Representative conformations of a) **SLU-PP-332**, b) **BE5112**, c) **BE5040**, and d) **BE5049** complexes with ERRα obtained from MD simulations.

To further investigate the interactions between ligands and the receptor, we performed protein-ligand contact analysis (Fig. 10). For ERRα, all five ligands have consistently high contacts frequencies with Leu305, Val369, Ala381, and Phe382, indicating stable engagement with key residues in the LBP. Residue Glu384 displays stronger and more persistent interactions with **SLU-PP-332** than with the other ligands, likely through hydrogen bonding mediated by its hydroxyl group (Fig. 11). This additional polar contact, together with the ligand’s dynamic binding behavior, may account for the more favorable Δ*G_EEL_* but lest favorable Δ*G_VDW_* observed for **SLU-PP-332**.

In contrast, the absence of hydroxyl group in **BE5138** and **BE5112** allows these ligands to shift slightly away from Glu384 while maintaining hydrogen bonding with the backbone of Phe382 (Figs. 11 and S133). Both compounds adopt a “sandwich-like” conformation between Phe328 and Phe382, promoting extensive π-π and hydrophobic contacts. The absence of the Glu384 H-bond confers additional conformational flexibility, permitting tighter packing surrounding hydrophobic residues and thus yielding more favorable Δ*G_VDW_* values of **BE5138** and **BE5112** compared to **SLU-PP-332**. The isoquinoline ring of **BE5112**, with its extended π-system and a nitrogen atom that subtly polarizes the ring, likely strengthens dispersive interactions with the nearby aromatic residues, explaining its particularly favorable Δ*G_VDW_* for ERRα.

Interestingly, the dichloro substituents on the aromatic ring of **BE5138** may increase the ring’s overall polarizability and local electron-withdrawing character, enhancing partial negative charge density around the chlorine atoms. This could strengthen electrostatic interactions with positively polarized residues, contributing to its more favorable Δ*G_EEL_* relative to **BE5112**.

The structural modification in **BE5040**, characterized by an additional methyl group relative to **SLU-PP-332**, enables the ligand to form a persistent hydrogen bond with the carboxylate side chain of Glu331. This interaction serves to anchor the hydroxyl head group proximal to Glu331, consequently preventing the ligand’s ─NH group from engaging in a hydrogen bond with the backbone of Phe382.

Conversely, **BE5049** incorporates a pyridine head group in place of the phenolic ring in **BE5040**. This compound retains the hydrogen bond between its ─NH group and the backbone of Phe382, as observed for **BE5112** and **BE5138**. However, the sterically shorter pyridine moiety is incapable of reaching Glu331 to form the hydrogen bond present in **BE5040**. Instead, it preferentially forms a hydrogen bond with the backbone of Leu305.

Furthermore, the methyl group adjacent to the naphthalene ring, a feature common to both **BE5040** and **BE5049**, reduces the conformational flexibility of the ligands. This restriction helps to stabilize the orientation of the naphthalene moiety within the allosteric binding site of ERRα.

### 2.5. qPCR and RNA-seq profiling of ERR agonists

**Fig. 12.**
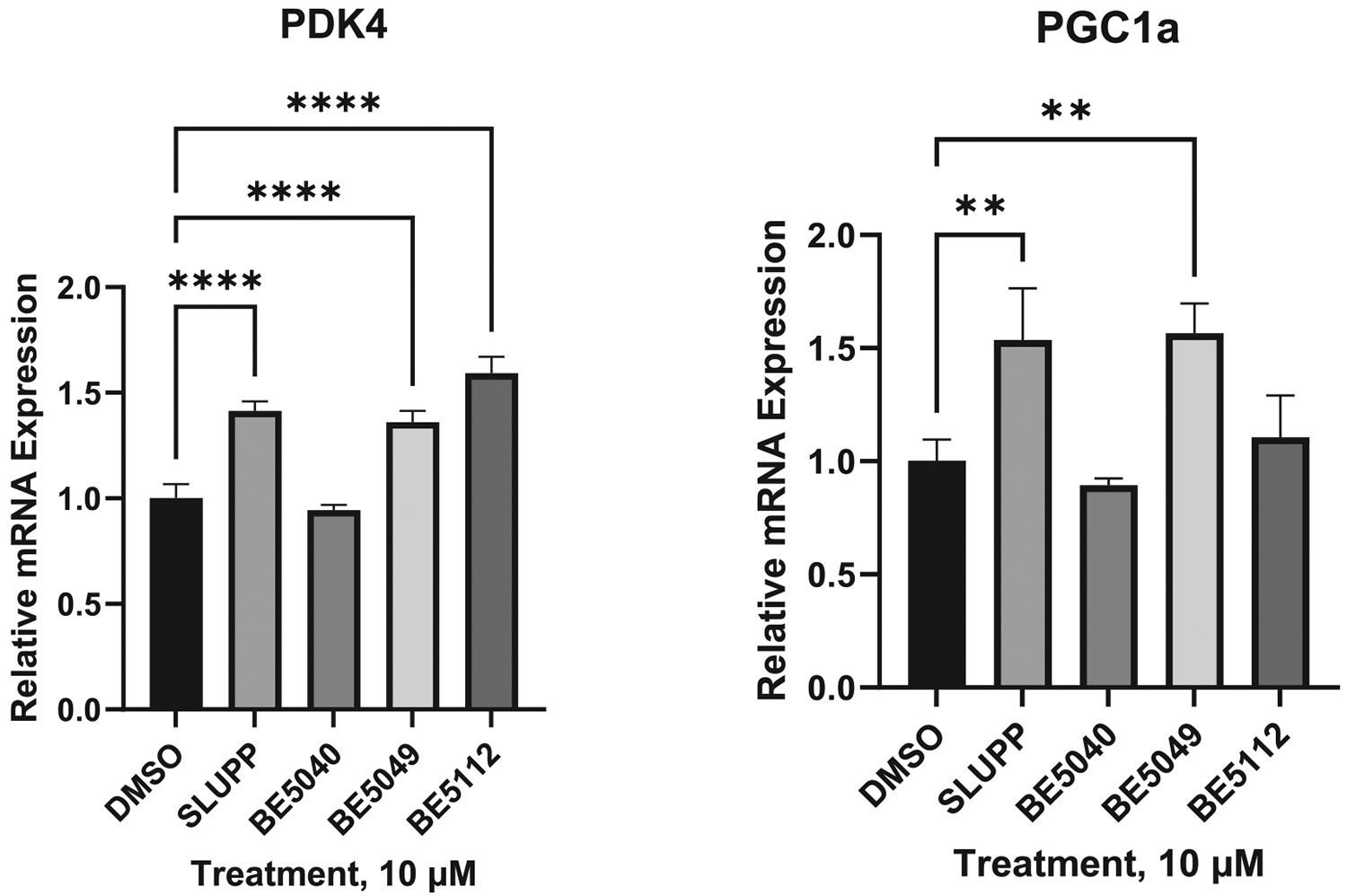
qPCR analysis of PDK4 (A) and PGC1α (B) mRNA expression in C2C12 myotubes treated with **SLU-PP-332**, **BE5040**, **BE5049**, and **BE5112** (10 μM, 24 h). Data are normalized to DMSO control and presented as mean ± SEM. Statistical significance is indicated for comparisons versus DMSO (PDK4: ****p < 0.0001; **PGC1α: *p* < 0.01).

To complement the SAR and reporter assay data, we assessed downstream transcriptional outputs in two complementary biological systems: (i) a rapid, targeted qPCR readout in C2C12 myotubes (skeletal muscle model; 24 h) to validate canonical ERR-responsive markers, and (ii) an unbiased transcriptome-wide analysis in NRVMs (primary cardiac model; 72 h) to evaluate pathway-level remodeling of mitochondrial metabolism. qPCR analysis of canonical ERR target genes PDK4 and PGC-1α revealed that **SLU-PP-332** significantly increased PDK4 mRNA levels relative to DMSO (Fig. 12A). **BE5049** and **BE5112** elicited comparable or greater induction of PDK4, with **BE5112** producing the highest response (~1.7-fold), consistent with its high efficacy in reporter assays. **SLU-PP-332** again produced a significant increase in PGC-1α relative to DMSO, and **BE5049** induced a robust elevation (~1.6-fold) (Fig. 12B). **BE5112** caused a non-significant increase in PGC-1α mRNA, suggesting gene- and context-dependent differences in transcriptional output rather than reduced intrinsic efficacy. In contrast, **BE5040** showed no detectable induction in both genes, despite its activity in the reporter assays.

Although qPCR was performed on the most recent series of optimized ligands, our RNA-seq studies were initiated earlier in the project and therefore focused on two high-priority chemotypes emerging at that stage: **BE2180** and **BE5112**. Importantly, **BE5112** is also included in the qPCR experiments and demonstrated strong PDK4 induction, allowing it to serve as a molecular bridge between the two datasets. **BE2180**, while not part of the qPCR set, was selected for transcriptomics because (i) it demonstrated potent ERR activation in the reporter assays, (ii) it represented an important early structural branch within ring B SAR (quinoline-2-yl series).

**SLU-PP-332** has been shown to be effective in the mouse transverse aortic constriction (TAC) model of reduced ejection fraction heart failure [26]. The compound has in vivo efficacy demonstrated by increasing the expression of ERR target genes in the heart leading to improved ejection fraction and contractile function while reducing cardiac stress and fibrosis. In neonatal rat ventricular myocytes (NRVMs), **SLU-PP-332** consistently upregulates genes within oxidative phosphorylation, the tricarboxylic acid (TCA) cycle, and fatty acid degradation pathways [27]. Increased mitochondrial biogenesis and respiration in myocytes induced by this compound have been reported [28,29].

**Fig. 13.**
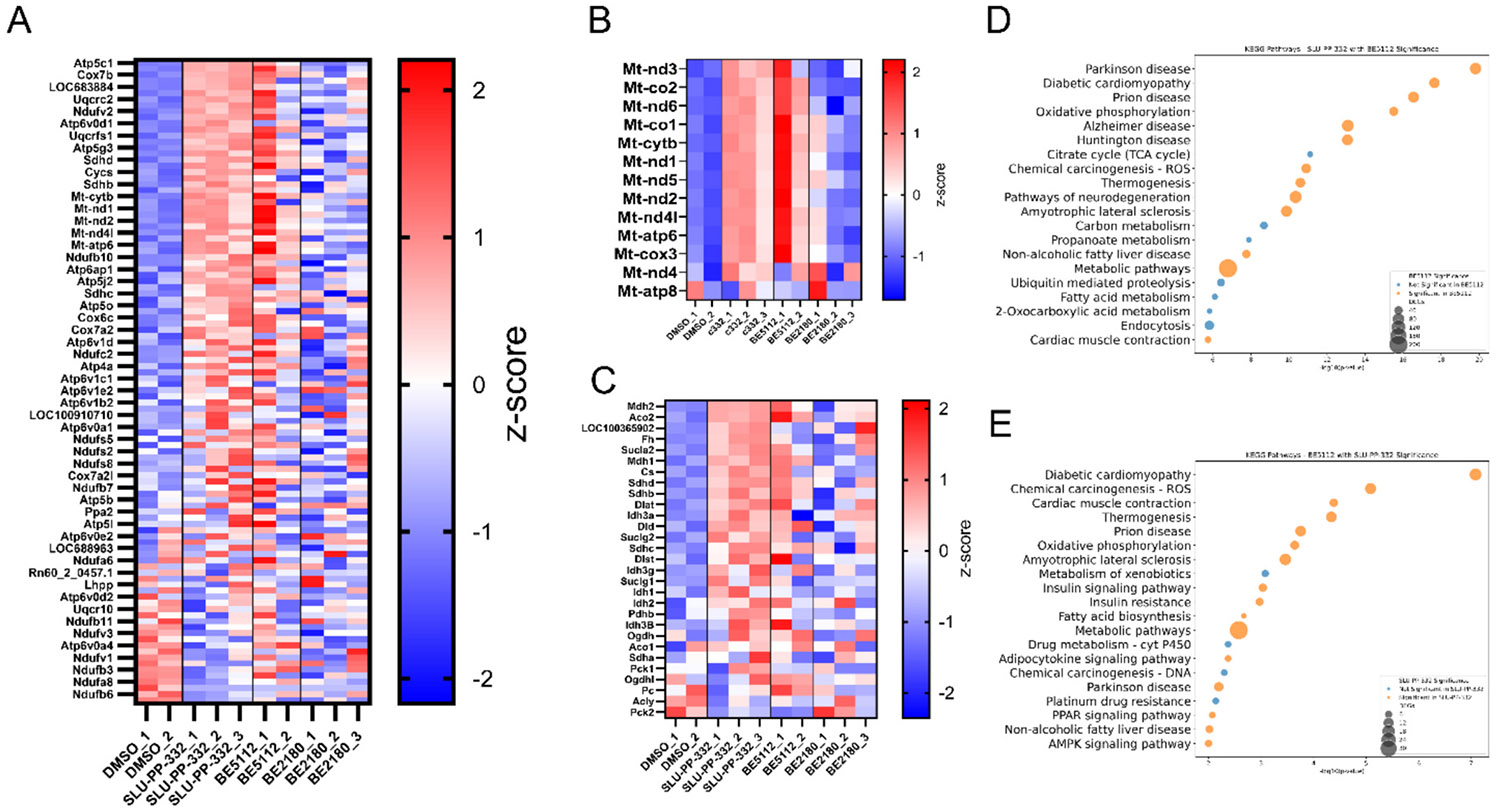
KEGG pathway heatmaps and enrichment analyses for NRVMs treated with **SLU-PP-332**, **BE5112**, or **BE2180** for 72 h. Gene-wise z-scores were computed from FPKM values for visualization purposes only across retained samples following quality control. Differential expression and pathway enrichment analyses were performed using raw count data with DESeq2-based normalization. Heatmaps show oxidative phosphorylation (A), mitochondrial genes (B), and TCA cycle (C). Dot plots display −log10(*p*-value) of top 20 KEGG pathways enriched among upregulated genes for **SLU-PP-332** (D) and **BE5112** (E) (Orange: Significant in **BE5112**/**SLU-PP-332**; Blue: Not significant in **BE5112**/**SLU-PP-332**; p-value ≤0.05).

To compare the transcriptional effects of new ERR ligands, NRVMs were treated for 72 h with **BE5112** or **BE2180** followed by transcriptome sequencing (RNA-seq). Following standard quality control, one replicate from the DMSO group and one from the **BE5112** group were excluded as outliers based on principal component analysis and poor global expression concordance. **BE5112** closely recapitulates the gene induction observed with **SLU-PP-332** across KEGG oxidative phosphorylation and TCA cycle pathways, as well as mitochondrial gene sets (Fig. 13A–C). Presumably, the mitochondrial encoded genes are modulated by the ERR nuclear receptor indirectly via regulation of key genes that are involved in mitochondrial biogenesis, which are well known ERR target genes. In contrast, **BE2180** shows limited overlap with **SLU-PP-332** or **BE5112** but does still active some pathways known to be response to ERR (i.e., fatty acid degradation pathway). KEGG pathway analysis reveals concordant upregulation of cellular energetics and fatty acid metabolism pathways with both **SLU-PP-332** and **BE5112** (Fig. 13D–E).

Although the highlighted pathways are clearly associated with activation of ERR we also examined the data for well-characterized ERR target genes more closely. *Aco2*, *Ldha*, and *Cpt1b* are significantly upregulated by treatment with both compounds. *Aco2*, a direct ERRγ target and TCA cycle enzyme, regulates oxidative phosphorylation and mitochondrial function [30]. *Ldha* is involved in the adaptation of metabolic processes and is a direct target gene of ERRα [31]. *Cpt1b*, essential for mitochondrial fatty acid oxidation in cardiac and skeletal muscle, exhibits ERRγ occupancy in cardiomyocytes [32–34]. These data are consistent with **BE5112** functioning in a similar manner to **SLU-PP-332** as an ERR agonist.

In contrast, **BE2180**, despite showing comparable binding potency, exhibited much lower efficacy in cell-based assays. This reduced transcriptional activity may reflect suboptimal receptor engagement or poor coactivator recruitment efficiency, resulting in limited downstream gene activation. Additionally, differences in cellular permeability, metabolic stability, or nuclear localization could restrict its effective intracellular concentration, thereby diminishing its functional response. Thus, under the NRVM RNA-seq conditions used here, **BE2180** behaves as a weak functional agonist, despite its strong ERR engagement in HEK293 reporter assays.

Despite the comprehensive structure–activity relationship analysis integrating chemical synthesis, cell-based reporter assays, transcriptomic profiling, ADME evaluation, and computational modeling, several limitations should be acknowledged. First, biological validation was restricted to in vitro systems (HEK293 cells, C2C12 myotubes, and NRVMs), which may not fully recapitulate the complexity of ERR signaling, tissue distribution, and pharmacodynamics in vivo. Second, transcriptomic analyses were performed on a limited subset of compounds and in a single primary cell type, potentially constraining the generalizability of pathway-level conclusions across chemotypes and tissues. Third, while molecular docking and molecular dynamics simulations provided mechanistic insight into ligand–receptor interactions, these approaches rely on available ERRα crystal structures and force-field assumptions that may not capture all relevant conformational states. Future studies will address these gaps through in vivo validation, expanded transcriptomic profiling, and comprehensive pharmacokinetic and safety assessment to define the translational potential of optimized ERR agonists.

## 3. Conclusions

In this study, we present the first systematic SAR investigation of the **SLU-PP-332** chemotype, a benchmark ERR agonist that has been widely adopted to probe ERR-dependent metabolic and exercise-responsive biology. Despite its extensive biological use, the structural basis underlying the activity and developability of **SLU-PP-332** had not been previously defined. This work addresses that gap by mapping how specific structural features govern ERR agonism, isoform selectivity, transcriptional efficacy, ligand efficiency, and drug-like properties. Our findings demonstrate that (i) ring B architecture is the dominant driver of agonist activity, with isoquinoline-4-yl and naphth-1-yl motifs yielding the most favorable profiles; (ii) ring A electronics and hydrazone substitutions tune isoform selectivity and ligand efficiency; and (iii) the carbonyl group is indispensable, with sulfonyl substitution abolishing activity.

Computational analyses further illuminate the mechanistic basis for these trends. Productive “sandwich-like” positioning between Phe328/Phe382, combined with context-dependent hydrogen-bonding and polar contacts, appears to differentiate ligand binding energetics and may explain efficacy differences observed across the series. Functionally, **BE5112** closely recapitulated **SLU-PP-332**–driven upregulation of oxidative phosphorylation and TCA-cycle pathways in NRVMs, indicating strong engagement of ERR-dependent metabolic programs and validating it as the strongest next-generation lead.

Across functional assays, in vitro ADME evaluation, docking, and MD simulations, **BE5112** and **BE5049** emerged as potent agonists, with **BE5049** exhibiting good metabolic stability, capable of robust target-gene activation. Together, these insights establish a foundation for the rational optimization of ERR agonists with improved potency, isoform selectivity, and pharmacokinetic properties, advancing the development of exercise-mimetic therapeutics.

Collectively, this work establishes design principles for tuning ERR agonist behavior rather than defining optimization solely by maximal activity in a single assay. By disclosing the SAR landscape surrounding **SLU-PP-332**, we deliver an expanded and mechanistically informed toolkit of ERR agonists and lay a foundation for the rational development of next-generation exercise mimetics with tailored biological and pharmacokinetic properties. More broadly, these findings advance understanding of how small-molecule ERR agonists can be engineered to differentially modulate metabolic transcriptional programs in a context-dependent manner.

## 4. Experimental

### 4.1. Chemistry

All materials were purchased from commercial suppliers and used without further purification. The purities of the final compounds were characterized by high-performance liquid chromatography (LC/MS) using a gradient elution program (Ascentis Express Peptide C18 column, acetonitrile/water 5/95/95/5, 5 min, 0.05% formic acid) and UV detection (254 nM). The purities of the final compounds were 95% or greater. Melting points of the compounds were recorded with Reach Devices (RD-MP) digital melting point determination apparatus. NMR spectra were recorded on a Bruker NMR 400 MHz Avance III spectrometer operating at 400 MHz for ^1^H NMR and 100 MHz for ^13^C NMR. Chemical shifts are given in part per million (ppm) relative to tetramethyl silane (TMS) and coupling constants *J* are given in Hertz.

#### 4.1.1. General procedures

##### 4.1.1.1. General procedure for the preparation of hydrazides (2).

Appropriate carboxylic acids (5.0 mmol) were heated under reflux in methanol (5.0 mL) for 2 h in presence of conc. H_2_SO_4_ (catalytic amount) with continuous stirring. The reaction was monitored by TLC till the acids were fully converted to the corresponding esters [Eluent:EtOAc/Hexanes(1:4)]. The reaction mixture was allowed to cool down to room temperature and hydrazine monohydrate 80% (20 mmol, 0.96 mL) was added slowly in an ice bath. The reaction was then warmed to room temperature and heated under reflux for another 1–2 h and followed by TLC till formation of hydrazide. The reaction mixture was kept at refrigerator till the product precipitated. All hydrazides were isolated in quantitative yields and in a pure form.

##### 4.1.1.2. General procedure for the preparation of N-acyl hydrazones (3a and 3b).

A mixture of the appropriate hydrazides 2 (1 mmol) and the appropriate (aldehydes / Acetophenones) (1.1 mmol) was refluxed in ethanol until the condensation was complete (monitored by TLC, 1–3 h). The reaction was allowed to cool to room temperature, and the formed precipitate was filtered and washed with cold ethanol. The purity of most compounds was ≈98% and in few cases the obtained solid was recrystallized from ethanol to give the desired product.

##### 4.1.1.3. General procedure for the preparation of N-sulphonyl hydrazones (6).

Hydrazine monohydrate (3.0 mmol) was added drop wise into the solution of corresponding sulfonyl chloride **(4)** (1.0 mmol) in THF (15.0 mL) at 0 °C. subsequently, the mixture was further stirred at 0 ° C for 30 min. After the completion of the reaction, the solvent was removed by evaporation, and the residue was extracted with dichloromethane (3 × 10.0 mL), and the combined organic layer was washed with water, and brine, and dried over Na_2_SO_4_. Concentration in vacuum gave the desired product **(5)** in yields range from 70 to 95%. The appropriate sulfono-hyhydrazides **(5)** (1.0 mmol) and appropriate aldehydes (1.0 mmol, 1.0 equiv.) were refluxed in absolute ethanol (6.0 mL) for 2–3 h, The formed precipitate was filtered off, washed with ethanol, dried, and recrystallized from EtOH to give N-sulphonyl hydrazone derivatives **(6)**. All acyl hydrazones reported here are known compounds their identity was confirmed by comparing their melting points and mass to literature (See supporting information). ^1^H and ^13^C NMR spectra of most active compounds are described here.

###### 4.1.1.3.1. (E)-4-Hydroxy-N′-(naphthalen-2-ylmethylene)benzohydrazide (SLU-PP-332) [35].

White solid, mp: 223–228 °C, yield: 192 mg (89%). ^1^H NMR (500 MHz, DMSO-*d*_6_) *δ* 11.80 (s, 1H), 10.19 (d, *J* = 3.5 Hz, 1H), 8.62 (s, 1H), 8.12 (s, 1H), 8.03–7.86 (m, 6H), 7.54 (dq, *J* = 6.8, 3.6 Hz, 2H), 6.92 (dq, *J* = 9.5, 2.4 Hz, 2H). LC/MS *m/z*: 291.1 [M + H]^+^.

###### 4.1.1.3.2. (E)-4-Hydroxy-N′-(quinolin-2-ylmethylene)benzohydrazide (BE2180) [36].

White solid, mp: 226–229.3 °C, Yield 124 mg (78%), ^1^H NMR (500 MHz, DMSO-*d*_6_) *δ* 11.75 (s, 1H), 8.60 (s, 1H), 8.11 (s, 1H), 8.04–7.89 (m, 4H), 7.89–7.79 (m, 2H), 7.55 (dt, *J* = 6.2, 3.4 Hz, 2H), 6.93–6.83 (m, 2H). LC/MS *m/z*: 292.1 [M + H]^+^.

###### 4.1.1.3.3. (E)-4-Hydroxy-N′-(Isoquinolin-3-ylmethylene)benzohydrazide (BE21465).

White solid, mp: 191–196 °C, yield 220 mg (87%). ^1^H NMR (500 MHz, DMSO-*d*_6_) *δ* 11.88 (s, 1H), 10.18 (d, *J* =4.5 Hz, 1H), 9.35 (d, *J* = 4.3 Hz, 1H), 8.65 (s, 1H), 8.40 (s, 1H), 8.21–8.04 (m, 2H), 7.93–7.75 (m, 3H), 7.70 (q, *J* = 6.5, 5.8 Hz, 1H), 6.90 (dd, *J* = 8.3, 4.3 Hz, 2H). ^13^C{^1^H} NMR (126 MHz, DMSO-*d*_6_) *δ* 168.41, 168.09, 163.37, 161.31, 152.89, 147.76, 147.53, 135.87, 131.59, 130.30, 128.76, 128.71, 128.13, 127.84, 124.19, 117.21, 115.55. LC/MS *m/z*: 292.1 [M + H]^+^.

###### 4.1.1.3.4. (E)-4-Hydroxy-N′-(quinolin-3-ylmethylene)benzohydrazide (BE5110).

Yellow solid, mp: 187–194 °C, yield 103 mg (81%). ^1^H NMR (500 MHz, DMSO-*d*_6_) *δ* 11.99 (s, 1H), 9.39 (s, 1H), 8.76 (s, 1H), 8.62 (s, 1H), 8.17–8.05 (m, 2H), 7.91–7.80 (m, 3H), 7.73 (t, *J* = 7.6 Hz, 1H), 6.89 (d, *J* = 8.2 Hz, 2H). ^13^C{^1^H} NMR (126 MHz, DMSO-*d*_6_) *δ* 161.45, 159.23, 158.94, 147.29, 144.74, 137.67, 132.21, 130.35, 129.38, 128.69, 128.63, 128.11, 126.67, 123.95, 117.19, 115.56, 114.88. LC/ MS *m/z*: 292.1 [M + H]^+^.

###### 4.1.1.3.5. (E)-4-Hydroxy-N′-(quinolin-6-ylmethylene)benzohydrazide (BE5116)

Yellow solid, mp: 191–198 °C, yield: 118 mg (74%). ^1^H NMR (500 MHz, DMSO-*d*_6_) *δ* 11.89 (s, 1H), 10.27 (s, 1H), 9.07 (dd, *J* = 4.6, 1.6 Hz, 1H), 8.74 (d, *J* =8.3 Hz, 1H), 8.64 (s, 1H), 8.33 (s, 2H), 8.16 (d, *J* =9.5 Hz, 1H), 7.85 (dd, *J* = 8.5, 1.4 Hz, 2H), 7.78 (dd, *J* = 8.4, 4.6 Hz, 1H), 6.89 (dd, *J* = 8.6, 1.3 Hz, 2H). ^13^C{^1^H} NMR (126 MHz, DMSO-*d*_6_) *δ* 161.34, 159.04, 158.75, 149.52, 145.86, 140.61, 134.34, 130.28, 128.77, 128.73, 128.66, 127.15, 124.14, 122.83, 117.31, 115.54, 114.99. LC/MS *m/z*: 292.1 [M + H]^+^.

###### 4.1.1.3.6. (E)-4-Hydroxy-N′-(quinoxalin-2-ylmethylene)benzohydrazide (BE5070).

Gray solid, mp 119–128 °C, yield 98 mg (65%). ^1^H NMR (500 MHz, DMSO-*d*_6_) *δ* 12.15 (s, 1H), 10.24 (s, 1H), 9.42 (s, 1H), 8.67–8.48 (m, 1H), 8.14–8.07 (m, 2H), 7.86 (ddd, *J* = 8.6, 6.2, 2.1 Hz, 4H), 6.92–6.86 (m, 2H). ^13^C{^1^H} NMR (126 MHz, DMSO-*d*_6_) *δ* 219.87, 169.54, 169.49, 161.56, 149.11, 143.29, 141.98, 141.74, 131.26, 131.05, 130.51, 129.52, 129.47, 123.76, 115.62. LC/MS *m/z*: 293.1 [M + H^+^].

###### 4.1.1.3.7. (E)-4-Hydroxy-N′-(naphthalen-1-ylmethylene)benzohydrazide (BE5068).

White solid, mp: 175–181 °C, yield: 114 mg (88%). ^1^H NMR (500 MHz, DMSO-*d*_6_) *δ* 11.77 (s, 1H), 10.20–10.14 (m, 1H), 8.61 (s, 1H), 8.12 (s, 1H), 7.96 (dddd, *J* = 22.3, 9.5, 6.5, 3.3 Hz, 4H), 7.87 (dd, *J* = 8.7, 2.8 Hz, 2H), 7.55 (dp, *J* = 6.7, 3.6 Hz, 2H), 6.91 (dd, *J* = 8.7, 2.8 Hz, 2H). ^13^C{^1^H} NMR (126 MHz, DMSO-*d*_6_) *δ* 167.89, 163.34, 161.22, 147.29, 134.12, 133.37, 132.78, 130.22, 128.93, 128.75, 128.24, 127.48, 127.19, 124.39, 123.18, 115.54. LC/MS *m/z*: 292.1 [M + H]^+^.

###### 4.1.1.3.8. (E)-4-Hydroxy-N′-(isoquinolin-4-ylmethylene)benzohydrazide (BE5113).

Yellow solid, mp: 184–191 °C, yield: 105 mg (71%). ^1^H NMR (500 MHz, DMSO-*d*_6_) *δ* 12.04 (s, 1H), 9.55 (s, 1H), 9.12 (d, *J* = 8.4 Hz, 1H), 8.98 (s, 1H), 8.82 (s, 1H), 8.36 (d, *J* =8.2 Hz, 1H), 8.07 (q, *J* = 7.1 Hz, 1H), 7.89 (dt, *J* = 7.7, 3.3 Hz, 3H), 6.91 (dt, *J* = 9.4, 2.3 Hz, 2H). ^13^C{^1^H} NMR (126 MHz, DMSO-*d*_6_) *δ* 163.46, 161.54, 159.72, 159.44, 159.16, 151.08, 143.86, 138.92, 134.99, 133.62, 130.41, 130.34, 129.60, 128.18, 126.57, 124.98, 123.80. LC/MS *m/z*: 292.1 [M + H]^+^.

###### 4.1.1.3.9. (E)-4-Hydroxy-N′-(quinolin-5-ylmethylene)benzohydrazide (BE5069).

Yellow solid, mp: 189–197 °C, yield 98 mg (63%). ^1^H NMR (500 MHz, DMSO-*d*_6_) *δ* 12.23 (s, 1H), 9.20–9.05 (m, 2H), 8.78 (d, *J* = 8.6 Hz, 1H), 8.18 (dt, *J* = 8.6, 1.6 Hz, 1H), 8.05 (d, *J* = 5.0 Hz, 1H), 7.98 (ddt, *J* = 8.5, 6.9, 1.7 Hz, 1H), 7.88 (ddt, *J* = 14.8, 8.9, 4.6 Hz, 3H), 6.92 (dd, *J* = 8.7, 2.0 Hz, 2H), 4.72 (s, 1H). ^13^C{^1^H} NMR (126 MHz, DMSO-*d*_6_) *δ* 168.09, 167.09, 161.68, 159.09, 158.80, 148.34, 144.75, 142.10, 132.07, 130.56, 129.10, 126.85, 125.44, 123.64, 119.87, 117.48, 115.68. LC/MS *m/z*: 292.1 [M + H]^+^.

###### 4.1.1.3.10. (E)-4-Hydroxy-N′-(quinolin-5-ylmethylene)benzohydrazide (BE5071).

Yellow solid, mp: 194–203 °C, yield 118 (82%). ^1^H NMR (500 MHz, DMSO-*d*_6_) *δ* 11.91 (s, 1H), 9.59 (s, 1H), 9.10–8.94 (m, 2H), 8.71 (d, *J* = 6.2 Hz, 1H), 8.32 (d, *J* = 8.2 Hz, 1H), 8.22 (d, *J* = 7.3 Hz, 1H), 7.93–7.83 (m, 3H), 6.93–6.88 (m, 2H). ^13^C{^1^H} NMR (126 MHz, DMSO-*d*_6_) *δ* 163.37, 161.42, 159.32, 151.52, 145.91, 140.08, 134.43, 133.99, 131.16, 130.24, 129.89, 128.86, 128.80, 124.00, 119.95, 118.12, 115.77. LC/MS *m/z*: 292.1 [M + H]^+^.

###### 4.1.1.3.11. (E)-4-Hydroxy-N′-(isoquinolin-5-ylmethylene)benzohydrazide (BE5117).

White solid, mp: 121–126 °C, yield 106 mg (78%). ^1^H NMR (500 MHz, DMSO-*d*_6_) *δ* 11.96 (s, 1H), 9.66 (s, 1H), 9.14–9.06 (m, 1H), 8.99 (s, 1H), 8.72 (d, *J* = 6.3 Hz, 1H), 8.35 (d, *J* =8.2 Hz, 1H), 8.24 (d, *J* = 7.3 Hz, 1H), 7.88 (dt, *J* = 7.7, 3.4 Hz, 3H), 6.97–6.84 (m, 2H). ^13^C {^1^H} NMR (126 MHz, DMSO-*d*_6_) *δ* 163.39, 161.46, 159.40, 151.02, 145.85, 139.01, 134.96, 134.26, 131.38, 130.26, 129.98, 129.16, 128.74, 123.96, 120.45, 118.00, 115.60. LC/MS *m/z*: 292.1 [M + H]^+^.

###### 4.1.1.3.12. (E)-N′-Benzylidene-4-methylbenzohydrazide (BE5082) [18].

White solid, mp: 132–144 °C, yield: 114 mg (84%). ^1^H NMR (500 MHz, DMSO-*d*_6_) *δ* 11.78 (s, 1H), 8.47 (s, 1H), 7.84 (d, *J* = 7.8 Hz, 2H), 7.73 (d, *J* = 7.2 Hz, 2H), 7.46 (d, *J* = 8.4 Hz, 3H), 7.34 (d, *J* = 7.7 Hz, 2H), 2.38 (s, 3H). LC/MS *m/z*: 239.1 [M + H]^+^.

###### 4.1.1.3.13. (E)-4-Methyl-N′-(naphthalen-2-ylmethylene)benzohydrazide (BE2181) [37].

White solid, mp 201–203.2 °C, yield: 152 mg (87%). ^1^H NMR (500 MHz, DMSO-*d*_6_) *δ* 11.87 (s, 1H), 8.62 (s, 1H), 8.15 (s, 1H), 8.03–7.92 (m, 4H), 7.87 (d, *J* = 7.8 Hz, 2H), 7.57 (dd, *J* = 6.2, 3.2 Hz, 2H), 7.35 (d, *J* = 7.9 Hz, 2H), 2.39 (s, 3H). LC/MS *m/z*: 289.1 [M + H]^+^.

###### 4.1.1.3.14. (E)-4-Methyl-N′-(quinolin-2-ylmethylene)benzohydrazide (BE2182) [37].

White solid, mp: 187–189.6 °C, Yield: 128 mg (84%). ^1^H NMR (500 MHz, DMSO-*d*_6_) *δ* 12.13 (s, 1H), 8.62 (s, 1H), 8.42 (d, *J* = 8.6 Hz, 1H), 8.13 (d, *J* =8.6 Hz, 1H), 8.05 (dd, *J* = 8.4, 2.5 Hz, 1H), 8.01 (d, *J* = 8.0 Hz, 1H), 7.87 (d, *J* = 7.7 Hz, 2H), 7.79 (tq, *J* = 8.4, 1.5 Hz, 1H), 7.63 (td, *J* = 7.4, 2.5 Hz, 1H), 7.36 (dd, *J* = 8.2, 2.6 Hz, 2H), 2.39 (d, *J* = 2.7 Hz, 3H). LC/MS *m/z*: 290.1 [M + H]^+^.

###### 4.1.1.3.15. (E)-N′-(Isoquinolin-3-ylmethylene)-4-methylbenzohydrazide (BE21466).

White solid, mp: 206–210 °C, yield 187 mg (91%). ^1^H NMR (500 MHz, DMSO-*d*_6_) *δ* 12.01 (s, 1H), 9.36 (s, 1H), 8.67 (s, 1H), 8.42 (s, 1H), 8.13 (dd, *J* = 14.3, 8.2 Hz, 2H), 7.88 (d, *J* = 7.8 Hz, 2H), 7.81 (t, *J* =7.6 Hz, 1H), 7.71 (t, *J* = 7.5 Hz, 1H), 7.35 (d, *J* = 7.9 Hz, 2H), 2.38 (s, 3H). ^13^C{^1^H} NMR (126 MHz, DMSO-*d*_6_) *δ* 177.81, 167.18, 163.63, 152.92, 148.49, 147.45, 142.45, 135.85, 131.56, 130.90, 129.52, 128.82, 128.74, 128.23, 128.11, 127.85, 117.38, 21.52. LC/MS *m/z*: 290.1 [M + H]^+^.

###### 4.1.1.3.16. (E)-4-Methyl-N′-(quinolin-3-ylmethylene)benzohydrazide (BE5109).

Yellow solid, mp: 191–196 °C, yield: 108 mg (78%). ^1^H NMR (500 MHz, DMSO-*d*_6_) *δ* 12.09 (s, 1H), 9.38 (s, 1H), 8.79–8.66 (m, 2H), 8.18–8.08 (m, 2H), 7.87 (ddd, *J* = 8.7, 4.8, 2.3 Hz, 3H), 7.75–7.69 (m, 1H), 7.34 (d, *J* = 8.0 Hz, 2H), 2.38 (s, 3H). ^13^C{^1^H} NMR (126 MHz, DMSO-*d*_6_) *δ* 163.61, 159.01, 158.72, 147.97, 146.27, 144.94, 142.52, 136.78, 131.74, 130.77, 129.51, 129.32, 128.37, 128.22, 127.80, 117.02, 114.72, 21.50. LC/MS *m/z*: 290.1 [M + H]^+^.

###### 4.1.1.3.17. (E)-4-Methyl-N′-(quinolin-6-ylmethylene)benzohydrazide (BE5115).

Yellow solid, mp: 189–193 °C, yield: 102 mg (83%). ^1^H NMR (500 MHz, DMSO-*d*_6_) *δ* 12.02 (s, 1H), 9.06 (d, *J* = 4.5 Hz, 1H), 8.78–8.61 (m, 2H), 8.33 (d, *J* = 7.7 Hz, 2H), 8.15 (d, *J* = 8.9 Hz, 1H), 7.93–7.72 (m, 3H), 7.34 (d, *J* = 7.5 Hz, 2H), 2.38 (d, *J* = 2.9 Hz, 3H). ^13^C{^1^H} NMR (126 MHz, DMSO-*d*_6_) *δ* 163.60, 159.37, 148.95, 146.40, 144.18, 142.46, 141.51, 134.44, 130.73, 129.45, 129.12, 128.84, 128.19, 126.34, 122.84, 117.49, 115.17, 21.44. LC/MS *m/z*: 290.1 [M + H]^+^.

###### 4.1.1.3.18. (E)-4-Methyl-N′-(quinoxalin-2-ylmethylene)benzohydrazide (BE5001).

Gray solid, mp: 212–224 °C, yield: 78 mg (62%). ^1^H NMR (500 MHz, DMSO-*d*_6_) *δ* 12.27 (s, 1H), 9.46 (s, 1H), 8.65 (s, 1H), 8.16–8.07 (m, 2H), 7.91–7.88 (m, 2H), 7.88–7.85 (m, 2H), 7.38 (d, *J* = 7.9 Hz, 2H), 2.40 (s, 3H). ^13^C{^1^H} NMR (126 MHz, DMSO-*d*_6_) *δ* 163.85, 148.99, 146.05, 143.31, 142.87, 142.04, 141.74, 131.28, 131.14, 130.50, 129.54, 129.48, 128.32, 21.54. LC/MS *m/z*: 291.1 [M + H]^+^.

###### 4.1.1.3.19. (E)-4-Methyl-N′-(naphthalen-1-ylmethylene)benzohydrazide (BE5086) [38].

White solid, mp: 186–188 °C, yield: 93 mg (71%). ^1^H NMR (500 MHz, DMSO-*d*_6_) *δ* 11.89 (s, 1H), 9.15 (s, 1H), 8.88 (d, *J* =8.6 Hz, 1H), 8.01 (dd, *J* = 8.2, 4.1 Hz, 2H), 7.93 (dd, *J* =16.9, 7.5 Hz, 3H), 7.67 (t, *J* =7.7 Hz, 1H), 7.60 (td, *J* = 7.6, 3.4 Hz, 2H), 7.36 (d, *J* = 7.8 Hz, 2H), 2.38 (s, 3H). LC/MS *m/z*: 290.1 [M + H]^+^.

###### 4.1.1.3.20. (E)-N′-(Isoquinolin-4-ylmethylene)-4-methylbenzohydrazide (BE5112).

White solid, mp: 178–179.8 °C, yield: 156 mg (88%). ^1^H NMR (500 MHz, DMSO-*d*_6_) *δ* 12.16 (s, 1H), 9.53 (s, 1H), 9.10 (d, *J* = 8.6 Hz, 1H), 8.98 (s, 1H), 8.83 (s, 1H), 8.36 (d, *J* = 8.2 Hz, 1H), 8.09 (t, *J* = 7.8 Hz, 1H), 7.89 (dt, *J* = 7.6, 3.5 Hz, 3H), 7.35 (d, *J* = 7.7 Hz, 2H), 2.38 (s, 3H). ^13^C{^1^H} NMR (126 MHz, DMSO-*d*_6_) *δ* 163.57, 159.33, 151.74, 144.89, 142.61, 140.04, 134.74, 133.48, 130.54, 129.51, 129.46, 128.26, 128.21, 126.13, 125.02, 117.48, 115.16, 21.47. LC/MS *m/z*: 290.1 [M + H]^+^.

###### 4.1.1.3.21. (E)-4-Methyl-N′-(quinolin-4ylmethylene)benzohydrazide (BE2113).

Yellow solid, mp: 218–225 °C, yield: 131 mg (82%). ^1^H NMR (500 MHz, DMSO-*d*_6_) *δ* 12.30 (s, 1H), 9.16 (s, 1H), 9.10 (d, *J* = 4.9 Hz, 1H), 8.80 (s, 1H), 8.18 (d, *J* = 8.5 Hz, 1H), 8.05–7.93 (m, 2H), 7.93–7.80 (m, 3H), 7.38 (d, *J* =7.9 Hz, 2H), 2.41 (s, 3H); ^13^C{^1^H} NMR (126 MHz, DMSO-*d*_6_) *δ* 167.57, 158.97, 158.69, 148.93, 145.61, 144.02, 141.11, 131.70, 129.62, 128.95, 128.32, 127.60, 125.39, 125.10, 120.14, 117.34, 115.01, 21.55. LC/MS *m/z*: 290.1 [M + H]^+^.

###### 4.1.1.3.22. (E)-4-Methyl-N′-(quinolin-5-ylmethylene)benzohydrazide (BE2347)

White solid, mp: 211–214 °C, yield: 110 mg (86%). ^1^H NMR (500 MHz, DMSO-*d*_6_) *δ* 12.09 (s, 1H), 9.69 (s, 1H), 9.15 (d, *J* = 6.5 Hz, 1H), 9.00 (s, 1H), 8.75 (d, *J* = 6.6 Hz, 1H), 8.34 (dd, *J* = 40.9, 7.8 Hz, 2H), 7.91 (dd, *J* = 23.2, 7.8 Hz, 3H), 7.42–7.27 (m, 2H), 2.37 (d, *J* = 2.0 Hz, 3H). ^13^C{^1^H} NMR (100 MHz, DMSO-*d*_6_) *δ* 163.54, 159.13, 150.95, 146.58, 142.60, 138.79, 135.46, 134.37, 131.68, 130.59, 129.84, 129.55, 129.33, 128.73, 128.16, 120.61, 115.07, 21.50. LC/MS *m/z*: 290.1 [M + H]^+^.

###### 4.1.1.3.23. (E)-N′-(Isoquinolin-5-ylmethylene)-4-methylbenzohydrazide (BE5118).

Yellow solid, mp: 216–218 °C, yield: 96 mg (61%). ^1^H NMR (500 MHz, DMSO-*d*_6_) 12.02 (s, 1H), 9.06 (d, *J* = 4.5 Hz, 1H), 8.78–8.62 (m, 2H), 8.33 (d, *J* = 7.7 Hz, 2H), 8.15 (d, *J* = 8.9 Hz, 1H), 7.87 (d, *J* = 7.7 Hz, 2H), 7.77 (dd, *J* = 8.3, 4.2 Hz, 1H), 7.34 (d, *J* = 7.5 Hz, 2H), 2.38 (d, *J* = 2.9 Hz, 3H). ^13^C{^1^H} NMR (126 MHz, DMSO-*d*_6_) *δ* 163.60, 159.09, 148.95, 146.40, 144.18, 142.46, 141.51, 134.44, 130.73, 129.45, 129.12, 128.84, 126.34, 122.84, 119.81, 117.49, 115.17, 21.44. LC/MS *m/z*: 290.1 [M + H]^+^.

###### 4.1.1.3.24. (E)-N′-(Naphthalen-2-ylmethylene)benzohydrazide (BE5066) [39].

White solid, mp: 217–219 °C, yield, 95 mg (83%). ^1^H NMR (500 MHz, DMSO-*d*_6_) *δ* 11.96 (s, 1H), 8.64 (s, 1H), 8.16 (s, 1H), 8.04–7.93 (m, 6H), 7.63–7.53 (m, 5H). LC/MS *m/z*: 275.1 [M + H]^+^.

###### 4.1.1.3.25. (E)-N′-(Quinolin-2-ylmethylene)benzohydrazide (BE5062) [18].

Faint yellow solid, mp: 170.4–172 °C, yield: 101 mg (68%). ^1^H NMR (500 MHz, DMSO-*d*_6_) *δ* 12.26 (s, 1H), 8.64 (s, 1H), 8.48 (d, *J* = 8.6 Hz, 1H), 8.16 (d, *J* = 8.7 Hz, 1H), 8.05 (dd, *J* = 17.6, 8.3 Hz, 2H), 7.96 (d, *J* = 7.6 Hz, 2H), 7.82 (ddd, *J* = 8.4, 6.8, 1.5 Hz, 1H), 7.69–7.61 (m, 2H), 7.57 (dd, *J* = 8.4, 6.9 Hz, 2H). LC/MS *m/z*: 276.1 [M + H]^+^.

###### 4.1.1.3.26. (E)-N′-(Isoquinolin-3-ylmethylene)benzohydrazide (BE21467).

White solid, mp: 121–124.6 °C, yield 86 mg (74%). ^1^H NMR (500 MHz, DMSO-*d*_6_) *δ* 12. 10 (d, *J* = 2.4 Hz, 1H), 9.37 (d, *J* = 2.3 Hz, 1H), 8.71–8.65 (m, 1H), 8.44 (d, *J* =2.4 Hz, 1H), 8.14 (td, *J* = 10.1, 9.1, 4.6 Hz, 2H), 8.01–7.92 (m, 2H), 7.84–7.78 (m, 1H), 7.75–7.67 (m, 1H), 7.64–7.59 (m, 1H), 7.56 (tt, *J* = 7.1, 1.8 Hz, 2H). ^13^C NMR (126 MHz, DMSO-*d*_6_) *δ* 168.11, 167.81, 163.82, 152.95, 148.78, 147.38, 135.84, 133.80, 132.39, 131.58, 129.02, 128.85, 128.79, 128.21, 128.13, 127.89, 117.46. LC/MS *m/z*: 276.1 [M + H]^+^.

###### 4.1.1.3.27. (E)-N′-(Quinolin-3-ylmethylene)benzohydrazide (BE5108).

White solid, mp: 246.2–248 °C, Yield: 104 mg (76%). ^1^H NMR (500 MHz, DMSO-*d*_6_) *δ* 12.09 (s, 1H), 9.32 (d, *J* = 2.2 Hz, 1H), 8.68 (s, 1H), 8.62 (d, *J* = 2.2 Hz, 1H), 8.10–8.04 (m, 2H), 7.96 (d, *J* = 7.5 Hz, 2H), 7.81 (ddd, *J* = 8.5, 6.8, 1.5 Hz, 1H), 7.69–7.64 (m, 1H), 7.62 (dd, *J* = 8.5, 6.5 Hz, 1H), 7.58–7.53 (m, 2H). ^13^C{^1^H} NMR (126 MHz, DMSO-*d*_6_) *δ* 219.70, 174.18, 169.09, 148.79, 145.83, 135.31, 133.72, 132.39, 130.92, 129.31, 129.13, 129.00, 128.16, 127.84. LC/MS *m/z*: 276.1 [M + H]^+^.

###### 4.1.1.3.28. (E)-N′-(Quinolin-6-ylmethylene)benzohydrazide (BE5114).

Faint yellow solid, mp: 221–225.1 °C, Yield: 85 mg (56%). ^1^H NMR (500 MHz, DMSO-*d*_6_) *δ* 12.08 (s, 1H), 9.06 (d, *J* = 4.5 Hz, 1H), 8.74–8.64 (m, 2H), 8.34 (d, *J* = 10.4 Hz, 2H), 8.16 (d, *J* = 8.7 Hz, 1H), 7.96 (d, *J* = 7.6 Hz, 2H), 7.77 (dd, *J* = 8.4, 4.5 Hz, 1H), 7.61 (d, *J* = 7.4 Hz, 1H), 7.55 (t, *J* = 7.6 Hz, 2H). ^13^C{^1^H} NMR (126 MHz, DMSO-*d*_6_) *δ* 163.80, 159.22, 158.93, 149.37, 146.77, 144.76, 144.75, 141.06, 134.23, 133.69, 132.38, 128.97, 128.76, 128.18, 126.82, 122.87, 117.41. LC/MS *m/z*: 276.1 [M + H]^+^.

###### 4.1.1.3.29. (E)-N′-Quinoxalin-2-ylmethylene)benzohydrazide (BE5064) [40].

Gray solid, mp: 171–173.4 °C, yield: 88 mg (62%). ^1^H NMR (500 MHz, DMSO-*d*_6_) *δ* 12.35 (s, 1H), 9.47 (s, 1H), 8.65 (s, 1H), 8.12 (td, *J* = 7.7, 3.5 Hz, 2H), 7.96 (d, *J* = 7.6 Hz, 2H), 7.89 (qd, *J* = 6.8, 3.5 Hz, 2H), 7.65 (t, *J* = 7.3 Hz, 1H), 7.58 (t, *J* = 7.5 Hz, 2H). LC/MS *m/z*: 277.1 [M + H]^+^.

###### 4.1.1.3.30. (E)-N′-(Naphthalen-1-ylmethylene)benzohydrazide (BE5067) [41].

White solid, mp: 216.3–217.5 °C, yield: 98 mg (75%). ^1^H NMR (500 MHz, DMSO-*d*_6_) *δ* 11.97 (s, 1H), 8.64 (s, 1H), 8.15 (s, 1H), 8.01 (d, *J* = 8.7 Hz, 2H), 7.98–7.91 (m, 4H), 7.59 (d, *J* = 7.3 Hz, 1H), 7.57–7.48 (m, 4H). LC/MS *m/z*: 275.1 [M + H]^+^.

###### 4.1.1.3.31. (E)-N′-(Isoquinolin-4-ylmethylene)benzohydrazide (BE5111).

White solid, mp: 198.2–199 °C, yield: 72 mg (53%). ^1^H NMR (500 MHz, DMSO-*d*_6_) *δ* 12.20 (s, 1H), 9.52 (s, 1H), 9.15 (d, *J* = 8.6 Hz, 1H), 8.99 (s, 1H), 8.83 (s, 1H), 8.35 (d, *J* = 8.2 Hz, 1H), 8.08 (t, *J* = 7.7 Hz, 1H), 7.99 (d, *J* = 7.6 Hz, 2H), 7.88 (t, *J* = 7.5 Hz, 1H), 7.63 (t, *J* = 7.4 Hz, 1H), 7.57 (t, *J* = 7.5 Hz, 2H). ^13^C{^1^H} NMR (126 MHz, DMSO-*d*_6_) *δ* 163.77, 159.10, 158.81, 152.49, 145.61, 141.36, 134.43, 133.53, 133.28, 132.51, 130.11, 129.33, 128.36, 128.19, 125.67, 125.05, 117.35. LC/MS *m/z*: 276.1 [M + H]^+^.

###### 4.1.1.3.32. (E)-N′-(Quinolin-4-ylmethylene)benzohydrazide (BE5063) [18].

Gray solid, mp: 170–174.3 °C, yield: 51 mg (34%). ^1^H NMR (500 MHz, MSO-*d_6_*) *δ* 12.39 (s, 1H), 9.17 (s, 1H), 9.11 (d, *J* = 4.9 Hz, 1H), 8.81 (s, 1H), 8.18 (d, *J* = 8.4 Hz, 1H), 8.06–7.97 (m, 3H), 7.95 (d, *J* = 8.0 Hz, 1H), 7.86 (s, 1H), 7.65 (t, *J* = 7.3 Hz, 1H), 7.58 (t, *J* = 7.5 Hz, 2H). LC/MS *m/z*: 276.1 [M + H]^+^.

###### 4.1.1.3.33. (E)-N′-(Quinolin-5-ylmethylene)benzohydrazide (BE5065).

White solid, mp: 189–197.1 °C, yield: 81 mg (56%). ^1^H NMR (500 MHz, DMSO-*d*_6_) *δ* 12.16 (s, 1H), 9.70 (s, 1H), 9.15 (d, *J* = 6.4 Hz, 1H), 9.03 (s, 1H), 8.77 (d, *J* = 6.4 Hz, 1H), 8.40 (d, *J* = 8.2 Hz, 1H), 8.30 (d, *J* = 7.3 Hz, 1H), 8.02–7.91 (m, 3H), 7.65–7.60 (m, 1H), 7.57 (dd, *J* = 8.2, 6.7 Hz, 2H). ^13^C{^1^H} NMR (126 MHz, DMSO-*d*_6_) *δ* 163.74, 159.30, 159.02, 150.84, 146.86, 138.60, 135.57, 134.45, 133.56, 132.45, 131.76, 129.84, 129.35, 128.73, 128.16, 120.69, 117.72, 115.39. LC/MS *m/z*: 276.1 [M + H]^+^.

###### 4.1.1.3.34. (E)-N′-(Isoquinolin-5-ylmethylene)benzohydrazide (BE5119).

White solid, mp: 189.2–197.1 °C, yield: 126 mg (88%). ^1^H NMR (500 MHz, DMSO-*d*_6_) *δ* 12.16 (s, 1H), 9.69 (s, 1H), 9.15 (d, *J* = 6.4 Hz, 1H), 9.03 (s, 1H), 8.77 (d, *J* = 6.6 Hz, 1H), 8.40 (d, *J* = 8.2 Hz, 1H), 8.30 (d, *J* = 7.3 Hz, 1H), 7.99 (d, *J* = 7.6 Hz, 2H), 7.94 (t, *J* = 7.8 Hz, 1H), 7.65–7.60 (m, 1H), 7.59–7.54 (m, 2H). ^13^C{^1^H} NMR (126 MHz, DMSO-*d*_6_) *δ* 161.73, 159.08, 158.80, 151.22, 146.97, 139.35, 135.34, 134.30, 133.59, 132.47, 131.66, 129.79, 129.20, 129.05, 128.79, 128.16, 120.41, 117.63, 115.30. LC/MS *m/z*: 276.1 [M + H]^+^.

###### 4.1.1.3.35. (E)-N′-(3,4-dichlorobenzylidene)-4-hydroxybenzohydrazide (BE5136) [42].

Yellow solid, mp: 249.6–251.1 °C, yield: 62 mg (45%). ^1^H NMR (500 MHz, DMSO-*d*_6_) *δ* 11.84 (s, 1H), 10.16 (s, 1H), 8.39 (s, 1H), 7.97–7.76 (m, 3H), 7.66 (q, *J* = 7.8 Hz, 2H), 6.88 (d, *J* = 8.0 Hz, 2H). LC/MS *m/z*: 301.1 [M + H]^+^.

###### 4.1.1.3.36. (E)-N′-(3,4-dichlorobenzylidene)-4-methylbenzohydrazide (BE5137) [43].

White solid, mp: 221.1–222.3 °C, yield: 93 mg (81%). ^1^H NMR (500 MHz, DMSO-*d*_6_) *δ* 11.97 (s, 1H), 8.42 (s, 1H), 7.96–7.77 (m, 3H), 7.68 (t, *J* = 7.8 Hz, 2H), 7.31 (d, *J* = 7.8 Hz, 2H), 2.36 (s, 3H). LC/MS *m/z*: 308.1 [M + H]^+^.

###### 4.1.1.3.37. (E)-N′-(3,4-Dichlorobenzylidene)benzohydrazide (BE5138) [43].

White solid, mp: 184.2–185.4 °C, yield: 89 mg (76%). ^1^H NMR (500 MHz, DMSO-*d*_6_) *δ* 12.05 (s, 1H), 8.43 (s, 1H), 7.98–7.88 (m, 3H), 7.73 (s, 2H), 7.61 (t, *J* = 7.4 Hz, 1H), 7.53 (t, *J* = 7.6 Hz, 2H). LC/MS *m/z*: 294.1 [M + H]^+^.

###### 4.1.1.3.38. (E)-N′-Benzylidene-4-hydroxybenzenesulfonohydrazide (BE21468).

Transparent crystal solid, mp: 214.6–218.1 °C, yield: 65 mg (79%). ^1^H NMR (500 MHz, DMSO-*d*_6_) *δ* 11.94 (s, 1H), 9.61 (s, 1H), 9.12–8.94 (m, 2H), 8.70 (d, *J* = 6.3 Hz, 1H), 8.32 (d, *J* = 8.2 Hz, 1H), 8.22 (d, *J* = 7.3 Hz, 1H), 7.88 (d, *J* = 8.6 Hz, 2H), 7.85 (d, *J* = 7.8 Hz, 1H), 6.91 (d, *J* = 8.5 Hz, 2H). ^13^C{^1^H} NMR (126 MHz, DMSO-*d*_6_) *δ* 219.82, 174.20, 172.54, 163.92, 161.11, 147.97, 134.56, 130.61, 130.16, 129.30, 127.59, 123.88, 115.63. LC/MS *m/z*: 277.1 [M + H]^+^.

###### 4.1.1.3.39. (E)-4-Hydroxy-N′-(naphthalen-2-ylmethylene)benzene-sulfonohydrazide (BE21469).

White solid, mp: 194–201 °C, yield: 45 mg (71%). ^1^H NMR (500 MHz, DMSO-*d*_6_) *δ* 11.64 (d, *J* = 2.5 Hz, 1H), 8.09 (d, *J* = 2.4 Hz, 1H), 8.01 (d, *J* = 2.5 Hz, 1H), 7.96–7.88 (m, 5H), 7.76 (dq, *J* = 8.6, 1.6 Hz, 1H), 7.69–7.59 (m, 3H), 7.57–7.51 (m, 2H). ^13^C {^1^H} NMR (126 MHz, DMSO-*d*_6_) *δ* 190.62, 147.67, 139.51, 134.13, 133.55, 133.17, 131.78, 129.74, 129.18, 128.99, 128.78, 128.21, 127.66, 122.59. LC/MS *m/z*: 327.1 [M + H]^+^.

###### 4.1.1.3.40. (E)-4-Hydroxy-N′-(naphthalen-1-ylmethylene)benzene-sulfonohydrazide (BE21470).

White solid, mp: 202–208.4 °C, yield: 104 mg (84%). ^1^H NMR (500 MHz, DMSO-*d*_6_) *δ* 11.77 (s, 1H), 10.20–10.14 (m, 1H), 8.61 (s, 1H), 8.12 (s, 1H), 7.96 (dd, *J* = 22.3, 9.5, 6.5, 3.3 Hz, 4H), 7.87 (dd, *J* = 8.7, 2.8 Hz, 2H), 7.55 (dp, *J* = 6.7, 3.6 Hz, 2H), 6.91 (dd, *J* = 8.7, 2.8 Hz, 2H). ^13^C{^1^H} NMR (126 MHz, DMSO-*d*_6_) *δ* 194.87, 148.05, 139.46, 137.26, 135.73, 133.78, 131.32, 131.10, 130.23, 130.19, 129.76, 128.67, 127.82, 126.74, 125.88, 124.62, 124.58. LC/MS *m/z*: 327.1 [M + H]^+^.

###### 4.1.1.3.41. (E)-4-Hydroxy-N′-(isoquinolin-4-ylmethylene)benzene-sulfonohydrazide (BE21471).

White solid, mp: 124.2–126 °C, yield: 93 mg (86%). ^1^H NMR (500 MHz, DMSO-*d*_6_) *δ* 11.99 (s, 1H), 9.39 (s, 1H), 8.76 (s, 1H), 8.62 (s, 1H), 8.17–8.05 (m, 2H), 7.91–7.80 (m, 3H), 7.73 (t, *J* = 7.6 Hz, 1H), 6.89 (d, *J* = 8.2 Hz, 2H). ^13^C{^1^H} NMR (126 MHz, DMSO-*d*_6_) *δ* 161.49, 154.21, 147.24, 137.66, 130.79, 130.47, 128.84, 128.54, 128.32, 127.84, 123.96, 118.01, 115.60. LC/MS *m/z*: 328.1 [M + H]^+^.

###### 4.1.1.3.42. (E)-N′-(3,4-Dichlorobenzylidene)-4-hydroxybenzenesulfonohydrazide (BE21472).

White solid, mp: 214–219 °C, yield: 114 mg (88%). ^1^H NMR (500 MHz, DMSO-*d*_6_) *δ* 11.81 (s, 1H), 7.90 (d, *J* = 8.8 Hz, 3H), 7.75 (dt, *J* = 5.0, 1.8 Hz, 1H), 7.66 (ddd, *J* = 9.1, 5.8, 2.7 Hz, 1H), 7.61 (ddd, *J* = 8.9, 6.7, 2.1 Hz, 3H), 7.56–7.51 (m, 1H). ^13^C{^1^H} NMR (126 MHz, DMSO-*d*_6_) *δ δ* 144.93, 139.37, 134.85, 133.65, 132.81, 132.11, 131.52, 129.79, 128.93, 127.61, 126.82. LC/MS *m/z*: 346.1 [M + H]^+^.

###### 4.1.1.3.43. (E)-N′-Benzylidene-4-methylbenzenesulfonohydrazide (BE21446) [44].

White solid, mp: 221–226.3 °C, yield: 108 mg (78%). ^1^H NMR (500 MHz, DMSO-*d*_6_) *δ* 11.78 (s, 1H), 8.47 (s, 1H), 7.84 (d, *J* = 7.8 Hz, 2H), 7.73 (d, *J* = 7.2 Hz, 2H), 7.46 (d, *J* = 8.4 Hz, 3H), 7.34 (d, *J* = 7.7 Hz, 2H), 2.38 (s, 3H). LC/MS *m/z*: 275.1 [M + H]^+^.

###### 4.1.1.3.44. (E)-4-Methyl-N′-(naphthalen-2-ylmethylene)benzenesulfonohydrazide (BE21447) [45].

White solid, mp: 145–151 °C, yield: 65 mg (79%). ^1^H NMR (500 MHz, DMSO-*d*_6_) *δ* 11.55 (d, *J* = 1.1 Hz, 1H), 8.07 (s, 1H), 8.01 (s, 1H), 7.96–7.92 (m, 1H), 7.90 (d, *J* = 8.6 Hz, 2H), 7.81 (dt, *J* = 8.6, 1.6 Hz, 2H), 7.76 (dq, *J* = 8.7, 1.6 Hz, 1H), 7.57–7.51 (m, 2H), 7.41 (d, *J* = 8.0 Hz, 2H), 2.34 (s, 3H). LC/MS *m/z*: 325.1 [M + H]^+^.

###### 4.1.1.3.45. (E)-N′-((5,6-Dihydronaphthalen-1-yl)methylene)-4-methylbenzenesulfonohydrazide (BE21448) [46].

White solid, mp: 112–118.6 °C, yield: 87 mg (67%). ^1^H NMR (500 MHz, DMSO-*d*_6_) *δ* 11.87 (s, 1H), 8.62 (s, 1H), 8.15 (s, 1H), 8.03–7.92 (m, 4H), 7.87 (d, *J* = 7.8 Hz, 2H), 7.57 (dd, *J* = 6.2, 3.2 Hz, 2H), 7.35 (d, *J* = 7.9 Hz, 2H), 2.39 (s, 3H). LC/MS *m/z*: 325.1 [M + H]^+^.

###### 4.1.1.3.46. (E)-N′-((7,8-Dihydroisoquinolin-4-yl)methylene)-4-methylbenzenesulfonohydrazide (BE21449).

White solid, mp: 104.3–105.8 °C, yield: 96 mg (84%). ^1^H NMR (500 MHz, DMSO-*d*_6_) *δ* 12.16 (s, 1H), 9.53 (s, 1H), 9.10 (d, *J* = 8.6 Hz, 1H), 8.98 (s, 1H), 8.83 (s, 1H), 8.36 (d, *J* = 8.2 Hz, 1H), 8.09 (t, *J* = 7.8 Hz, 1H), 7.89 (dt, *J* = 7.6, 3.5 Hz, 3H), 7.35 (d, *J* = 7.7 Hz, 2H), 2.38 (s, 3H). ^13^C{^1^H} NMR (126 MHz, DMSO-*d*_6_) *δ* 167.58, 163.78, 154.36, 148.16, 147.86, 142.64, 137.20, 130.77, 130.54, 129.56, 128.49, 128.37, 128.29, 128.29, 127.76, 117.97, 21.54. LC/MS *m/z*: 326.1 [M + H]^+^.

###### 4.1.1.3.47. (E)-N′-(3,4-Dichlorobenzylidene)-4-methylbenzenesulfonohydrazide (BE21450) [47].

White solid, mp: 143–149.2 °C, yield: 52 mg (73%). ^1^H NMR (500 MHz, DMSO-*d*_6_) *δ* 11.97 (s, 1H), 8.42 (s, 1H), 7.96–7.77 (m, 3H), 7.68 (t, *J* = 7.8 Hz, 2H), 7.31 (d, *J* = 7.8 Hz, 2H), 2.36 (s, 3H). LC/MS *m/z*: 344.1 [M + H]^+^.

###### 4.1.1.3.48. (E)-4-Hydroxy-N′-(1-phenylethylidene)benzohydrazide (BE5035) [48].

White solid, mp: 244.4–246.1 °C, yield: 138.32 mg (91%). ^1^H NMR (500 MHz, DMSO-*d*6) δ10.54 (s, 1H), 10.10 (s, 1H), 7.81 (dd, *J* = 17.2, 6.8 Hz, 4H), 7.43 (h, *J* = 3.3, 2.5 Hz, 3H), 6.90–6.78 (m, 2H), 2.35 (s, 3H). ^13^C{^1^H} NMR (126 MHz, DMSO-*d*_6_) *δ* 206.72, 160.93, 138.75, 135.87, 129.69, 128.80, 126.77, 125.58, 124.97, 124.31, 115.27, 31.47. LC/MS *m/z*: 255.1 [M + H]^+^.

###### 4.1.1.3.49. (E)-N′-(1-(4-(Tert-butyl)phenyl)ethylidene)-4-hydroxybenzohydrazide (BE5039).

White solid, mp: 241.3–242.5 °C, yield: 106.4 mg (70%). ^1^H NMR (500 MHz, DMSO-*d*_6_) *δ* 10.47 (d, *J* = 3.3 Hz, 1H), 10.04 (s, 1H), 7.81–7.69 (m, 4H), 7.46–7.40 (m, 2H), 6.85 (dd, *J* = 9.3, 3.0 Hz, 2H), 2.32 (d, *J* = 3.2 Hz, 3H), 1.29 (d, *J* = 3.3 Hz, 9H). ^13^C {^1^H} NMR (126 MHz, DMSO-*d*_6_) δ 198.41, 167.56, 160.88, 152.36, 135.98, 126.57, 125.55, 125.02, 115.25, 34.87, 31.47. LC/MS *m/z*: 311.1 [M + H]^+^.

###### 4.1.1.3.50. (E)-4-Hydroxy-N′-(1-(naphthalen-2-yl)ethylidene)benzohydrazide (BE5040) [49].

White solid powder, mp: 250 °C, yield: 135.28 mg (89%). ^1^H NMR (500 MHz, DMSO-*d*_6_) δ 10.63–10.53 (m, 1H), 10.08 (d, *J* = 1.5 Hz, 1H), 8.30 (s, 1H), 8.11 (s, 1H), 8.03–7.98 (m, 1H), 7.93 (d, *J* = 9.0 Hz, 2H), 7.85–7.80 (m, 2H), 7.58–7.53 (m, 2H), 6.89–6.86 (m, 2H), 2.48 (s, 3H). ^13^C{^1^H} NMR (126 MHz, DMSO-*d*_6_) *δ* 161.01, 136.14, 133.72, 133.24, 129.00, 128.13, 127.95, 127.28, 126.92, 126.80, 124.97, 124.10, 115.32, 17.49. LC/MS *m/z*: 305.1 [M + H]^+^.

###### 4.1.1.3.51. (E)-N′-(1-(2,4-Dichlorophenyl)ethylidene)-4-hydroxybenzohydrazide (BE5041).

White solid, mp: 248.3–250.3 °C, yield: 159.12 mg (78%). ^1^H NMR (500 MHz, DMSO-*d*_6_) *δ* 10.03 (s, 1H), 10.01–9.99 (m, 1H), 7.74 (t, *J* = 1.6 Hz, 1H), 7.53 (ddd, *J* = 8.3, 2.2, 1.0 Hz, 1H), 7.51–7.45 (m, 2H), 7.43 (dt, *J* = 8.2, 1.1 Hz, 1H), 6.78–6.73 (m, 2H), 2.23 (s, 3H). ^13^C{^1^H} NMR (126 MHz, DMSO-*d*_6_) *δ* 169.30, 160.87, 134.93, 133.98, 132.01, 131.23, 129.92, 128.52, 124.75, 115.16, 24.80. LC/MS *m/z*: 324.1 [M + H]^+^.

###### 4.1.1.3.52. (E)-N′-(1-(3,4-Dichlorophenyl)ethylidene)-4-hydroxybenzohydrazide (BE5042).

White solid, mp: 242.4–244 °C, yield: 122 mg (62%). ^1^H NMR (500 MHz, DMSO-*d*_6_) *δ* 10.62 (s, 1H), 10.10 (s, 1H), 8.01 (s, 1H), 7.78 (d, *J* = 8.3 Hz, 3H), 7.71–7.65 (m, 1H), 6.90–6.83 (m, 2H), 2.35 (d, *J* = 2.6 Hz, 3H). ^13^C{^1^H} NMR (126 MHz, DMSO-*d*_6_) *δ* 161.09, 139.36, 132.14, 131.75, 131.01, 128.35, 126.84, 124.70, 115.30, 17.49. LC/MS m/z: 324.1 [M + H]^+^.

###### 4.1.1.3.53. (E)-N′-(1-(2,5-Dichlorophenyl)ethylidene)-4-hydroxybenzohydrazide (BE5043).

White solid, mp: 223.1–224 °C, yield: 110 mg (67%). ^1^H NMR (500 MHz, DMSO-*d*_6_) *δ* 10.58 (s, 1H), 10.09 (s, 1H), 7.80–7.75 (m, 2H), 7.56 (d, *J* = 8.5 Hz, 1H), 7.51 (dd, *J* = 8.5, 2.6 Hz, 1H), 7.49 (d, *J* = 2.5 Hz, 1H), 6.85 (dd, *J* = 8.4, 6.0 Hz, 2H), 2.34 (s, 3H). ^13^C{^1^H} NMR (126 MHz, DMSO-*d*_6_) *δ* 167.09, 141.30, 132.21, 131.90, 130.38, 130.33, 130.31, 124.57, 115.29, 18.55. LC/MS *m/z*: 324.1 [M + H]^+^.

###### 4.1.1.3.54. (E)-4-Hydroxy-N′-(1-(2-methoxyphenyl)ethylidene)benzohydrazide (BE5044).

White solid, mp: 218–219.7 °C, yield: 96 mg (78%). ^1^H NMR (500 MHz, DMSO-*d*_6_) *δ* 10.05 (s, 1H), 9.29 (s, 1H), 7.50–7.35 (m, 3H), 7.26 (dt, *J* = 7.5, 1.4 Hz, 1H), 7.19 (dd, *J* = 8.4, 1.0 Hz, 1H), 7.08 (ddt, *J* = 8.6, 7.4, 1.1 Hz, 1H), 6.79–6.73 (m, 2H), 3.82 (d, *J* = 1.2 Hz, 3H), 2.23 (s, 3H). ^13^C{^1^H} NMR (126 MHz, DMSO-*d*_6_) 194.68, 160.96, 155.67, 131.53, 131.01, 128.58, 124.49, 123.48, 121.58, 115.54, 56.15, 24.89. LC/MS *m/z*: 285.1 [M + H]^+^.

###### 4.1.1.3.55. (E)-4-Hydroxy-N′-(1-(3-methoxyphenyl)ethylidene)benzohydrazide (BE5045).

White solid, mp: 219–221.4 °C, yield: 103 mg (81%). ^1^H NMR (500 MHz, DMSO-*d*_6_) *δ* 10.53 (d, *J* = 2.4 Hz, 1H), 10.09–10.05 (m, 1H), 7.79 (dt, *J* = 9.1, 2.3 Hz, 2H), 7.34 (dd, *J* = 15.9, 7.9 Hz, 3H), 6.99 (dd, *J* = 8.0, 2.6 Hz, 1H), 6.88–6.85 (m, 2H), 3.79 (s, 3H), 2.34 (d, *J* = 1.5 Hz, 3H). ^13^C{^1^H} NMR (126 MHz, DMSO-*d*_6_) *δ* 160.94, 159.68, 159.66, 140.22, 129.86, 124.95, 119.32, 115.40, 55.56, 17.48. LC/MS *m/z*: 285.1 [M + H]^+^.

###### 4.1.1.3.56. (E)-N′-(1-(2-Fluoro-4-methoxyphenyl)ethylidene)-4-hydroxybenzohydrazide (BE5046).

White solid, mp: 203–206.2 °C, yield: 98 mg (79%). ^1^H NMR (500 MHz, DMSO-*d*_6_) *δ* 10.47 (s, 1H), 10.06 (s, 1H), 7.80–7.74 (m, 2H), 7.56 (t, *J* = 8.9 Hz, 1H), 6.92–6.80 (m, 4H), 3.81 (d, *J* = 1.8 Hz, 3H), 2.32 (d, *J* = 2.5 Hz, 3H). ^13^C{^1^H} NMR (126 MHz, DMSO-*d*_6_) *δ* 162.32, 161.78, 161.69, 160.97, 160.35, 130.88, 124.86, 120.04, 119.95, 115.29, 111.08, 111.06, 102.37, 102.16, 56.24, 18.01. LC/MS *m/z*: 303.1 [M + H]^+^.

###### 4.1.1.3.57. (E)-N′-(1-(3-Fluoro-4-methoxyphenyl)ethylidene)-4-hydroxybenzohydrazide (BE5047).

White solid, mp: 232–234.2 °C, yield: 78 mg (71%). ^1^H NMR (500 MHz, DMSO-*d*_6_) *δ* 10.49 (s, 1H), 10.06 (s, 1H), 7.77 (d, *J* = 8.2 Hz, 2H), 7.71–7.52 (m, 2H), 7.20 (t, *J* = 8.8 Hz, 1H), 6.88–6.81 (m, 2H), 3.88 (d, *J* = 1.1 Hz, 3H), 2.31 (d, *J* = 1.1 Hz, 3H). ^13^C{^1^H} NMR (126 MHz, DMSO-*d*_6_) *δ* 167.74, 152.67, 150.74, 131.74, 131.69, 124.95, 123.50, 115.27, 113.79, 56.54, 39.49. LC/MS *m/z*: 303.1 [M + H]^+^.

###### 4.1.1.3.58. (E)-4-Methyl-N′-(1-phenylethylidene)benzohydrazide (BE5027) [50].

White solid crystal, mp: 194.1–196.7 °C, yield: 338.82 mg (94%). ^1^H NMR (500 MHz, DMSO-*d*_6_) *δ* 10.69 (s, 1H), 7.99–7.65 (m, 4H), 7.55–7.18 (m, 5H), 2.44–2.28 (m, 6H). ^13^C{^1^H} NMR (126 MHz, DMSO-*d*_6_) *δ* 219.71, 169.08, 138.61, 129.85, 129.27, 128.81, 128.37, 128.35, 126.83, 21.49, 15.01. LC/MS *m/z*: 253.1 [M + H]^+^.

###### 4.1.1.3.59. (E)-N′-(1-(2,4-Dichlorophenyl)ethylidene)-4-methylbenzohydrazide (BE5031).

White solid powder, mp: 169–172.1 °C, yield: 121.5 mg (81%). ^1^H NMR (500 MHz, DMSO-*d*_6_) *δ* 10.73 (s, 1H), 7.88–7.64 (m, 3H), 7.48 (dd, *J* = 14.8, 5.9 Hz, 2H), 7.31 (d, *J* = 8.4 Hz, 2H), 2.37 (d, *J* = 5.8 Hz, 3H), 2.34 (d, *J* = 5.2 Hz, 3H).^13^C{^1^H} NMR (126 MHz, DMSO-*d*_6_) *δ* 207.54, 167.14, 145.92, 138.58, 134.37, 132.65, 132.04, 129.65, 129.22, 127.97, 115.54, 109.97, 21.50, 18.81. LC/MS *m/z*: 322.1 [M + H]^+^.

###### 4.1.1.3.60. (E)-N′-(1-(3,4-Dichlorophenyl)ethylidene)-4-methylbenzohydrazide (BE5032).

White solid powder, mp: 188–191 °C, yield: 111.13 mg (74%). ^1^H NMR (500 MHz, DMSO-*d*_6_) *δ* 10.79 (s, 1H), 8.03 (s, 1H), 7.80 (s, 3H), 7.69 (d, *J* = 8.4 Hz, 1H), 7.32 (d, *J* = 7.8 Hz, 2H), 2.38 (s, 3H), 2.36 (s, 3H). ^13^C{^1^H} NMR (126 MHz, DMSO-*d*_6_) *δ* 207.77, 139.26, 131.76, 131.04, 129.27, 129.26, 128.44, 126.93, 39.50, 21.52. LC/MS *m/z*: 322.1 [M + H]^+^.

###### 4.1.1.3.61. (E)-N′-(1-(2,5-Dichlorophenyl)ethylidene)-4-methylbenzohydrazide (BE5033).

White solid powder, mp: 191.6–193.8 °C, yield: 118.5 mg (79%). ^1^H NMR (500 MHz, DMSO-*d*_6_) *δ* 10.77 (s, 1H), 7.86–7.71 (m, 2H), 7.55 (d, *J* = 5.4 Hz, 1H), 7.52 (dd, *J* = 3.3, 2.3 Hz, 1H), 7.49 (d, *J* = 2.5 Hz, 1H), 7.31 (d, *J* = 7.7 Hz, 2H), 2.38 (s, 3H), 2.35 (s, 3H). ^13^C{^1^H} NMR (126 MHz, DMSO-*d*_6_) *δ* 167.24, 167.23, 150.73, 141.20, 132.23, 131.89, 130.37, 130.28, 129.26, 128.39, 115.56, 115.53, 115.27, 21.50. LC/MS *m/z*: 322.1 [M + H]^+^.

###### 4.1.1.3.62. (E)-N′-(1-(4-(Tert-butyl)phenyl)ethylidene)-4-methylbenzohydrazide (BE5034).

White solid powder, mp: 192.3–195 °C, yield: 204 mg (68%). ^1^H NMR (500 MHz, DMSO-*d*_6_) *δ* 10.64 (s, 1H), 7.79 (s, 4H), 7.45 (d, *J* = 7.9 Hz, 2H), 7.31 (d, *J* = 7.8 Hz, 2H), 2.38 (s, 3H), 2.34 (s, 3H), 1.30 (s, 9H). ^13^C{^1^H} NMR (126 MHz, DMSO-*d*_6_) *δ* 211.60, 167.60, 152.33, 135.87, 132.13, 129.27, 128.38, 126.65, 125.57, 96.11, 34.89, 31.47, 21.50. LC/MS *m/z*: 309.1 [M + H]^+^.

###### 4.1.1.3.63. (E)-N′-(1-Phenylethylidene)isonicotinohydrazide (BE21478) [51].

White solid, mp:180–182 °C, yield: 125 mg (93%). ^1^H NMR (500 MHz, DMSO-*d*_6_) *δ* 11.03 (s, 1H), 8.80–8.70 (m, 2H), 7.91–7.76 (m, 3H), 7.60 (d, *J* = 22.8 Hz, 1H), 7.49–7.41 (m, 2H), 7.34 (s, 1H), 2.39 (s, 3H). LC/MS *m/z*: 240.1 [M + H]^+^.

###### 4.1.1.3.64. (E)-N′-(Naphthalen-2-ylmethylene)isonicotinohydrazide (BE5048).

Yellow solid, mp: 210–211.4 °C, yield: 74 mg (63%). ^1^H NMR (500 MHz, DMSO-*d*_6_) *δ* 12.18 (s, 1H), 8.83–8.78 (m, 2H), 8.63 (s, 1H), 8.19 (s, 1H), 8.05–7.98 (m, 3H), 7.97–7.92 (m, 1H), 7.89–7.83 (m, 2H), 7.60–7.54 (m, 2H). ^13^C{^1^H} NMR (126 MHz, DMSO-*d*_6_) *δ* 162.16, 150.82, 149.43, 140.98, 134.35, 133.31, 132.25, 129.61, 129.05, 128.87, 128.27, 127.76, 127.29, 123.14, 122.04. LC/MS *m/z*: 276.1 [M + H]^+^.

###### 4.1.1.3.65. (E)-N′-(1-(Naphthalen-2-yl)ethylidene)isonicotinohydrazide (BE5049) [52].

Yellow solid, mp: 222.4–226.1 °C, yield: 121 mg (88%). ^1^H NMR (500 MHz, DMSO-*d*_6_) *δ*. 11.10 (s, 1H), 8.83–8.72 (m, 2H), 8.36 (s, 1H), 8.17 (dd, *J* = 17.4, 7.7 Hz, 1H), 8.06–7.99 (m, 1H), 7.96 (d, *J* = 8.6 Hz, 2H), 7.85 (dd, *J* = 11.0, 5.5 Hz, 2H), 7.60–7.47 (m, 2H), 2.52 (s, 3H). ^13^C{^1^H} NMR (126 MHz, DMSO-*d*_6_) δ 167.38, 156.91, 150.62, 141.63, 135.69, 133.94, 133.17, 129.12, 128.24, 127.98, 127.53, 127.32, 127.00, 124.11, 122.38, 15.09. LC/MS *m/z*: 290.1 [M + H]^+^.

###### 4.1.1.3.66. (E)-N′-(4-Methylbenzylidene)isonicotinohydrazide (BE5050) [53].

White solid, mp: 201–202.4 °C, yield: 92 mg (86%). ^1^H NMR (500 MHz, DMSO-*d*_6_) *δ* 12.00 (s, 1H), 8.80–8.75 (m, 2H), 8.43 (s, 1H), 7.85–7.79 (m, 2H), 7.67–7.61 (m, 2H), 7.29 (d, *J* = 7.9 Hz, 2H), 2.35 (s, 3H). ^13^C{^1^H} NMR (126 MHz, DMSO-*d*_6_) *δ* 167.78, 161.98, 150.79, 149.53, 141.01, 140.75, 131.80, 129.98, 127.73, 121.98, 21.53. LC/MS *m/z*: 240.1 [M + H]^+^.

###### 4.1.1.3.67. (E)-N′-(1-(p-Tolyl)ethylidene)isonicotinohydrazide (BE21479).

White solid, mp: 202–203 °C, yield: 112 mg (84%). ^1^H NMR (500 MHz, DMSO-*d*_6_) *δ* 10.97 (s, 1H), 8.76 (d, *J* = 5.0 Hz, 2H), 7.78 (dd, *J* = 13.8, 6.4 Hz, 4H), 7.26 (d, *J* = 7.8 Hz, 2H), 2.35 (s, 6H). ^13^C{^1^H} NMR (126 MHz, DMSO-*d*_6_) *δ* 150.36, 130.23, 128.87, 128.85, 127.03, 122.44, 40.05, 39.89, 39.72, 39.55, 15.31. LC/MS *m/z*: 254.1 [M + H]^+^.

###### 4.1.1.3.68. (E)-N′-(1-(4-Methoxyphenyl)ethylidene)isonicotinohydrazide (BE21480).

White solid, mp: 204.4–207.8 °C, yield: 129 mg (96%). ^1^H NMR (500 MHz, DMSO-*d*_6_) *δ* 10.96 (s, 1H), 8.79–8.67 (m, 2H), 7.87–7.76 (m, 4H), 7.04–6.96 (m, 2H), 3.81 (s, 3H), 2.34 (s, 3H). ^13^C{^1^H} NMR (126 MHz, DMSO-*d*_6_) *δ* 162.64, 161.11, 157.73, 150.58, 149.85, 141.68, 130.62, 128.60, 122.28, 114.22, 55.73, 15.15. LC/MS *m/z*: 270.1 [M + H]^+^.

###### 4.1.1.3.69. (E)-N′-(4-(Trifluoromethyl)benzylidene)isonicotinohydrazide (BE5051).

White solid, mp: 189–192 °C, yield: 114 mg (93%). ^1^H NMR (500 MHz, DMSO-*d*_6_) δ 12.26 (s, 1H), 8.84–8.78 (m, 2H), 8.54 (s, 1H), 7.98 (d, *J* = 8.1 Hz, 2H), 7.86–7.80 (m, 4H). ^13^C{^1^H} NMR (126 MHz, DMSO-*d*_6_) *δ* 167.43, 166.70, 162.34, 150.84, 147.69, 140.72, 138.46, 130.56, 129.96, 128.32, 126.25, 126.22, 125.62, 123.54, 123.45, 122.01. LC/MS *m/z*: 294.1 [M + H]^+^.

###### 4.1.1.3.70. (E)-N′-(2-Chlorobenzylidene)isonicotinohydrazide (BE5052) [54].

White solid, mp: 225–226.7 °C, yield: 98 mg (81%). ^1^H NMR (500 MHz, DMSO-*d*_6_) δ 12.28 (s, 1H), 8.88 (s, 1H), 8.82–8.78 (m, 2H), 8.04 (dd, *J* = 7.4, 2.2 Hz, 1H), 7.87–7.81 (m, 2H), 7.55 (dd, *J* = 7.5, 1.7 Hz, 1H), 7.47 (hd, *J* = 7.3, 6.5, 1.7 Hz, 2H). ^13^C{^1^H} NMR (126 MHz, DMSO-*d*_6_) *δ* 162.15, 150.85, 145.36, 140.67, 133.87, 132.29, 131.77, 130.46, 128.17, 127.46, 122.00. LC/MS *m/z*: 260.1 [M + H]^+^.

###### 4.1.1.3.71. (E)-N′-(Quinoxalin-2-ylmethylene)isonicotinohydrazide (BE5056).

Brown solid, mp: 226–228.3 °C, yield: 76 mg (86%). ^1^H NMR (500 MHz, DMSO-*d*_6_) *δ* 12.54 (s, 1H), 9.45 (s, 1H), 8.89–8.73 (m, 2H), 8.63 (s, 1H), 8.19–8.02 (m, 2H), 7.92–7.83 (m, 4H). ^13^C{^1^H} NMR (126 MHz, DMSO-*d*_6_) *δ* 169.10, 162.63, 150.95, 148.63, 147.51, 143.28, 142.16, 141.72, 140.46, 131.37, 129.62, 122.07. LC/MS *m/z*: 278.1 [M + H]^+^.

###### 4.1.1.3.72. (E)-N′-((3-Methyl-1H-pyrazol-4-yl)methylene)isonicotinohydrazide (BE5054).

White solid, mp: 250.0 °C, yield: 96 mg (78%). ^1^H NMR (500 MHz, DMSO-*d*_6_) *δ* 12.89 (d, *J* = 40.8 Hz, 1H), 11.71 (s, 1H), 8.76 (dt, *J* = 4.4, 1.5 Hz, 2H), 8.41 (s, 1H), 7.79 (dt, *J* = 4.4, 1.4 Hz, 2H), 7.73 (s, 1H), 2.39 (s, 3H). ^13^C{^1^H} NMR (126 MHz, DMSO-*d*_6_) *δ* 167.87, 161.37, 150.73, 149.86, 148.17, 143.92, 141.25, 123.43, 121.91, 121.89, 10.27. LC/MS *m/z*: 230.1 [M + H]^+^.

###### 4.1.1.3.73. (E)-N′-(Thiazol-2-ylmethylene)isonicotinohydrazide (BE5055).

White solid, mp: 222.1–223.8 °C, yield: 105 mg (87%). ^1^H NMR (500 MHz, DMSO-*d*_6_) *δ* 12.36 (s, 1H), 8.81 (dt, *J* = 4.2, 2.2 Hz, 2H), 8.70–8.65 (m, 1H), 8.02–7.97 (m, 1H), 7.94–7.87 (m, 1H), 7.82 (dd, *J* = 4.6, 1.6 Hz, 2H). ^13^C{^1^H} NMR (126 MHz, DMSO-*d*_6_) *δ* 167.75, 167.37, 164.31, 162.32, 150.93, 144.66, 143.71, 140.46, 122.92, 121.98. LC/MS *m/z*: 233.1 [M + H]^+^.

###### 4.1.1.3.74. (E)-N′-((4-Methylthiazol-5-yl)methylene)isonicotinohydrazide (BE5058).

Yellow solid, mp: 250.0 °C, yield: 106 mg (89%). ^1^H NMR (500 MHz, DMSO-*d*_6_) *δ* 12.08 (s, 1H), 9.10 (s, 1H), 8.83–8.77 (m, 2H), 8.76 (s, 1H), 7.84–7.78 (m, 2H), 2.51 (s, 3H). ^13^C {^1^H} NMR (126 MHz, DMSO-*d*_6_) *δ* 189.09, 167.37, 161.76, 155.81, 155.22, 142.50, 140.70, 127.82, 121.86, 15.83. LC/MS *m/z*: 247.1 [M + H]^+^.

###### 4.1.1.3.75. (E)-N′-(Thiazol-5-ylmethylene)isonicotinohydrazide (BE5059).

Yellow solid, mp: 245–247.8 °C, yield: 102 mg (86%). ^1^H NMR (500 MHz, DMSO-*d*_6_) *δ* 12.21 (s, 1H), 9.20 (s, 1H), 8.79 (dt, *J* = 4.5, 1.1 Hz, 2H), 8.75 (s, 1H), 8.33 (s, 1H), 7.81 (dt, *J* = 4.5, 1.1 Hz, 2H). ^13^C{^1^H} NMR (126 MHz, DMSO-*d*_6_) *δ* 167.87, 162.10, 157.10, 150.85, 146.84, 142.15, 140.69, 135.08, 123.62, 121.97. LC/MS *m/z*: 233.1 [M + H]^+^.

###### 4.1.1.3.76. (E)-N′-((5-Methyl-1H-imidazol-4-yl)methylene)isonicotinohydrazide (BE5060) [55].

Yellow solid, mp: 244.1–243.4 °C, yield: 340 mg (88%). ^1^H NMR (500 MHz, DMSO-*d*_6_) *δ* 12.19 (d, *J* = 9.9 Hz, 1H), 8.81 (t, *J* = 11.6 Hz, 3H), 8.50 (d, *J* =9.7 Hz, 1H), 7.83 (q, *J* = 5.3 Hz, 2H), 2.43 (d, *J* = 10.2 Hz, 3H). LC/MS *m/z*: 230.1 [M + H]^+^.

### 4.2. Co-transfection luciferase assay

HEK293 cells (ATCC) were maintained in Dulbecco’s modified Eagle’s medium (DMEM) supplemented with 10% fetal bovine serum at 37 ° C under 5% CO_2_. Twenty-four hours prior to transfection HEK293 cells were plated in 96-well plates at a density of 32 × 10^3^ cells/well. Transfections were performed using Lipofectamine^™^ 3000 (Invitrogen). Each well was transfected with 2.5 μL (1000 ng/μL) of ERRE (reporter) (Addgene plasmid # 37851) [56], and 1.0 μL (500 ng/μL) of ERRα (Addgene plasmid # 10975) [57] or ERRγ (Addgene plasmid # 120435) [58]. Eight hours post-transfection, the cells were treated with vehicle or ligands. Twenty-four hours post-treatment, the luciferase activity was measured using the One-Glo^™^ luciferase assay system (Promega). Reporter activity was quantified as raw luminescence values and normalized to identically transfected vehicle-treated wells. Data are presented as relative luciferase activity compared to vehicle control. The values indicated represent the means ± S.E. from four independently transfected wells. The experiments were repeated at least three times.

### 4.3. Kinetic solubility

From a 10 mM DMSO stock, 2 μL compound was added to 198 μL prewarmed pH 7.4 phosphate buffered saline in a 96-well plate. The plate was maintained at ambient temperature for 24 h on an orbital shaker. Samples were centrifuged through a Millipore Multiscreen Solvinter 0.45 μm low-binding PTFE hydrophilic filter plate and analyzed by LC-MS/MS or HPLC. The peak area was compared to standards of known concentration.

### 4.4. Liver microsome stability assays

Microsome stability was evaluated by incubating 1 μM test compound with 1 mg/mL hepatic microsomes in 100 mM KPi, pH 7.4 at 37 ° C with shaking. The reaction was initiated by adding NADPH (1 mM final concentration). Aliquots were removed at 0, 5, 10, 20, 40, and 60 min and added to acetonitrile (5× v:v) to stop the reaction and precipitate the protein. NADPH dependence of the reaction was evaluated with ─NADPH samples. At the end of the assay, the samples were centrifuged through a Millipore Multiscreen Solvinter 0.45 μm low binding PTFE hydrophilic filter plate and analyzed by LC-MS/MS. Data is log transformed and represented as half-life and intrinsic clearance.

### 4.5. Computational methods

Models of ERRα bound with **BE5112**, **BE5138**, **BE5040**, **BE5049**, and **SLU-PP-332** were built from X-ray crystal structures of ERRα-**DS45500853** (PDB: 7E2E) [24]. Ligands were modeled using UCSF Chimera [59]. Each complex was first energy-minimized *in vacuum* using the steepest descent and conjugate gradient methods while keeping the ligand constrained, followed by an additional energy minimization of the entire complex using Amber [60]. Complexes were neutralized and solvated with an octahedral water box of TIP3P water molecules using Tleap. The FF14SB force field parameters were used for all receptor residues [61], and the general Amber force field (GAFF) was applied to ligand molecules [62,63]. The system temperature was gradually increased to 300 K under isothermal–isovolumetric (NVT) dynamics using a Langevin thermostat with a 2 fs timestep and a 1.0 ps^−1^ collision frequency, applying progressively weaker restraints on the complex over 500 ps. Equilibration to ambient conditions (300 K, 1 atm) was then carried out under isothermal–isobaric (NPT) dynamics with gradually reduced restraints over an additional 500 ps, employing an isotropic Berendsen barostat with a 0.2 ps relaxation time. Subsequently, the system was equilibrated for 200 ps under NVT dynamics without restraints, after which two independent production runs of 400 ns each were performed with a 2 fs timestep under NVT dynamics for each complex system. Three-dimensional periodic boundary condition was applied for all simulations. A 10.0 Å cutoff distance was used for nonbonded interactions, and long-range electrostatic interactions were computed using the particle mesh Ewald (PME). Bonds involving hydrogen atoms were constrained at their equilibrium length via SHAKE algorithm. Ligand-protein interactions were analyzed with CPPTRAJ [64], and binding free energies of complexes were determined using MM/GBSA [65]. Clustering analysis was performed with a hierarchical agglomerative (bottom-up) approach, applying a minimum average distance of 3.0 Å between clusters.

Docking of the selected ligands was performed using Glide (Schrödinger) [66]. Nine representative ERRα receptor conformations were obtained from K-means clustering of the molecular dynamics (MD) trajectories of the ERRα complexes. The top three representative conformations from each complex, accounting for 43.4%, 51.7%, and 80.6% of the simulation trajectories for **BE5112**, **BE5138**, and **SLU-PP-332**, respectively, were used for docking calculations.

### 4.6. Gene expressionCells were treated for 24 h with 10 μM of different compounds. After treatment, cells were harvested, and total RNA was extracted using RNeasy Mini Kit according to the manufacturer instruction. Total RNA was extracted using TRizol Reagent (Invitrogen) and then 0.2 μg of total RNA was reverse transcribed using AzuraFlex cDNA Synthesis Kit (Azura). qRT-PCR was carried out with the AzuraView GreenFast qPCR Blue Mix (Azura) using a QuantoStudio7 qPCR System with the following primer sets:

36b4-F ACCTCCTTCTTCCAGGCTT

36b4-R CCCACCTTGTCTCCAGTCTTT

PGC-1a-F CCACCGCCAACCAAGAGGGC

PGC-1a-R AGCCGGAGACTGGGCCGTTT

PDK4-F TCCTTCACACCTTCACCACA

PDK4-R AAAGAGGCGGTCAGTAATCC

All samples were run in triplicates, and the analysis was completed by determining ΔΔCt values. The reference gene used was 36b4, a ribosomal protein gene.

### 4.7. RNA sequencing and analysis

Poly(A)-selected RNA-seq libraries were prepared by Novogene from total RNA isolated from neonatal rat ventricular myocytes (NRVMs) after 72 h compound treatment (input: 200 ng; RIN ≥ 4, Agilent Bioanalyzer). Libraries were sequenced on an Illumina NovaSeq to generate paired-end 2 × 150 bp reads at ~20 million read pairs per sample (*n* = 3 per treatment). Vendor quality control assessed base quality, GC content, duplication, and adapter contamination; only libraries passing QC were analyzed. FASTQ files were aligned to the rat reference genome (Rnor_6.0, rn6) with HISAT2 (v2.0.5) using unstranded settings and default parameters. Gene-level counts were obtained with featureCounts (v1.5.0-p3) against the NCBI rn6 annotation (release 106), and expression was summarized as raw counts and FPKM. Differential expression was performed on raw counts using DESeq2 (v1.36.0) with default sizefactor normalization and the Wald test; *P* values were adjusted for multiple testing using the Benjamini–Hochberg procedure, and genes with *p*-value ≤0.05 were considered differentially expressed. Functional enrichment for GO terms and KEGG pathways was conducted with limma (v3.52.1). Dot plots of top enriched pathways were generated in Python using matplotlib (v3.10.1) and seaborn (v0.13.2). Heatmaps were generated in R by computing z-scores per gene across samples and plotted in GraphPad Prism (v10.6.0).

## Supplementary Material

1

## Figures and Tables

**Table 1:** In vitro ERRα and ERRγ agonistic activity of 4-hydroxy-benzohydrazides.

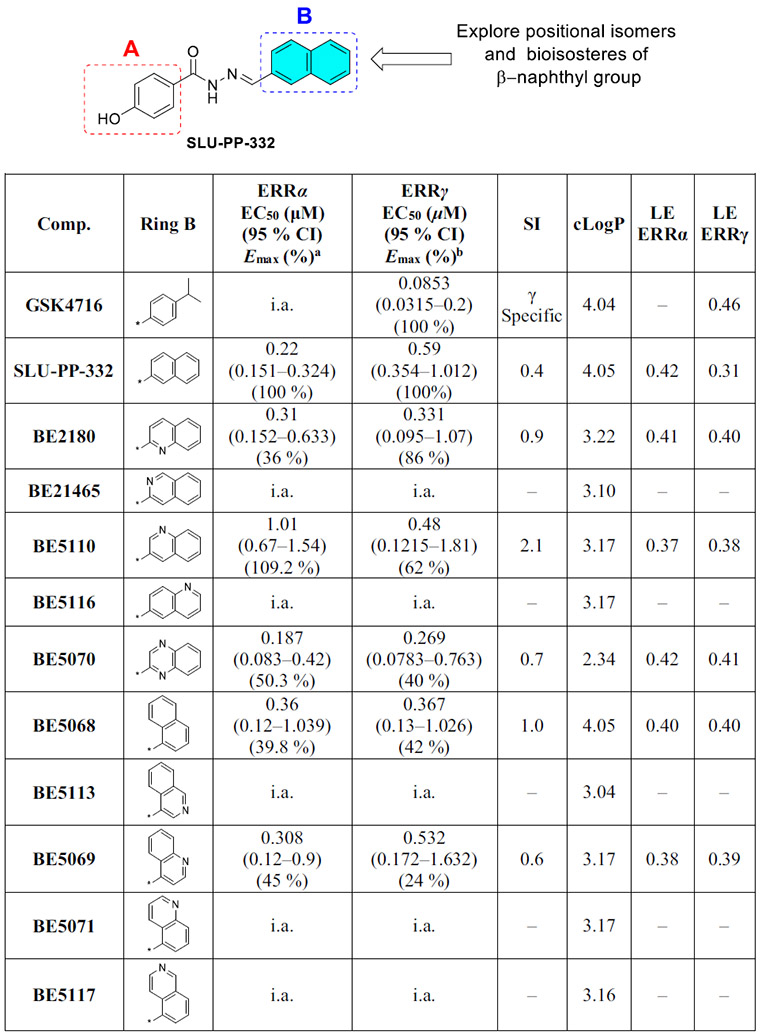

a *E*_max_ (%) Vs **SLU-PP-332** toward ERRα.

b *E*_max_ (%) Vs **SLU-PP-332** toward ERRγ.

CI = Confidence interval; i.a. = inactive; SI = EC_50_ (ERRα) / EC_50_ (ERRγ).

**Table 2:** In vitro ERRα and ERRγ agonistic activity of 4-methyl-benzohydrazides.

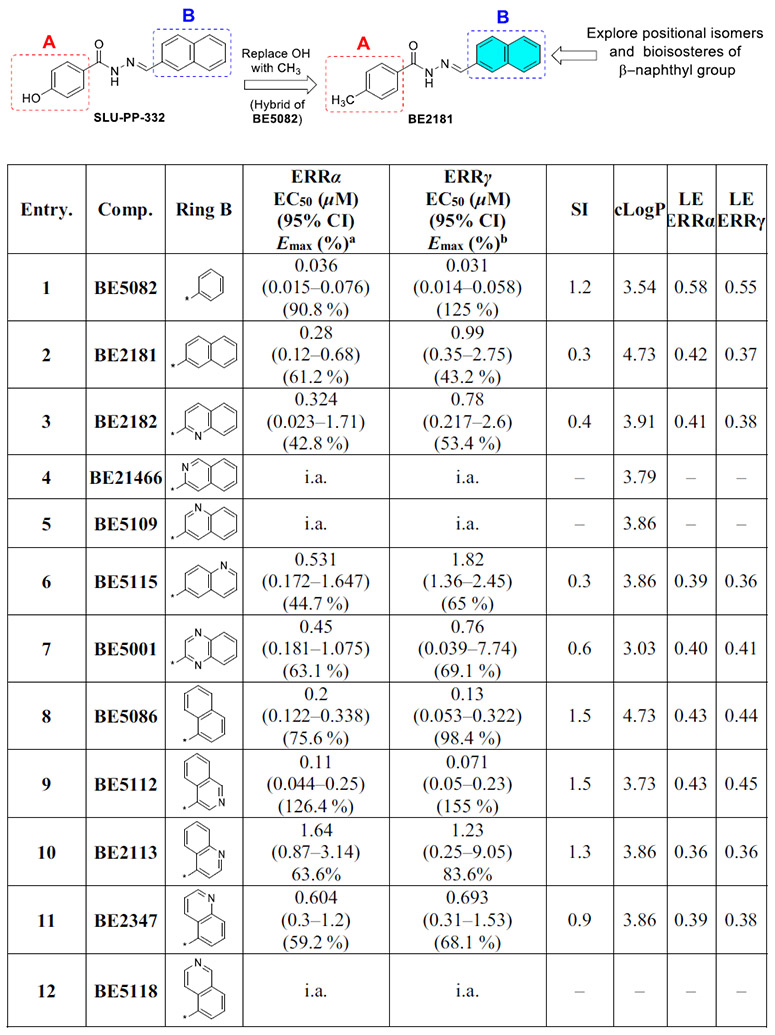

a *E*_max_ (%) Vs **SLU-PP-332** toward ERRα.

b *E*_max_ (%) Vs **SLU-PP-332** toward ERRγ.

CI = Confidence interval; i.a. = inactive; SI = EC_50_ (ERRα) / EC_50_ (ERRγ).

**Table 3:** In vitro ERRα and ERRγ agonistic activity of benzohydrazides.

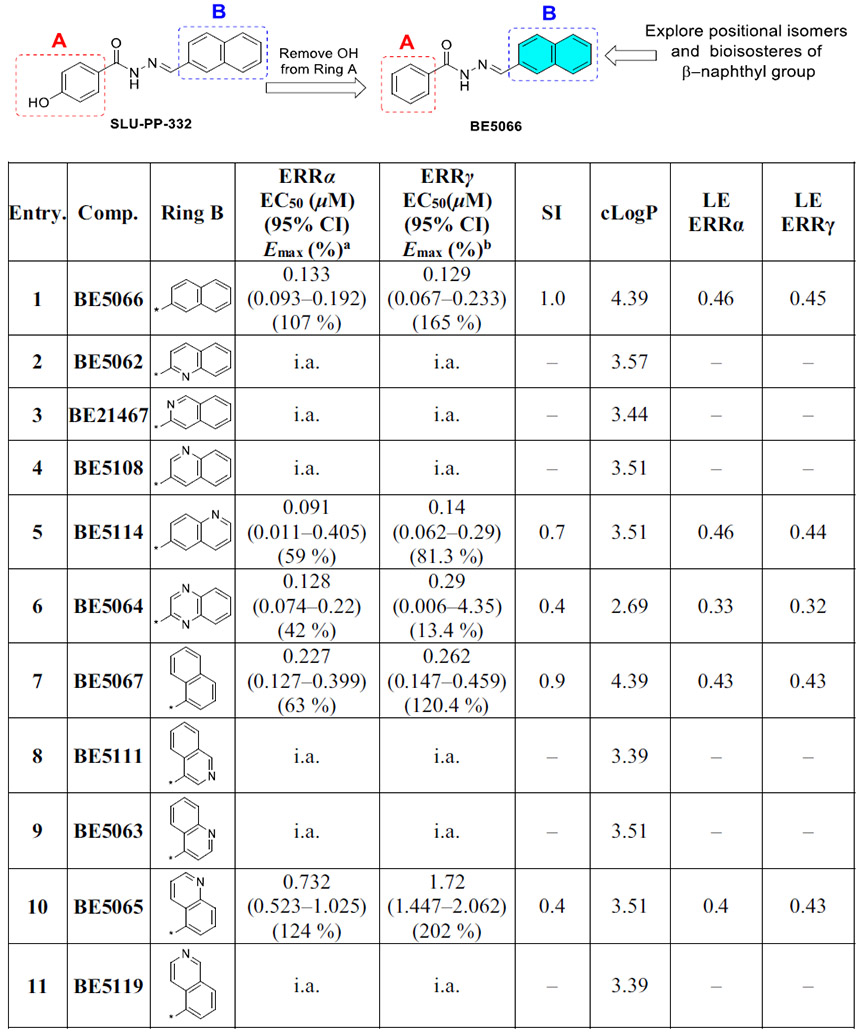

a *E*_max_ (%) Vs **SLU-PP-332** toward ERRα.

b *E*_max_ (%) Vs **SLU-PP-332** toward ERRγ.

CI = Confidence interval; i.a. = inactive; SI = EC_50_ (ERRα) / EC_50_ (ERRγ).

**Table 4:** In vitro ERRα and ERRγ agonistic activity of 3,4-dichlorobenzylidene-4-substituted-benzohydrazide.

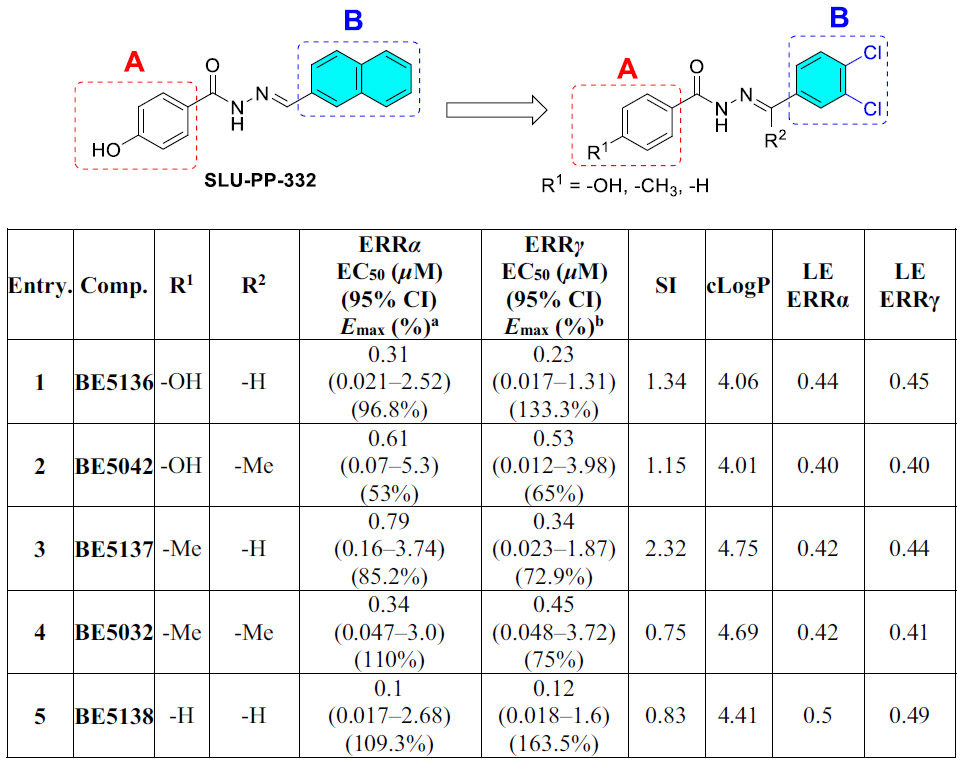

a *E*_max_ (%) Vs **SLU-PP-332** toward ERRα.

b *E*_max_ (%) Vs **SLU-PP-332** toward ERRγ.

CI = Confidence interval; i.a. = inactive; SI = EC_50_ (ERRα) / EC_50_ (ERRγ).

**Table 5:** In vitro ERRα and ERRγ agonistic activity of 4-hydroxy-ethylidene benzohydrazide.

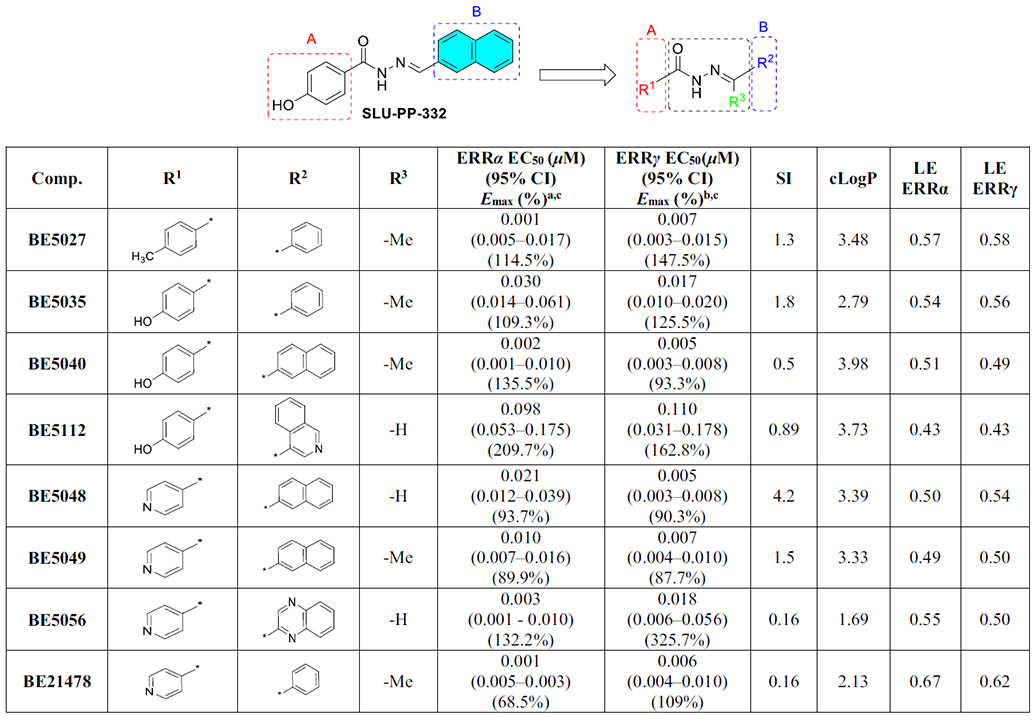

a *E*_max_ (%) Vs **SLU-PP-332** toward ERRα.

b *E*_max_ (%) Vs **SLU-PP-332** toward ERRγ.

c EC_50_ was calculated from the eleven-point assay run.

CI = Confidence interval; i.a. = inactive; SI = EC_50_ (ERRα) / EC_50_ (ERRγ).

**Table 6:** In-vitro ADME parameters of selected ERR agonists.

Comp.	cLogP	LE ERRα	LE ERRγ	Kinetic solubility (μM)	HLM t_1/2_ (min)	Human liver microsome Cl_int_ – (μL/min/mg)
SLU-PP-332	4.05	0.42	0.31	0.2	31.3	22
BE5082	3.54	0.58	0.55	3.1	18.1	38
BE5086	4.73	0.43	0.44	46.4	16.3	42
BE2180	3.22	0.41	0.40	11.2	47.7	15
BE5066	4.39	0.46	0.45	2.3	15.3	45
BE5112	3.73	0.43	0.43	21.7	6.6	105
BE5138	4.41	0.50	0.49	1.1	22	31
BE5049	3.33	0.49	0.50	7.1	39.3	18
BE5040	3.98	0.51	0.49	3	28.5	24
BE5027	3.48	0.57	0.58	66	11.7	59

**Table 7:** Binding energies of selected ERRα complexes in unit of kcal/mol.

Complex	ΔG_VDW_	SE	ΔG_EEL_	SE	ΔG_solv_	SE	ΔG_bind_	SE
ERRα-BE5112	− 44.18	0.05	− 14.60	0.06	24.81	0.05	− 33.96	0.05
ERRα-BE5138	− 40.21	0.06	− 19.53	0.10	25.38	0.08	− 34.36	0.07
ERRα-SLU-PP-332	− 37.12	0.06	− 28.43	0.13	30.12	0.09	− 35.43	0.08

## Data Availability

Data will be made available on request.

## Appendix A. Supplementary data

Supplementary data to this article can be found online at https://doi.org/10.1016/j.ijbiomac.2026.151450.
