# Supplementary material for: Chemical optimization of the exercise mimetic SLU-PP-332 enables insight into estrogen-related receptor signaling
Source: Int J Biol Macromol. Author manuscript; Available in PMC 2026 Apr 27. (PMC13112601; doi:10.1016/j.ijbiomac.2026.151450)

Chemical Optimization of the Exercise Mimetic SLU-PP-332 Enables Insight into Estrogen-Related Receptor Signaling

Contents

**1. Structure–Activity Relationship (SAR) Data Tables**	**S3-S7**
**2. Cell-Based Screening of Compounds in ERRα and ERRγ Assays**	**S8-S10**
**3. ^{1}H and ^{13}C NMR Spectra of Synthesized Compounds**	**S11-S80**
**4. Computational Figure S133**	**S80**
**4. LC–MS Characterization of ERR-Active Compounds**	**S81-S93**

1. Structure–Activity Relationship (SAR) Data Tables

Table S1. *In vitro* ERRα and ERRγ agonistic activity of 4-hydroxy-ethylidene benzohydrazide.

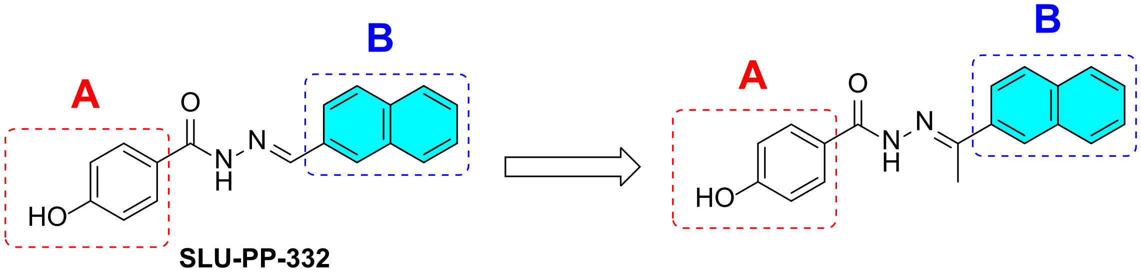

SLU-PP-332

**Comp.**	**Ring B**	**ERR*α* EC_{50} (*μ*M) E_{max} (%)[a]**	**ERR*γ* EC_{50} (*μ*M) E_{max} (%)[b]**	**cLogP**
**BE5039**	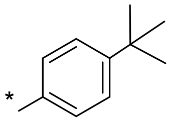	i.a.	i.a.	2.44
**BE5041**	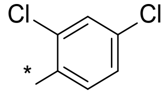	i.a.	i.a.	4.01
**BE5043**	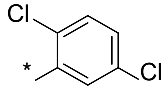	i.a.	i.a.	4.01
**BE5044**	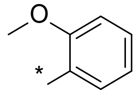	i.a.	i.a.	2.72
**BE5045**	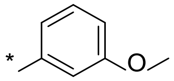	i.a.	i.a.	2.72
**BE5046**	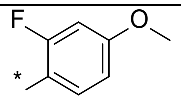	i.a.	i.a.	2.82
**BE5047**	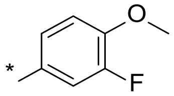	i.a.	i.a.	2.82

[a] E_{max} (%) Vs **SLU-PP-332** towards ERRα
[b] E_{max} (%) Vs **SLU-PP-332** towards ERRγ
i.a. = inactive

Table S2. *In vitro* ERRα and ERRγ agonistic activity of 4-methyl-ethylidene benzohydrazide.

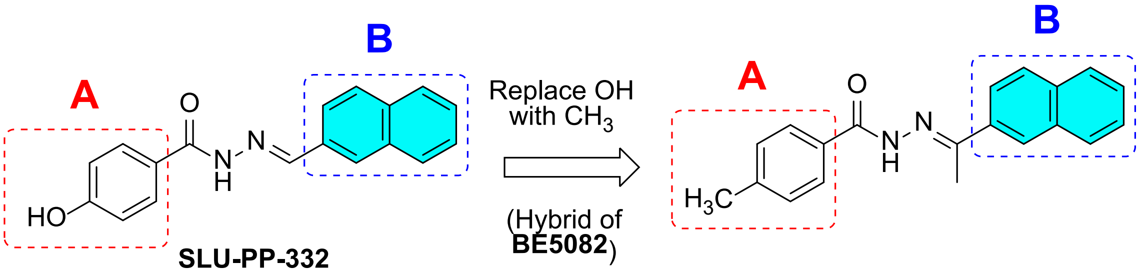

Comp.	Ring B	ERRα EC_{50} (μM) E_{max} (%)[a]	ERRγ EC_{50} (μM) E_{max} (%)[b]	cLogP
**BE5031**	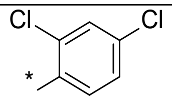	i.a.	i.a.	4.69
**BE5033**	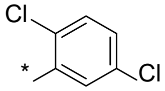	i.a.	i.a.	4.69
**BE5034**	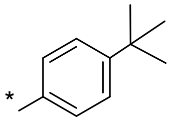	i.a.	i.a.	5.06

[a] E_{max} (%) Vs **SLU-PP-332** towards ERRα
[b] E_{max} (%) Vs **SLU-PP-332** towards ERRγ
i.a. = inactive

Table S3. *In vitro* ERRα and ERRγ agonistic activity of isoniazid benzohydrazide.

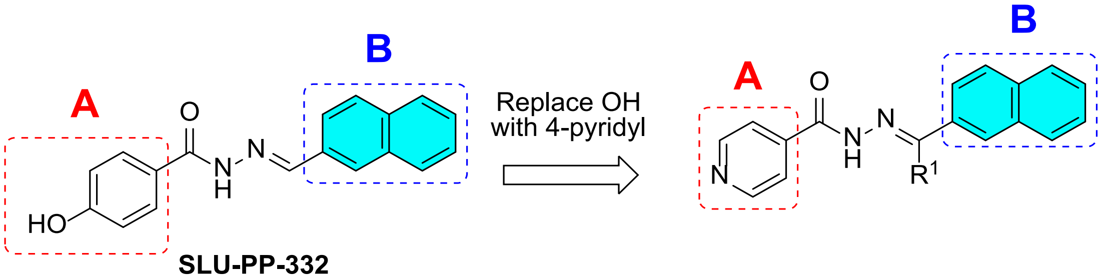

SLU-PP-332

Comp.	Ring B	R^1	ERR*α* EC_{50} (*μ*M) E_{max} (%)[a]	ERR*γ* EC_{50} (*μ*M) E_{max} (%)[b]	cLogP
**BE5050**	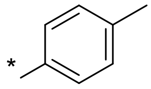	-H	i.a.	i.a.	2.54
**BE21479**	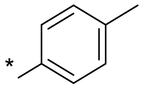	-Me	i.a.	i.a.	2.48
**BE21480**	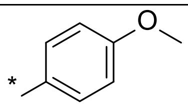	-Me	i.a.	i.a.	2.06
**BE5051**	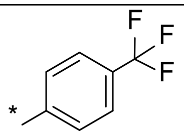	-H	i.a.	i.a.	3.05
**BE5052**	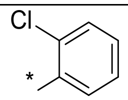	-H	i.a.	i.a.	2.8
**BE5054**	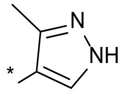	-H	i.a.	i.a.	0.49
**BE5055**	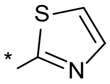	-H	i.a.	i.a.	1.03
**BE5058**	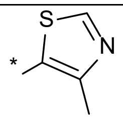	-H	i.a.	i.a.	1.45
**BE5059**	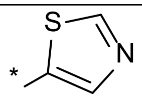	-H	i.a.	i.a.	1.06
**BE5060**	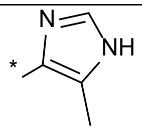	-H	i.a.	i.a.	0.81

Table S4. ***In vitro*** **ERRα and ERRγ agonistic activity of 4-hydroxy-benzenesulfonohydrazide**

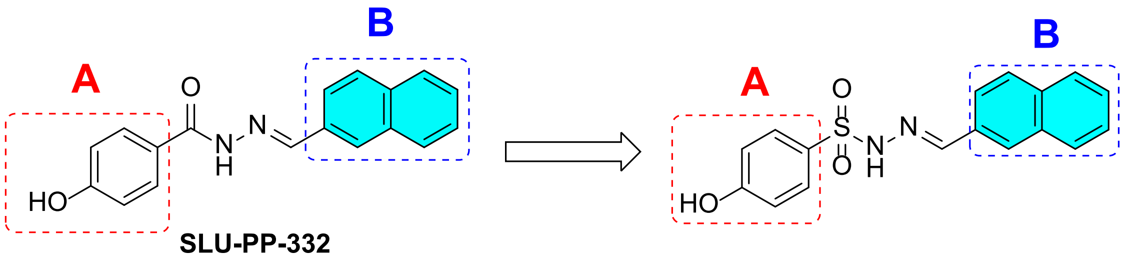

Comp.	Ring B	ERR*α* EC$_{50}$ (*μ*M) *E*$_{max}$ (%)[a]	ERR*γ* EC$_{50}$ (*μ*M) *E*$_{max}$ (%)[b]	cLogP
**BE21468**	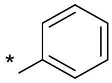	i.a.	i.a.	1.93
**BE21469**	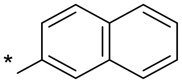	i.a.	i.a.	3.12
**BE21470**	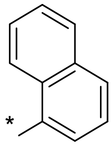	i.a.	i.a.	2.92
**BE21471**	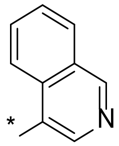	i.a.	i.a.	2.92
**BE21472**	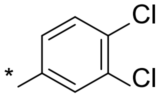	i.a.	i.a.	3.14

[a]*E*$_{max}$ (%) Vs SLU-PP-332 towards ERRα
[b]*E*$_{max}$ (%) Vs SLU-PP-332 towards ERRγ
i.a. = inactive

Table S5. ***In vitro*** **ERRα and ERRγ agonistic activity of 4-methyl-benzenesulfonohydrazide**

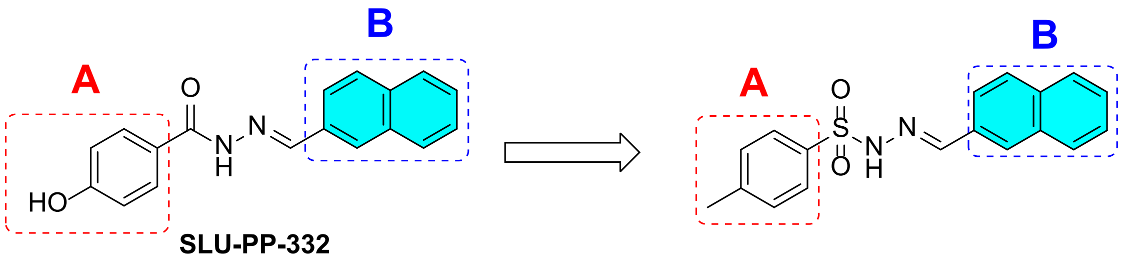

SLU-PP-332

**Comp.**	**Ring B**	**ERR*α* EC50 (*μ*M) *E*max (%)[a]**	**ERR*γ* EC50 (*μ*M) *E*max (%)[b]**	**cLogP**
**BE21446**	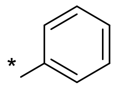	i.a.	i.a.	2.62
**BE21447**	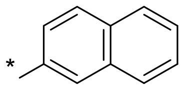	i.a.	i.a.	3.81
**BE21448**	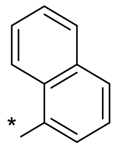	i.a.	i.a.	3.81
**BE21449**	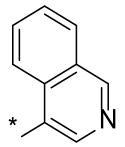	i.a.	i.a.	2.81
**BE21450**	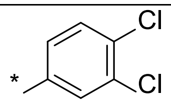	i.a.	i.a.	3.83

[a]*E*max (%) Vs SLU-PP-332 towards ERRα
[b]*E*max (%) Vs SLU-PP-332 towards ERRγ
i.a. = inactive

2. Cell-Based Screening of Compounds in ERRα and ERRγ Assays

ERR Alpha **ERR Gamma**

A

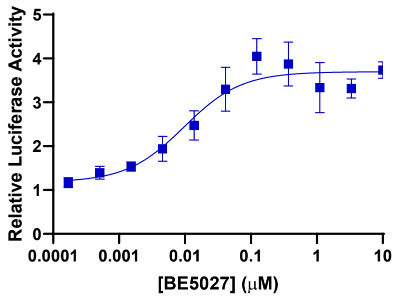

B

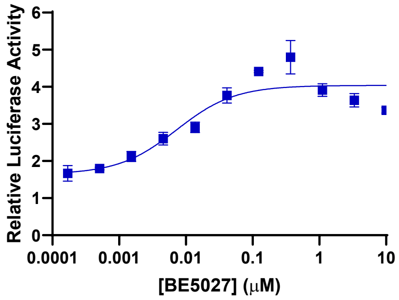

A

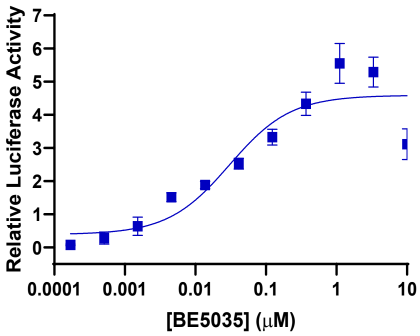

B

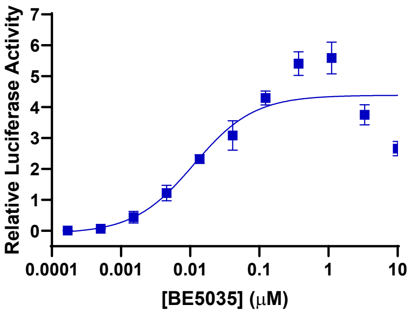

A

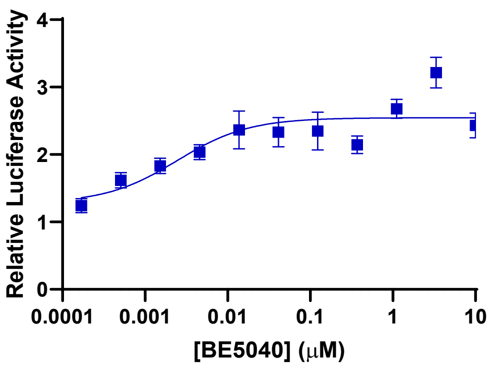

B

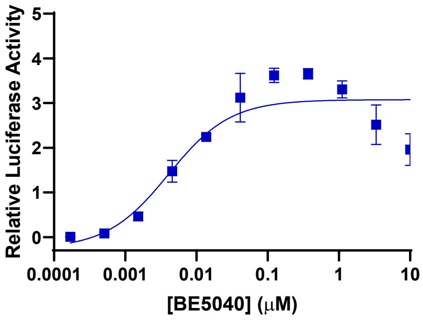

A

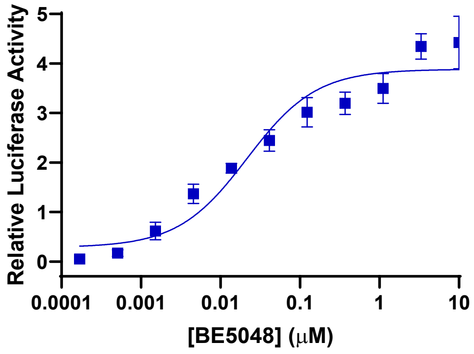
Relative Luciferase Activity
0
1
2
3
4
5
0.0001
0.001
0.01
0.1
1
10
[BE5048] (μM)

B

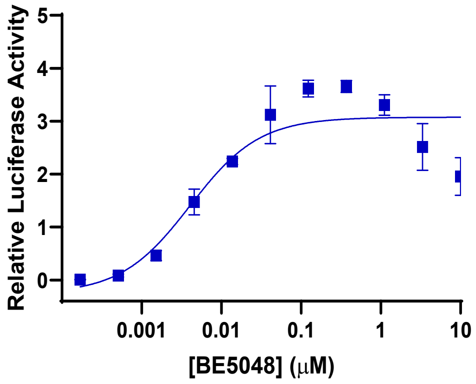
Relative Luciferase Activity
0
1
2
3
4
5
0.001
0.01
0.1
1
10
[BE5048] (μM)

A

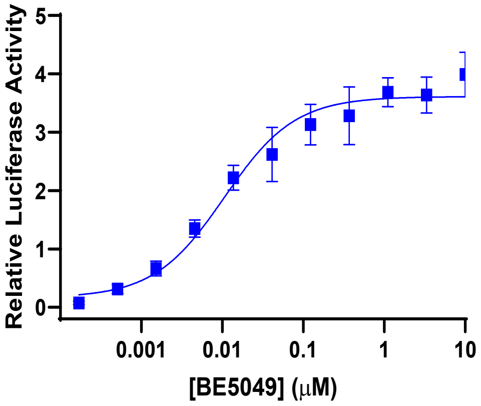
Relative Luciferase Activity
0
1
2
3
4
5
0.001
0.01
0.1
1
10
[BE5049] (μM)

B

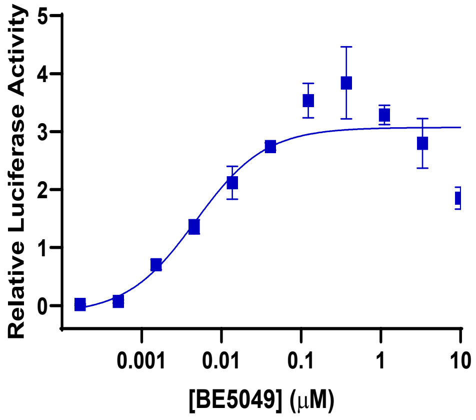
Relative Luciferase Activity
0
1
2
3
4
5
0.001
0.01
0.1
1
10
[BE5049] (μM)

A

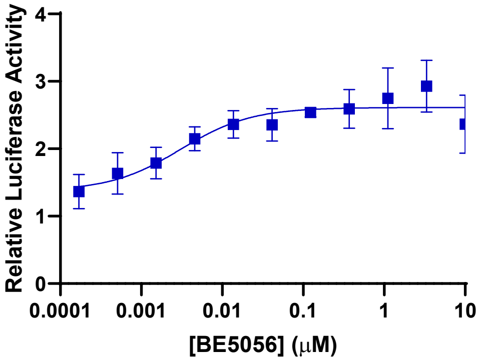
Relative Luciferase Activity
0
1
2
3
4
0.0001
0.001
0.01
0.1
1
10
[BE5056] (μM)

B

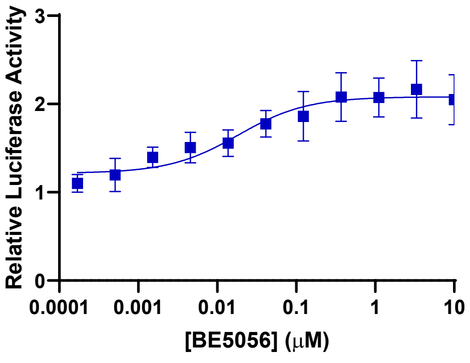
Relative Luciferase Activity
0
1
2
3
0.0001
0.001
0.01
0.1
1
10
[BE5056] (μM)

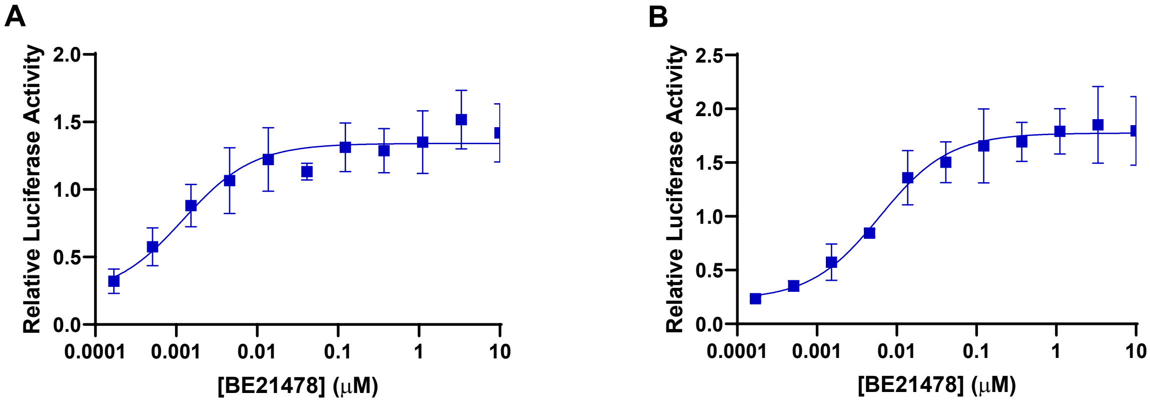

A
Relative Luciferase Activity
2.0
1.5
1.0
0.5
0.0
0.0001
0.001
0.01
0.1
1
10
[BE21478] (μM)
B
Relative Luciferase Activity
2.5
2.0
1.5
1.0
0.5
0.0
0.0001
0.001
0.01
0.1
1
10
[BE21478] (μM)

3. ^{1}H and ^{13}C NMR Spectra of Synthesized Compounds

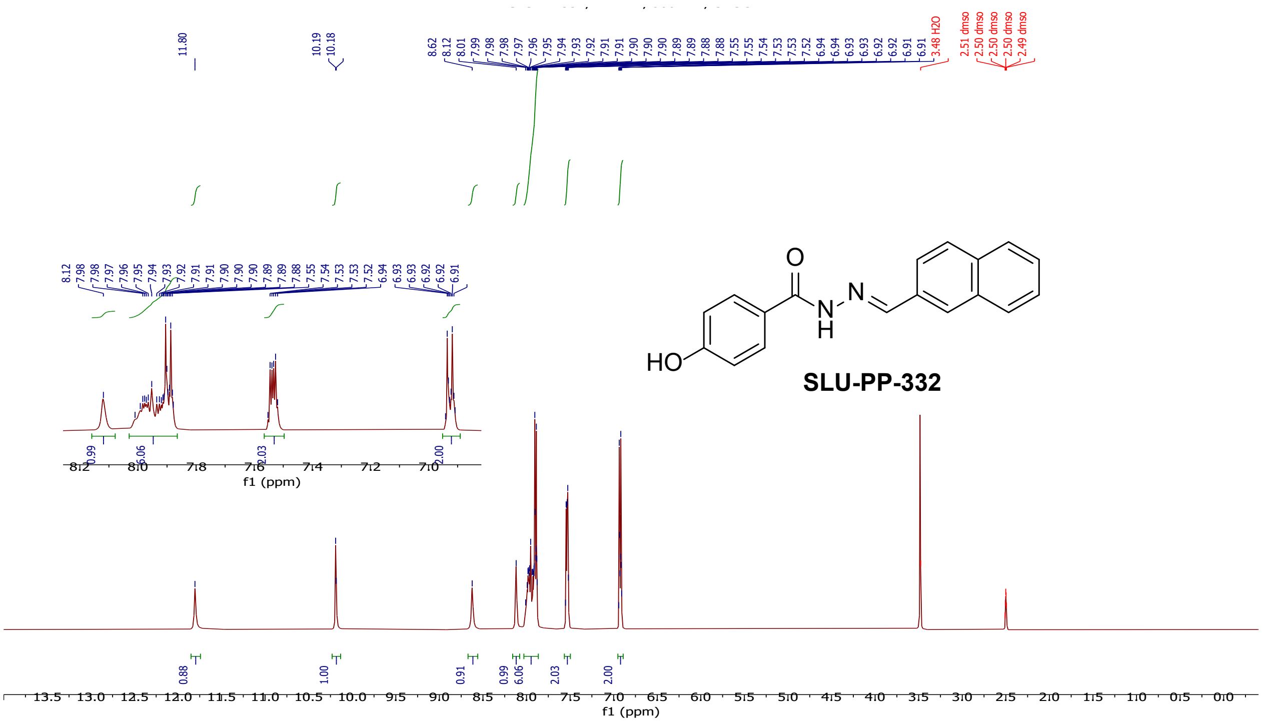

Figure S1. ^{1}H **NMR spectra of compound SLU-PP-332**

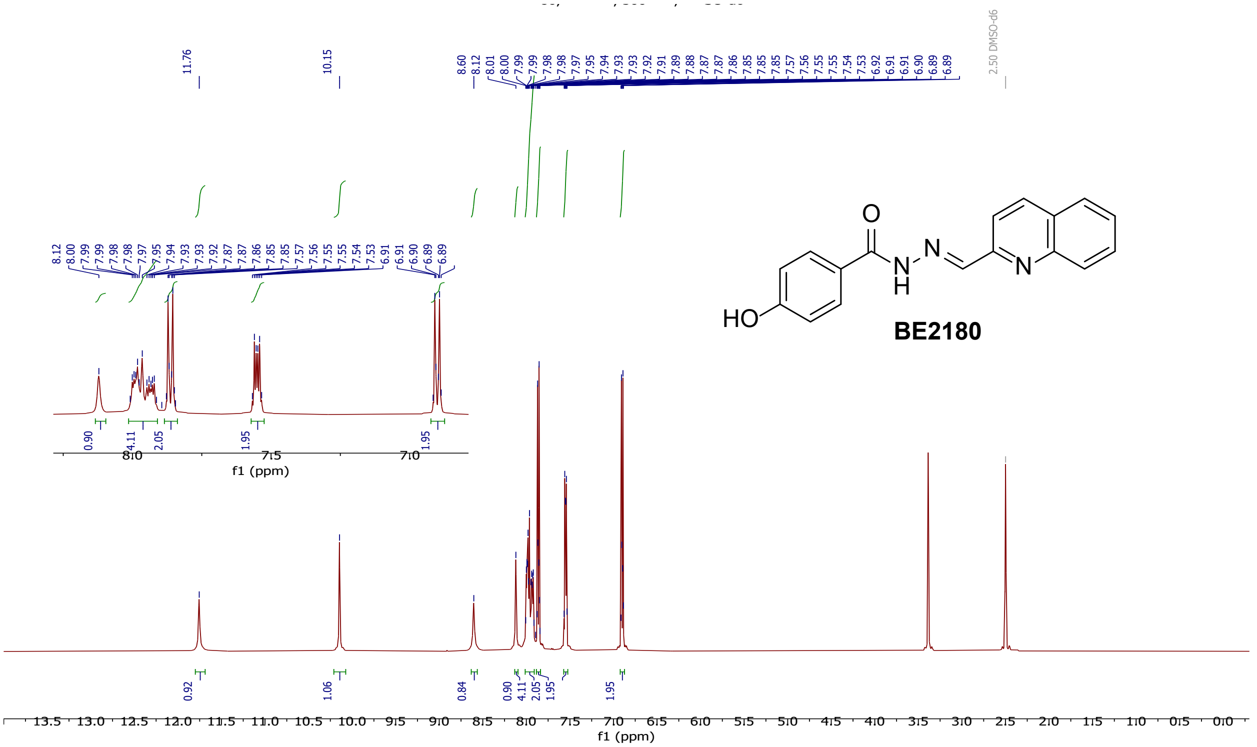

Figure S2. ^{1}H **NMR spectra of compound BE2180**

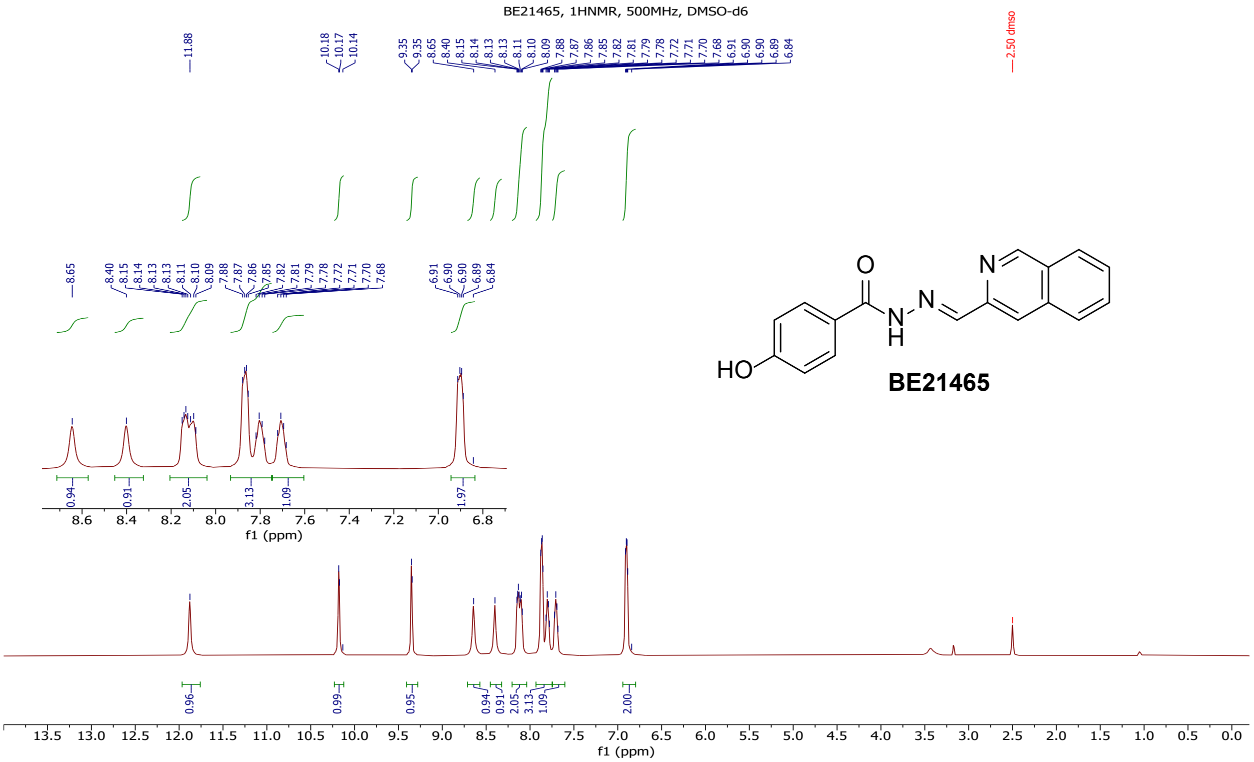

Figure S3. ^{1}H NMR spectra of compound BE21465

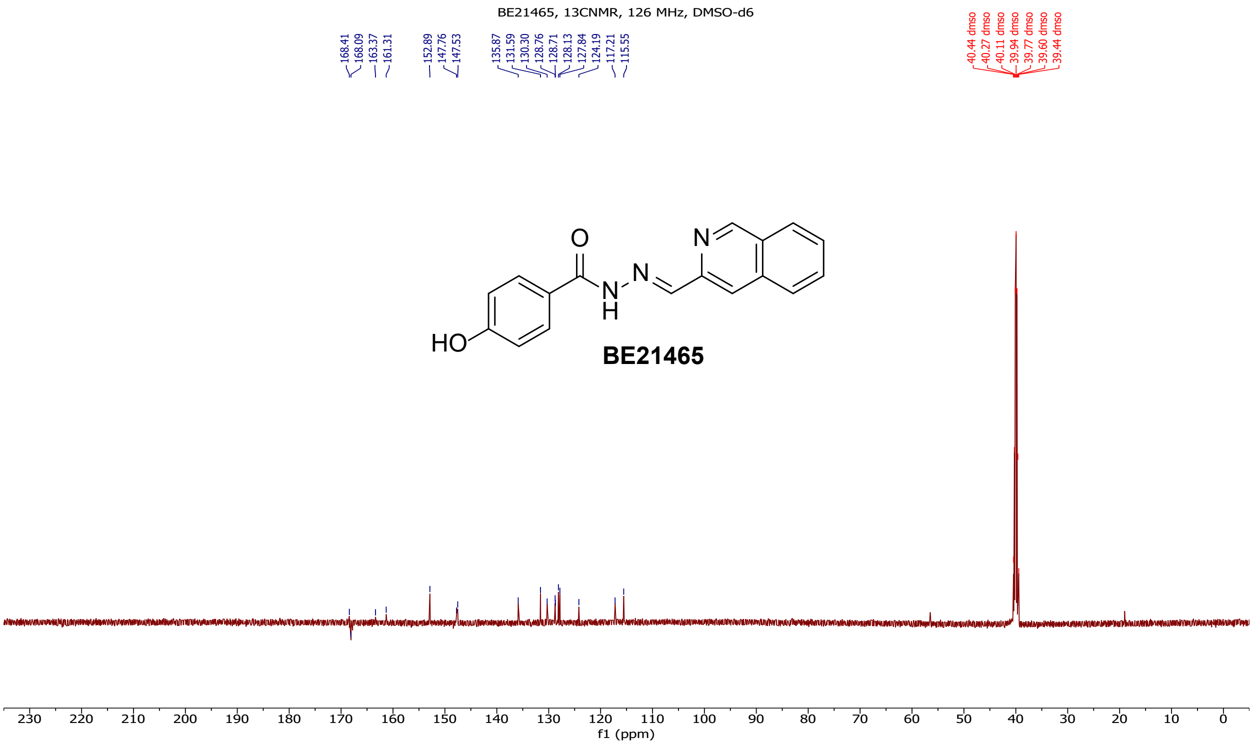

Figure S4. ^{13}C{^{1}H} NMR spectra of compound BE21465

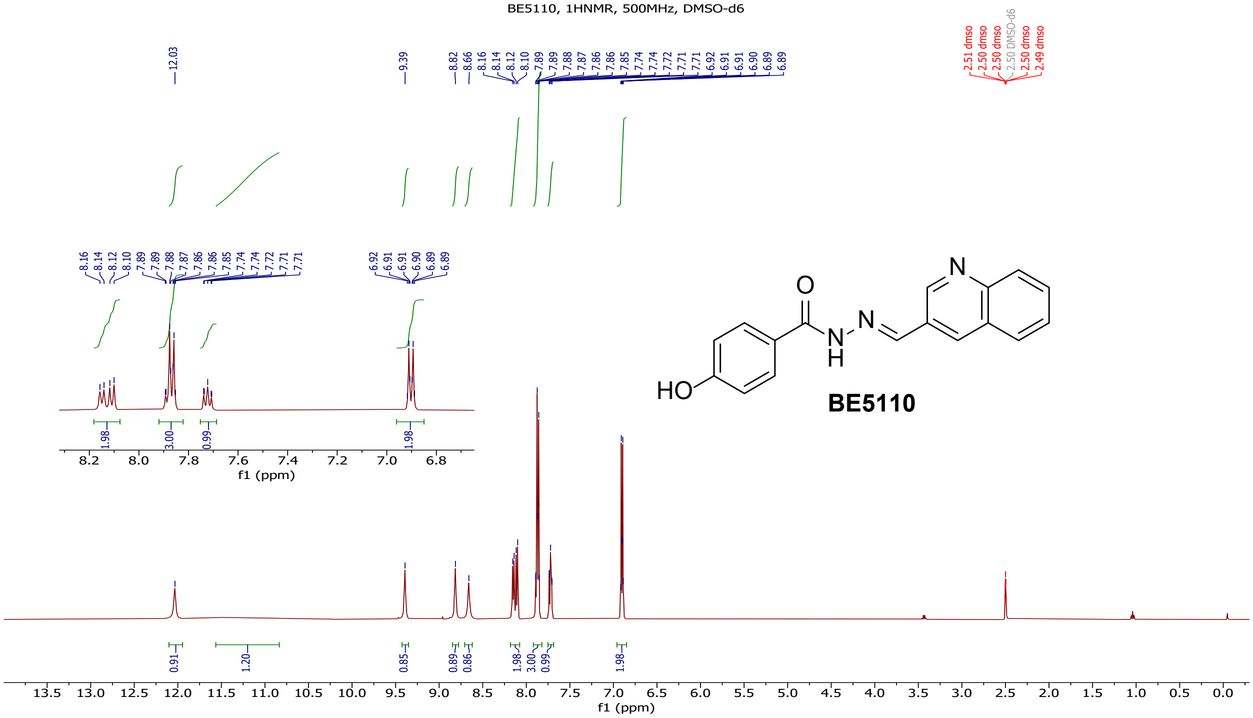

Figure S5. ^{1}H **NMR spectra of compound BE5110**

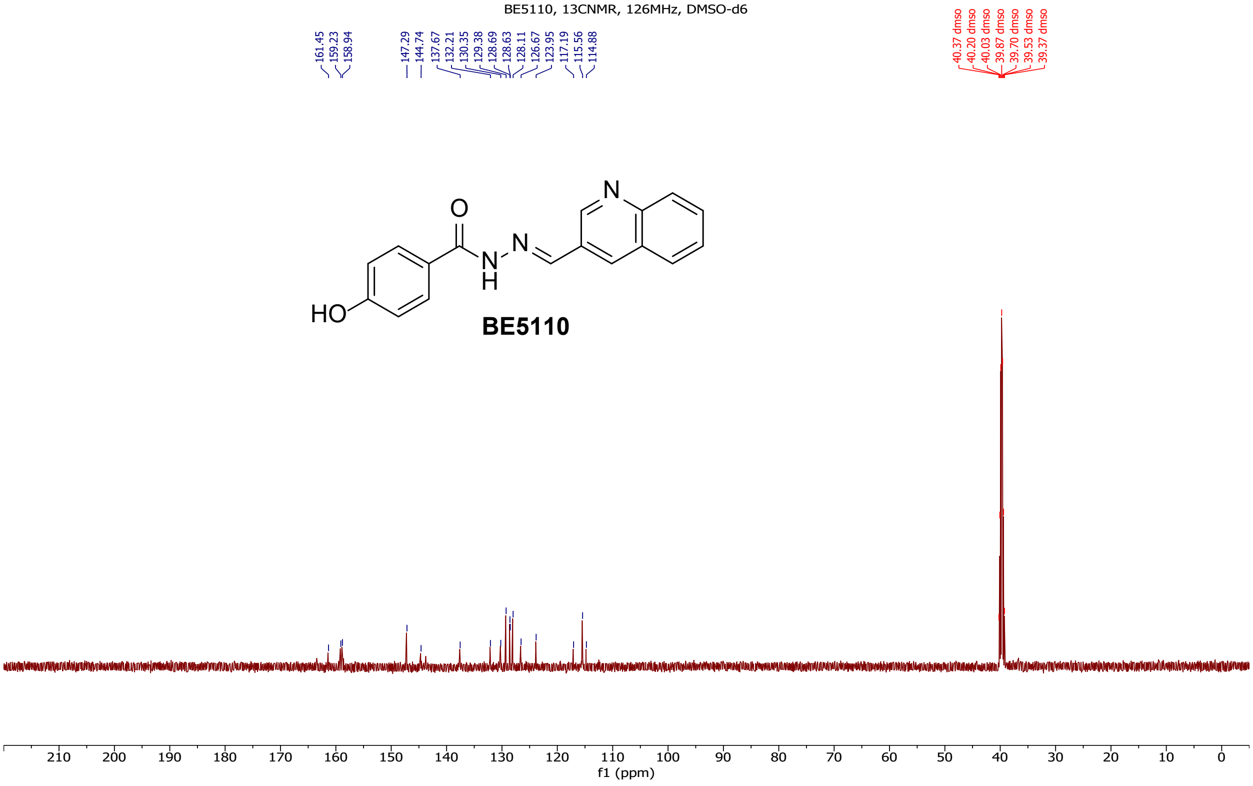

Figure S6. $^{13}C\{^{1}H\}$ **NMR spectra of compound BE5110**

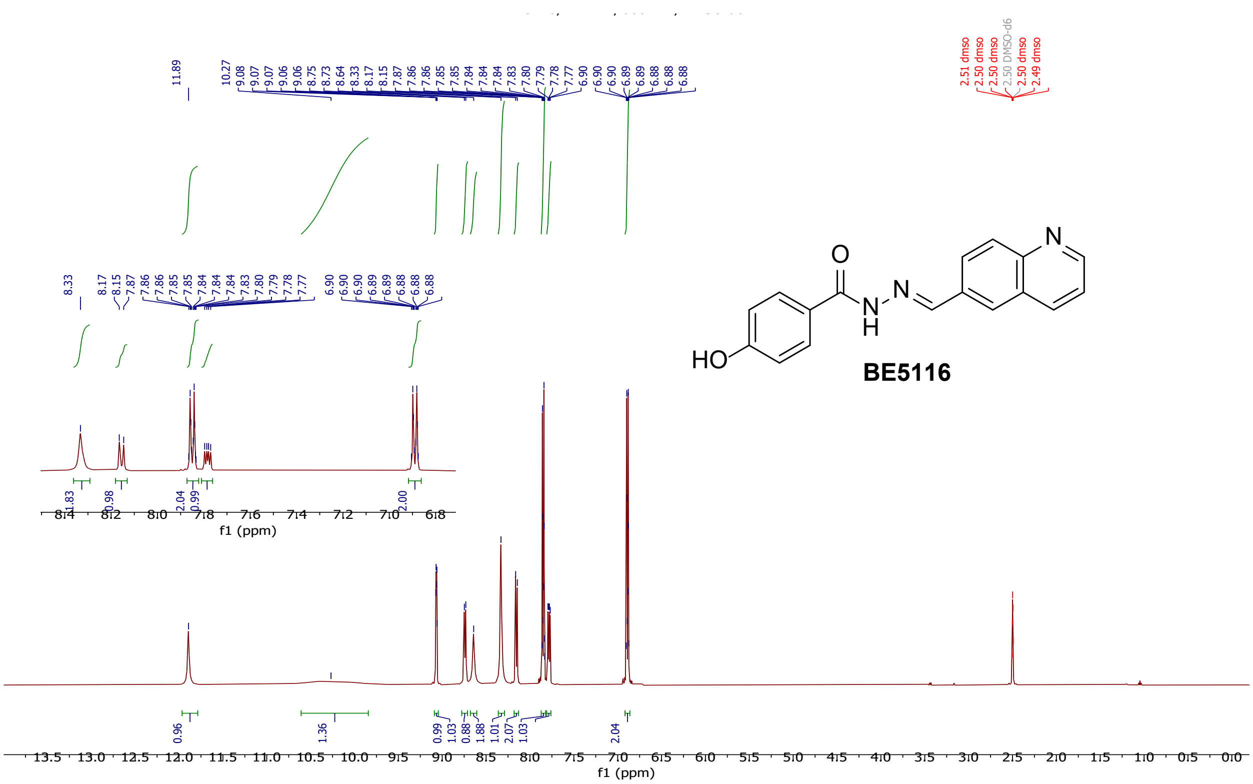

Figure S7. ^{1}H NMR spectra of compound BE5116

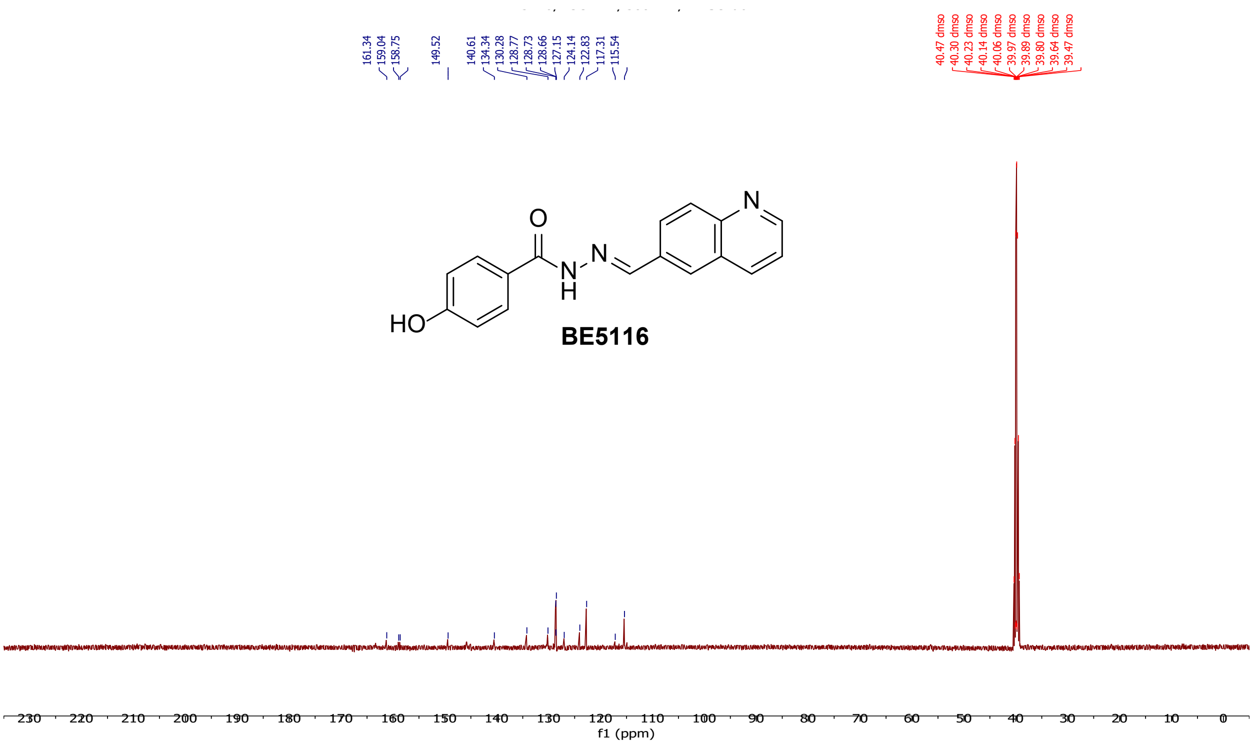

Figure S8. ^{13}C{^{1}H} NMR spectra of compound BE5116

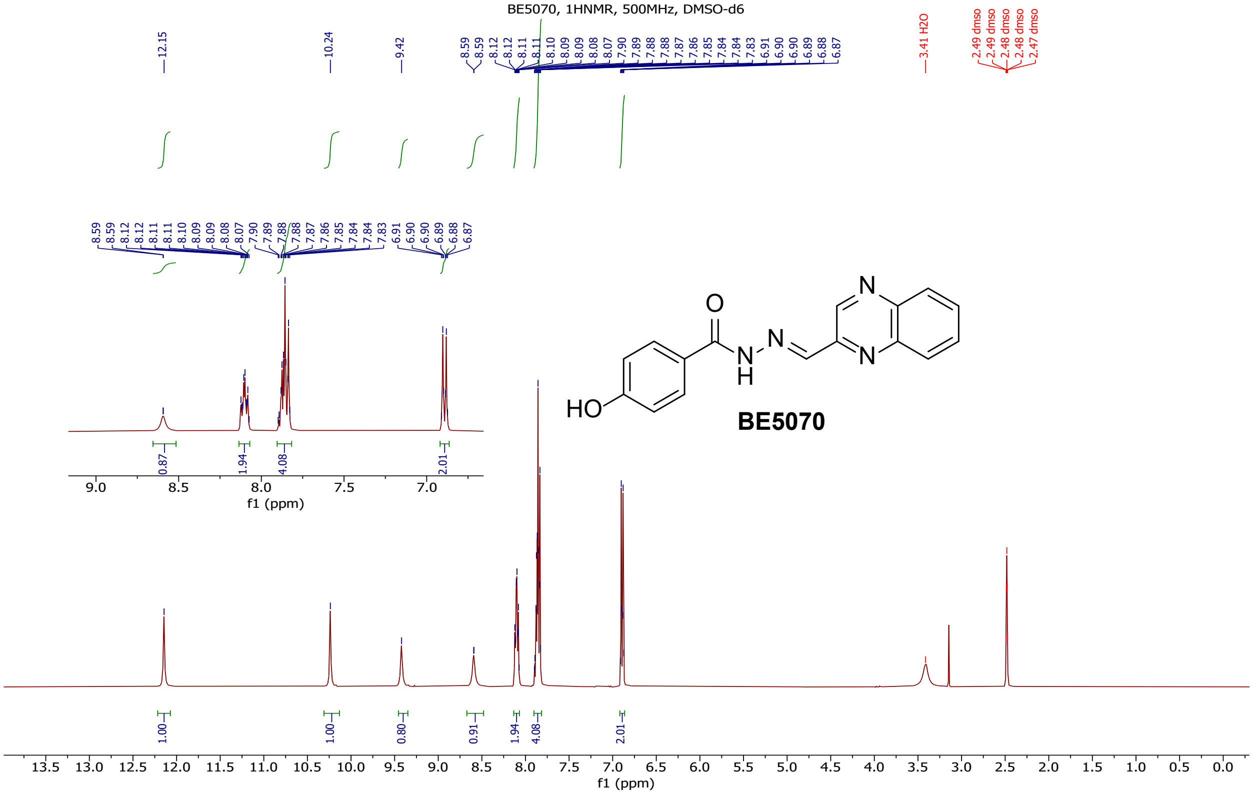

Figure S9. ^{1}H NMR spectra of compound BE5070

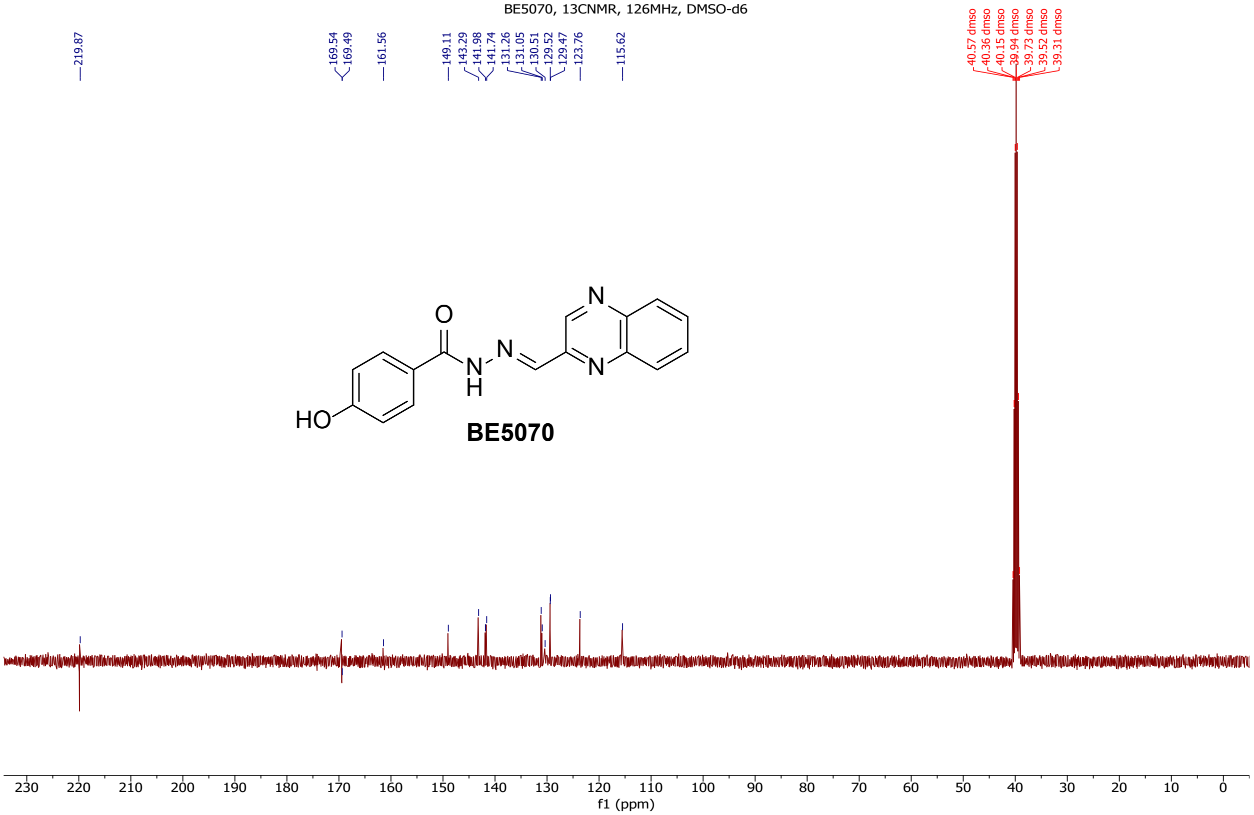

Figure S10. ^{13}C{^{1}H} NMR spectra of compound BE5070

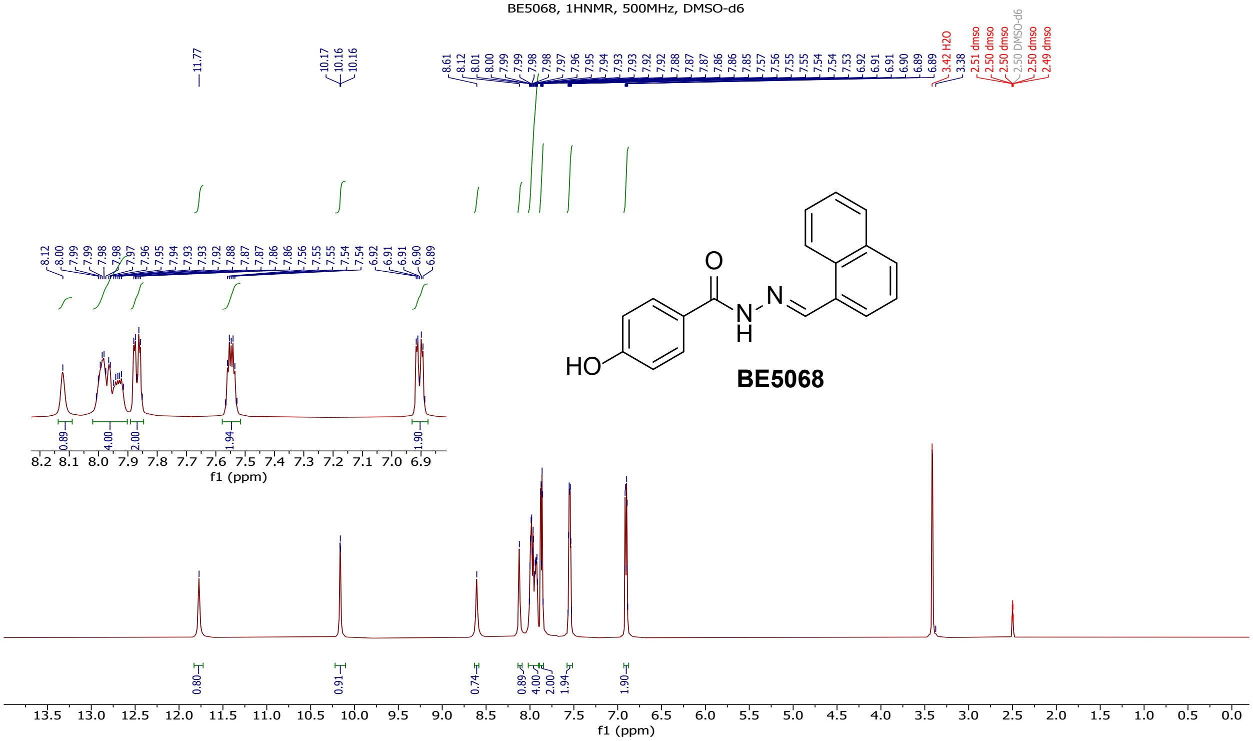

Figure S11. ^{1}H NMR spectra of compound BE5068

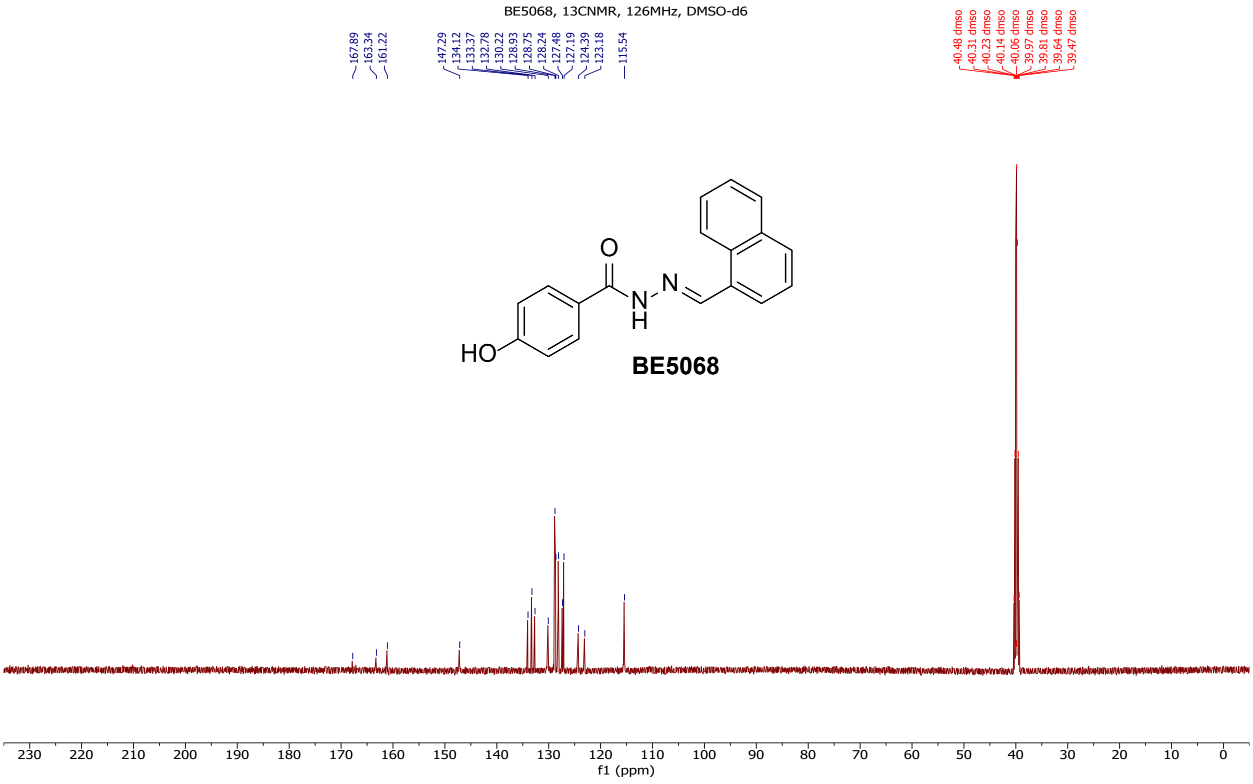

Figure S12. ^{13}C{^{1}H} NMR spectra of compound BE5068

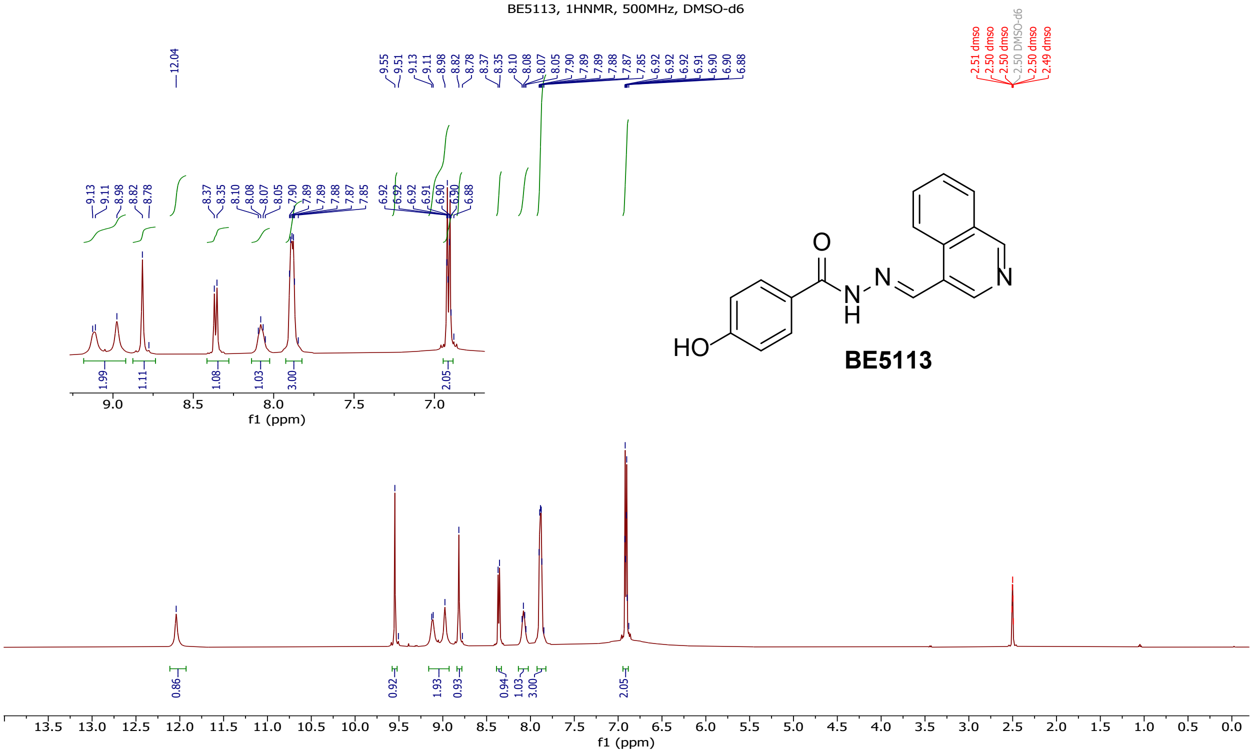

Figure S13. ^{1}H NMR spectra of compound BE5113

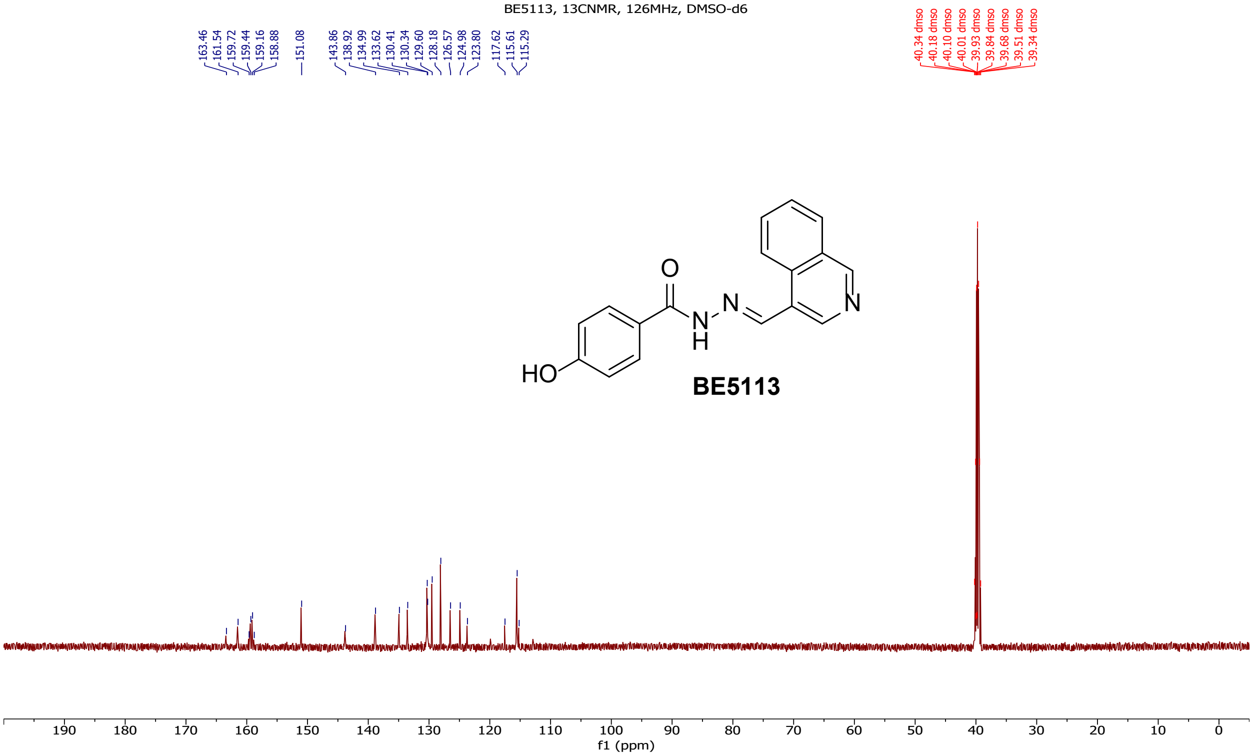

Figure S14. $^{13}C\{^{1}H\}$ NMR spectra of compound BE5113

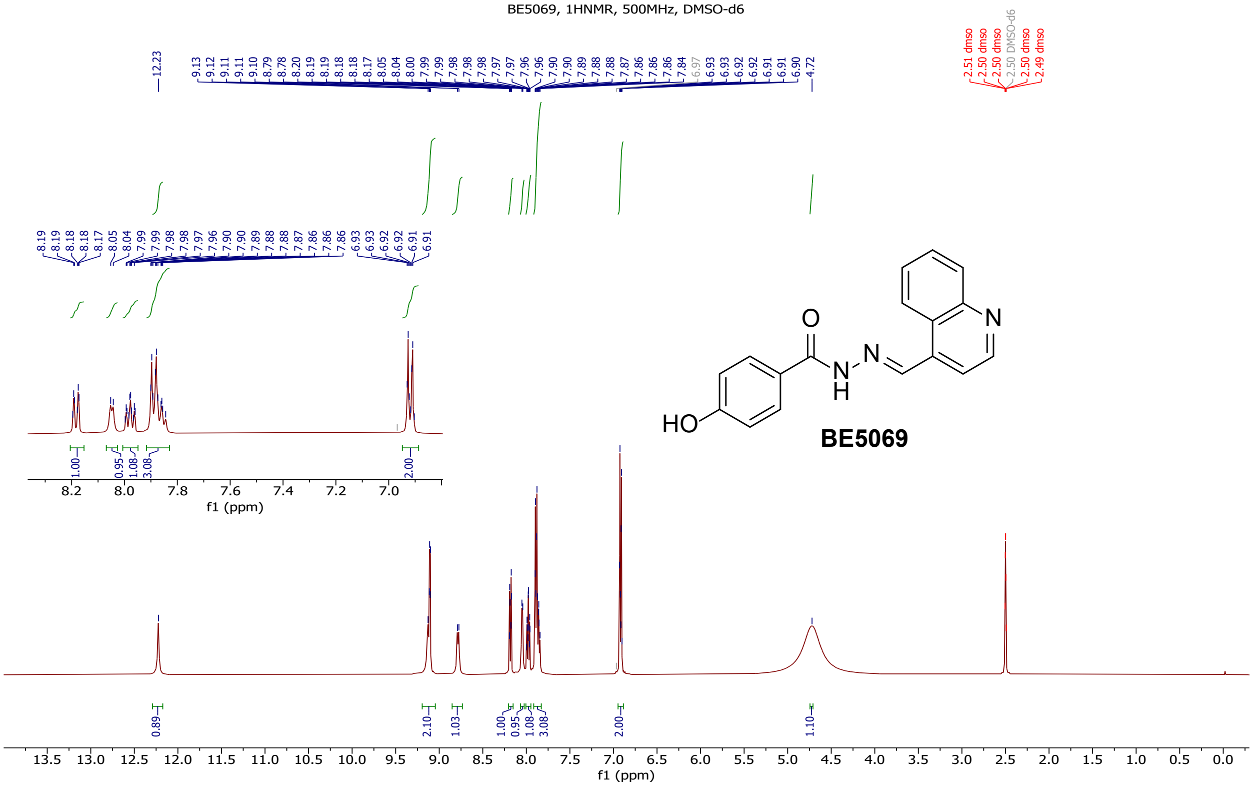

Figure S15. ^{1}H NMR spectra of compound BE5069

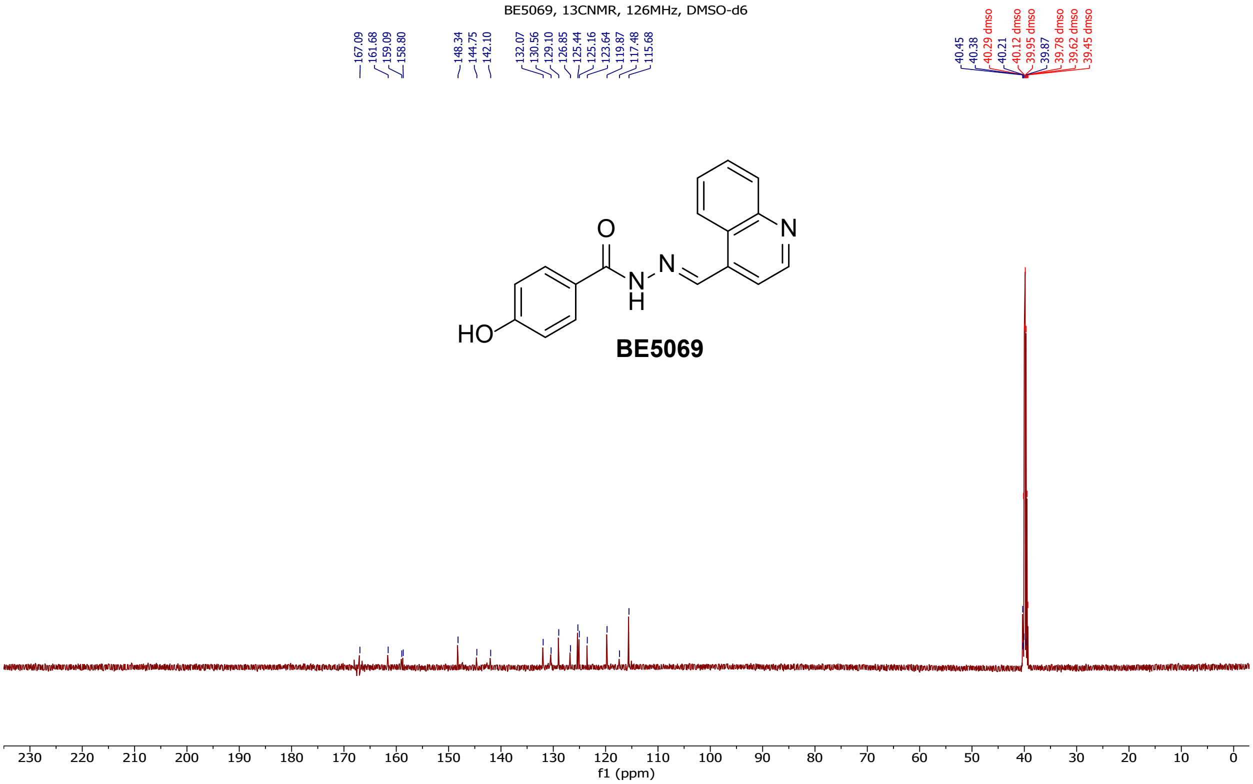

Figure S16. 13C{^{1}H} NMR spectra of compound BE5069

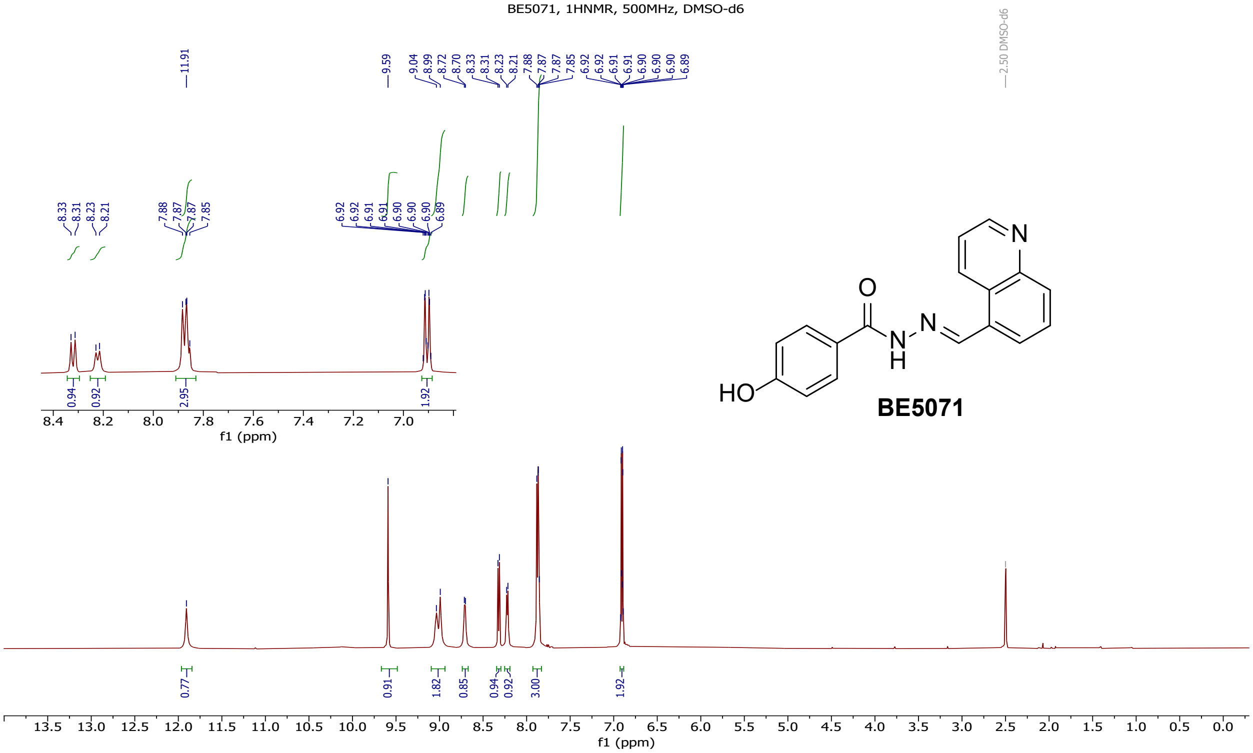

Figure S17. ^{1}H NMR spectra of compound BE5071

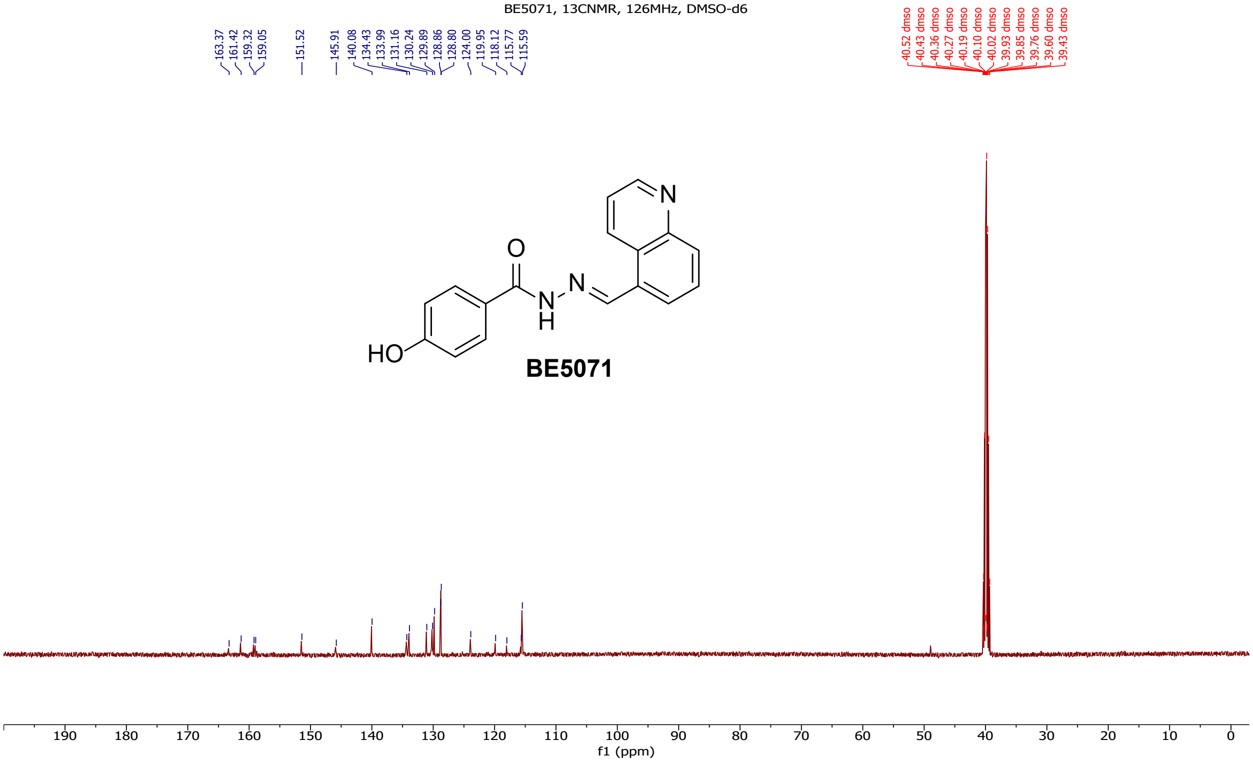

Figure S18. ^{13}C{^{1}H} NMR spectra of compound BE5071

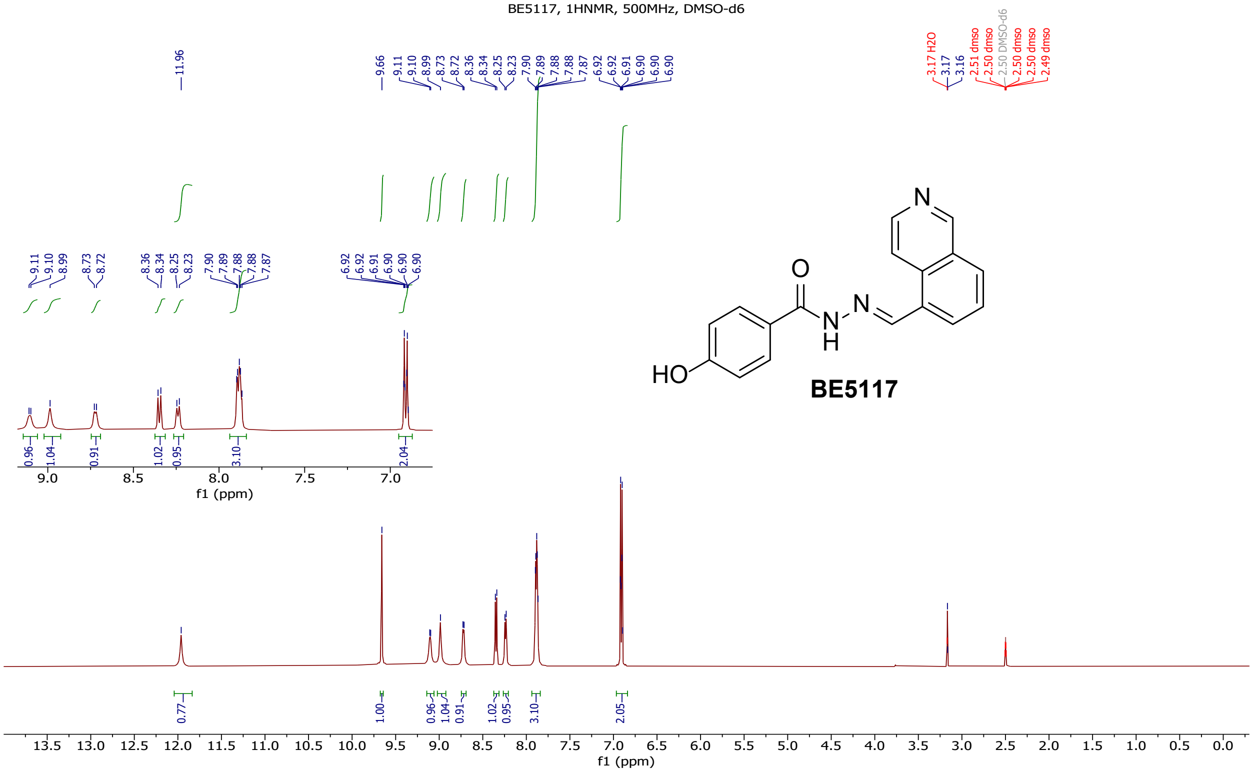

Figure S19. ^{1}H NMR spectra of compound BE5117

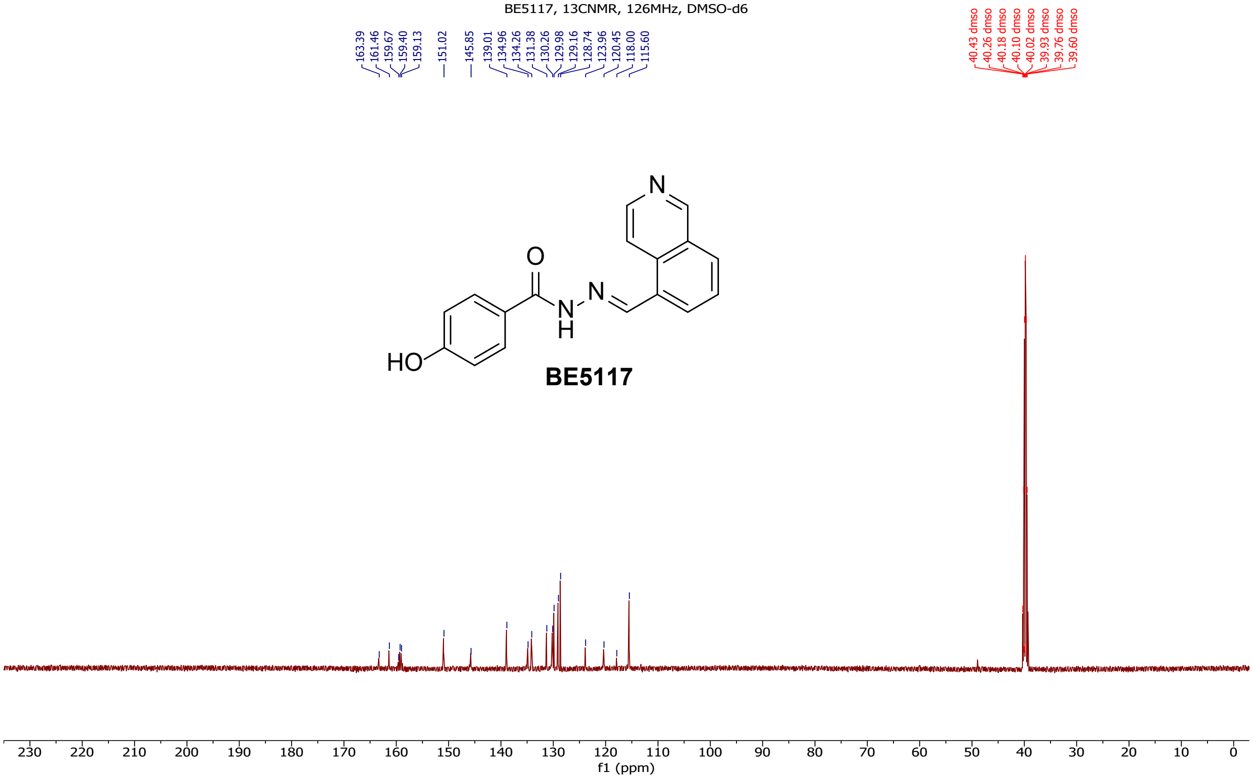

Figure S20. ^{13}C{^{1}H} NMR spectra of compound BE5117

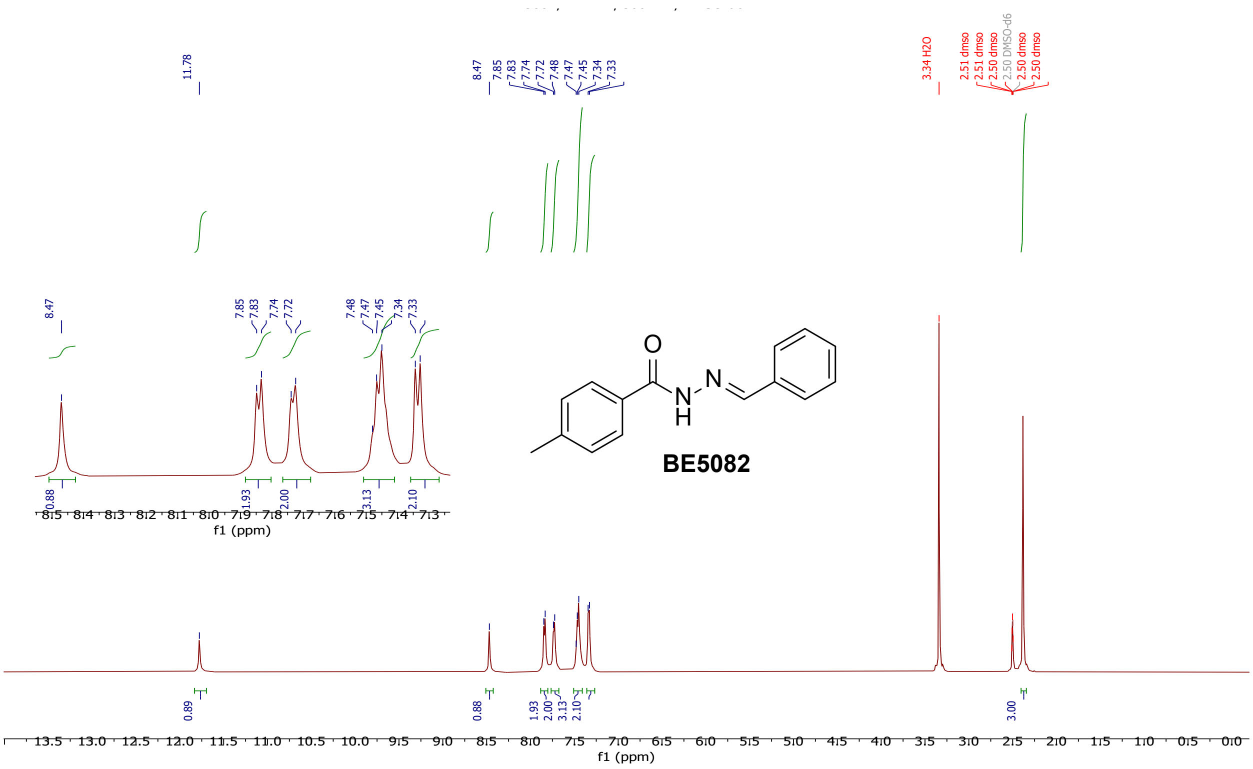

Figure S21. ^{1}H NMR spectra of compound BE5082

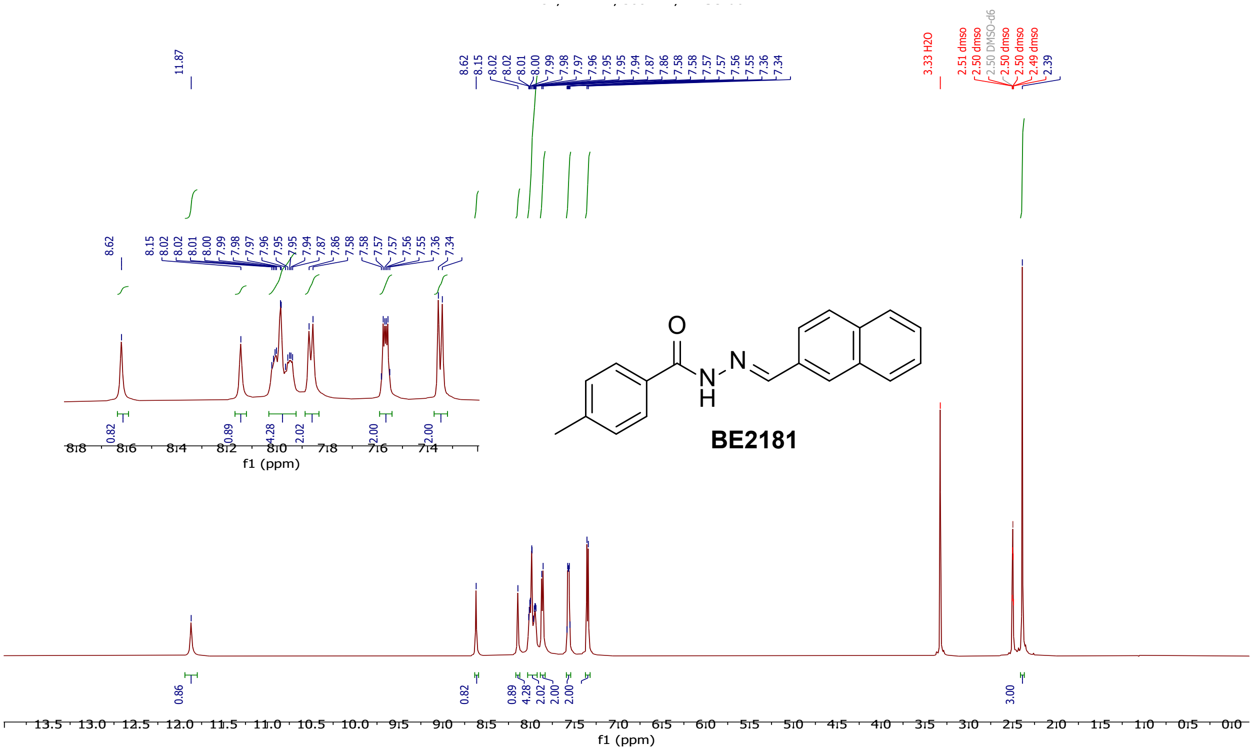

Figure S22. ^{1}H NMR spectra of compound BE2181

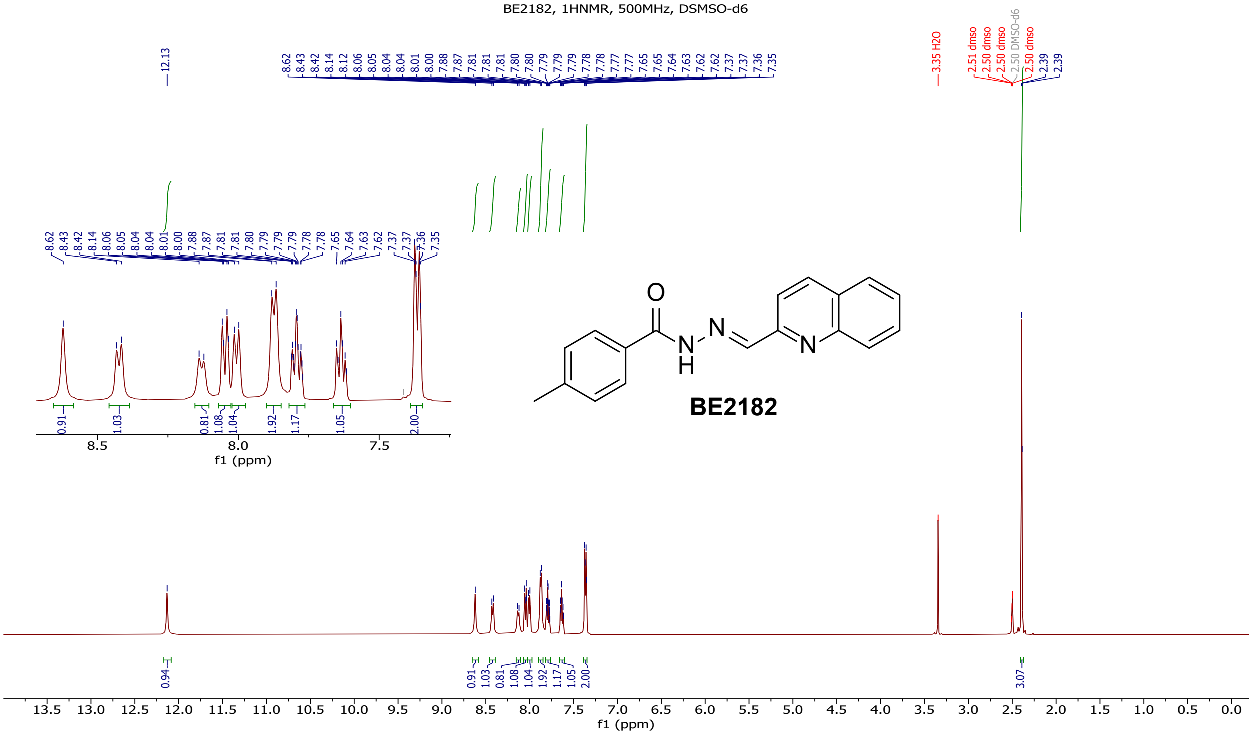

Figure S23. ^{1}H NMR spectra of compound BE2182

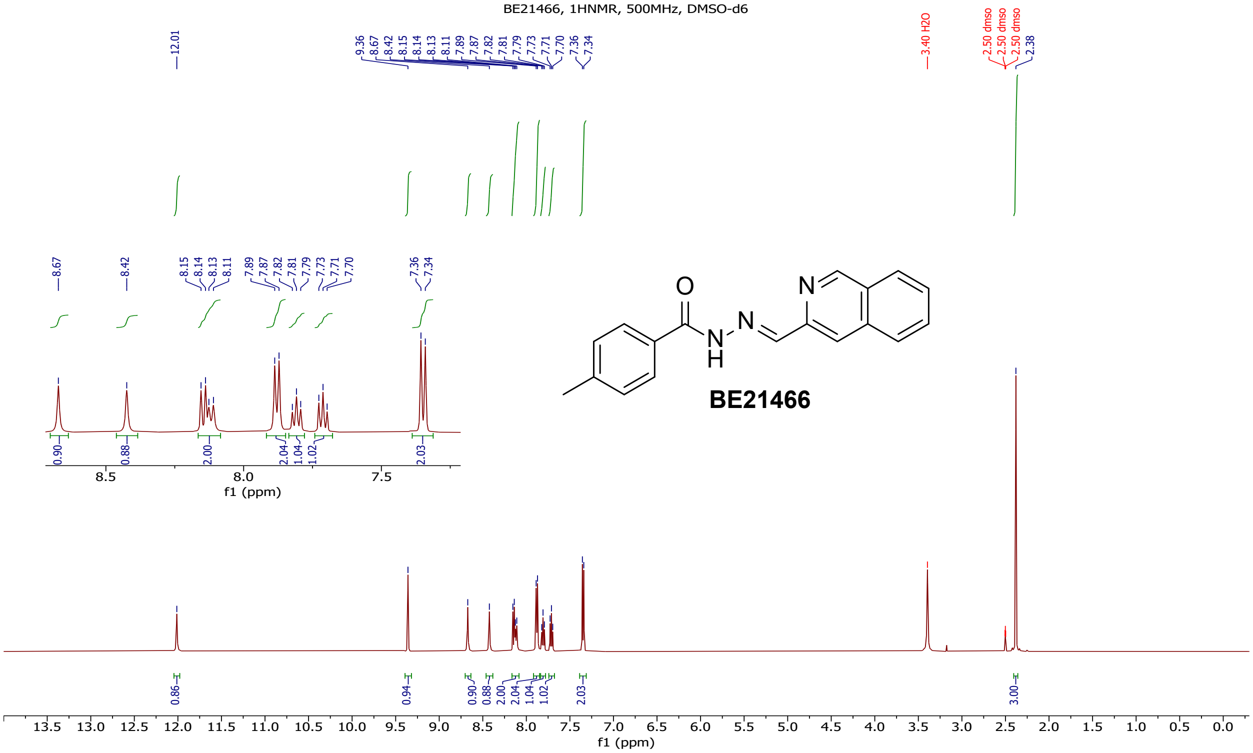

Figure S24. ^{1}H NMR spectra of compound BE21466

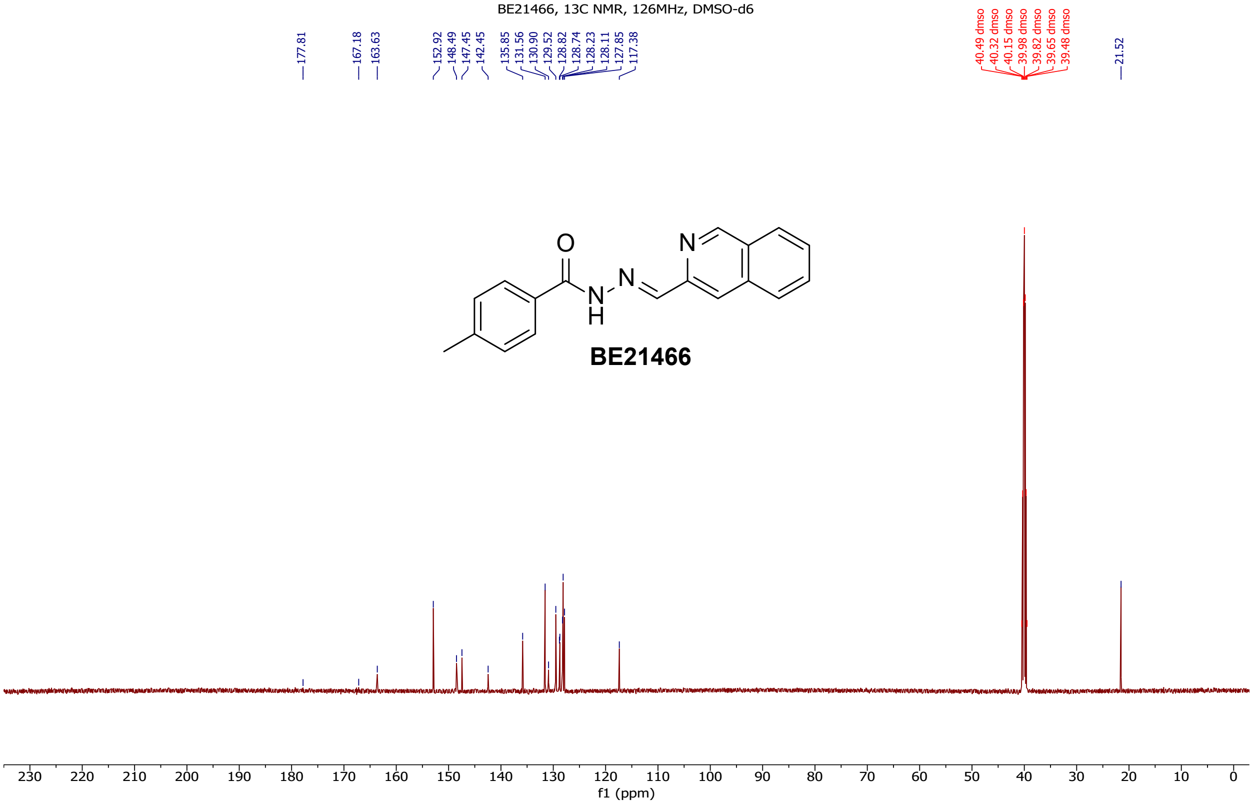

Figure S25. ^{13}C{^{1}H} NMR spectra of compound BE21466

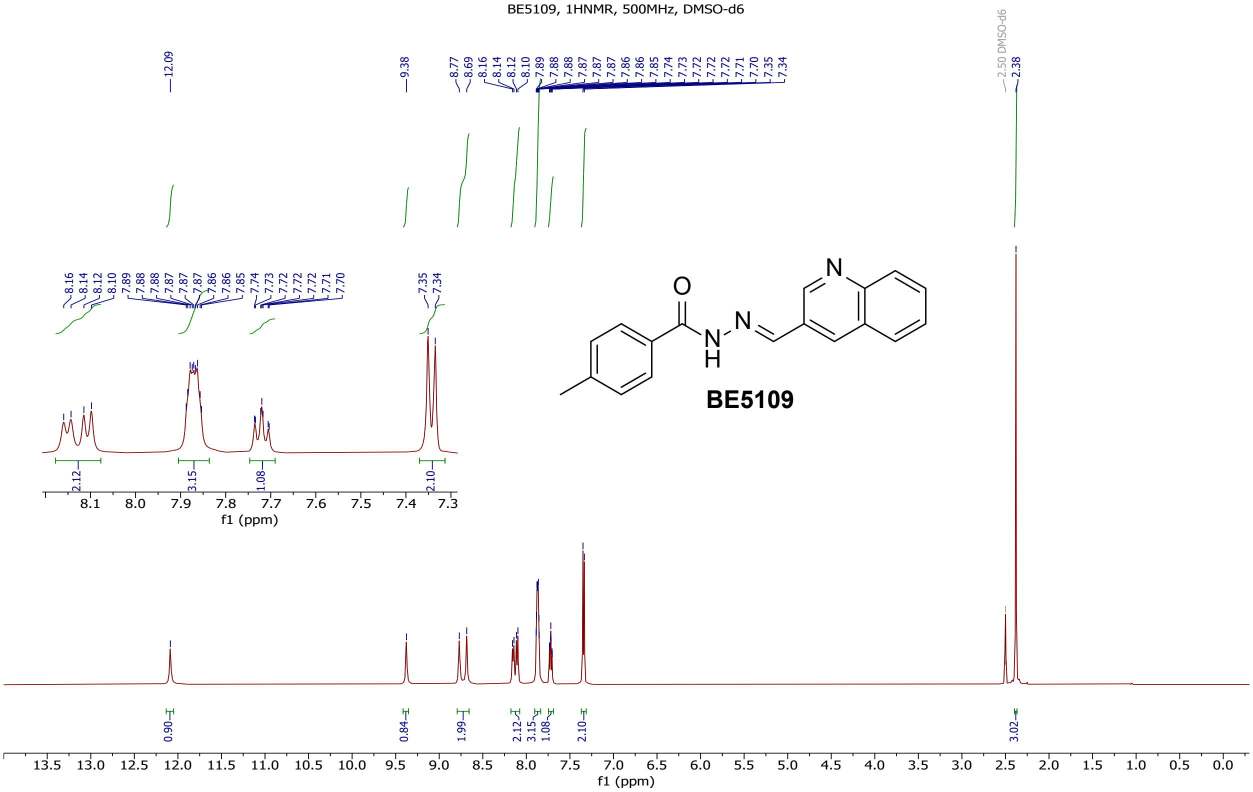

Figure S26. ^{1}H **NMR spectra of compound BE5109**

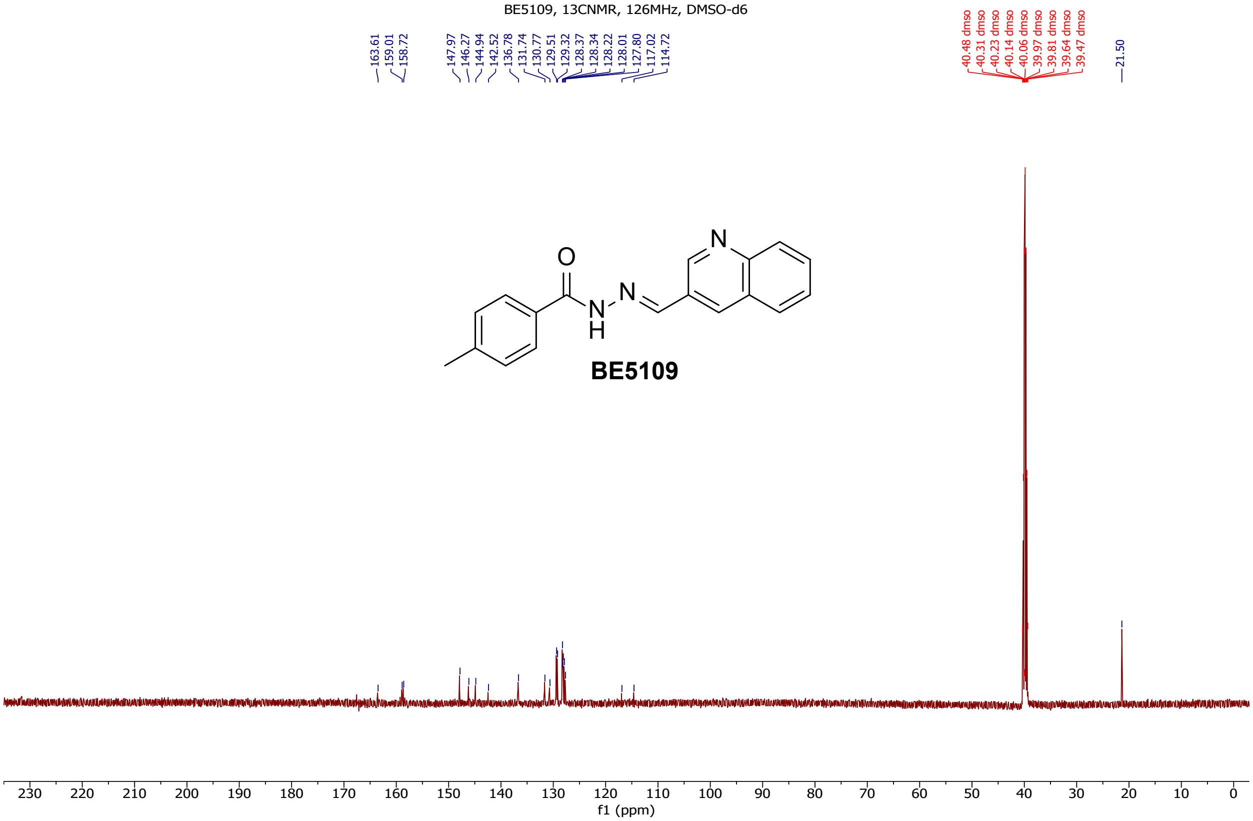

Figure S27. $^{13}C\{^{1}H\}$ **NMR spectra of compound BE5109**

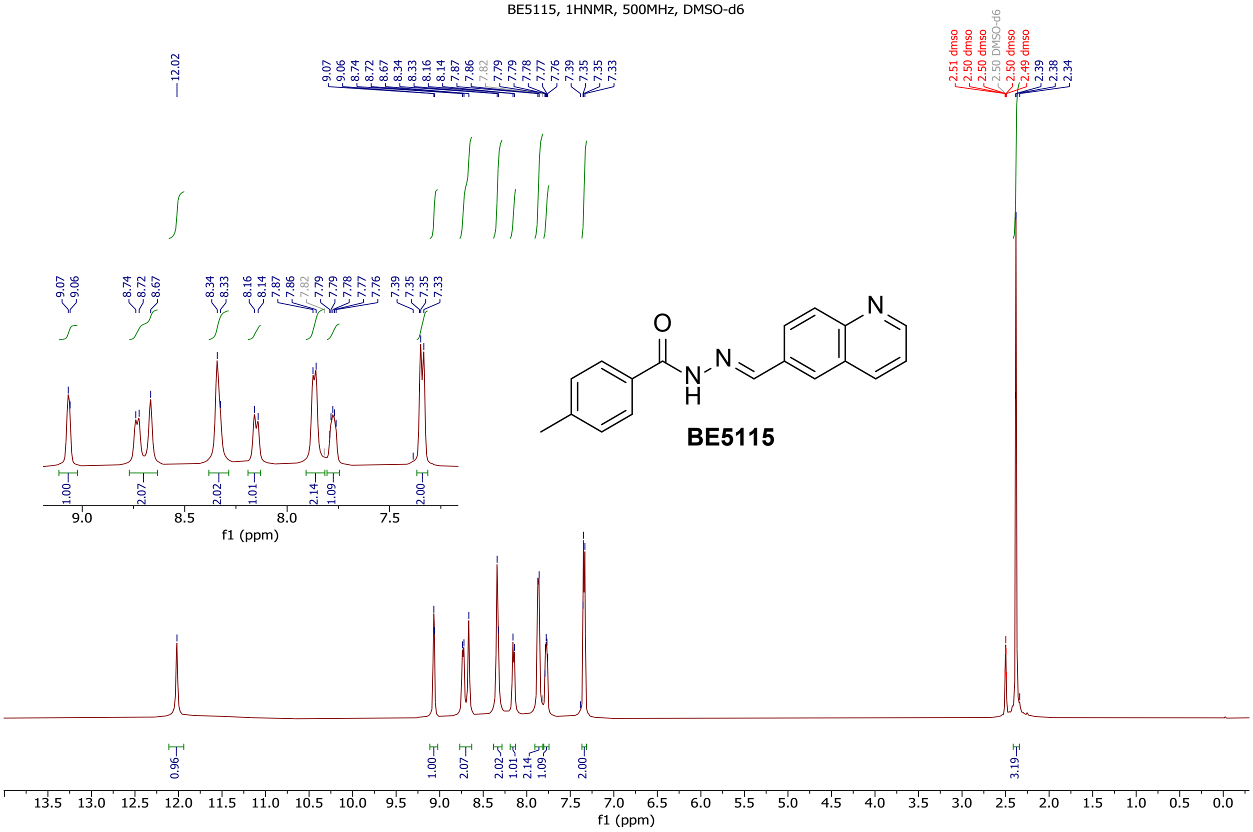

Figure S28. ^{1}H NMR spectra of compound BE5115

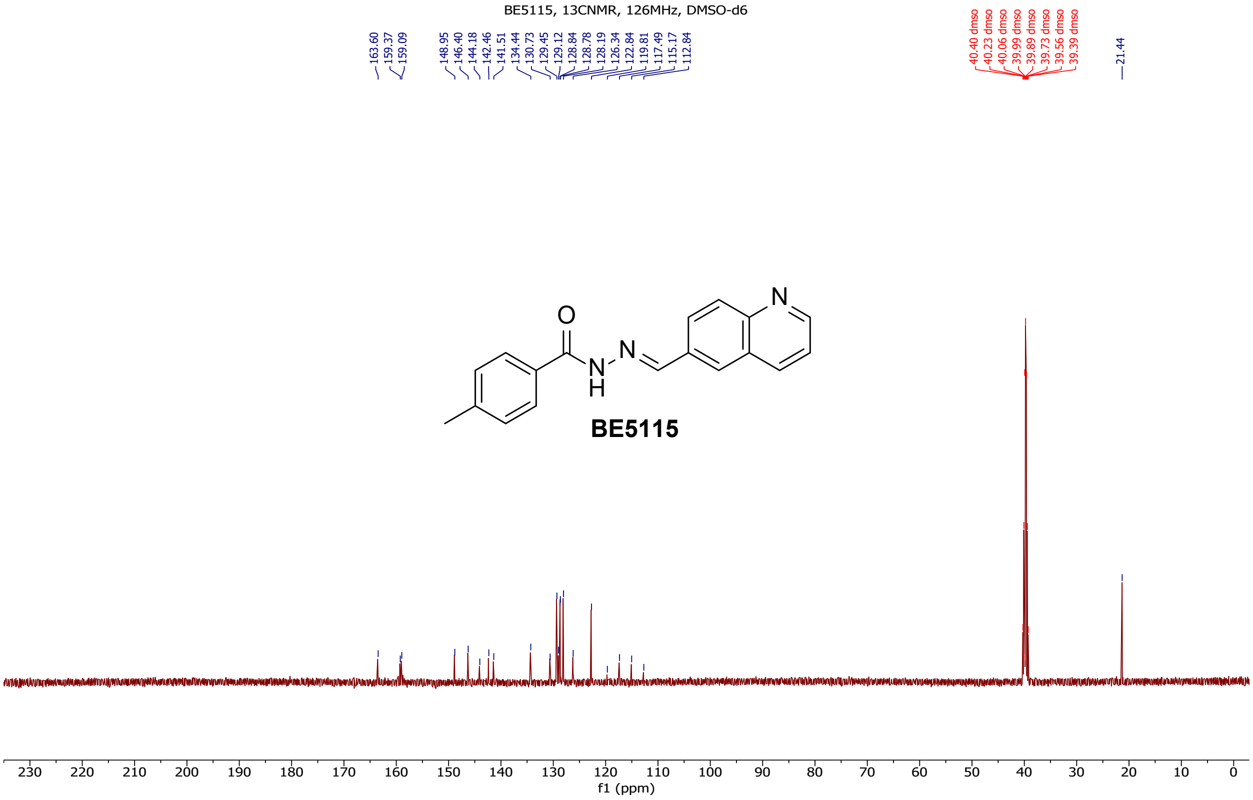

Figure S29. ^{13}C{^{1}H} NMR spectra of compound BE5115

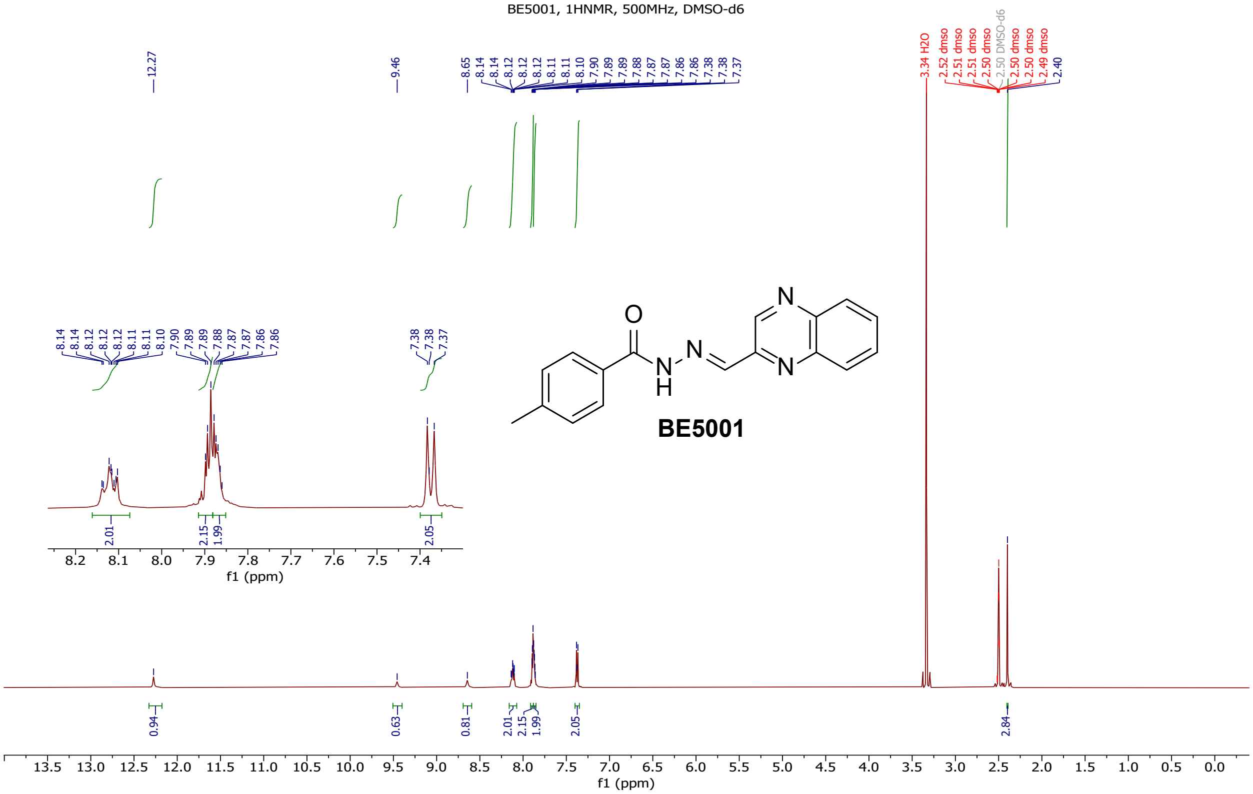

Figure S30. ^{1}**H NMR spectra of compound BE5001**

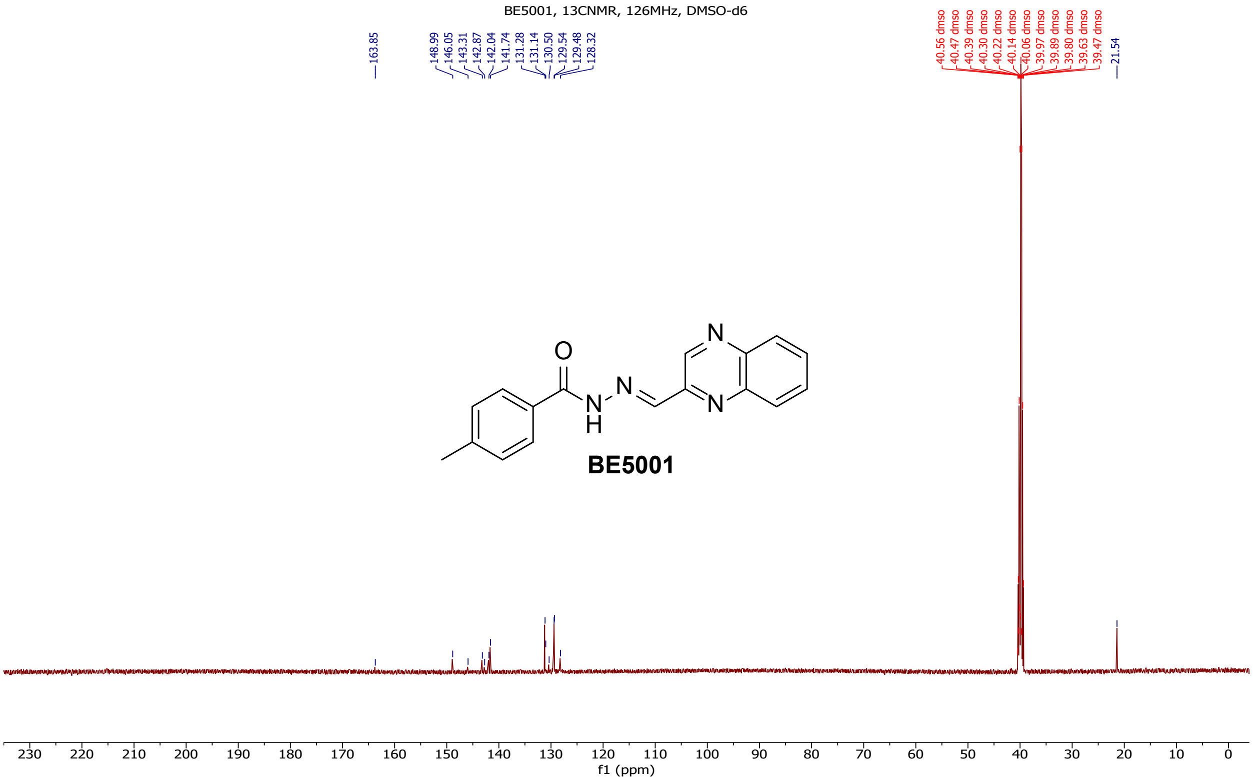

Figure S31. ^{13}**C{**^{1}**H} NMR spectra of compound BE5001**

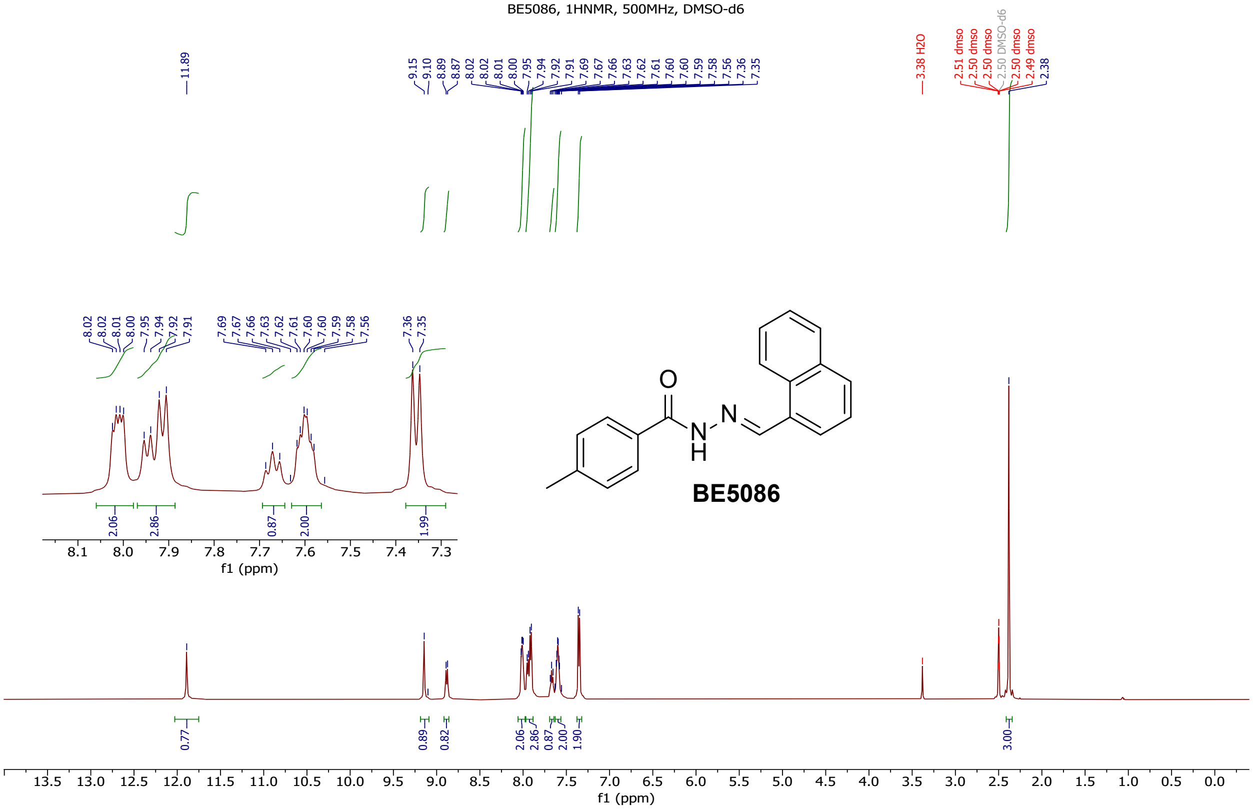

Figure S32. 1H NMR spectra of compound BE5086

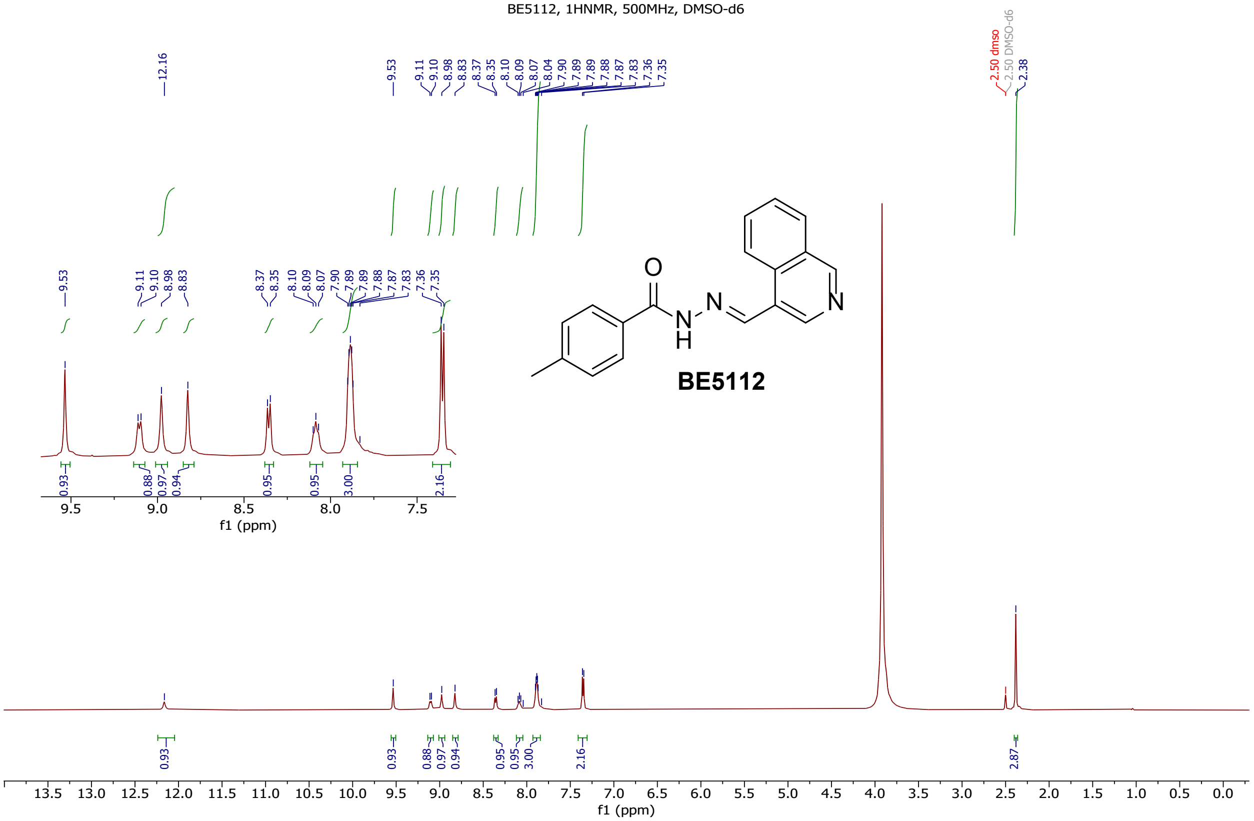

Figure S33. ¹**H NMR spectra of compound BE5112**

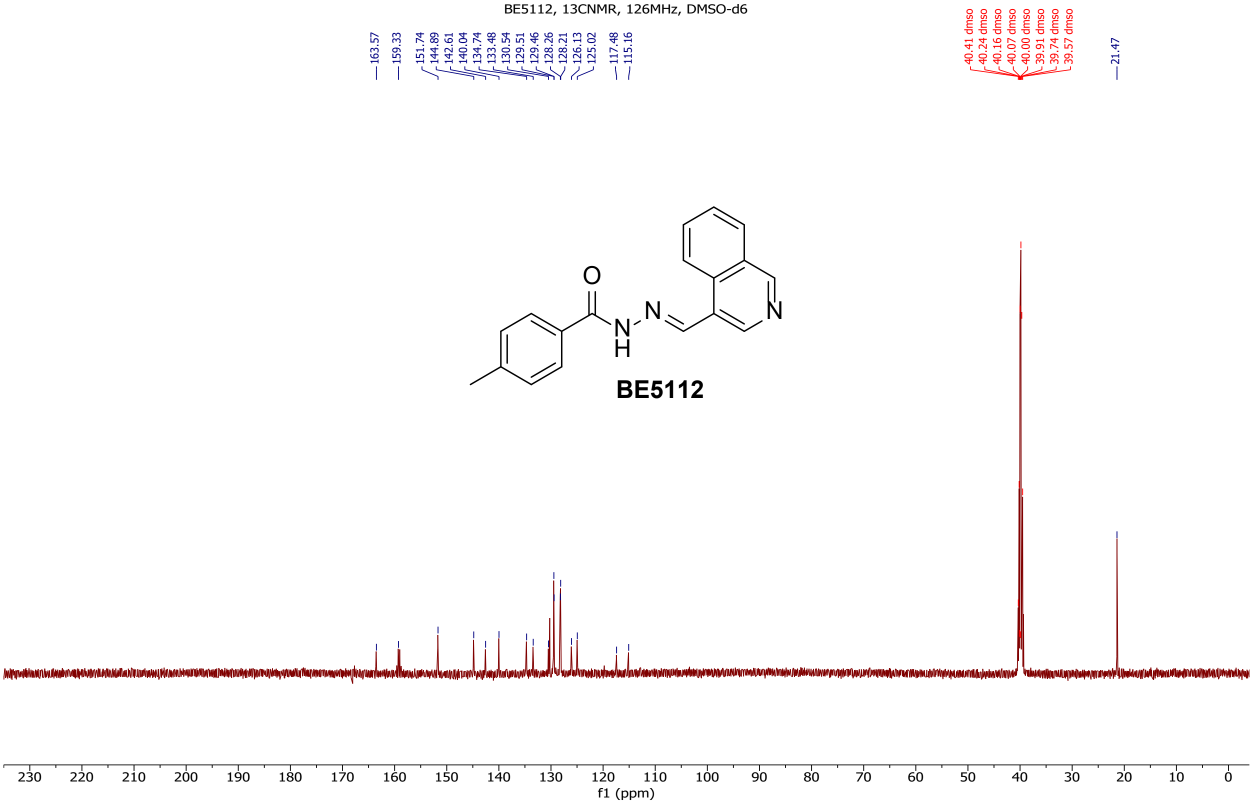

Figure S34. ¹³**C{**¹**H} NMR spectra of compound BE5112**

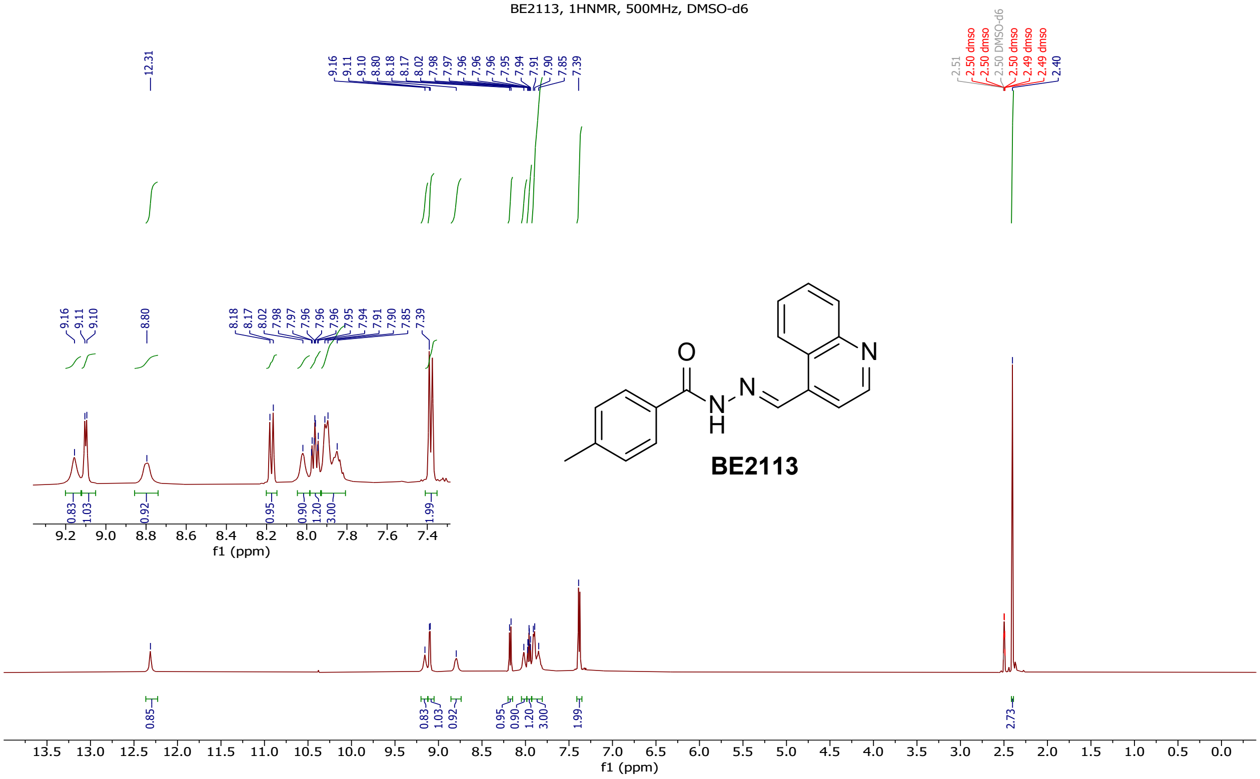

Figure S35. 1H NMR spectra of compound BE2113

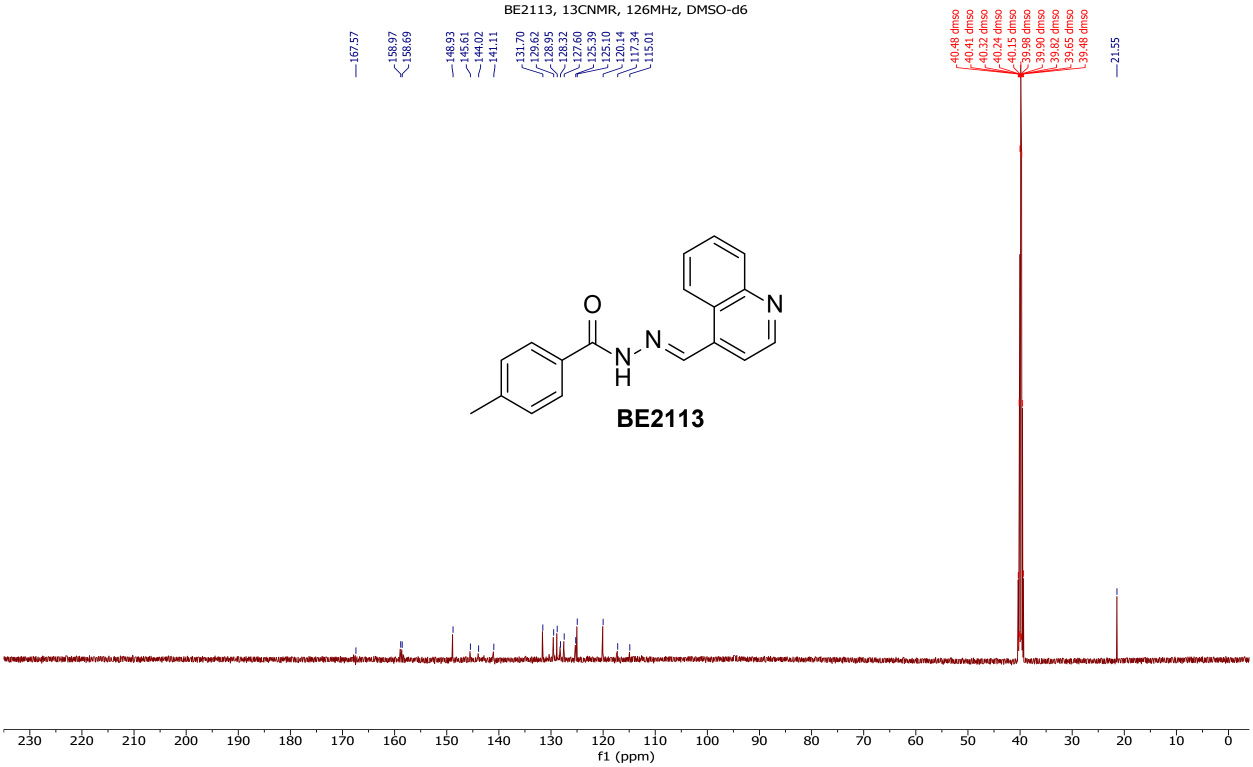

Figure S36. $^{13}C\{^{1}H\}$ NMR spectra of compound BE2113

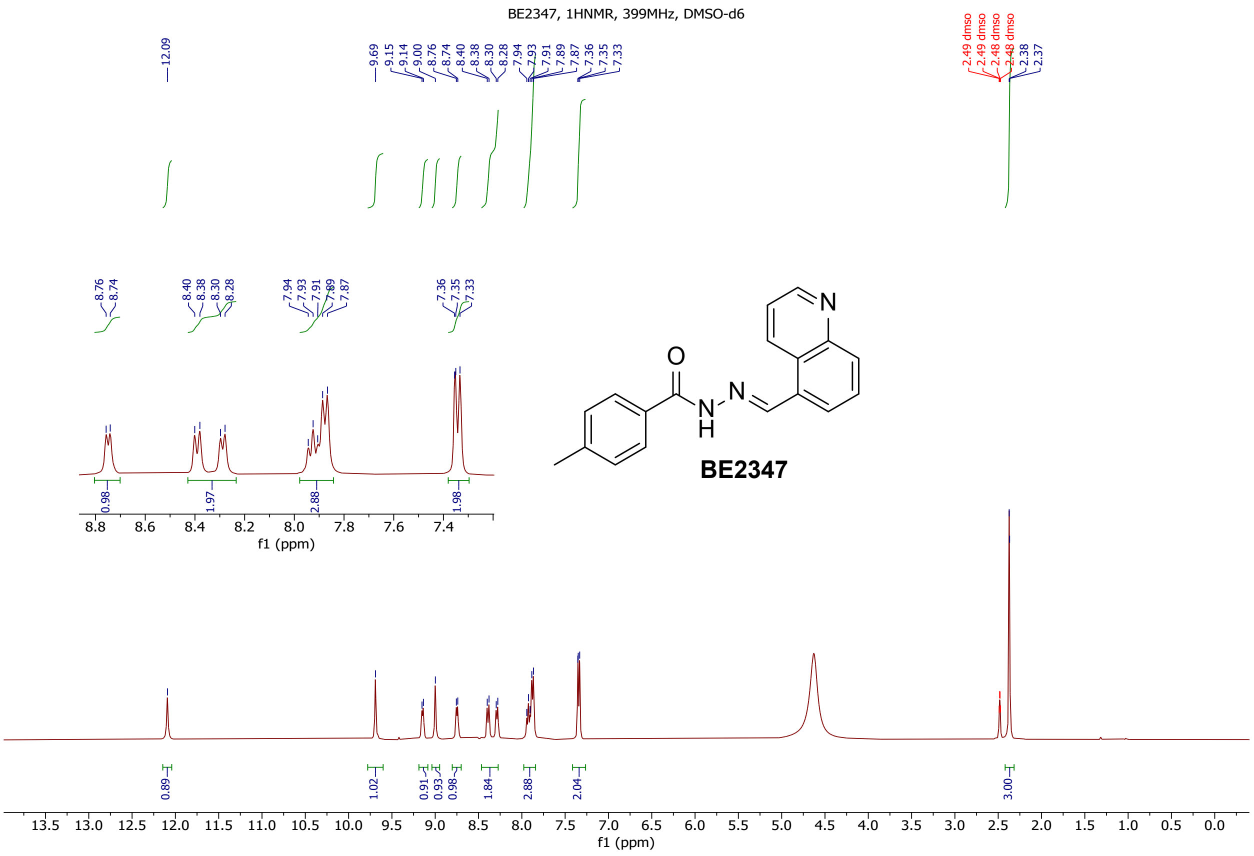

Figure S37. ¹H NMR spectra of compound BE2347

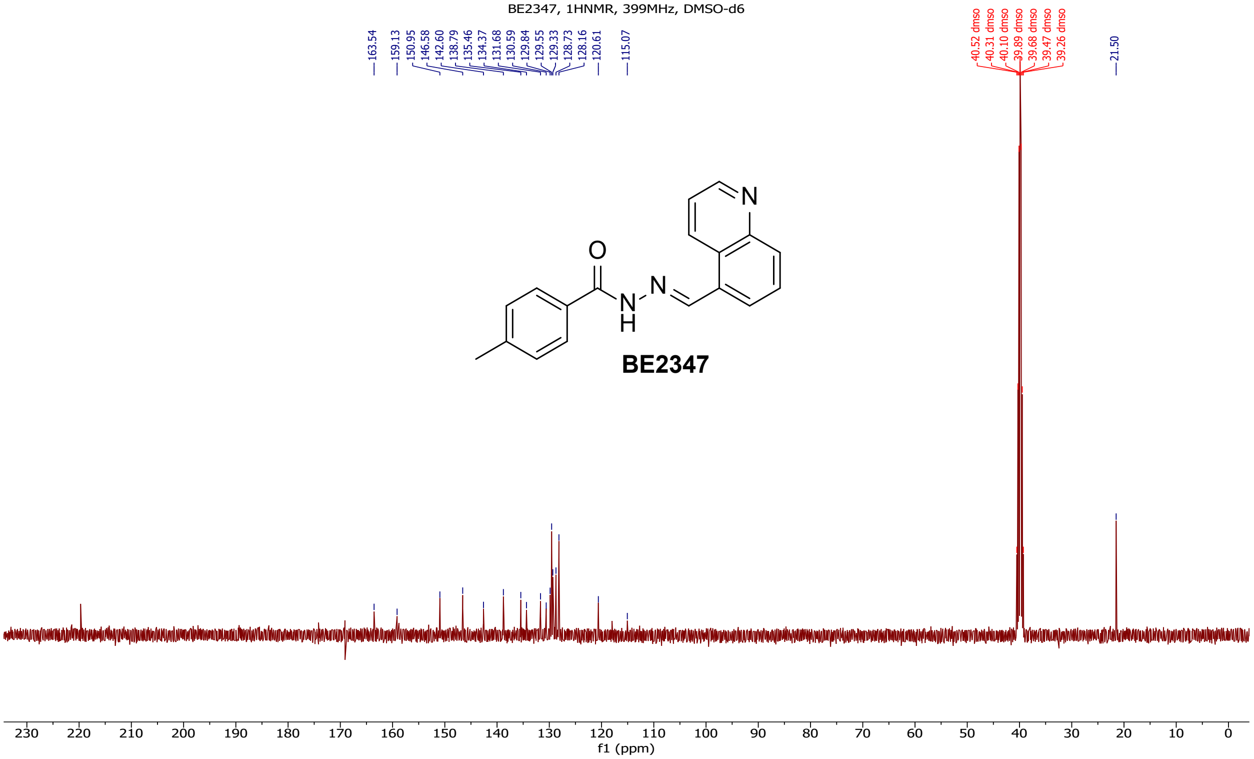

Figure S38. ¹H NMR spectra of compound BE2347

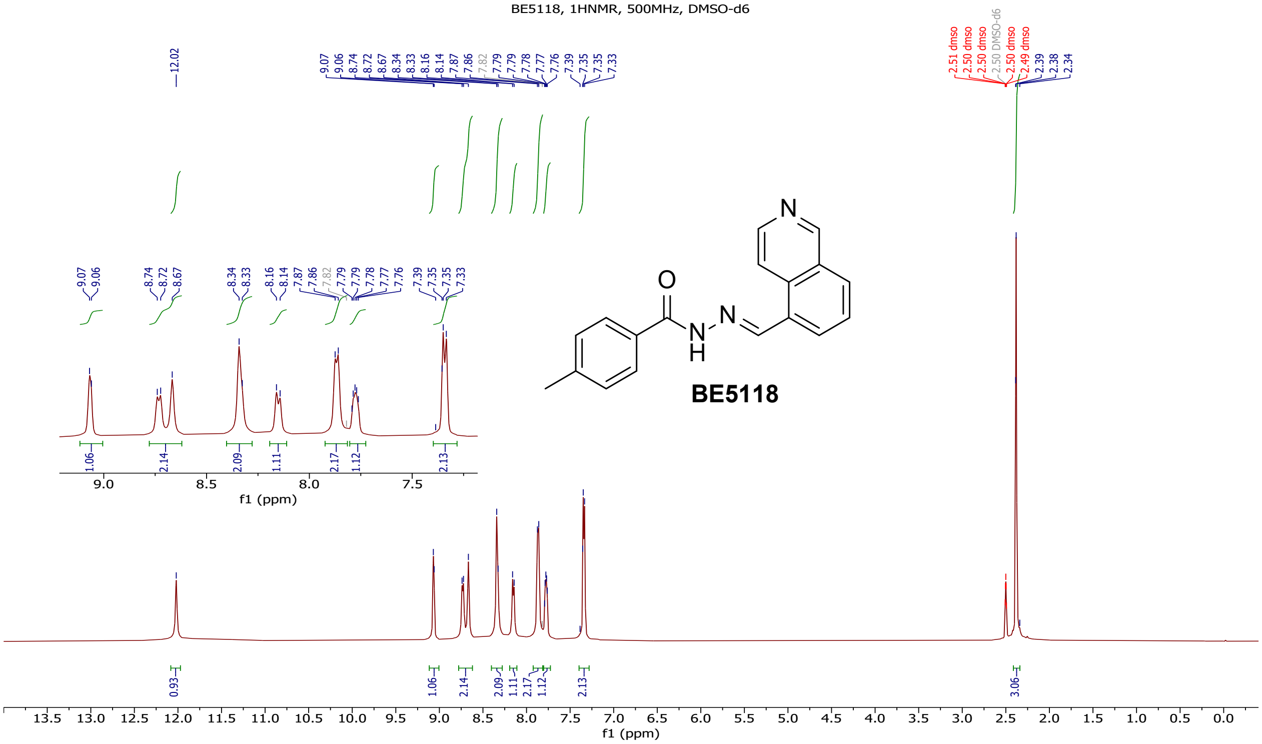

Figure S39. ^{1}H NMR spectra of compound BE5118

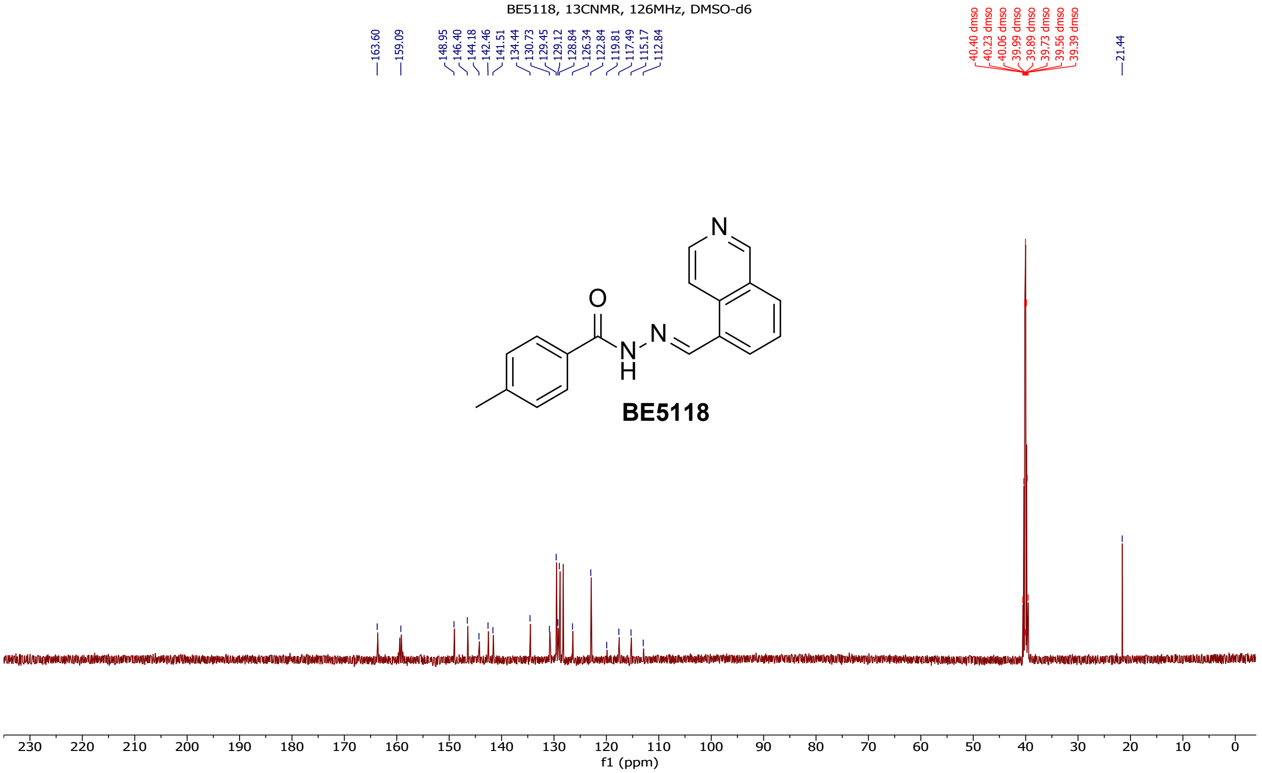

Figure S40. ^{13}C{^{1}H} NMR spectra of compound BE5118

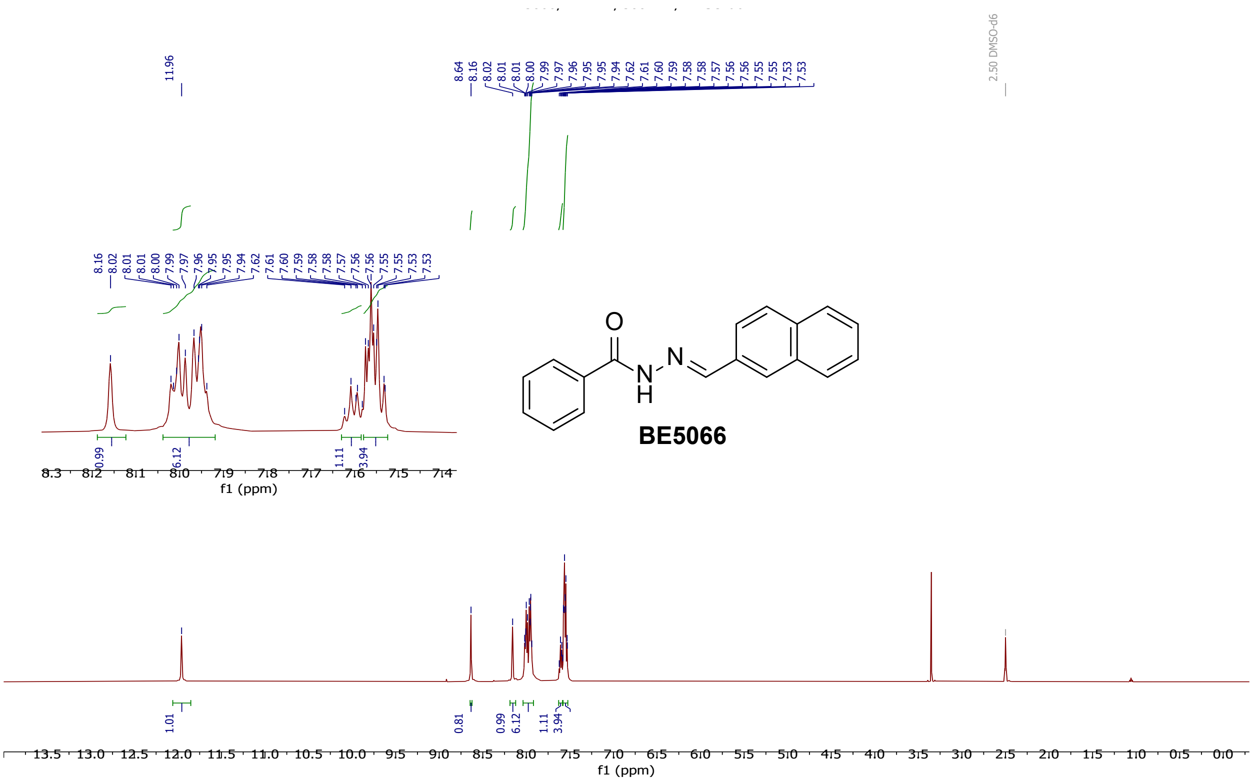

Figure S41. ^{1}H NMR spectra of compound BE5066

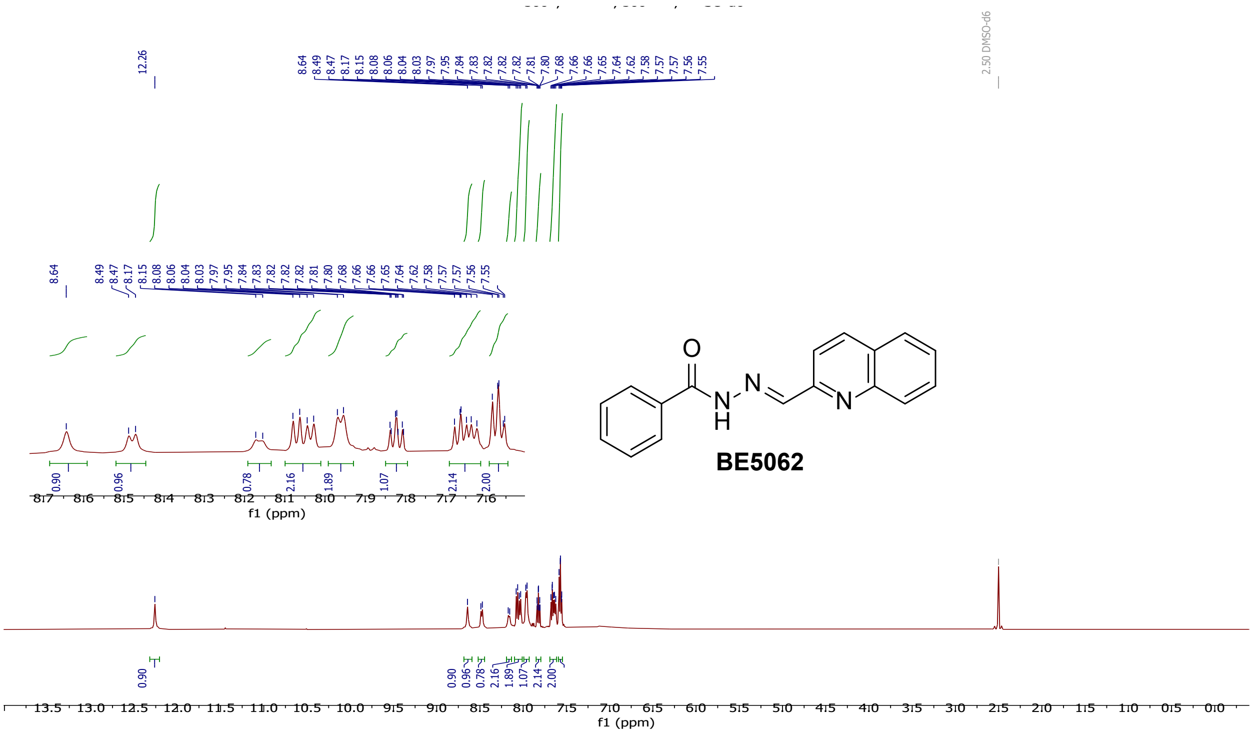

Figure S42. ^{1}H NMR spectra of compound BE5062

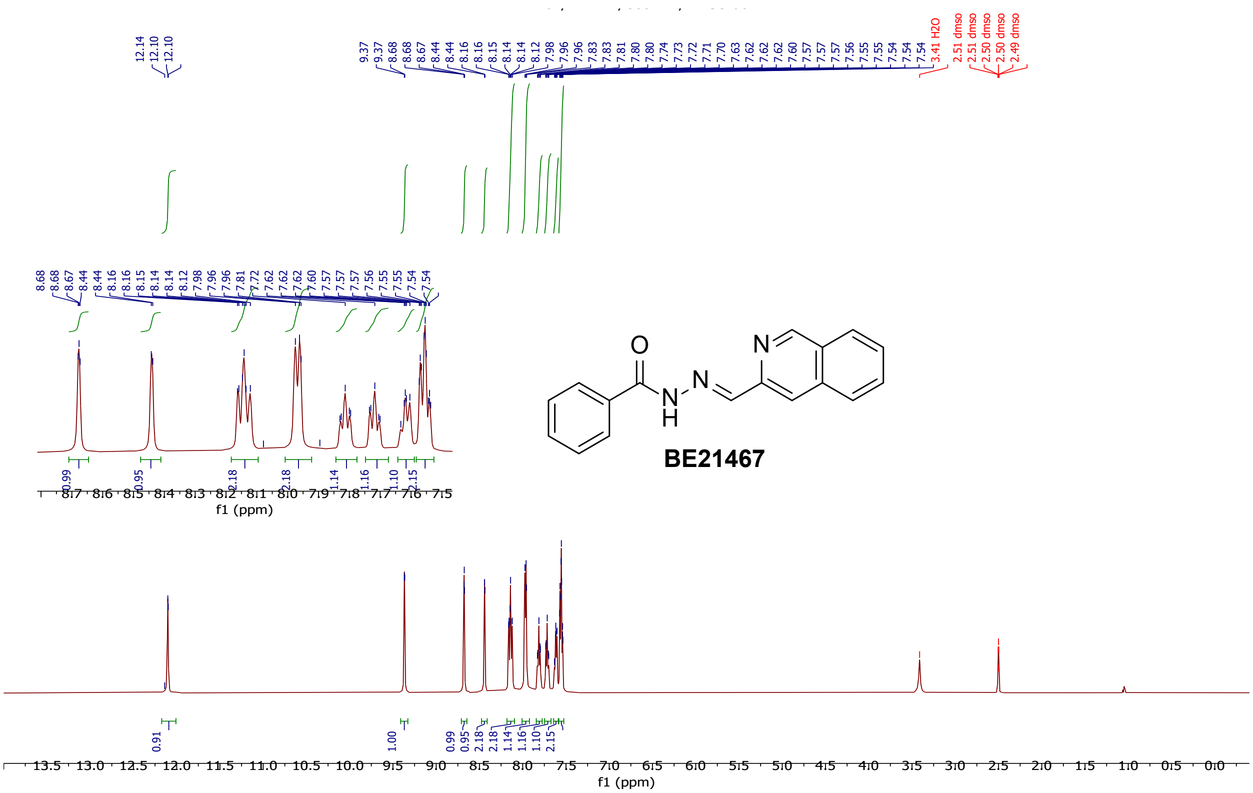

Figure S43. ^{1}H **NMR spectra of compound BE21467**

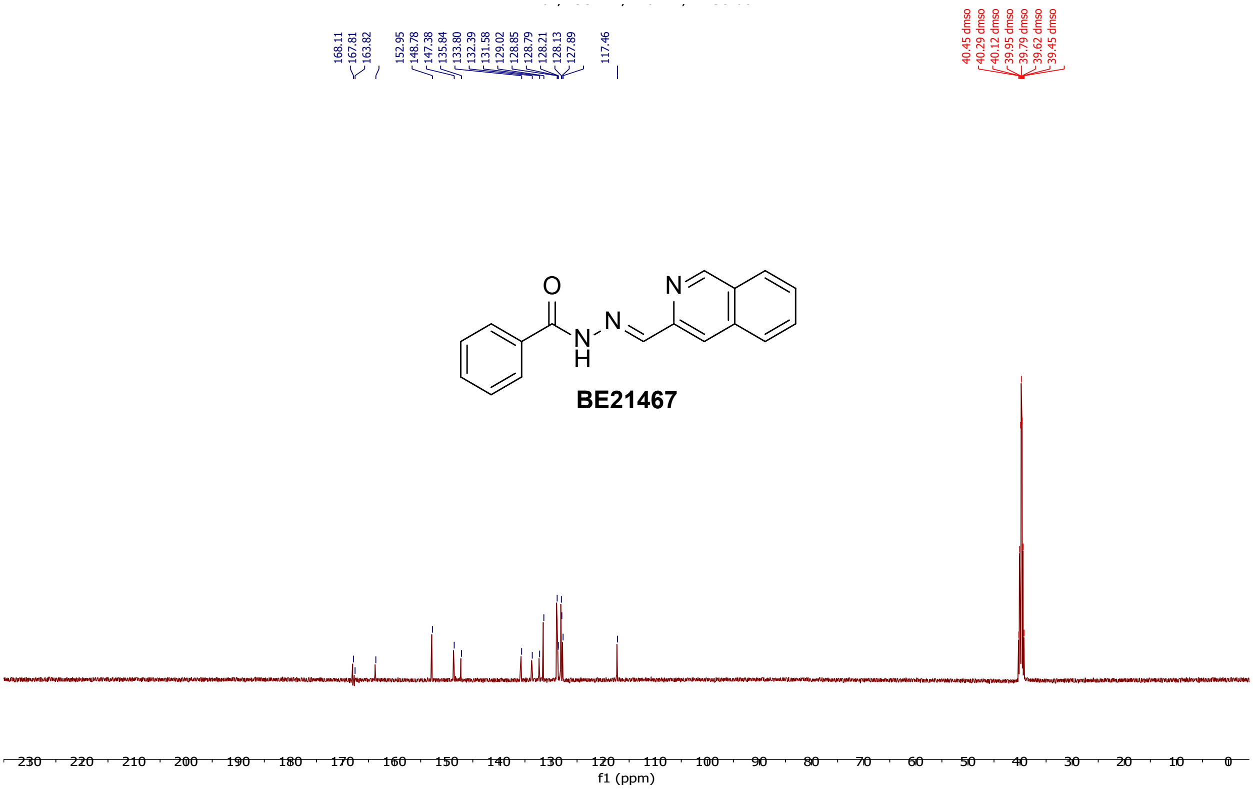

Figure S44. $^{13}C\{^{1}H\}$ **NMR spectra of compound BE21467**

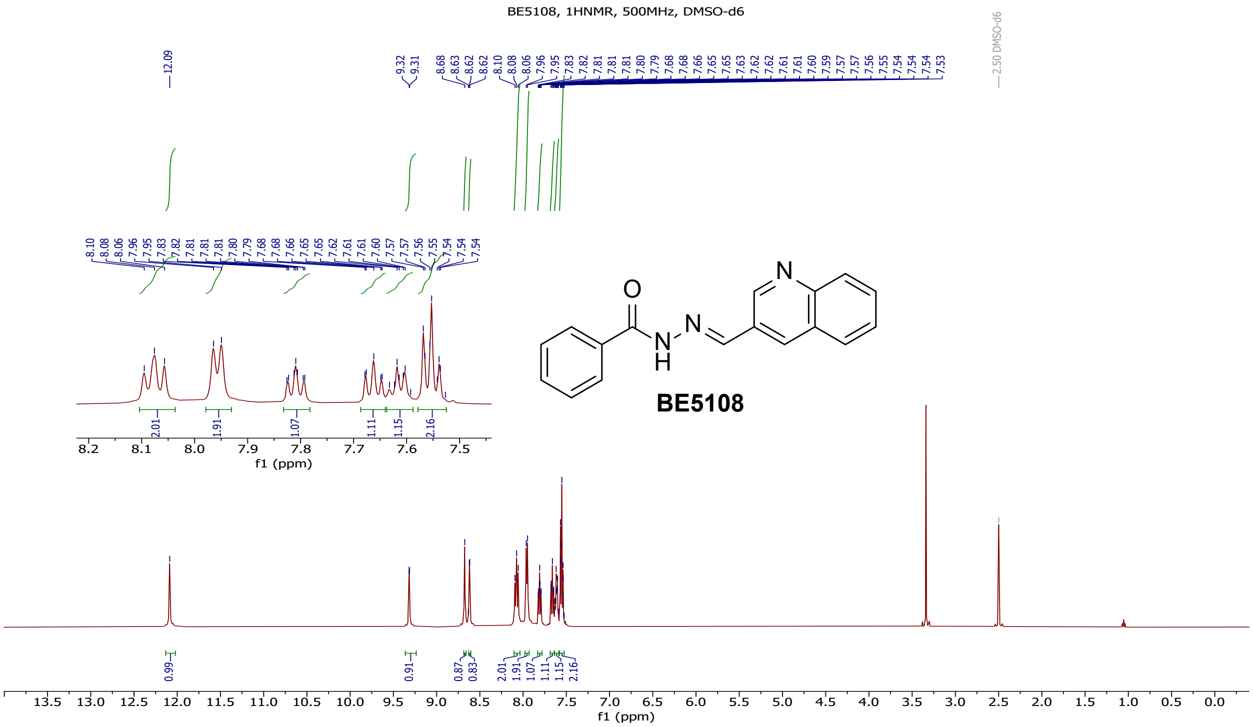

Figure S45. ^{1}H NMR spectra of compound BE5108

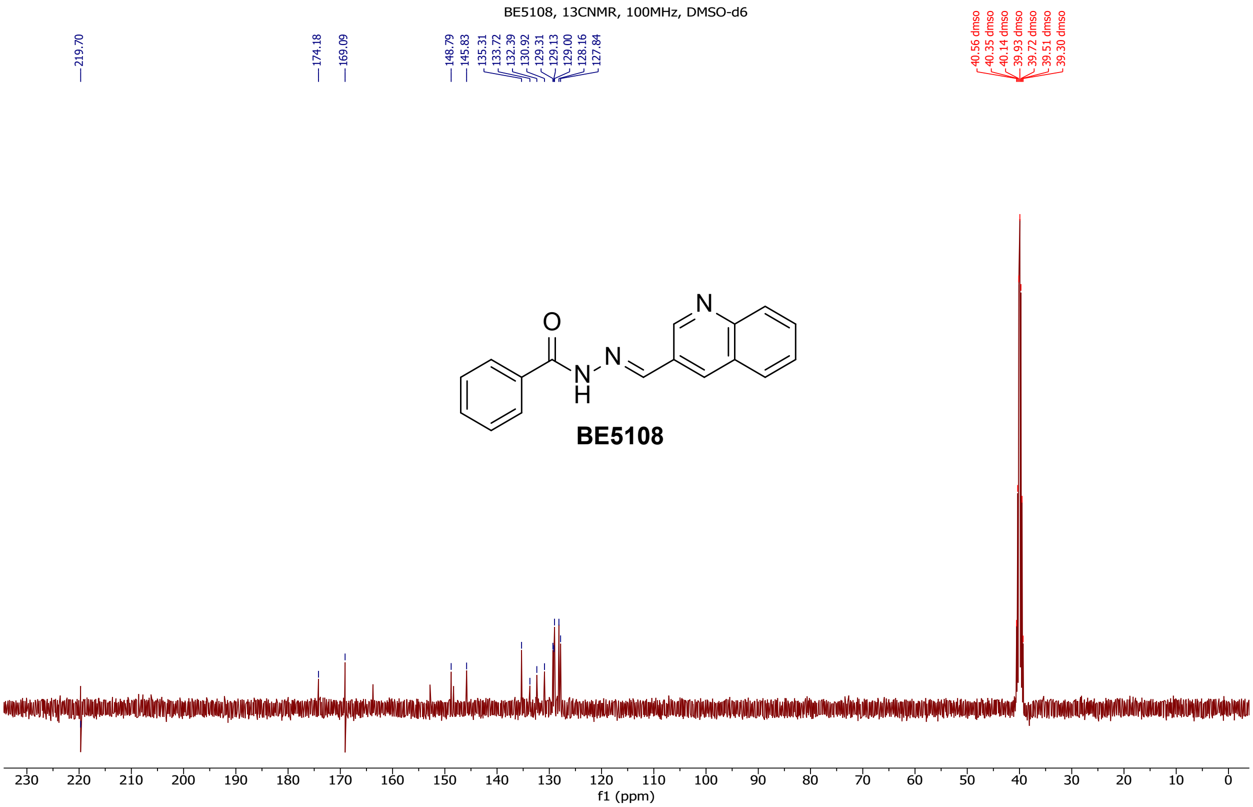

Figure S46. $^{13}C\{^{1}H\}$ NMR spectra of compound BE5108

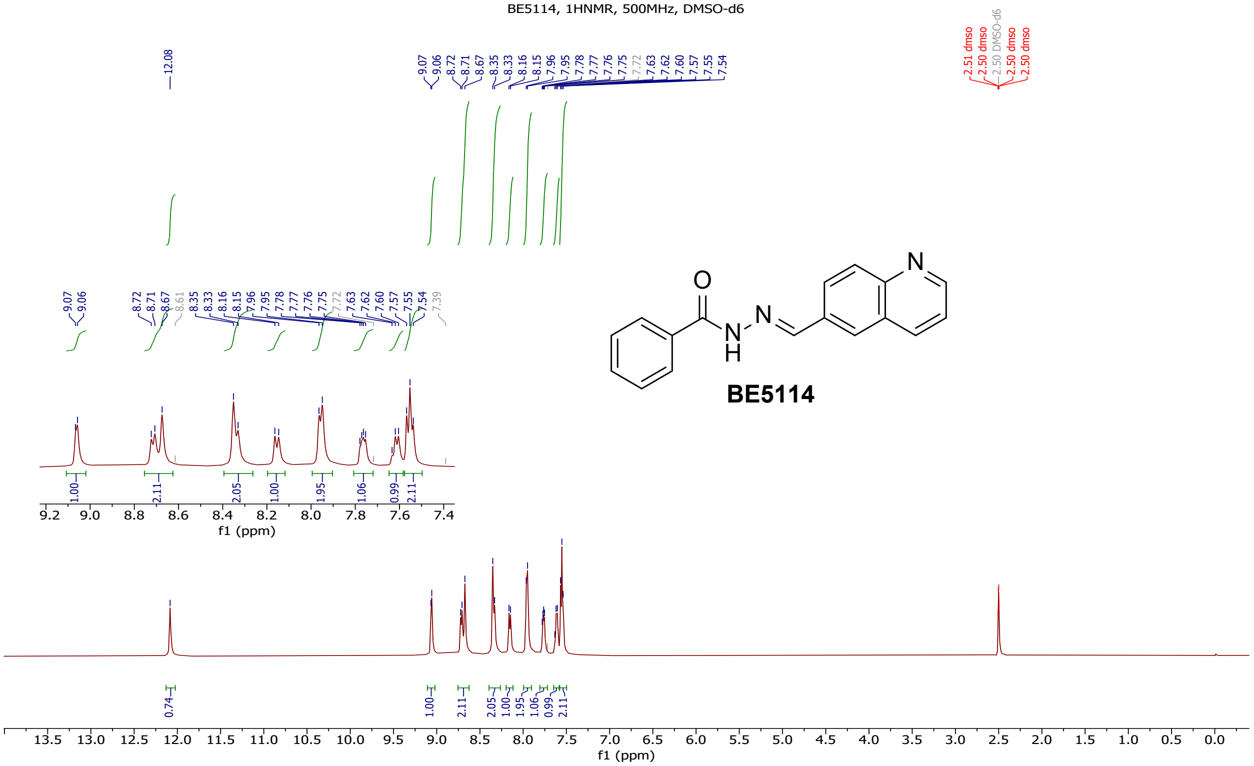

Figure S47. ^{1}H NMR spectra of compound BE5114

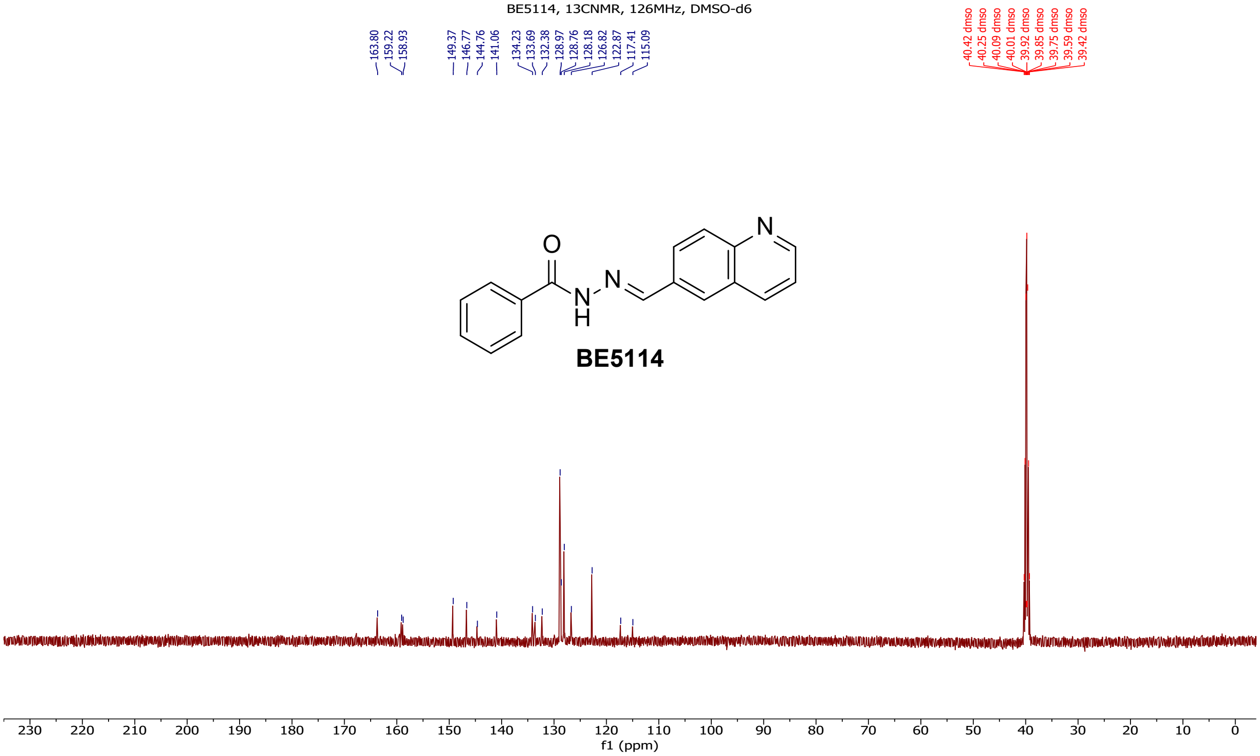

Figure S48. ^{13}C{^{1}H} NMR spectra of compound BE5114

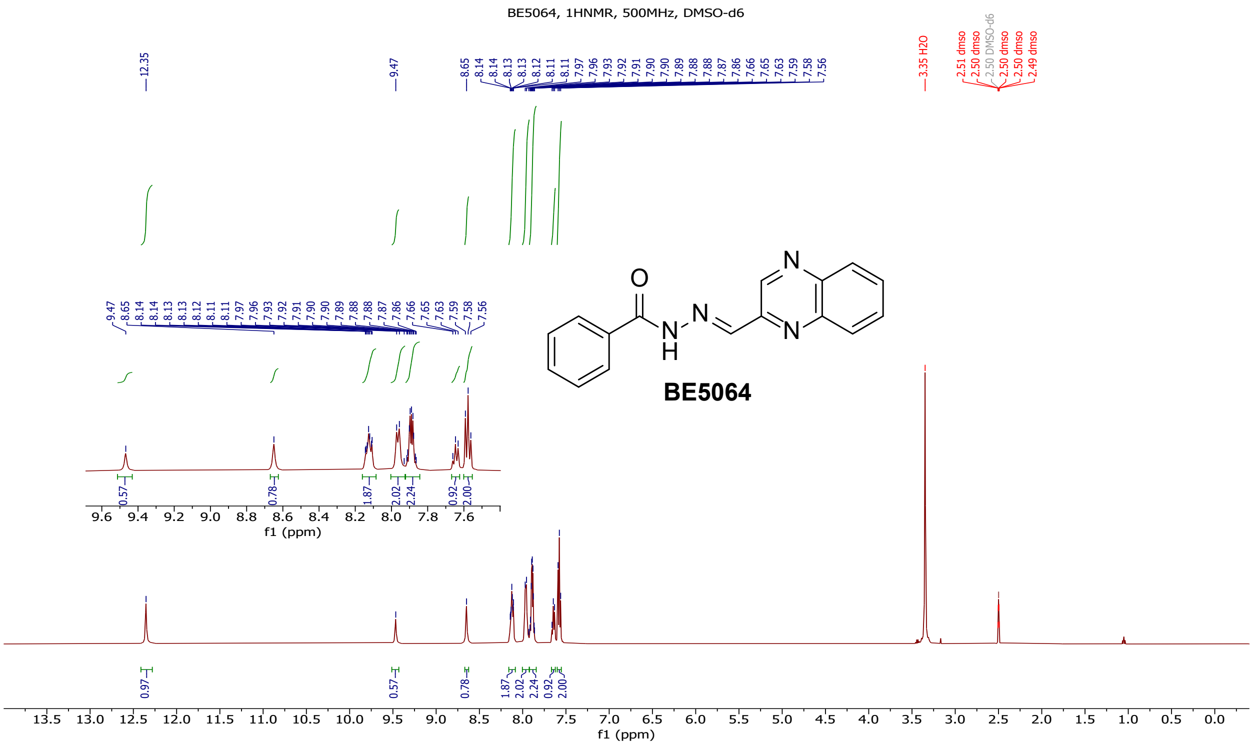

Figure S49. ^{1}H NMR spectra of compound BE5064

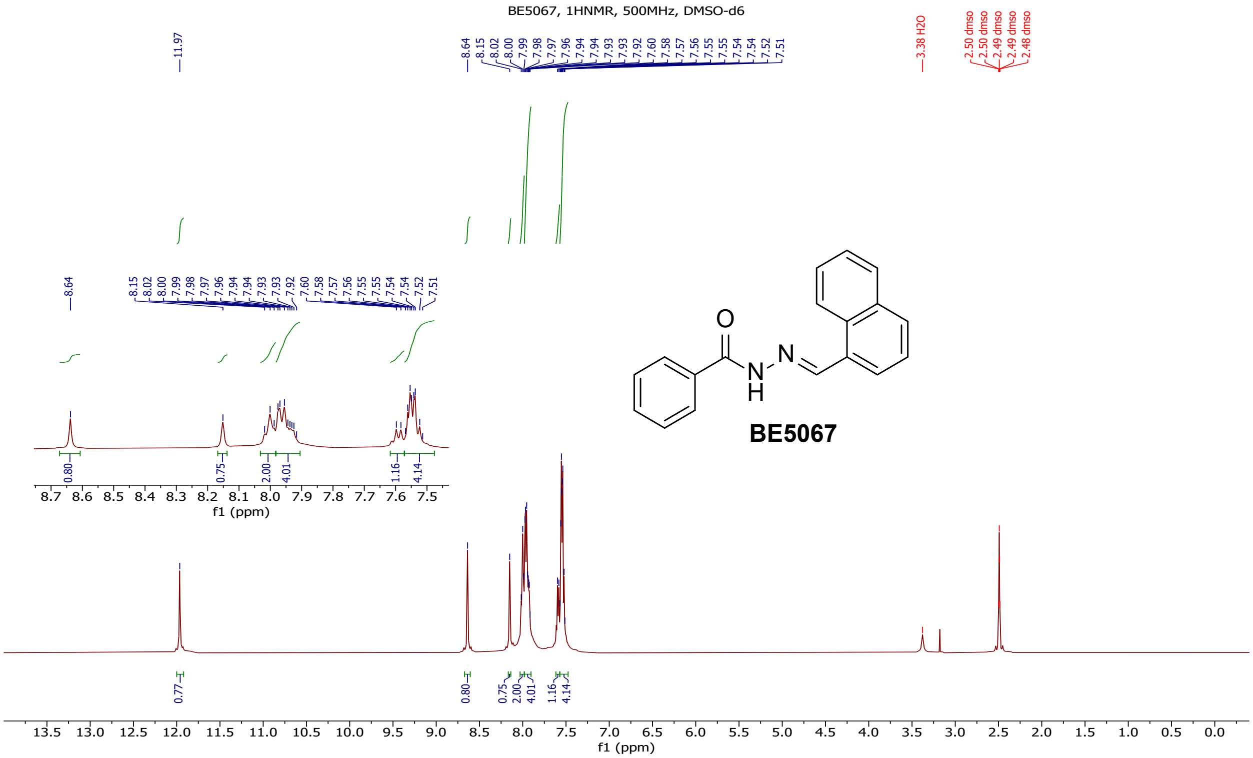

Figure S50. ^{1}H NMR spectra of compound BE5067

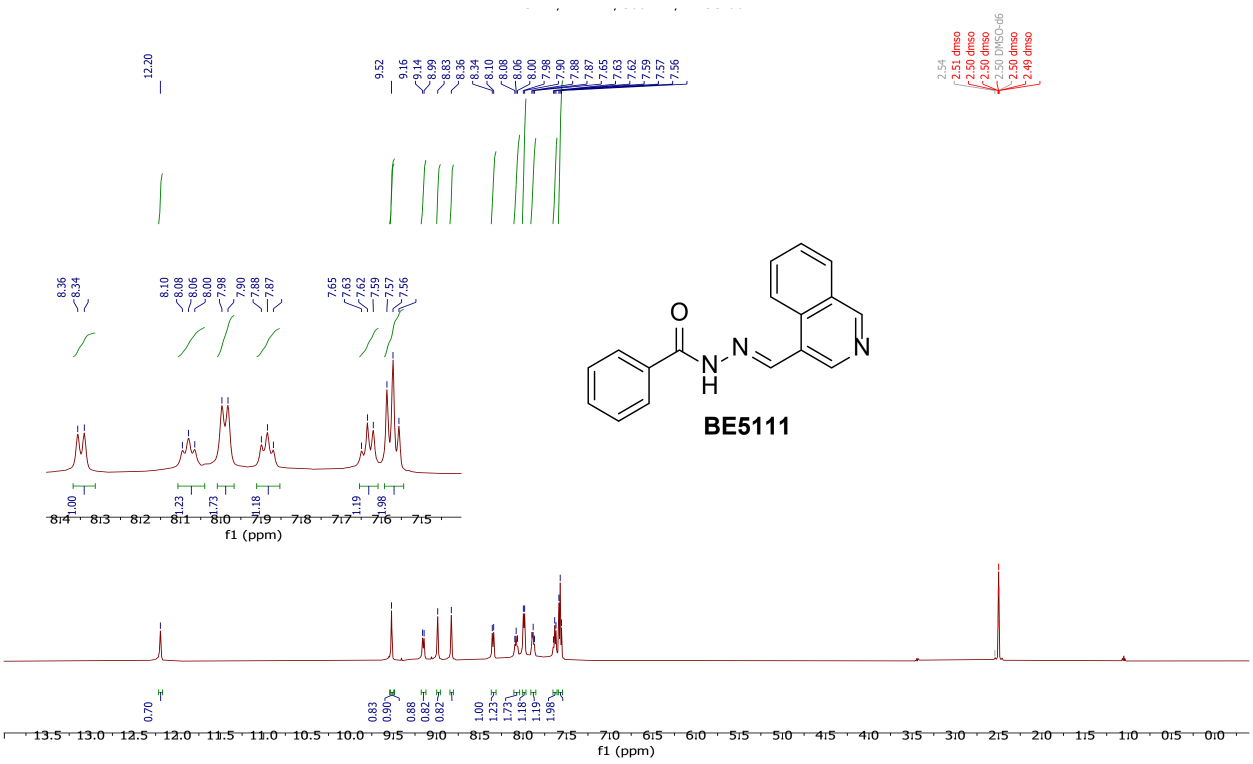

Figure S51. ^{1}H NMR spectra of compound BE5111

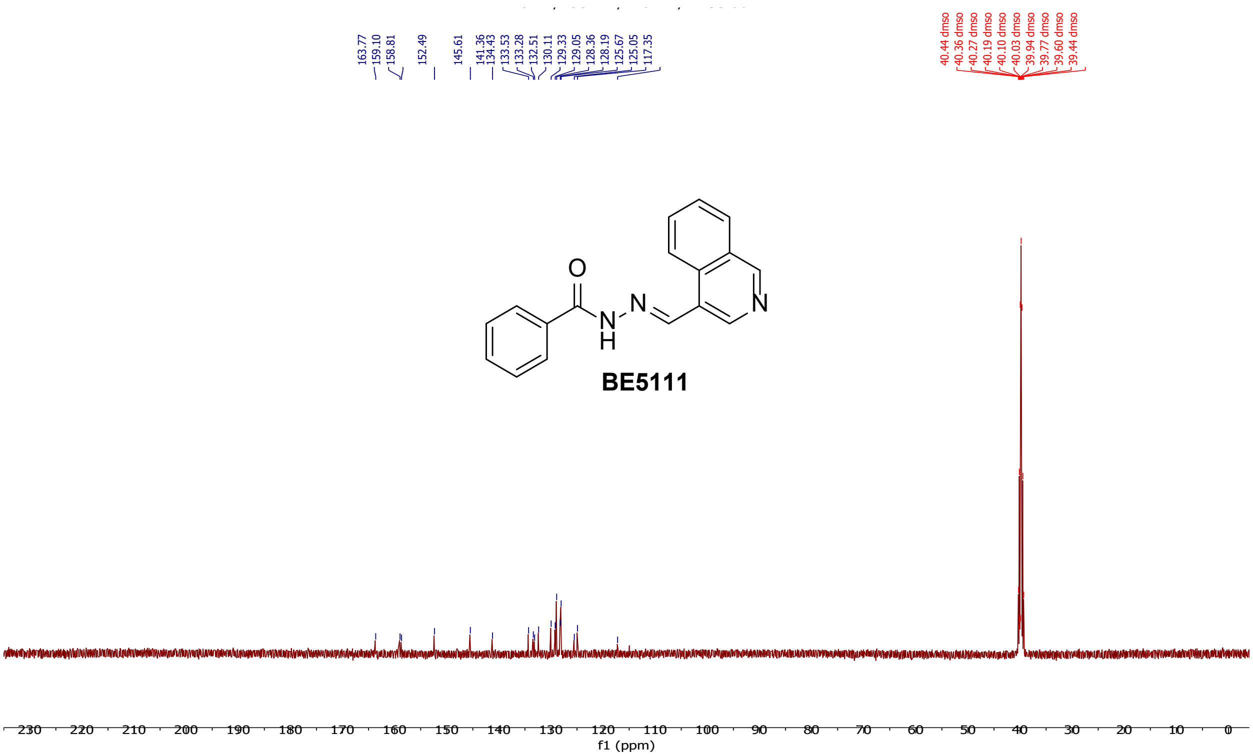

Figure S52. ^{13}C{^{1}H} NMR spectra of compound BE5111

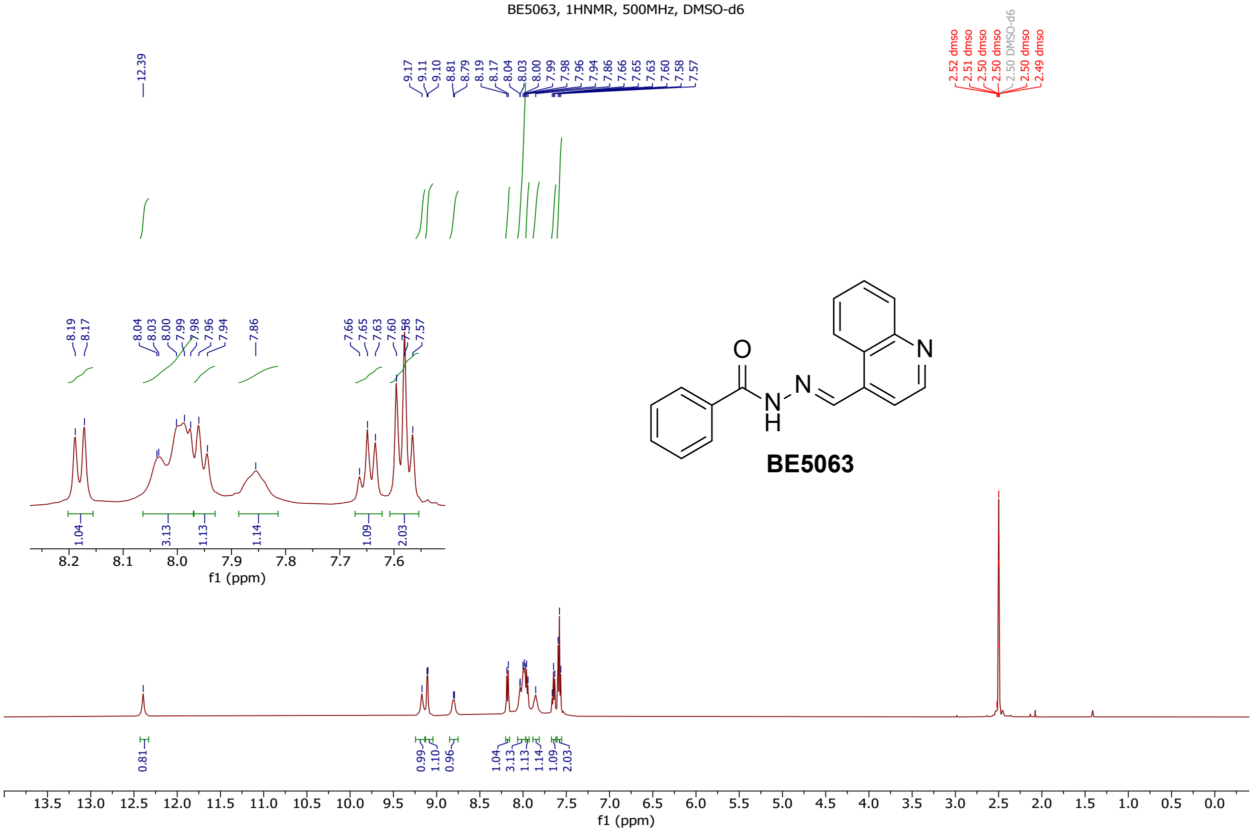

Figure S53. ^{1}H NMR spectra of compound BE5063

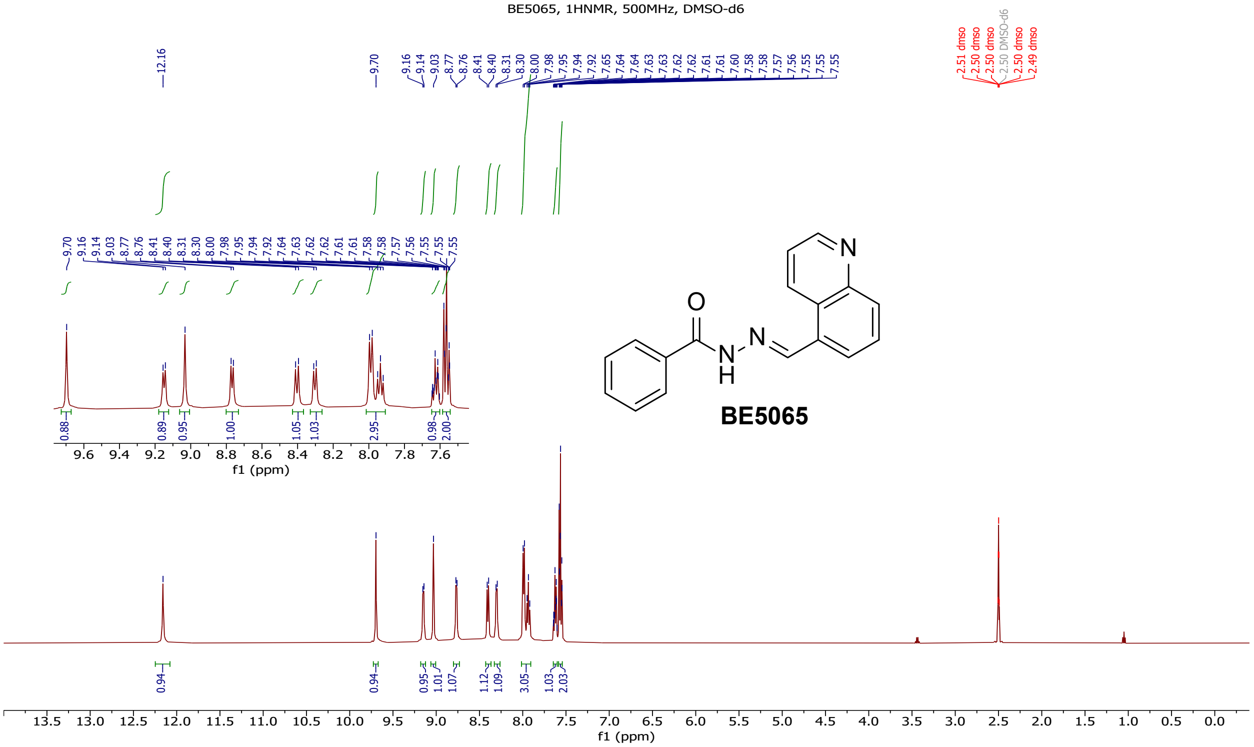

Figure S54. **^{1}H NMR spectra of compound BE5065**

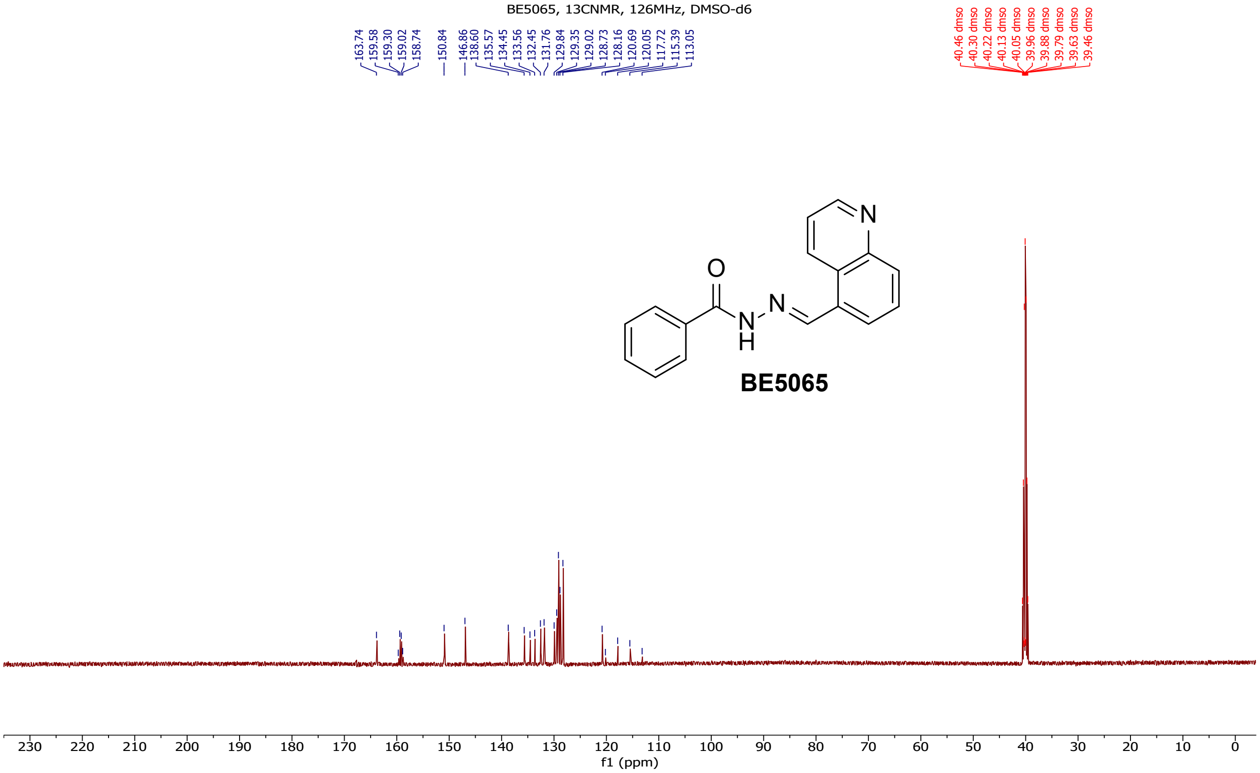

Figure S55. **^{13}C{^{1}H} NMR spectra of compound BE5065**

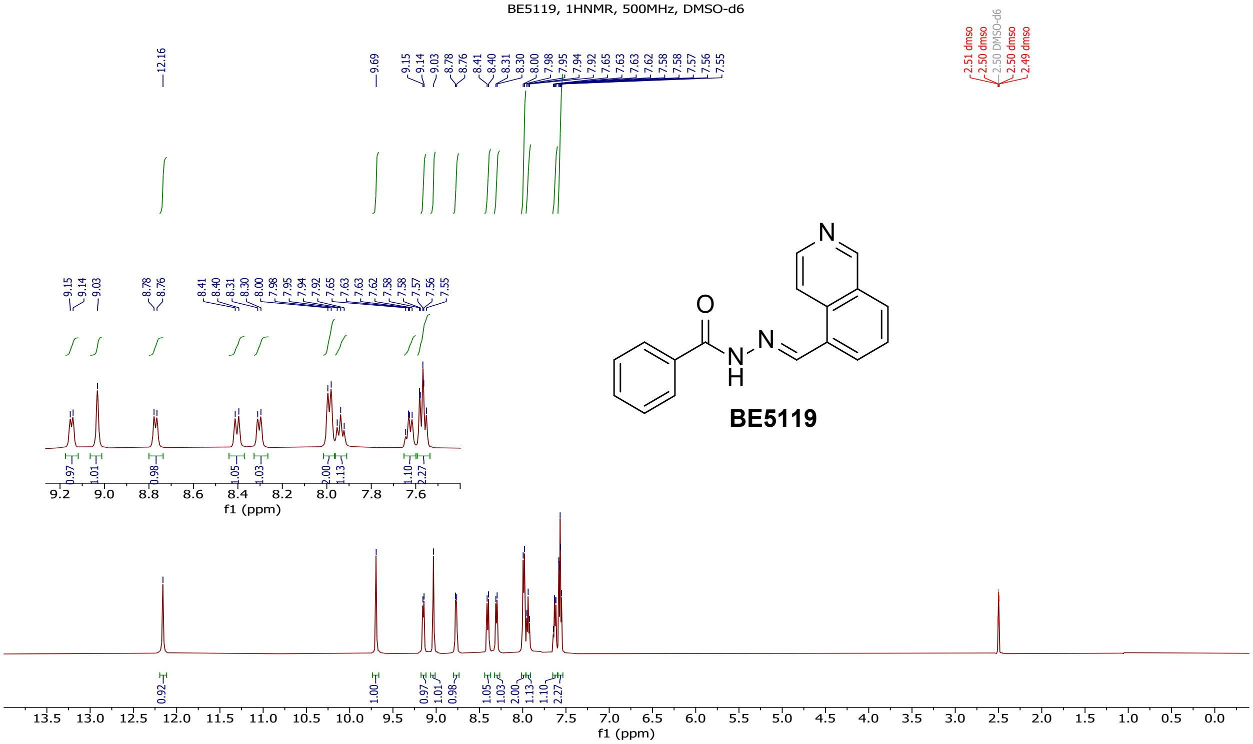

Figure S56. ^{1}H NMR spectra of compound BE5119

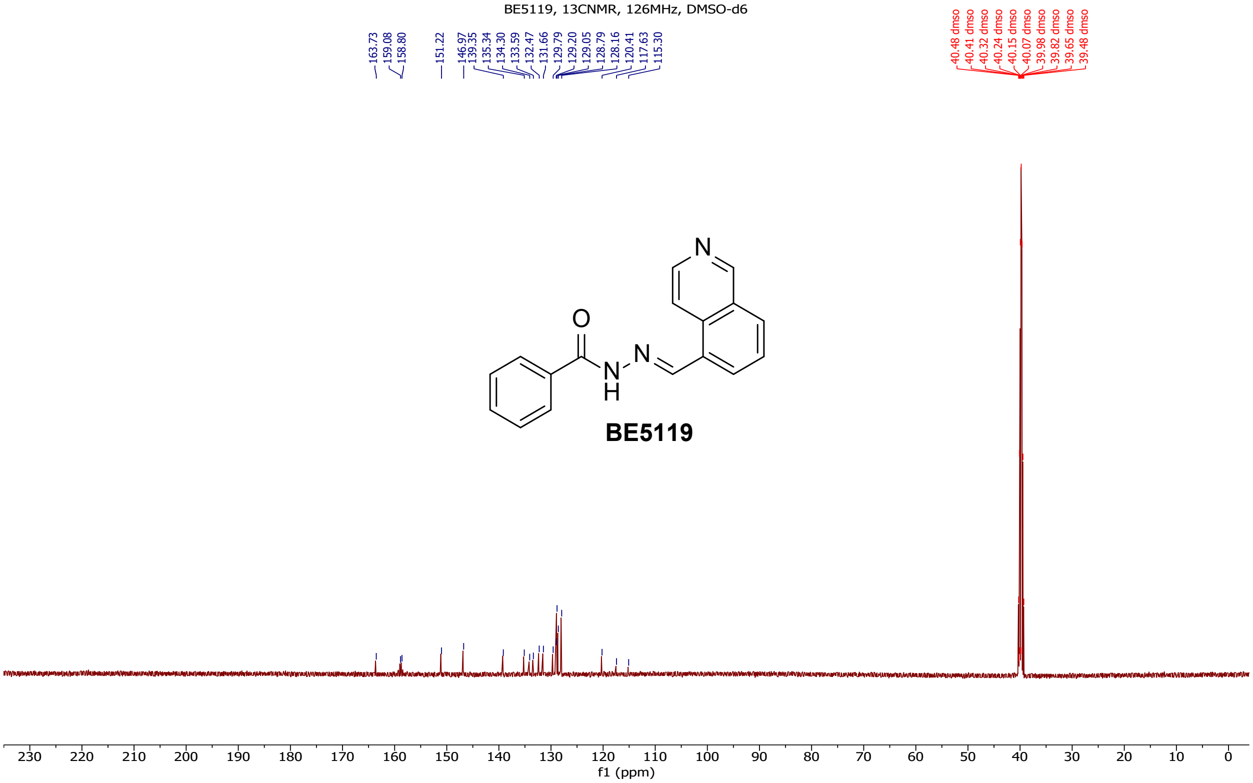

Figure S57. ^{13}C{^{1}H} NMR spectra of compound BE5119

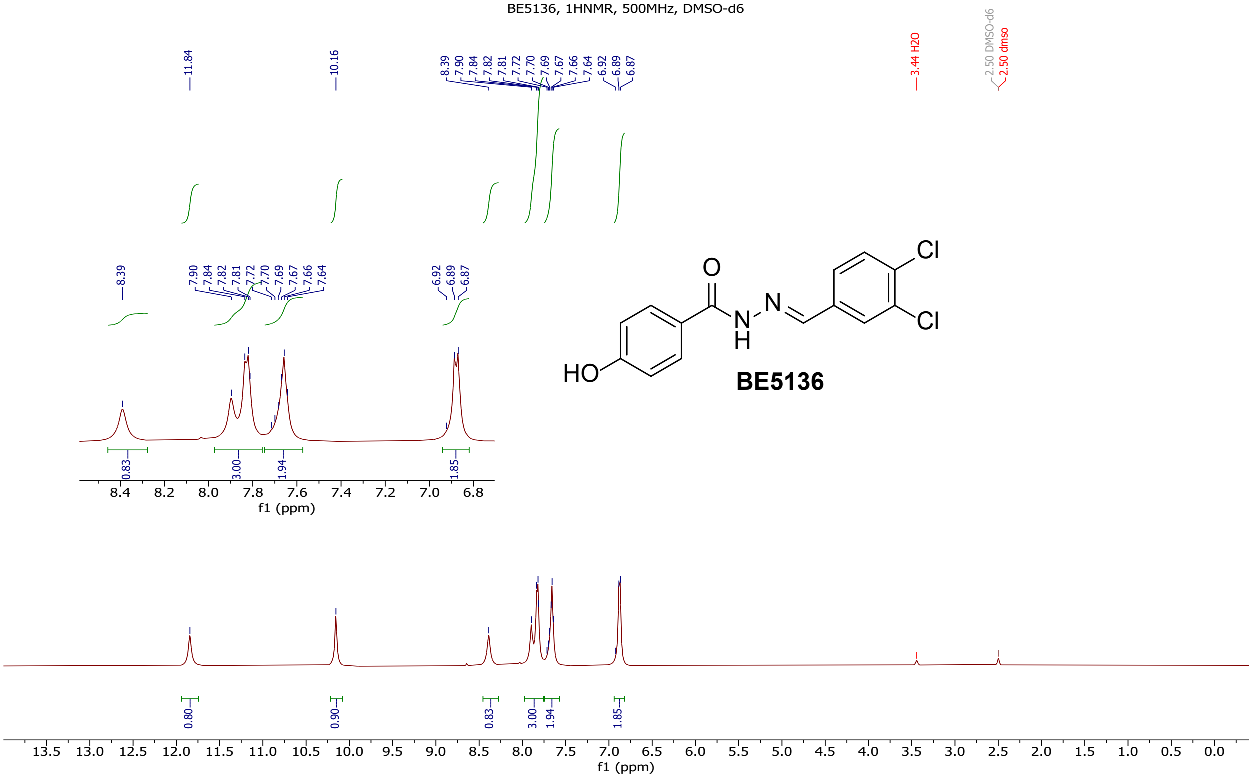

Figure S58. ^{1}H NMR spectra of compound BE5136

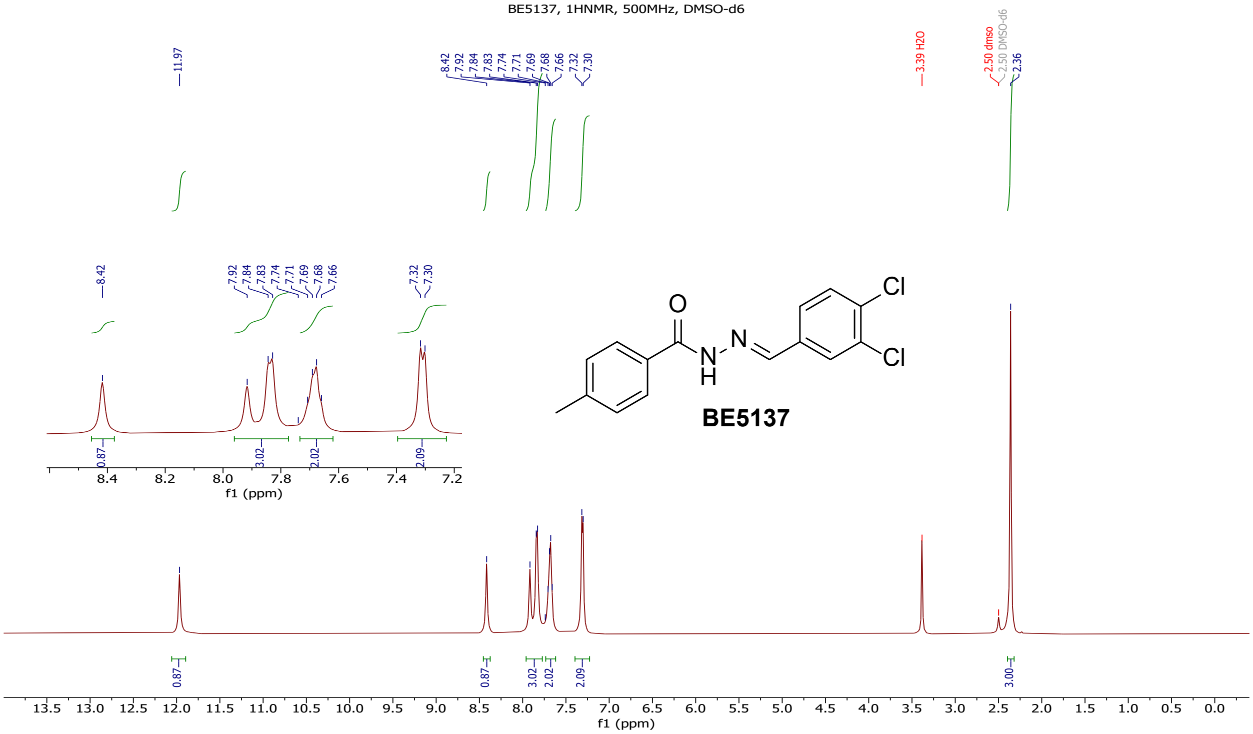

Figure S59. ^{1}H NMR spectra of compound BE5137

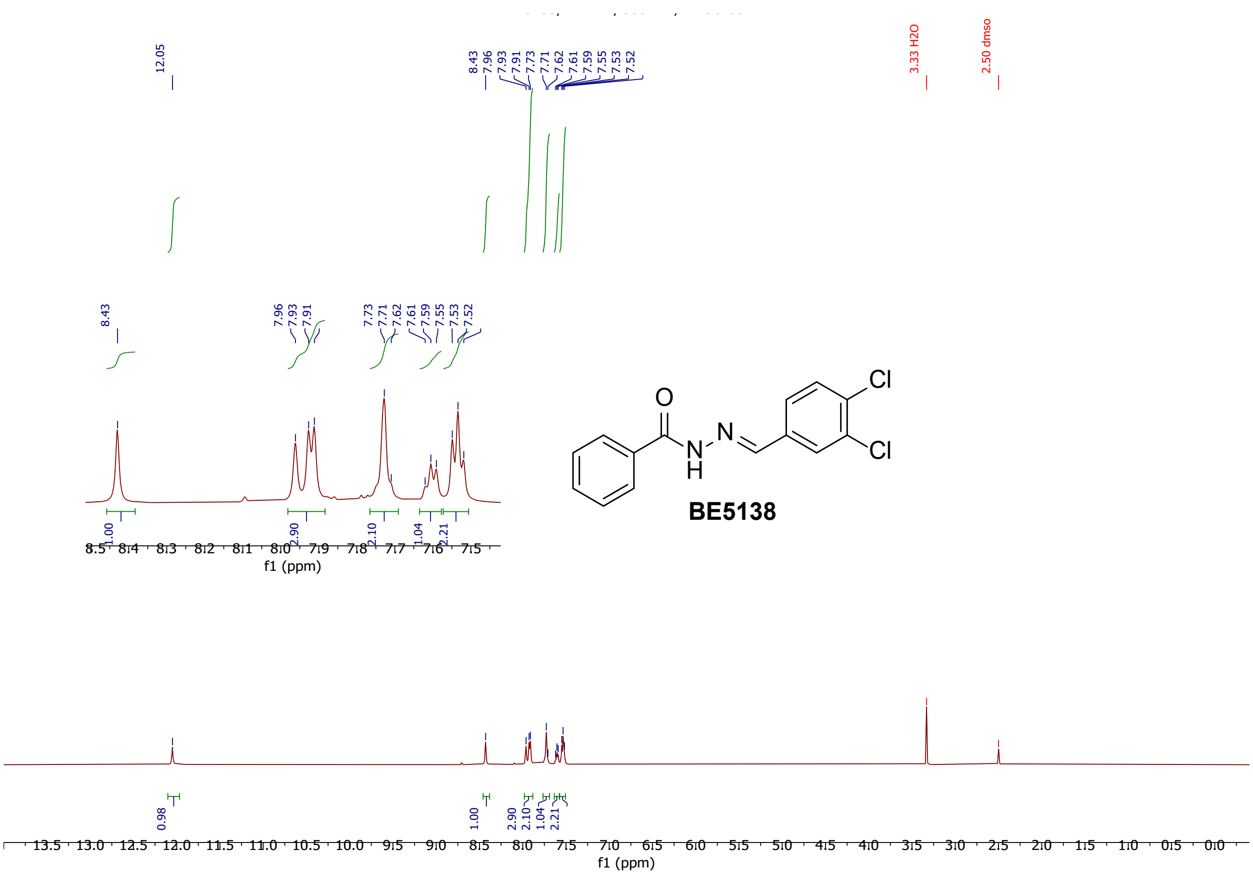

Figure S60. ^{1}H NMR spectra of compound BE5138

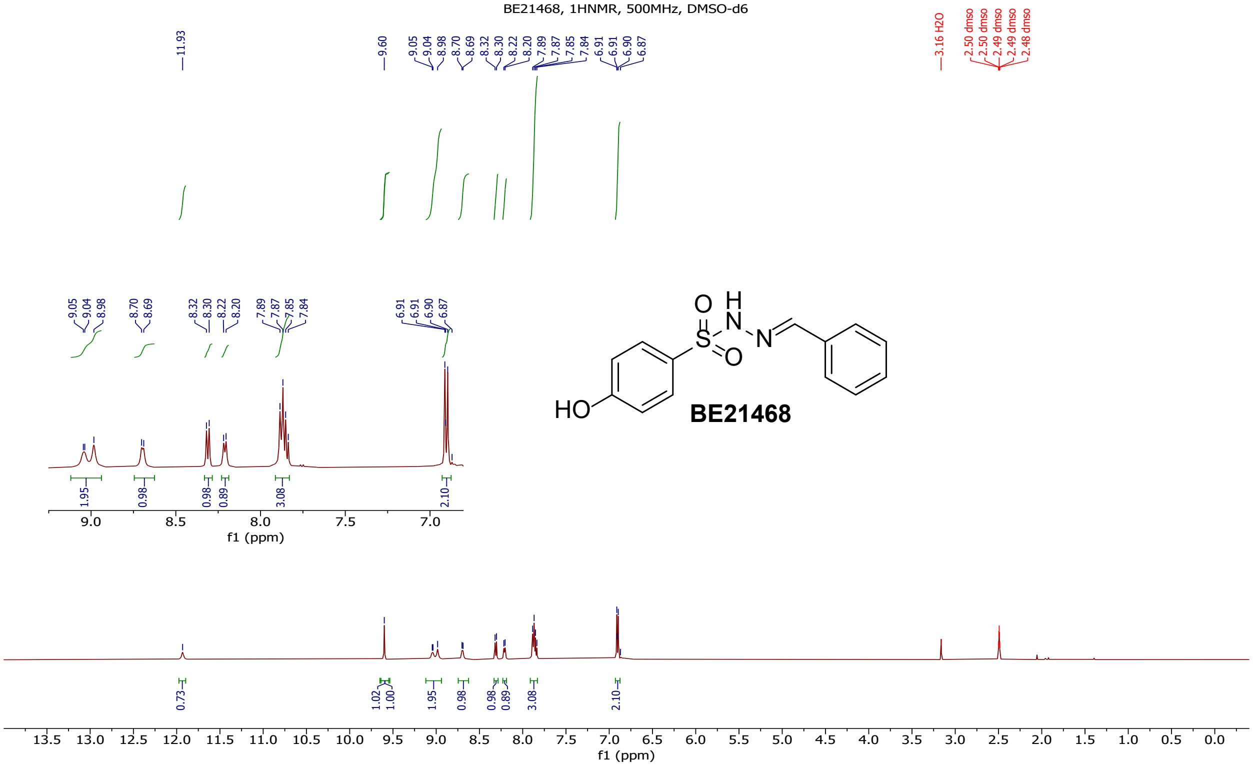

Figure S61. ^{1}H NMR spectra of compound BE21468

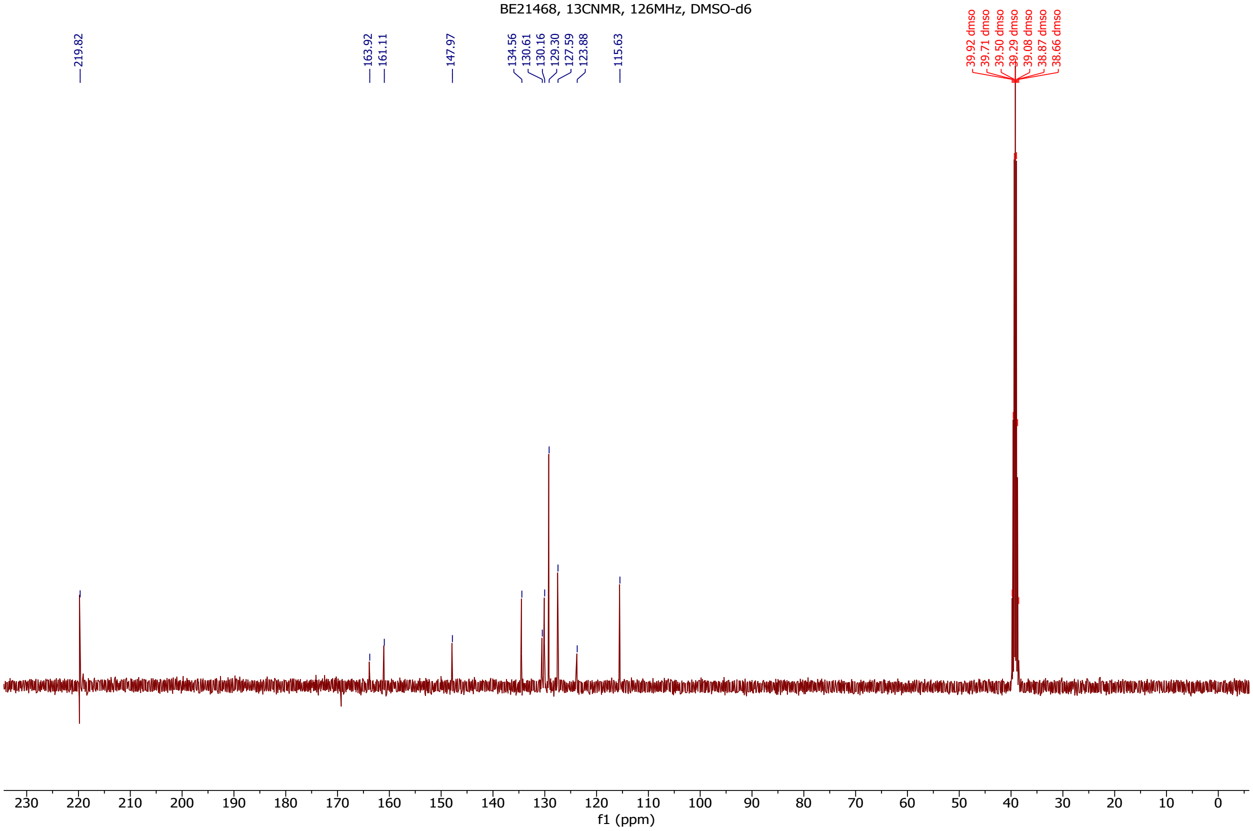

Figure S62. ^{13}C{^{1}H} NMR spectra of compound BE21468

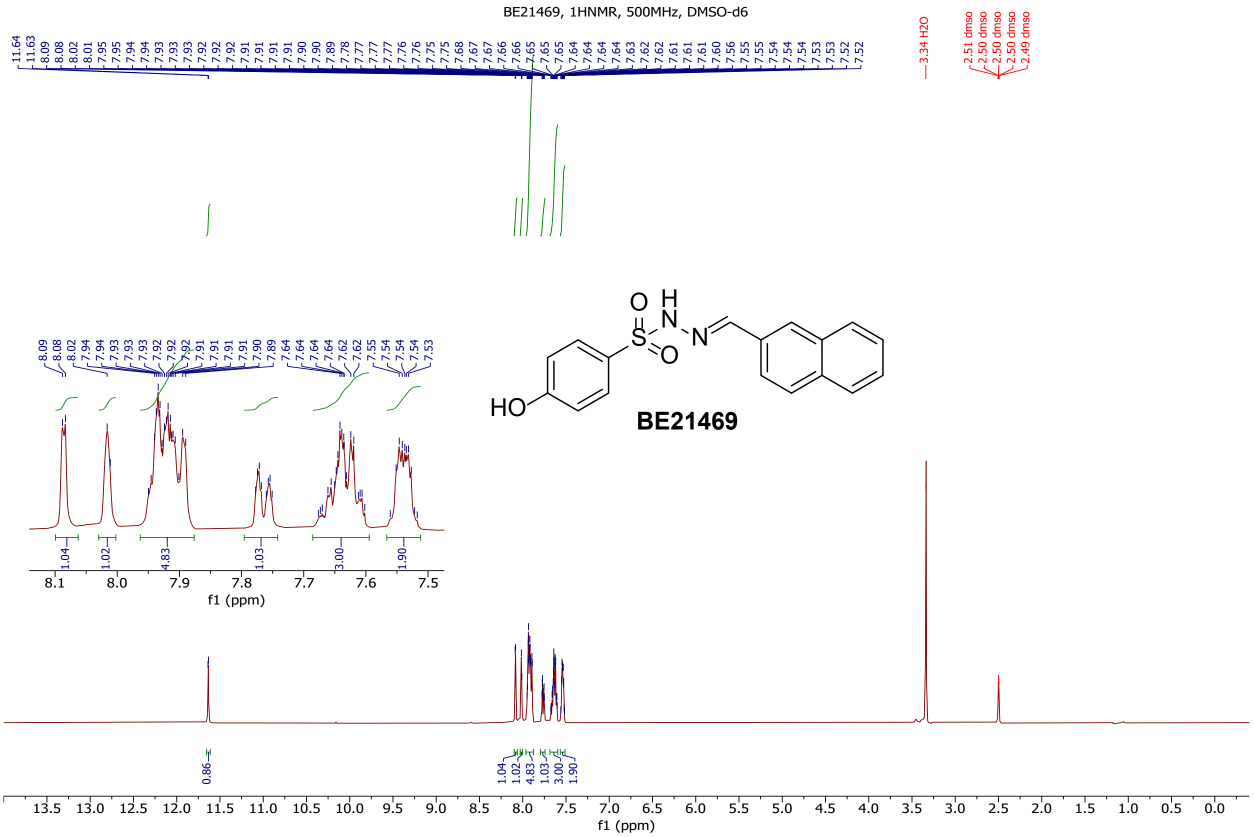

Figure S63. ^{1}H NMR spectra of compound BE21469

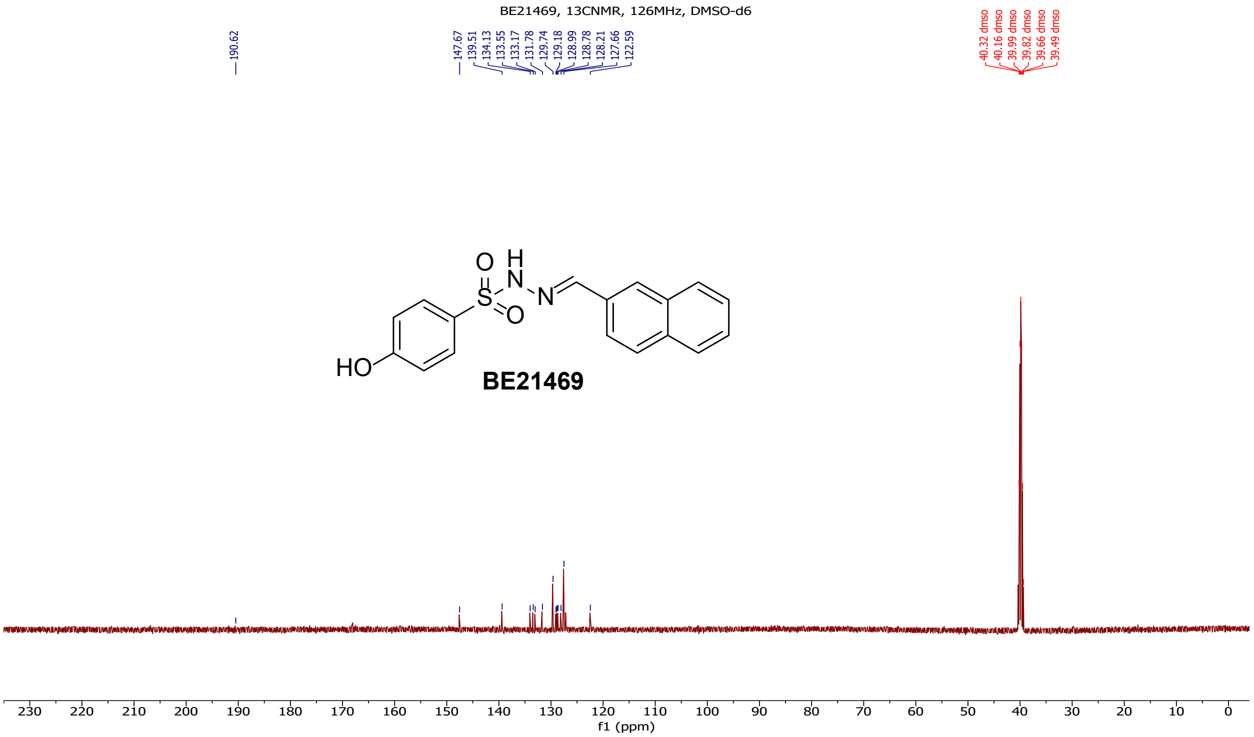

Figure S64. $^{13}C\{^{1}H\}$ NMR spectra of compound BE21469

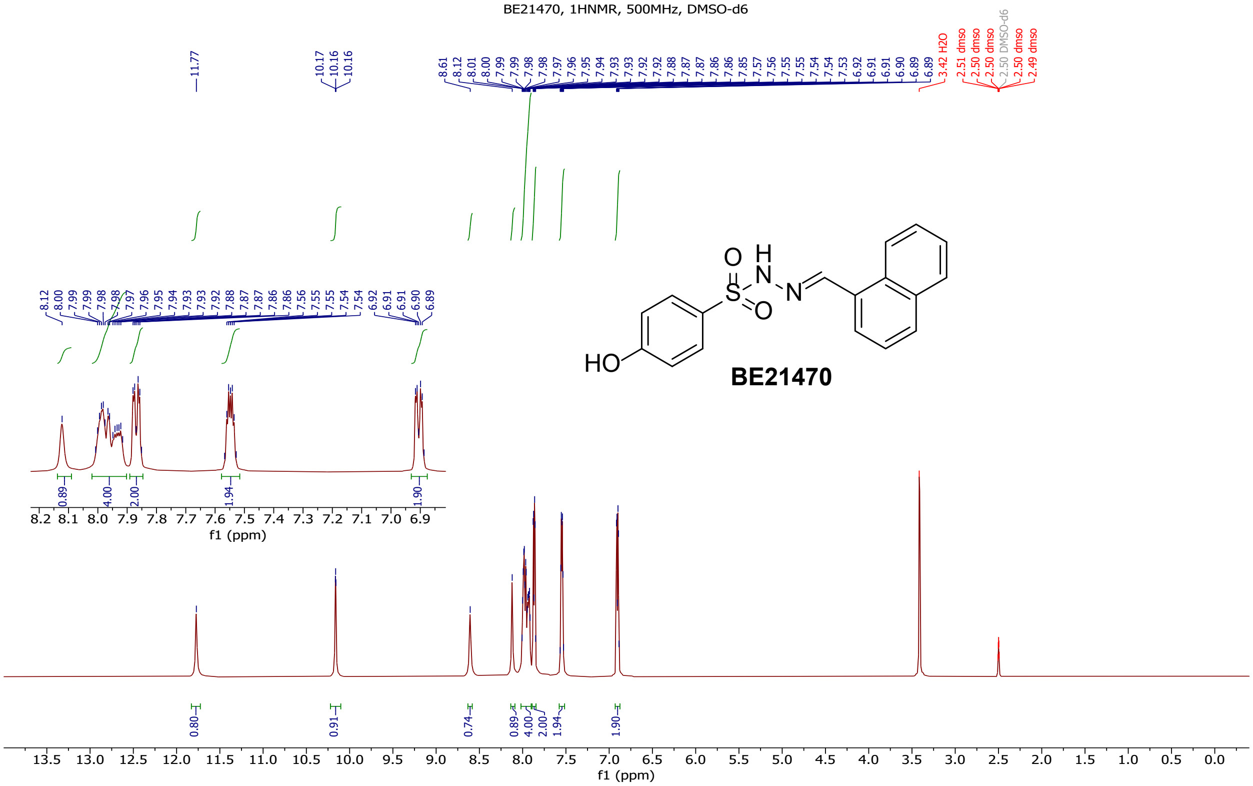

Figure S65. ^{1}**H NMR spectra of compound BE21470**

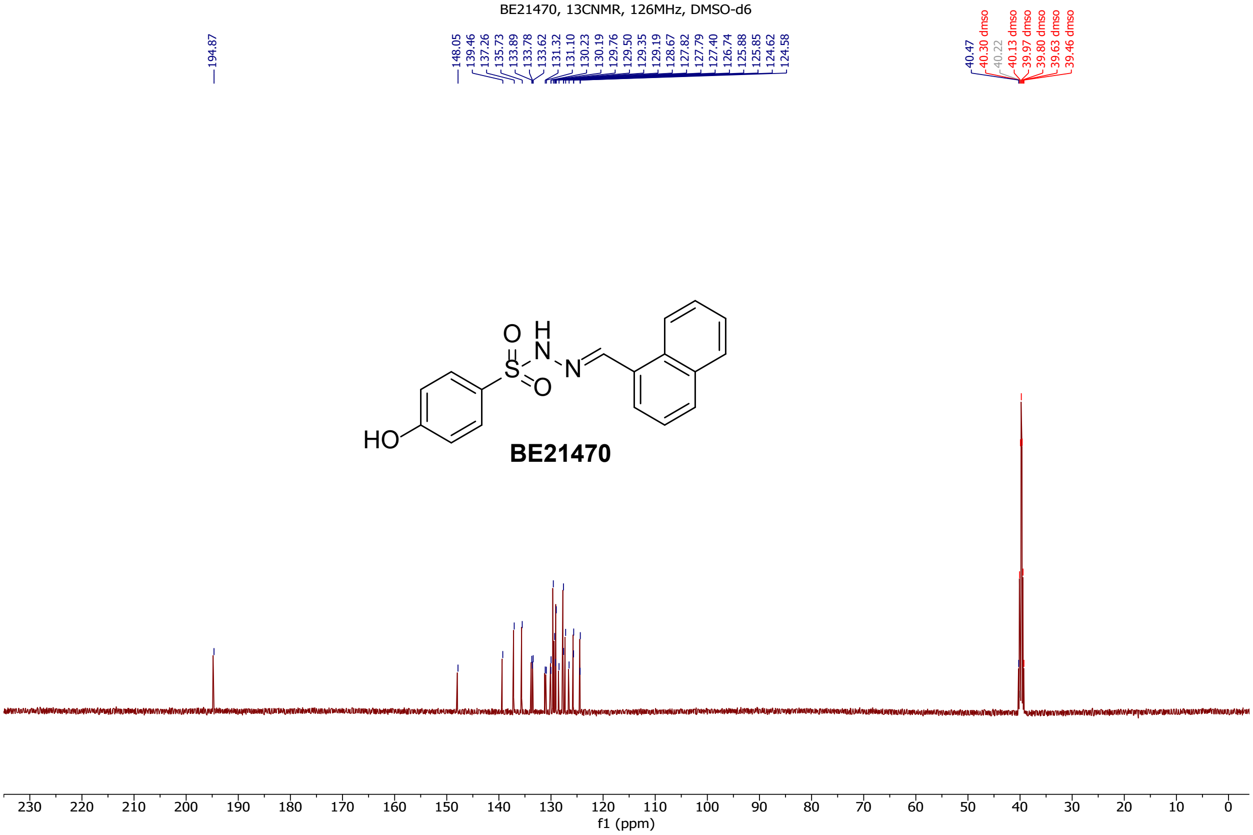

Figure S66. ^{13}**C{**^{1}**H} NMR spectra of compound BE21470**

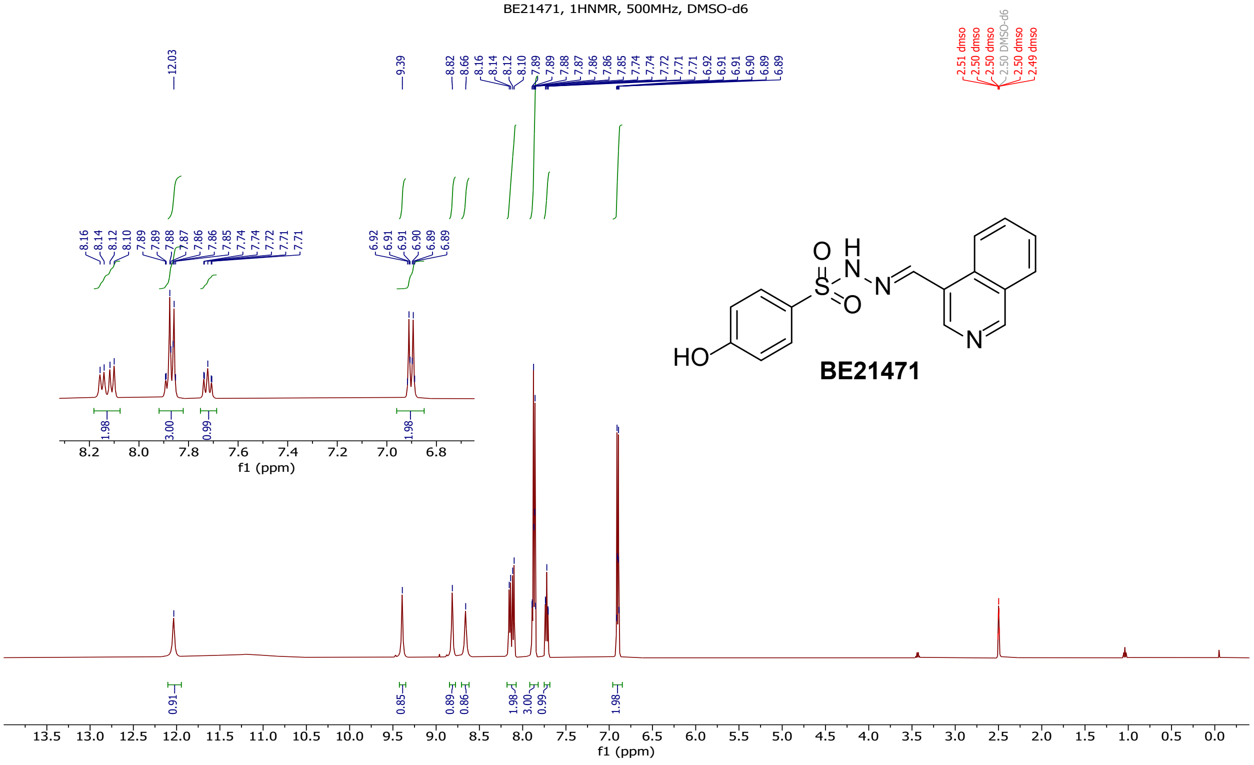

Figure S67. ^{1}H NMR spectra of compound BE21471

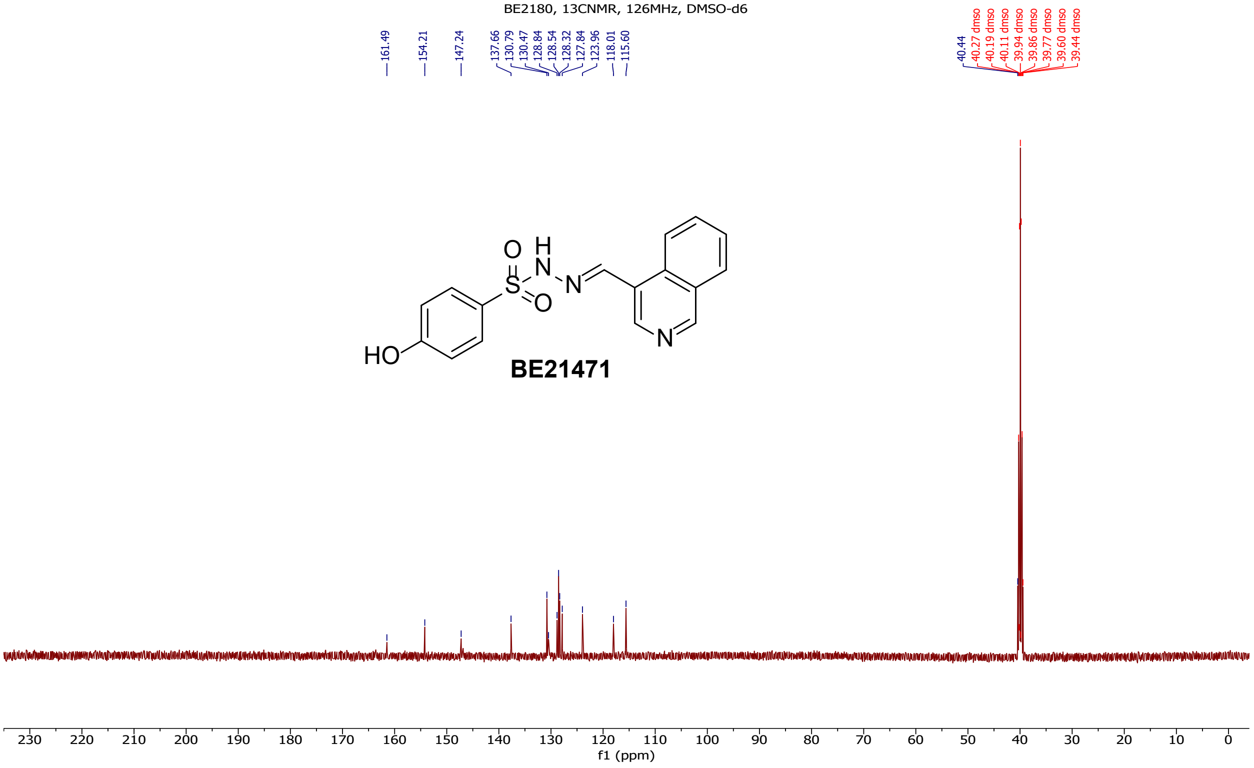

Figure S68. $^{13}C\{^{1}H\}$ NMR spectra of compound BE21471

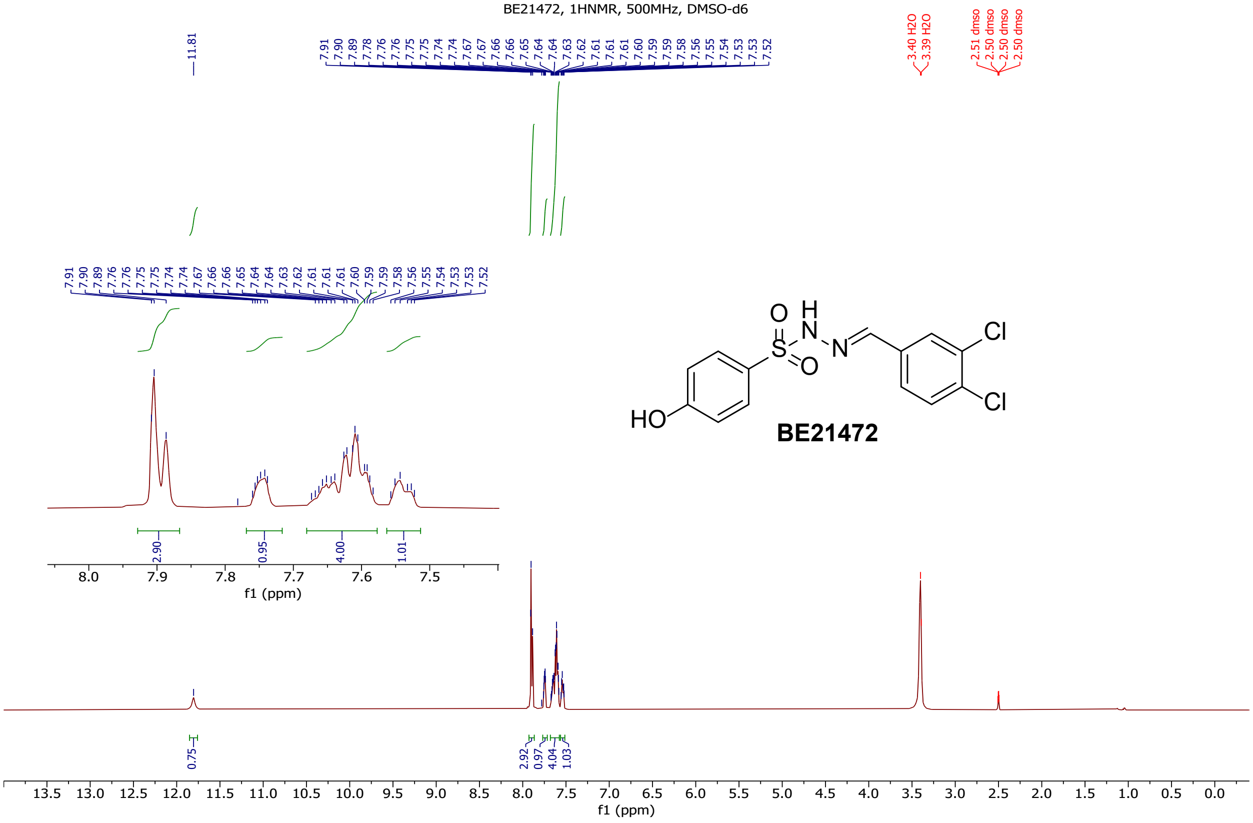

Figure S69. ^{1}H NMR spectra of compound BE21472

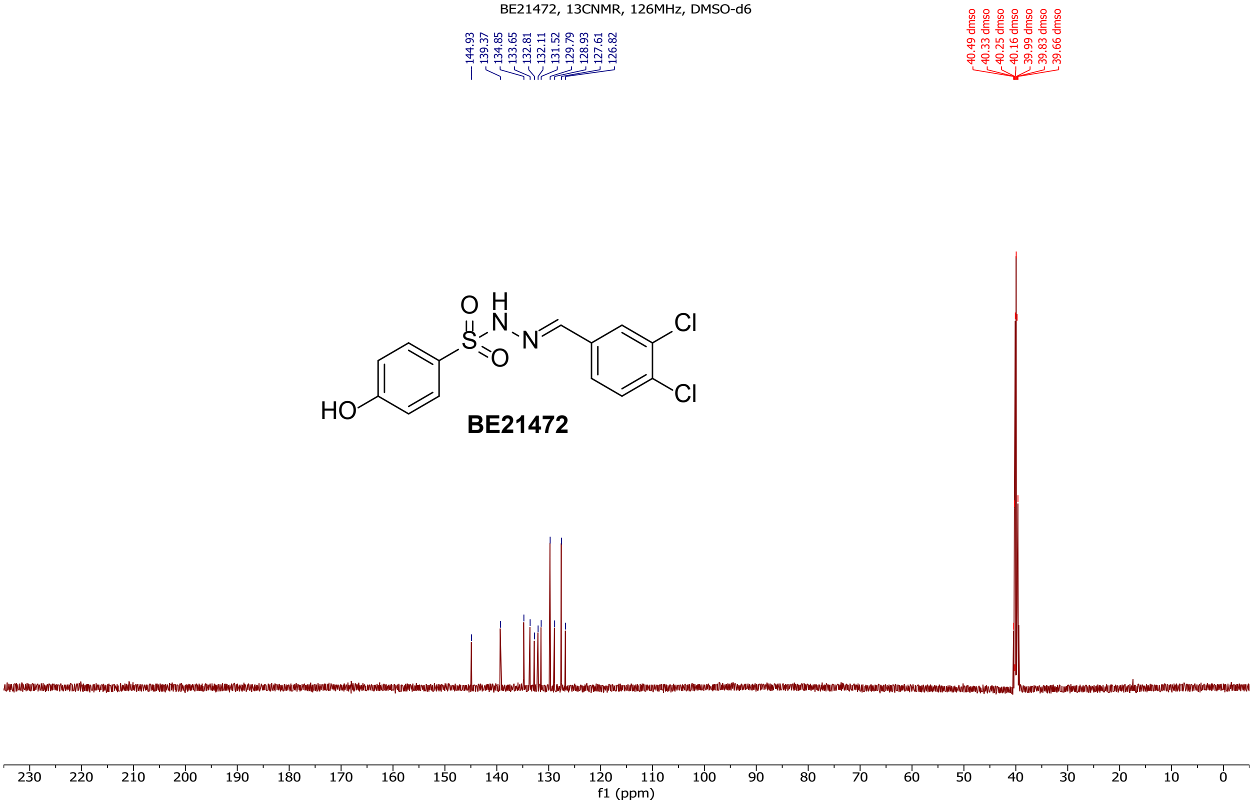

Figure S70. ^{13}C{^{1}H} NMR spectra of compound BE21472

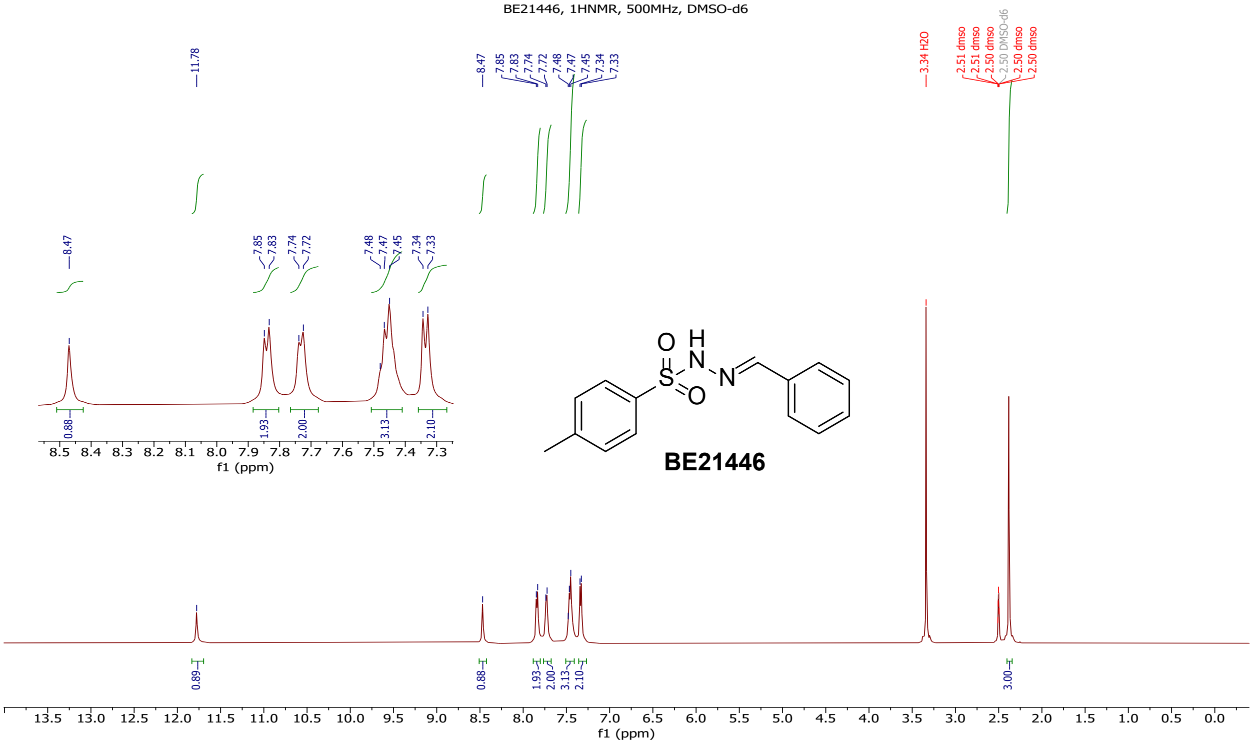

Figure S71. ^{1}H NMR spectra of compound BE21446

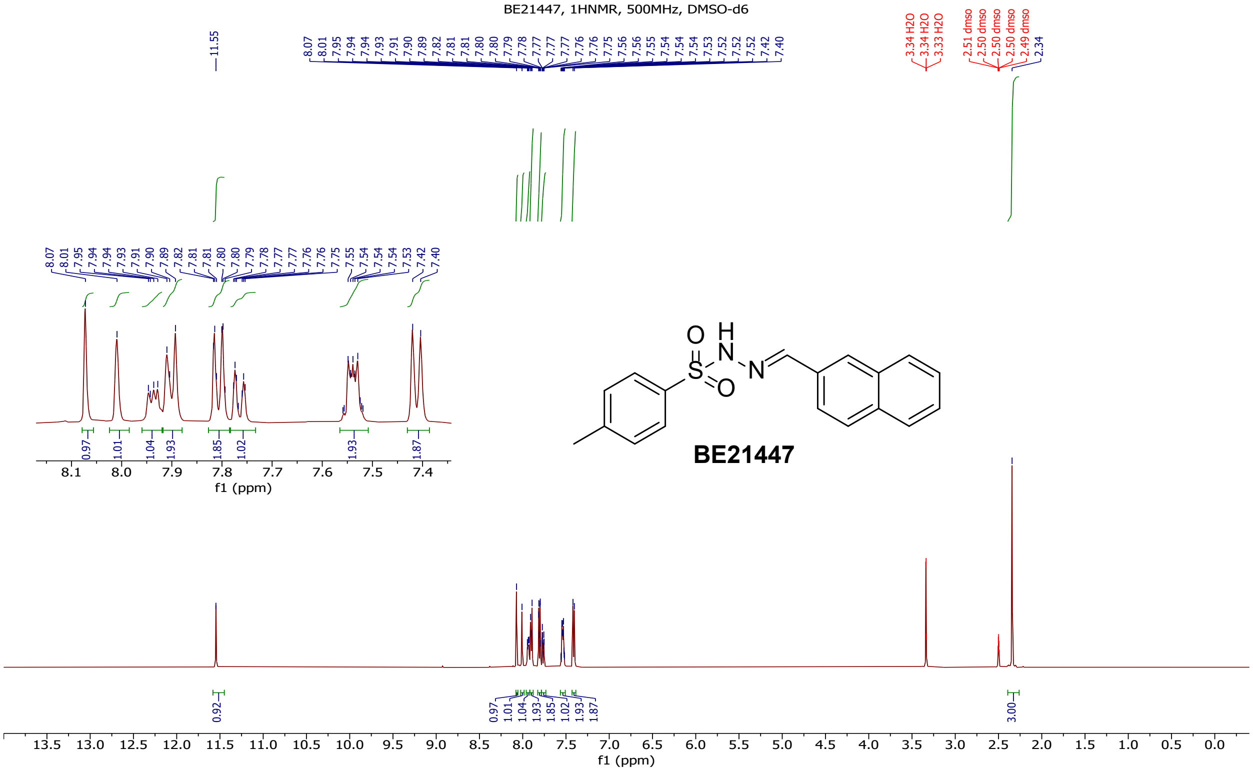

Figure S72. ^{1}H NMR spectra of compound BE21447

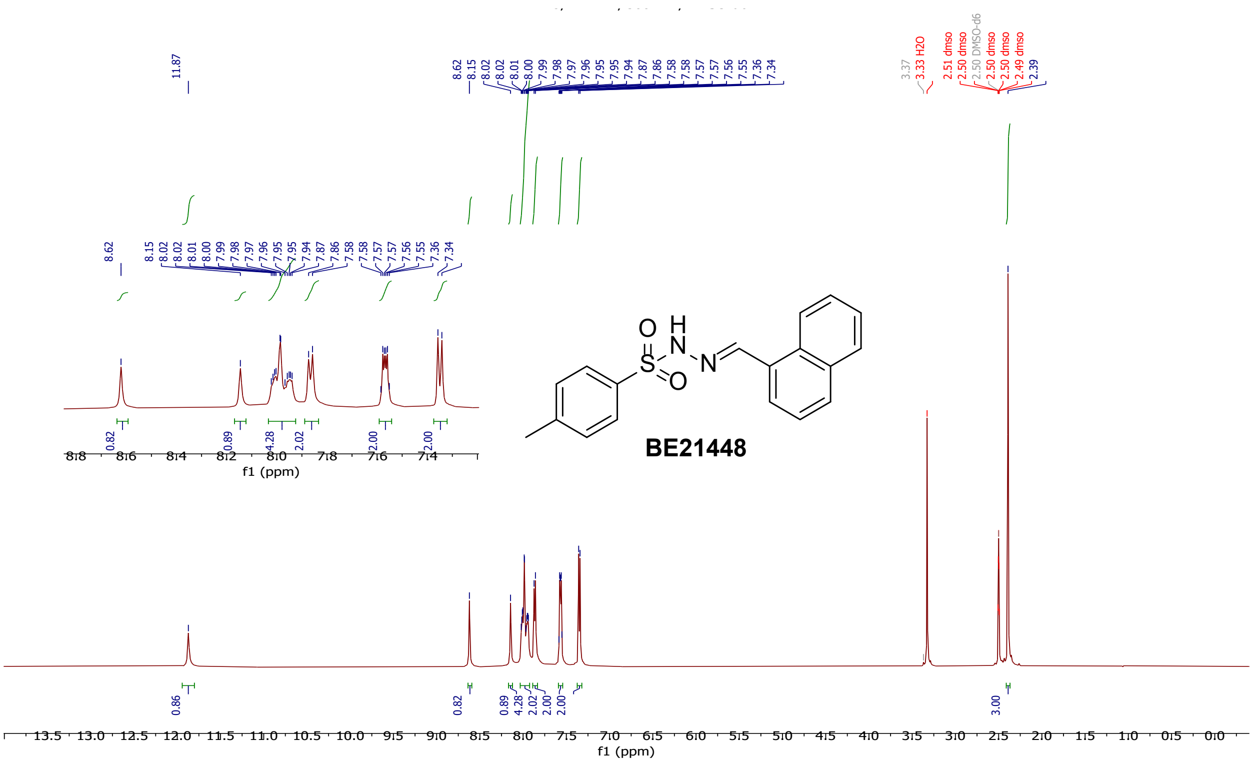

Figure S73. ^{1}H NMR spectra of compound BE21448

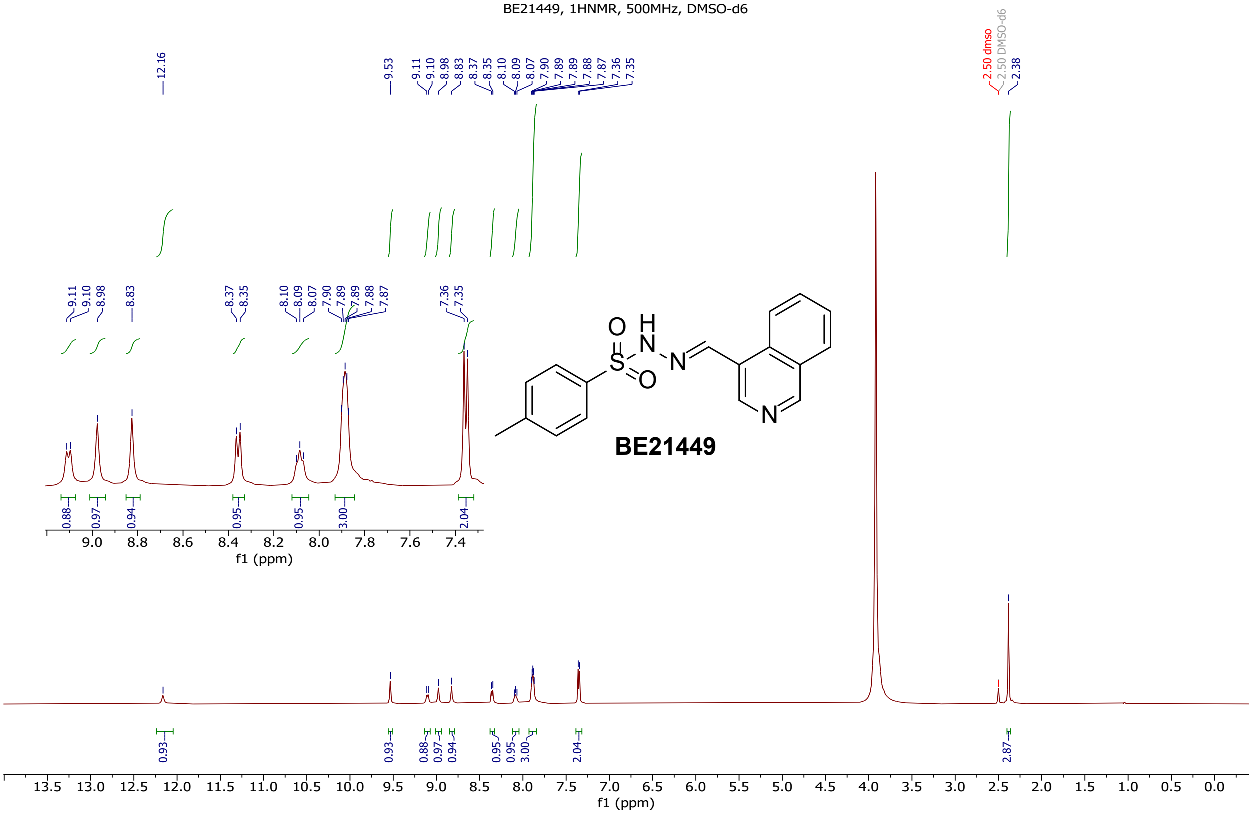

Figure S74. ^{1}**H NMR spectra of compound BE21449**

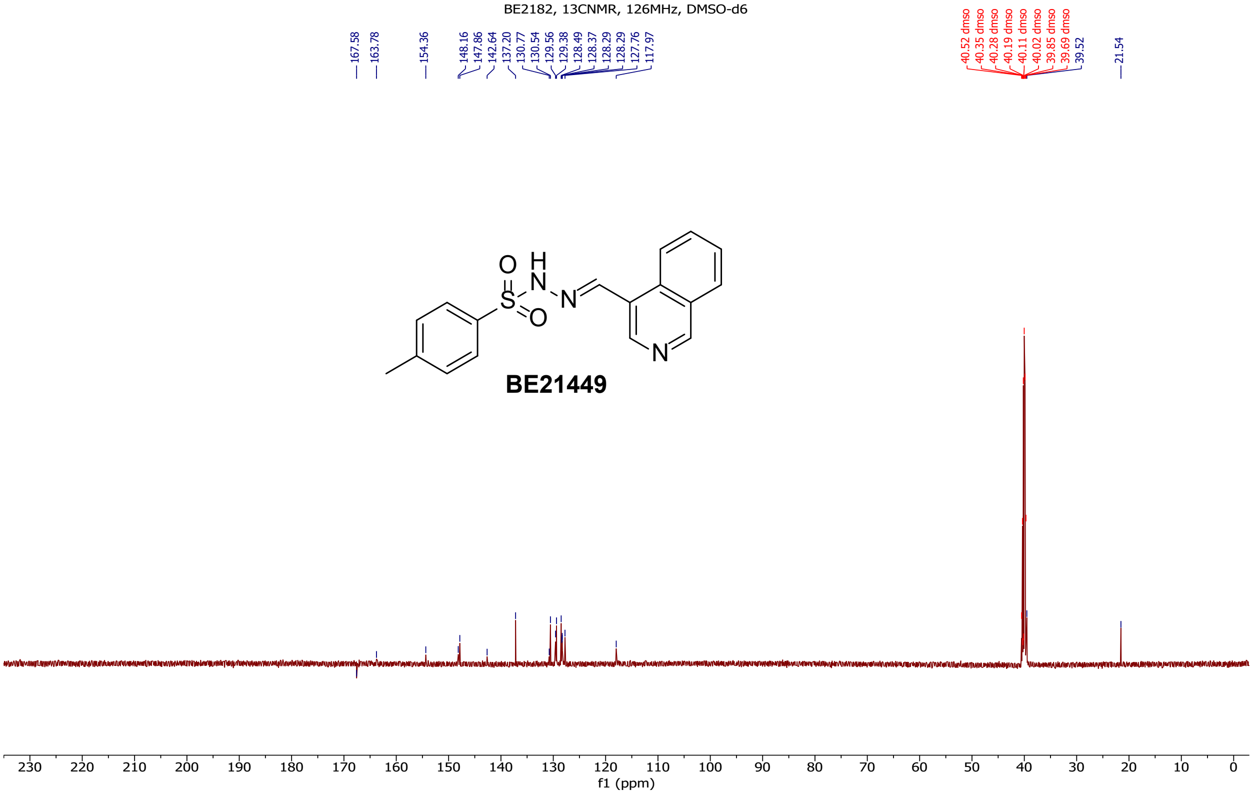

Figure S75. ^{13}**C{**^{1}**H} NMR spectra of compound BE21449**

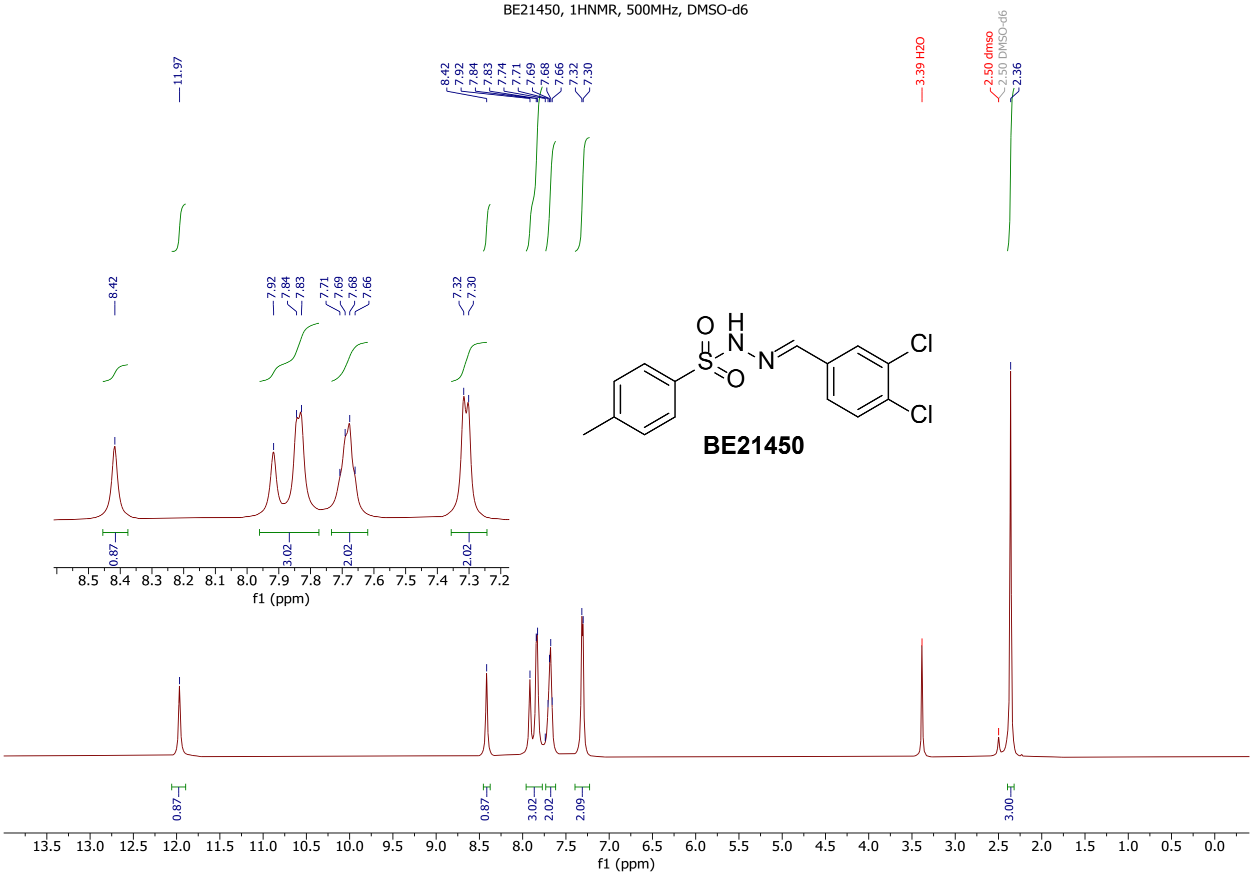

Figure S76. ^{1}H NMR spectra of compound BE21450

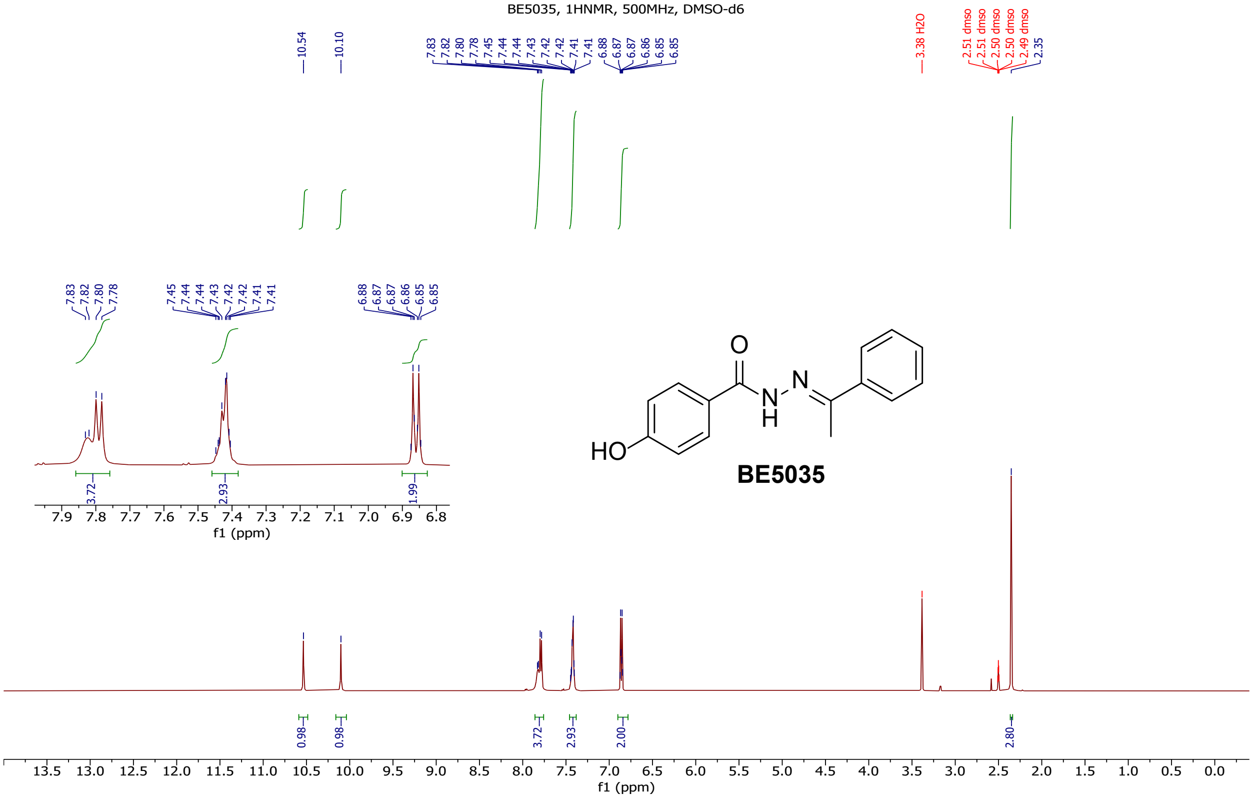

Figure S77. ^{1}H NMR spectra of compound BE5035

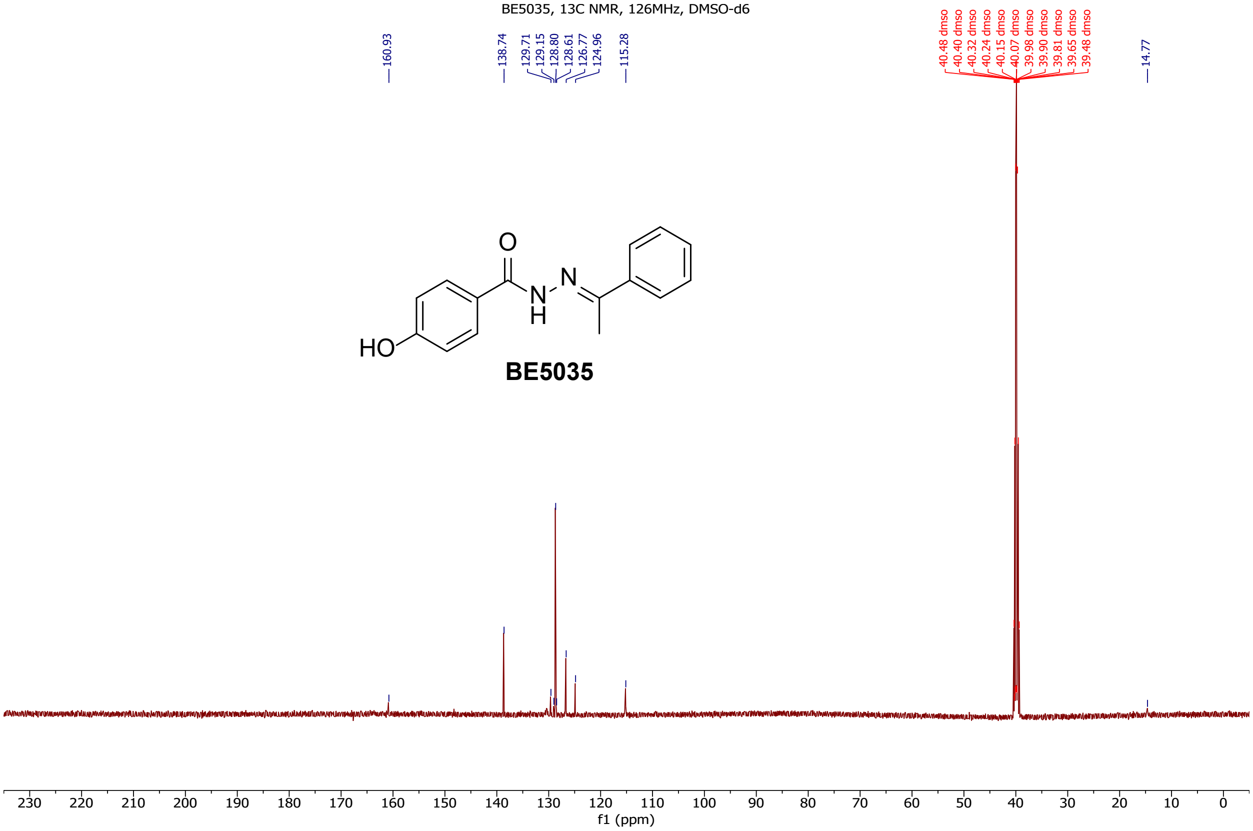

Figure S78. $^{13}C\{^{1}H\}$ NMR spectra of compound BE5035

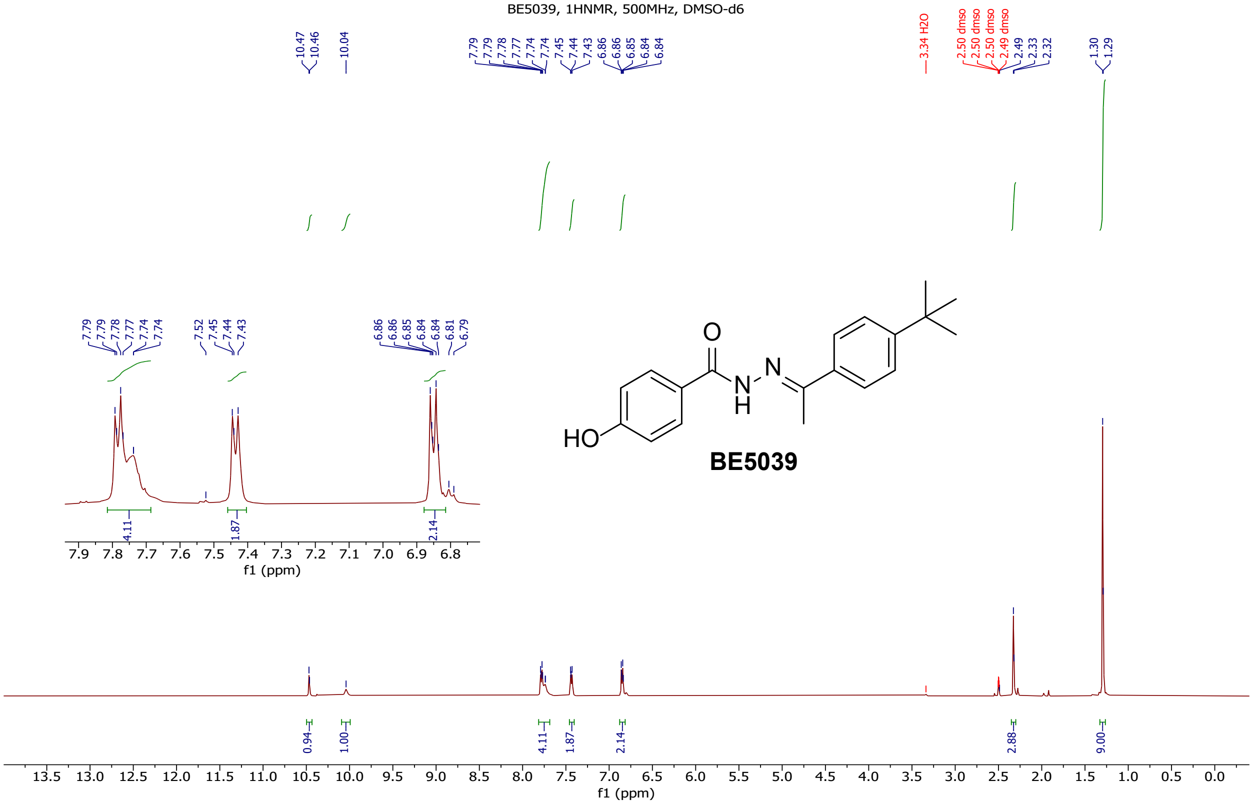

Figure S79. ^{1}H NMR spectra of compound BE5039

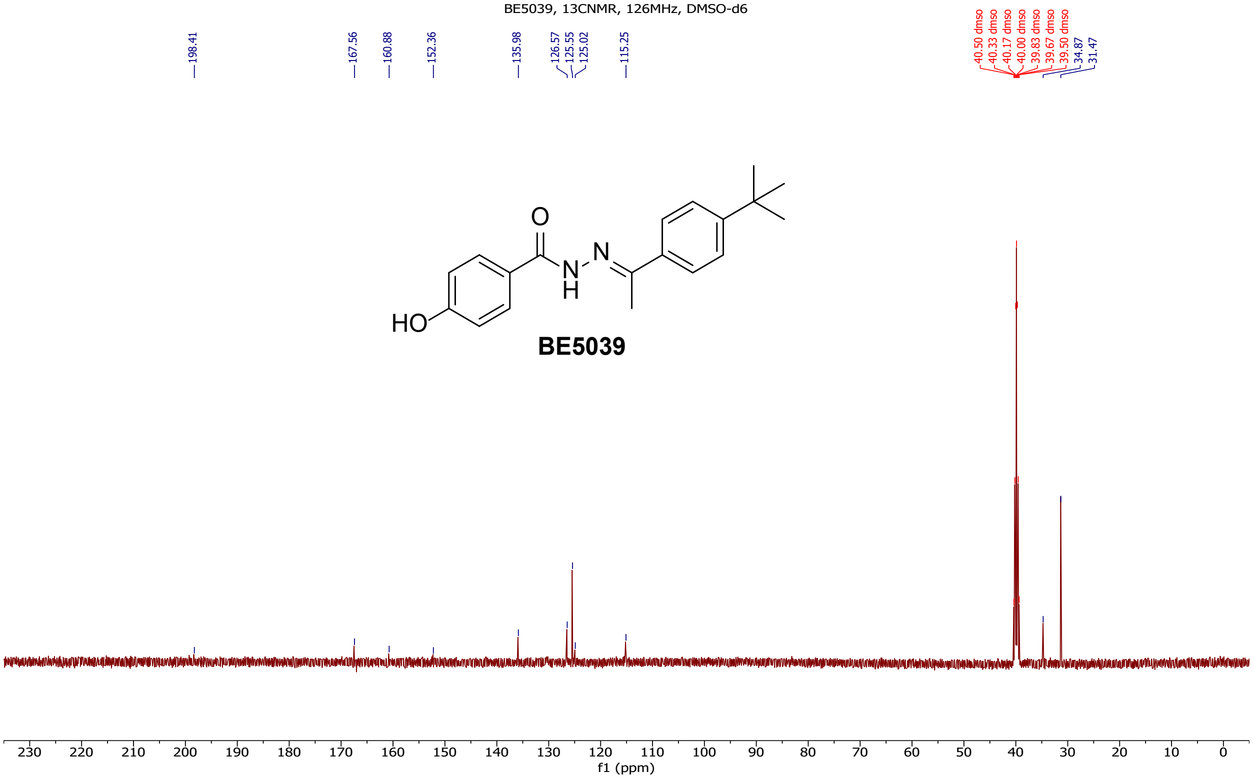

Figure S80. $^{13}C\{^{1}H\}$ NMR spectra of compound BE5039

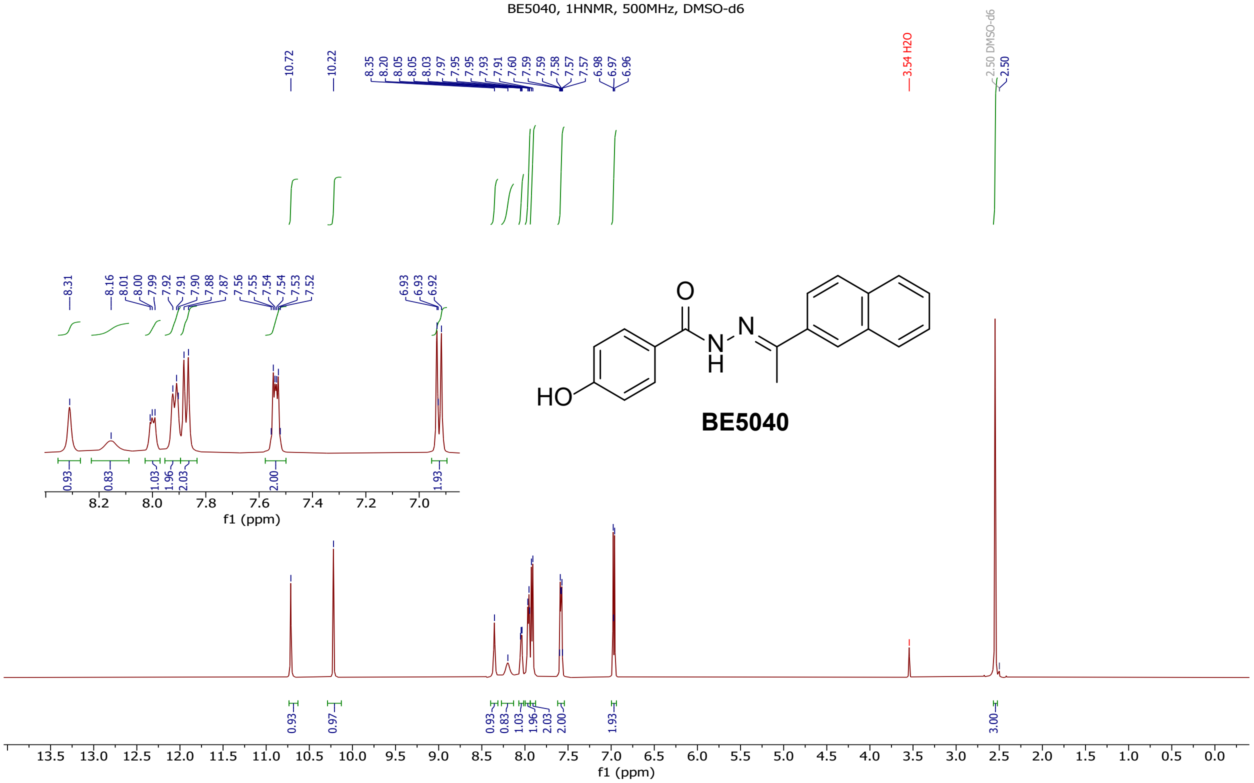

Figure S81. ^{1}H NMR spectra of compound BE5040

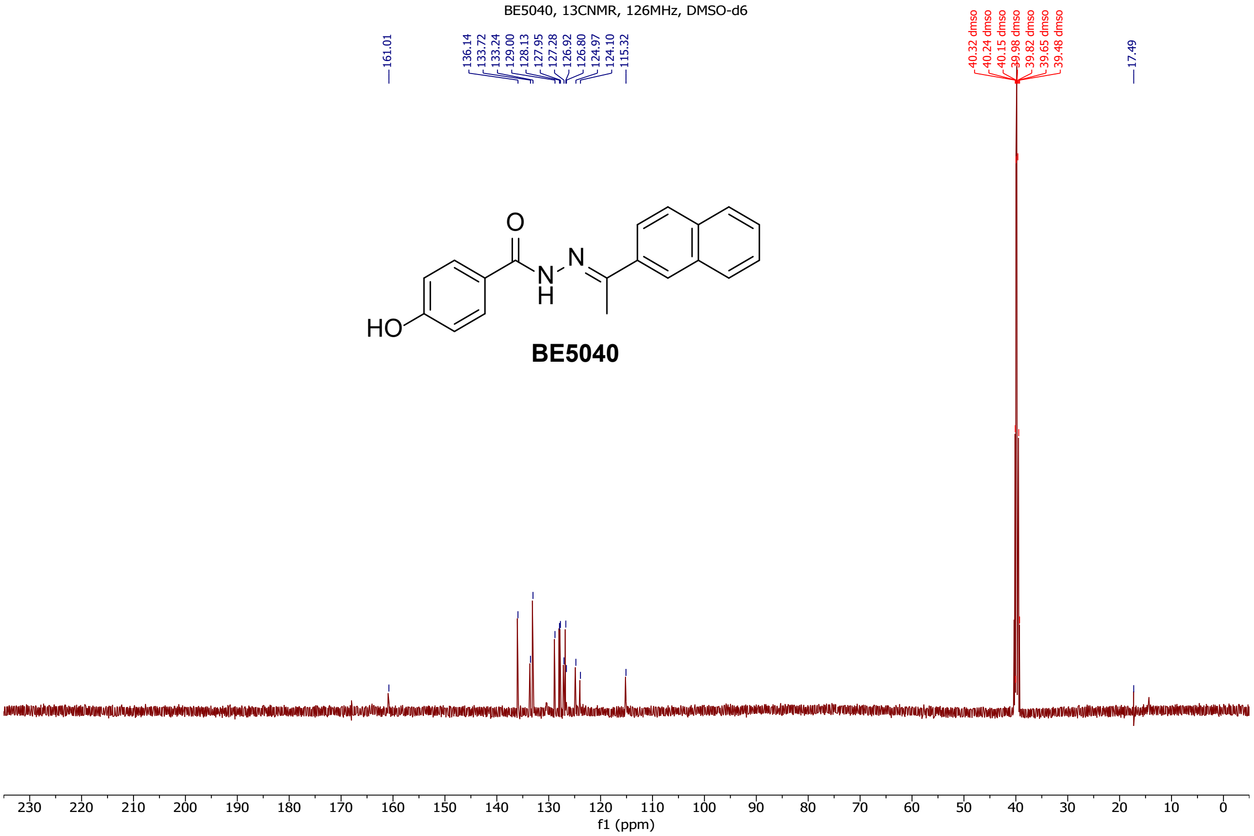

Figure S82. ^{13}C{^{1}H} NMR spectra of compound BE5040

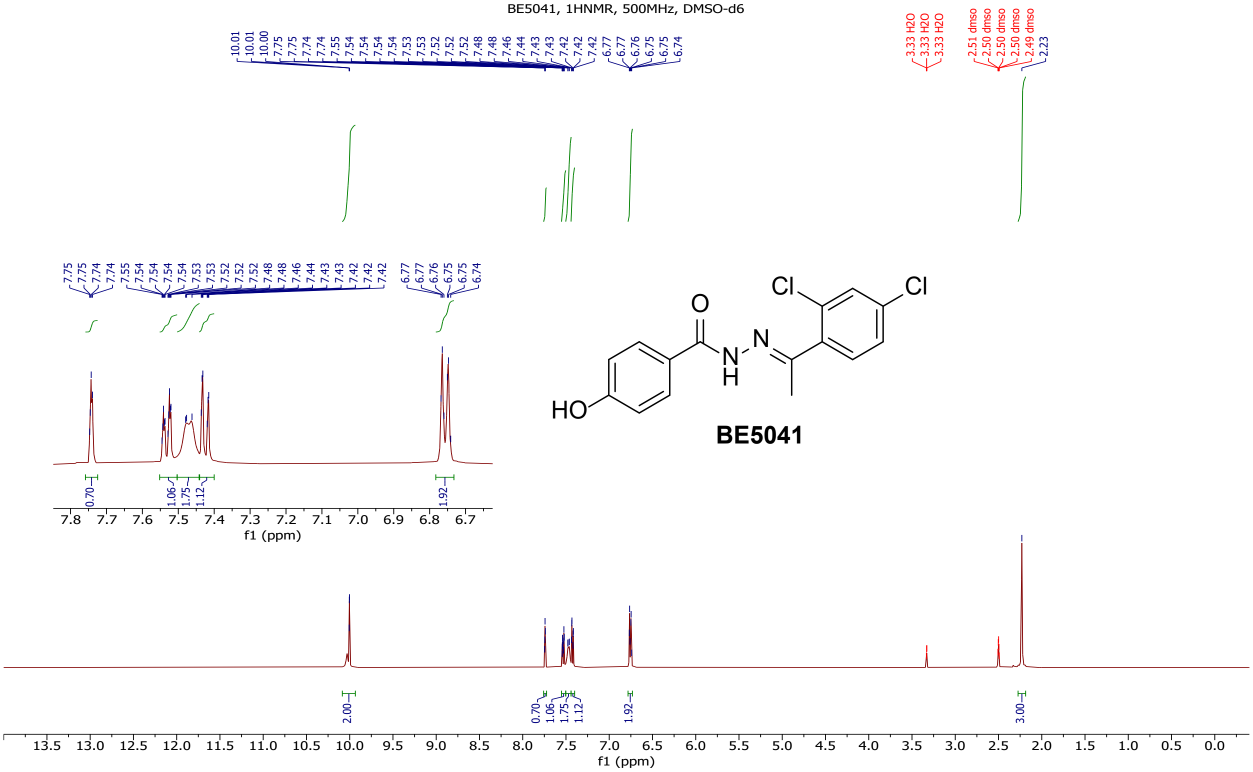

Figure S83. ^{1}H **NMR spectra of compound BE5041**

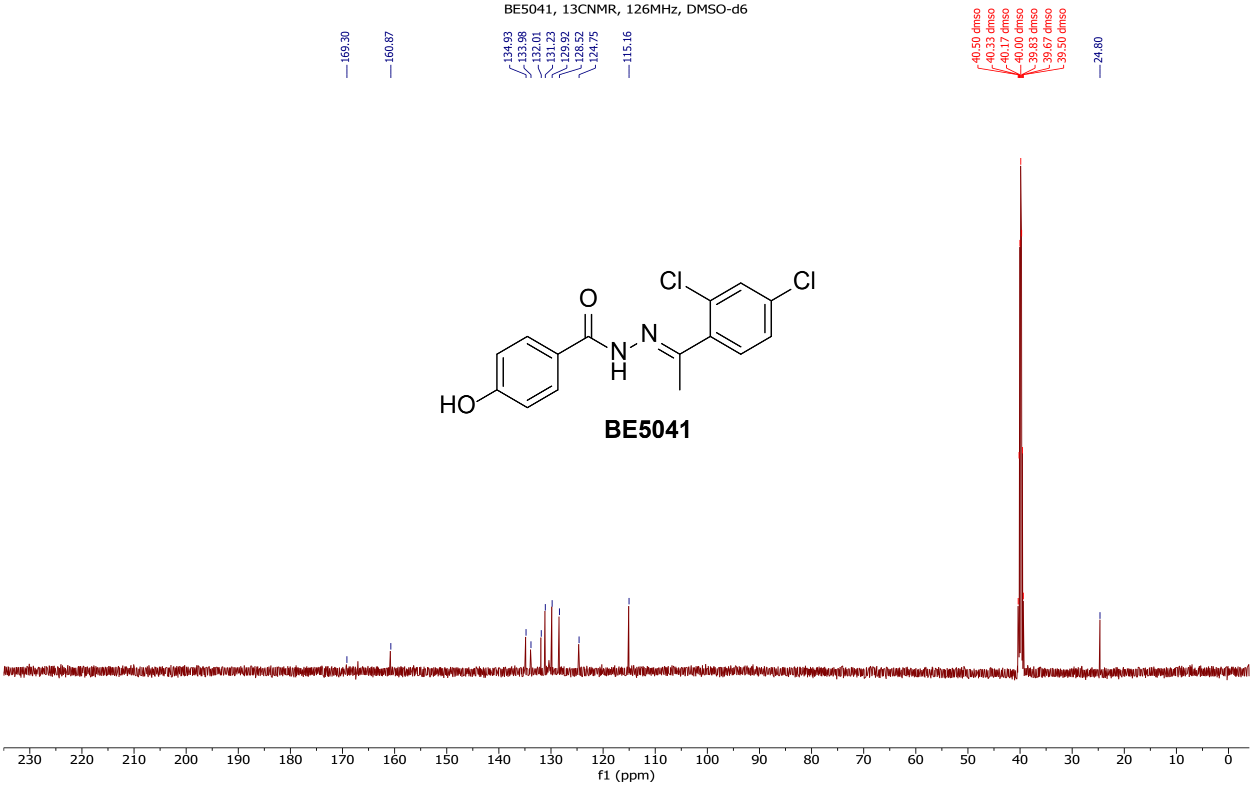

Figure S84. ^{13}C{^{1}H} **NMR spectra of compound BE5041**

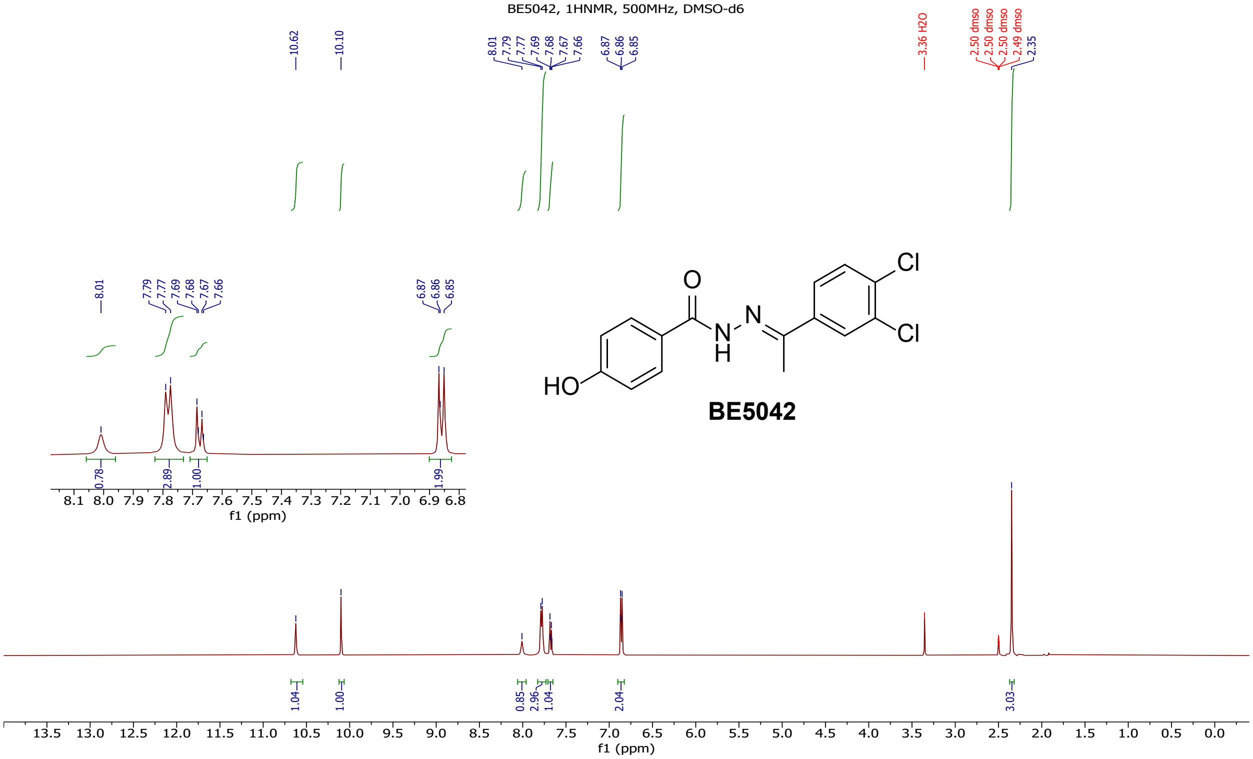

Figure S85. ^{1}H NMR spectra of compound BE5042

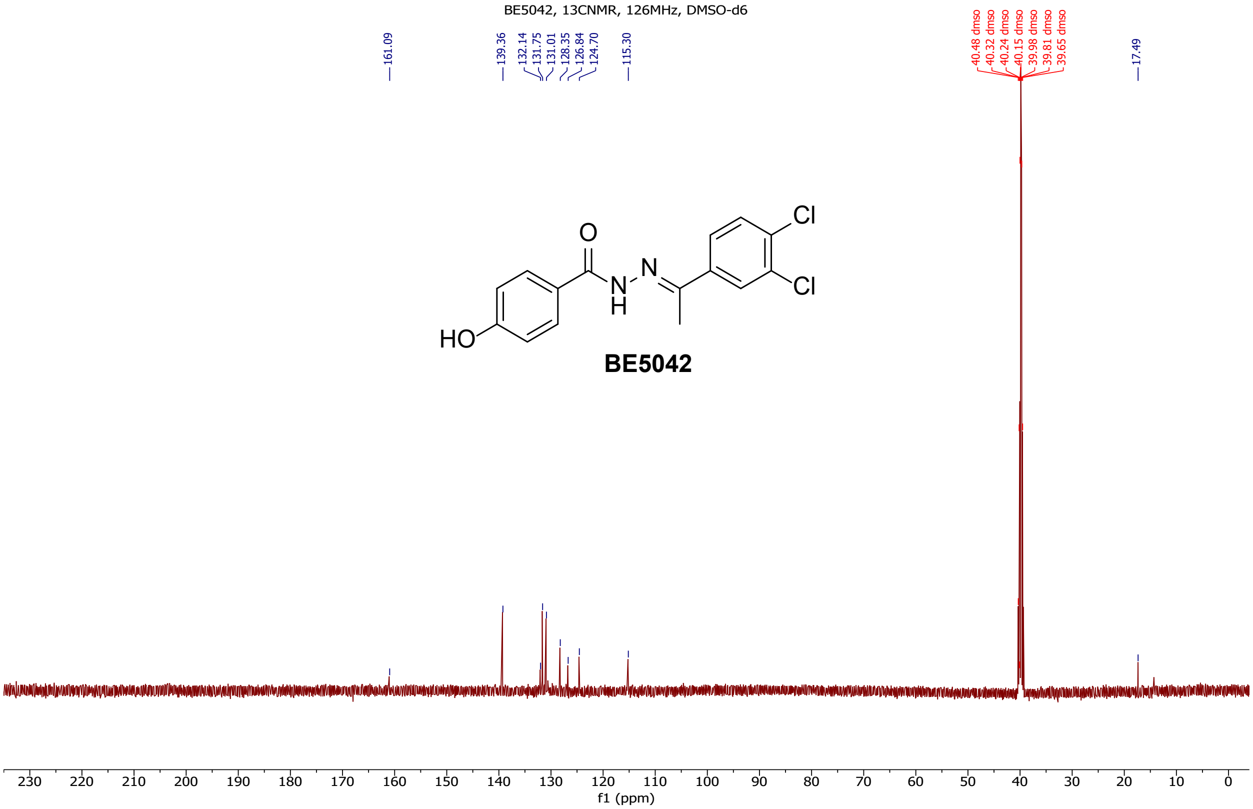

Figure S86. ^{13}C{^{1}H} NMR spectra of compound BE5042

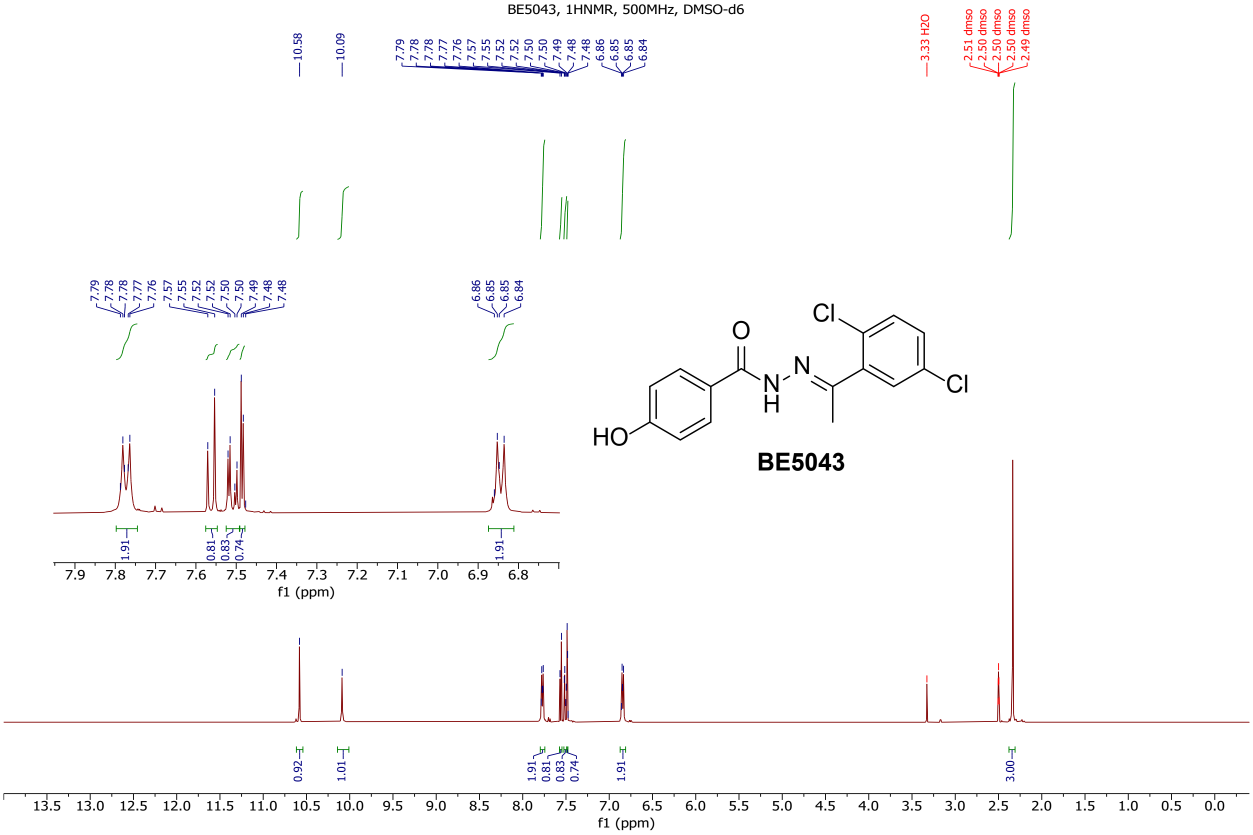

Figure S87. ^{1}**H NMR spectra of compound BE5043**

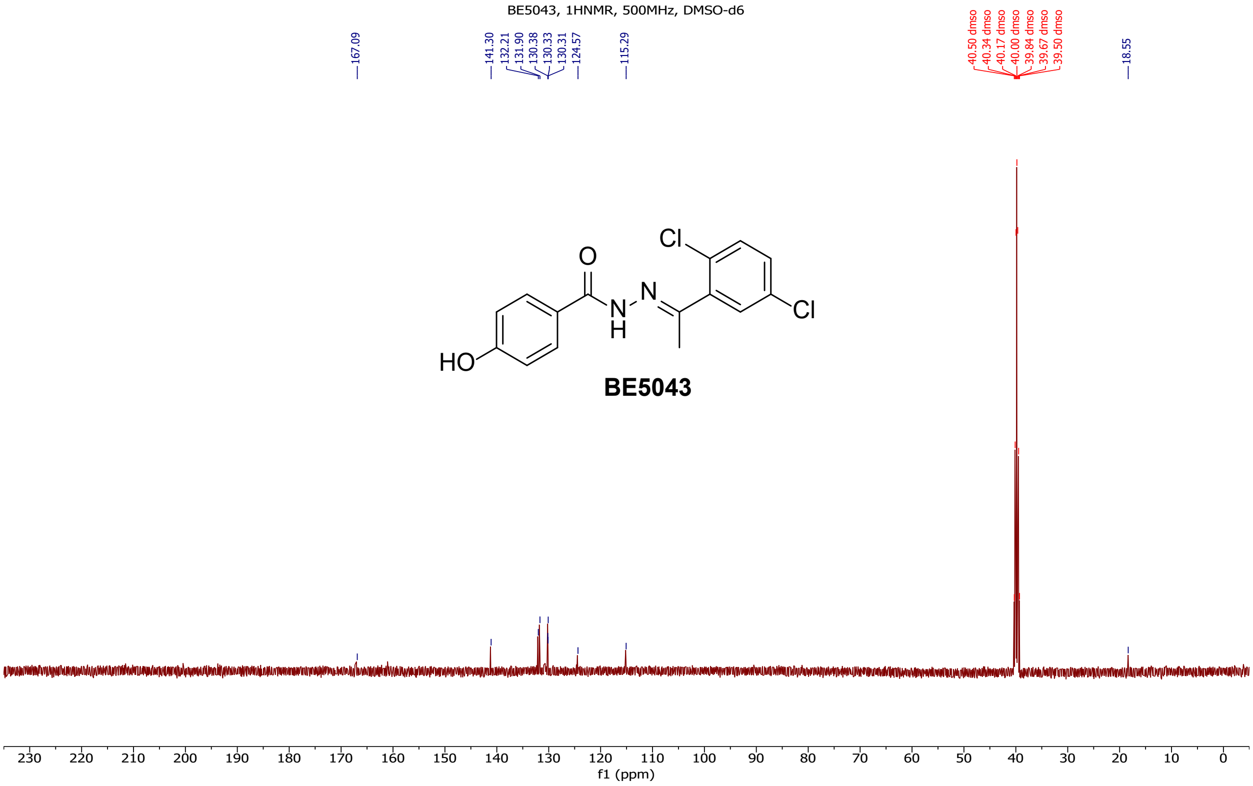

Figure S88. ^{13}**C{**^{1}**H} NMR spectra of compound BE5043**

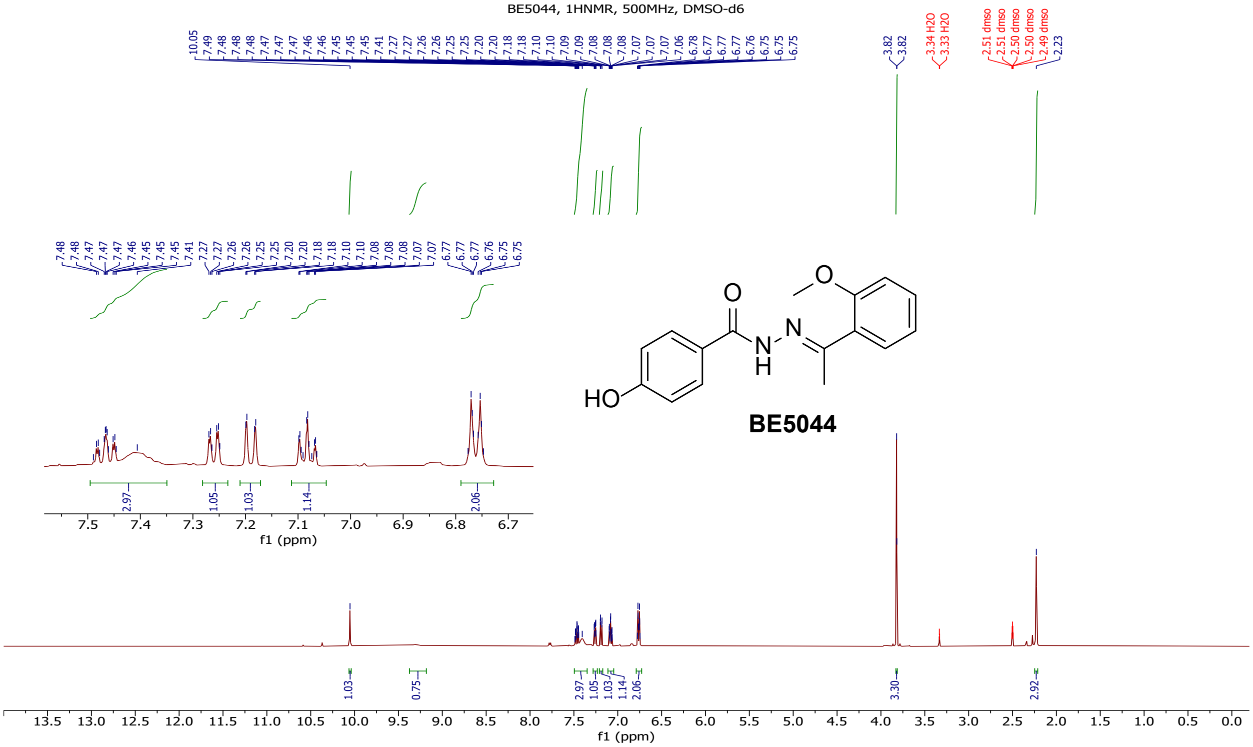

Figure S89. ^{1}H NMR spectra of compound BE5044

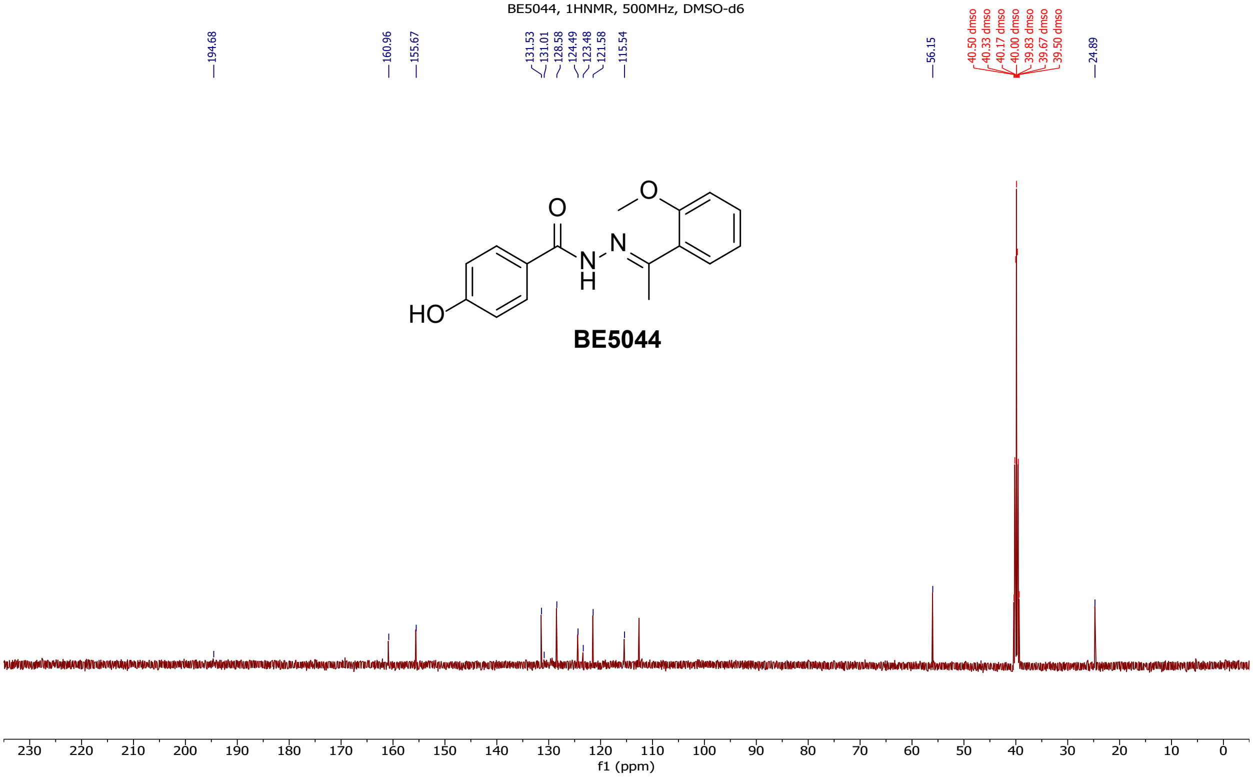

Figure S90. ^{13}C{^{1}H} NMR spectra of compound BE5044

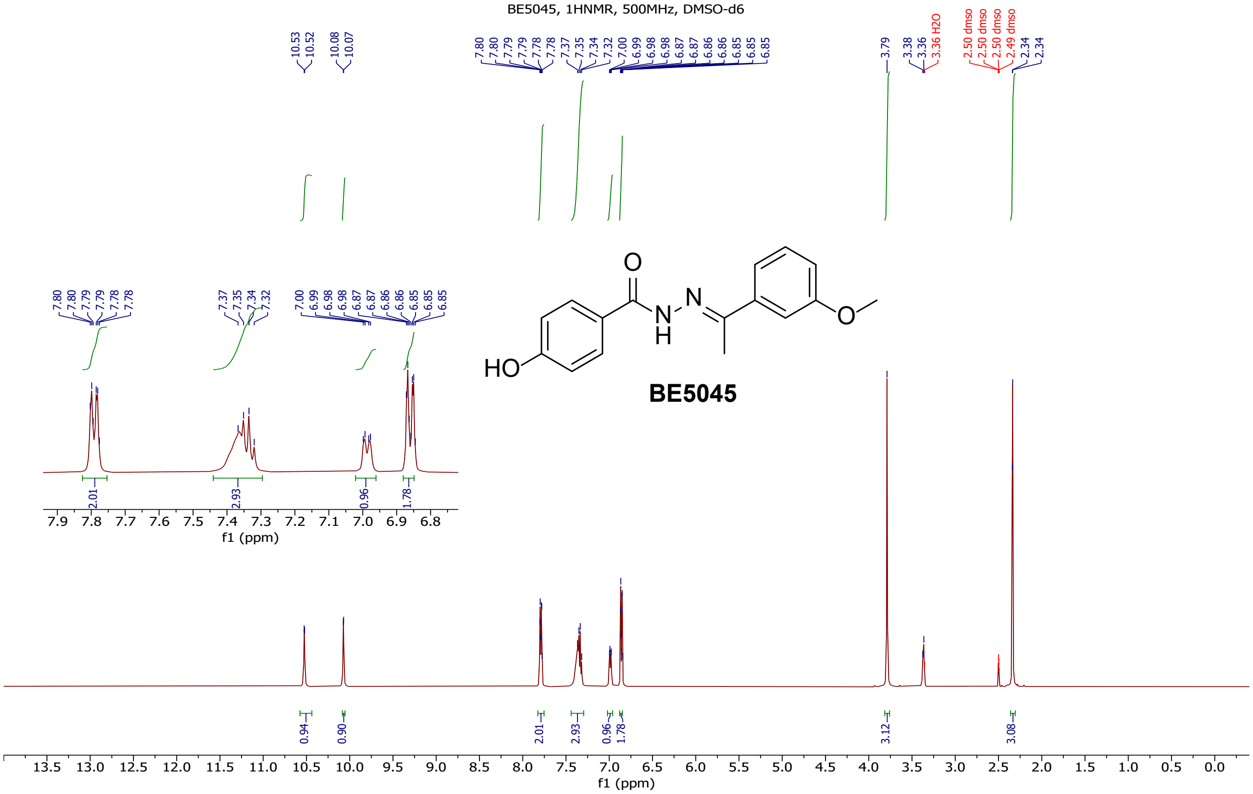

Figure S91. ^{1}H NMR spectra of compound BE5045

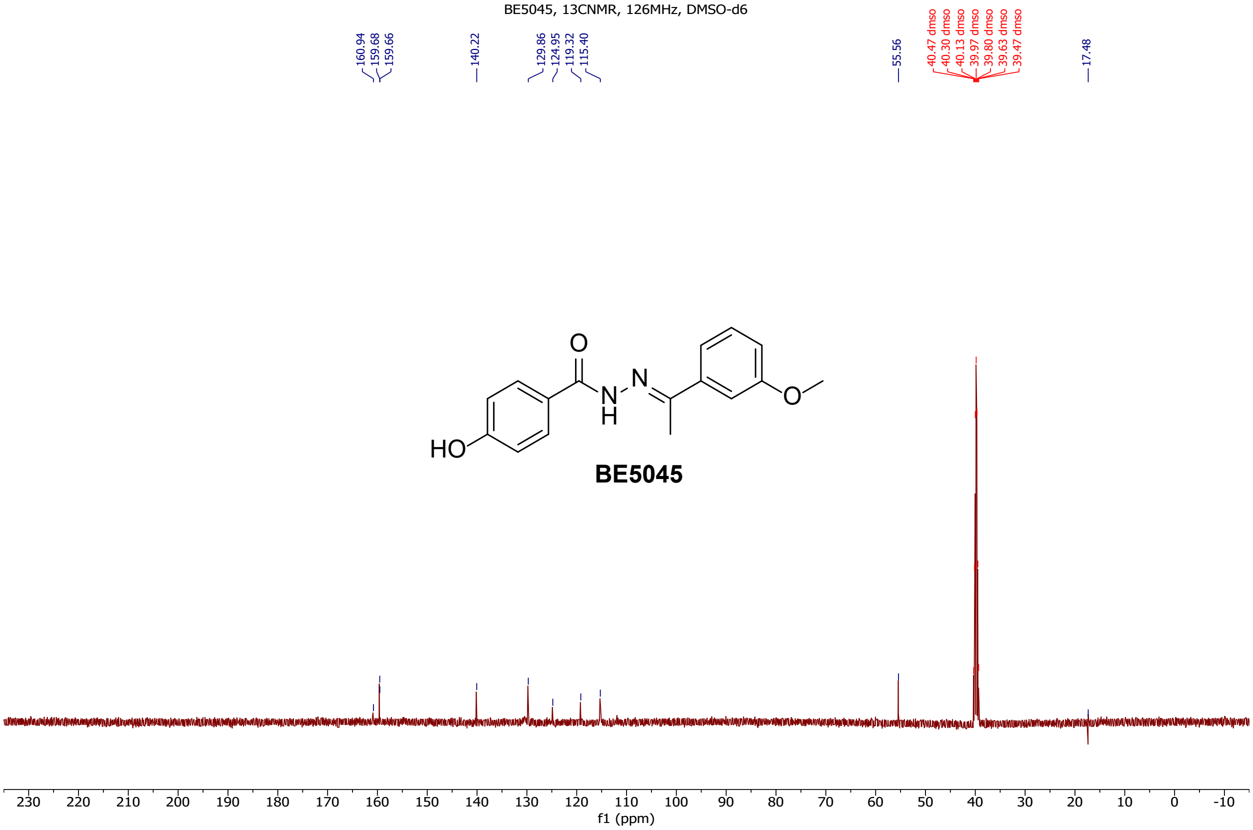

Figure S92. $^{13}C\{^{1}H\}$ NMR spectra of compound BE5045

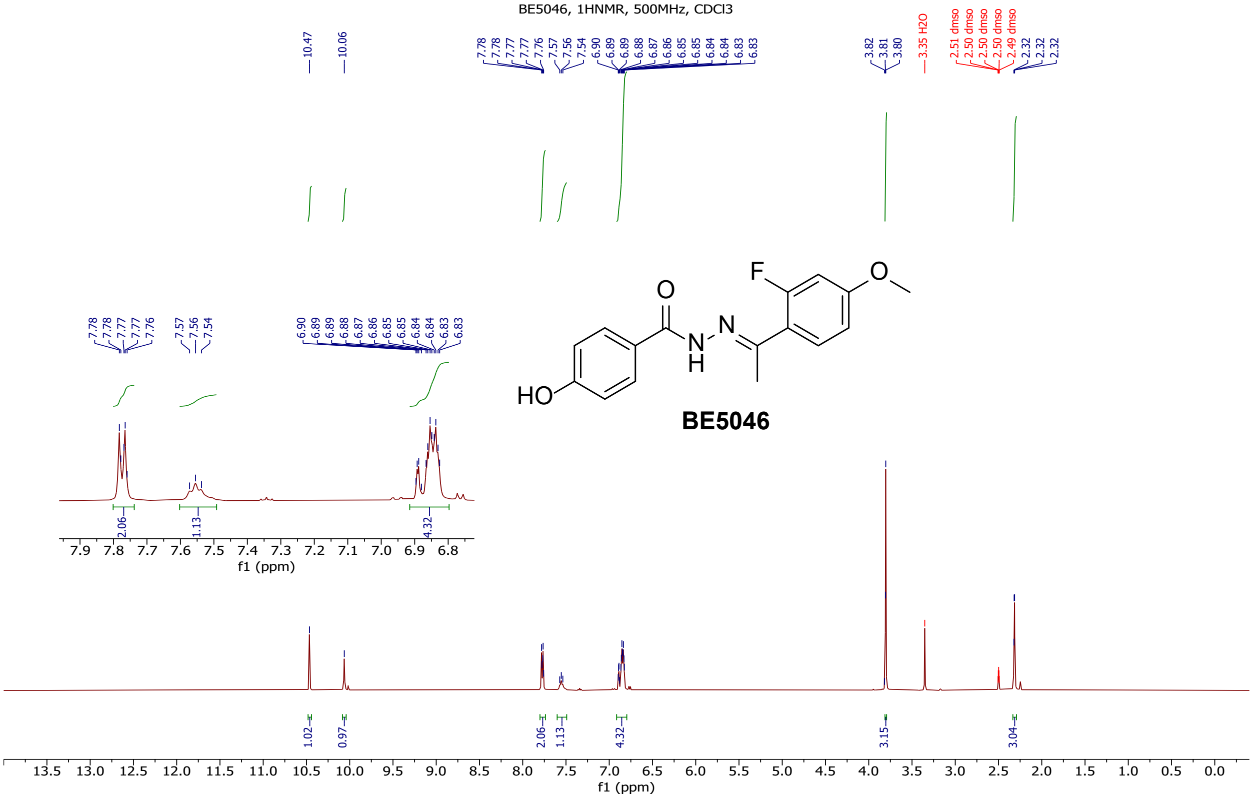

Figure S93. ^{1}H NMR spectra of compound BE5046

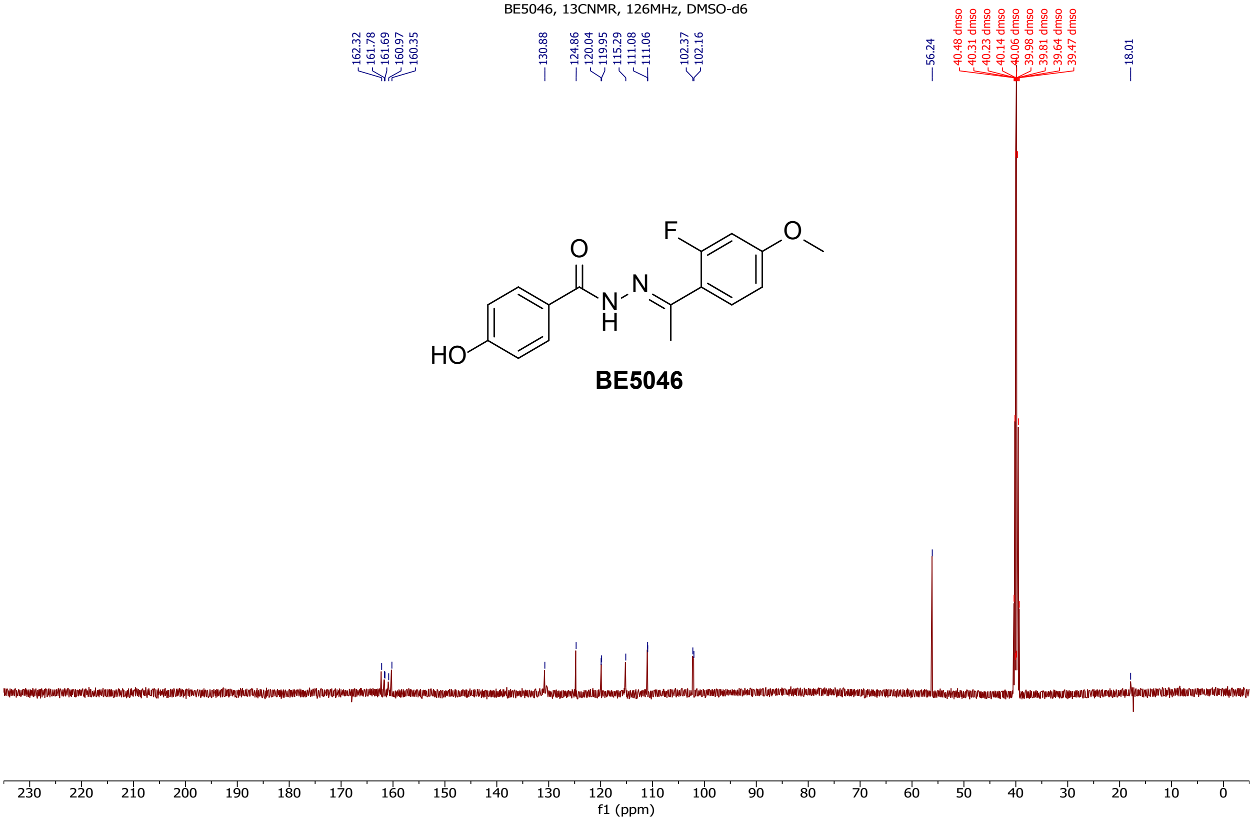

Figure S94. ^{13}C{^{1}H} NMR spectra of compound BE5046

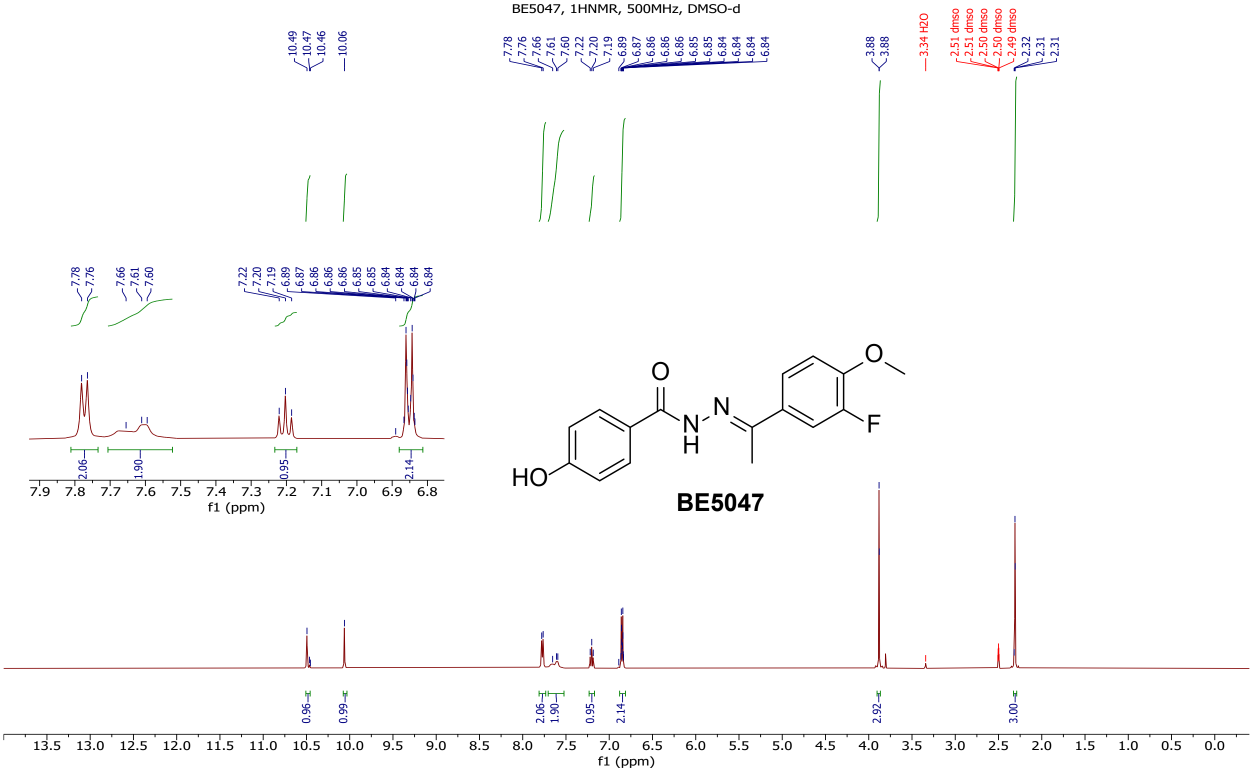

Figure S95. ^{1}H NMR spectra of compound BE5047

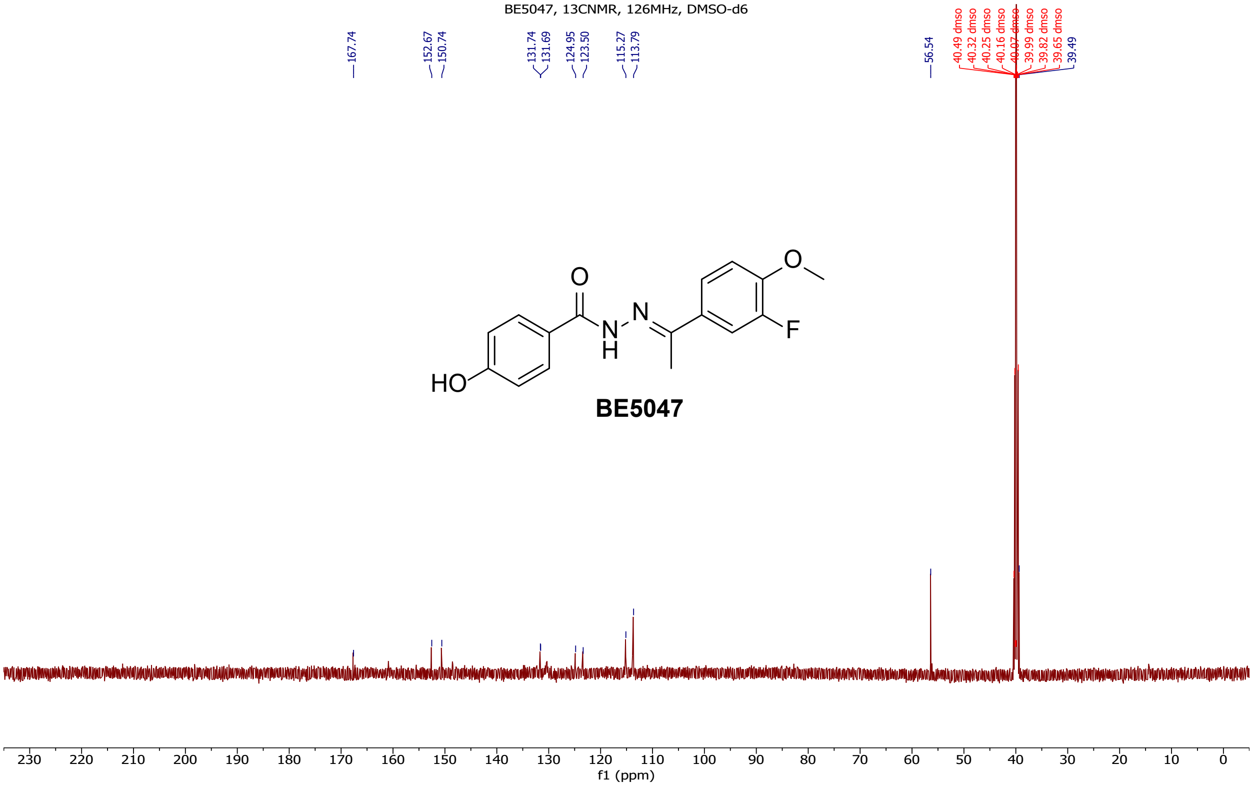

Figure S96. $^{13}C\{^{1}H\}$ NMR spectra of compound BE5047

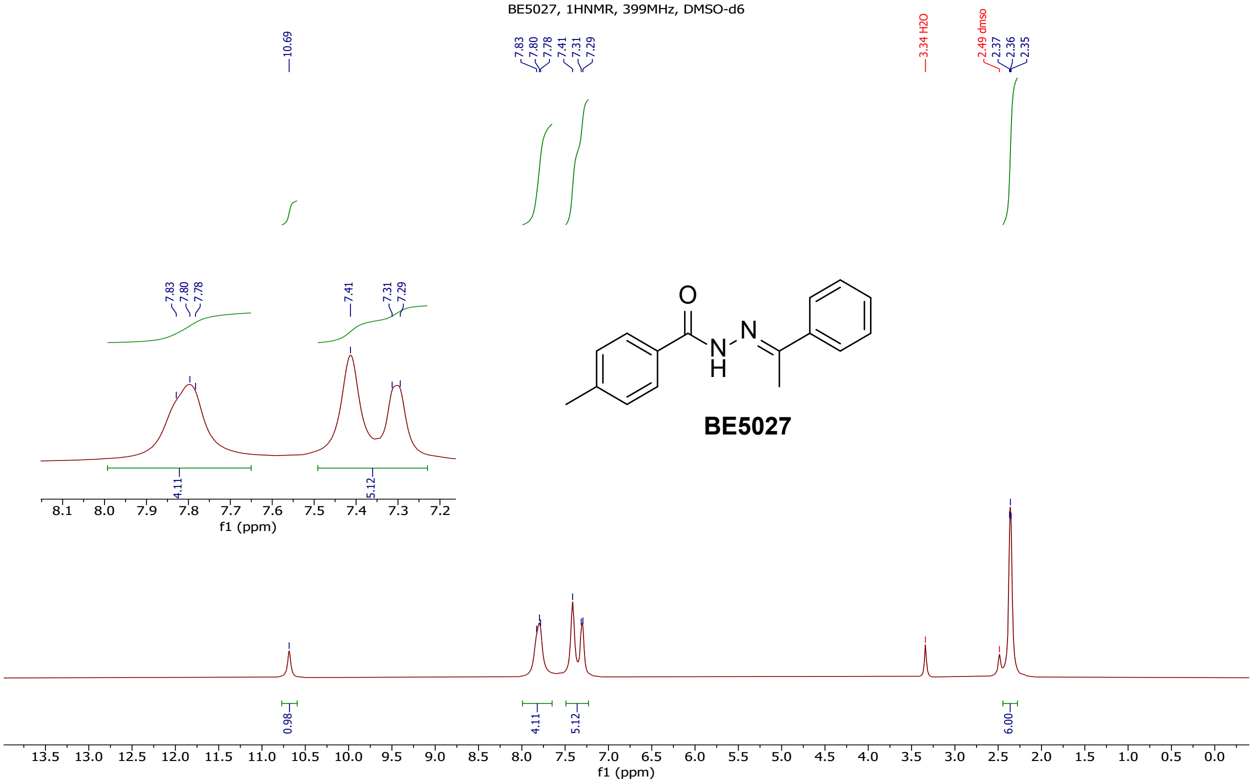

Figure S97. ^{1}H NMR spectra of compound BE5027

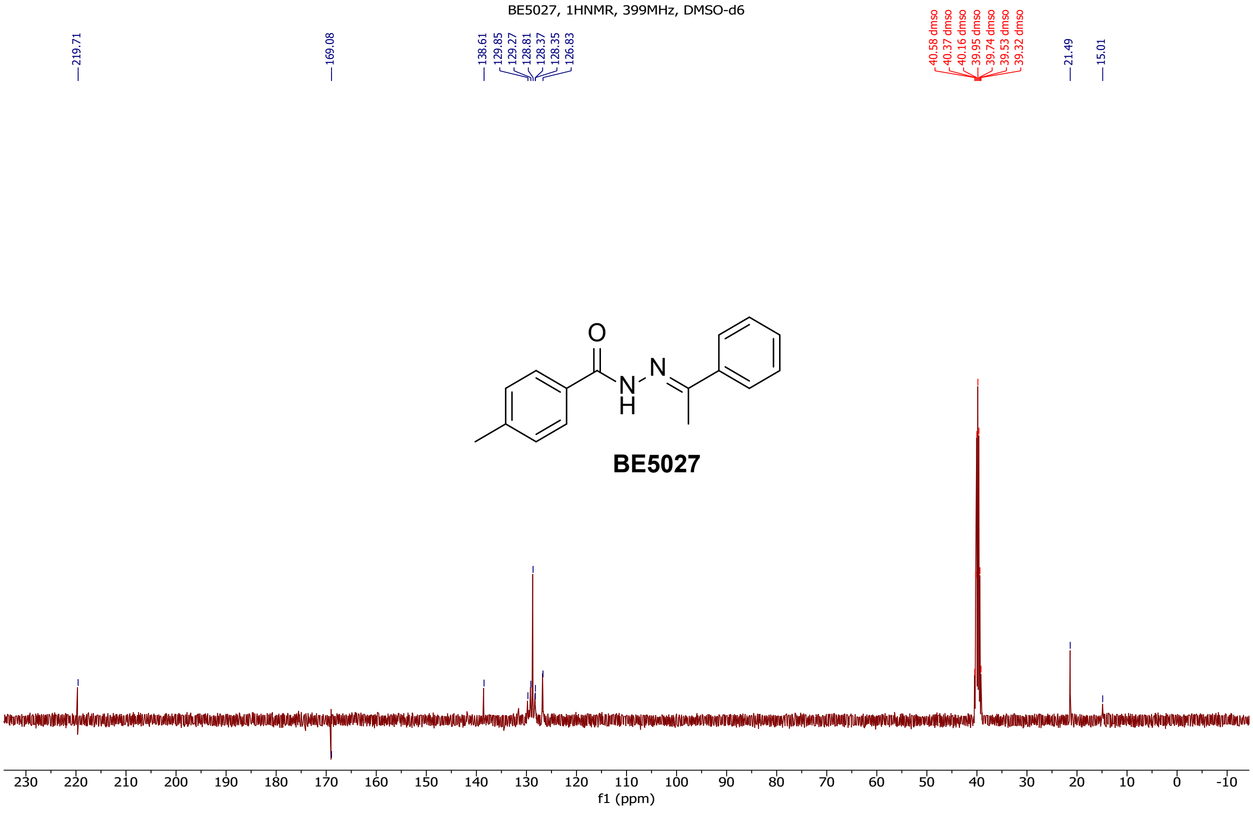

Figure S98. ^{13}C{^{1}H} NMR spectra of compound BE5027

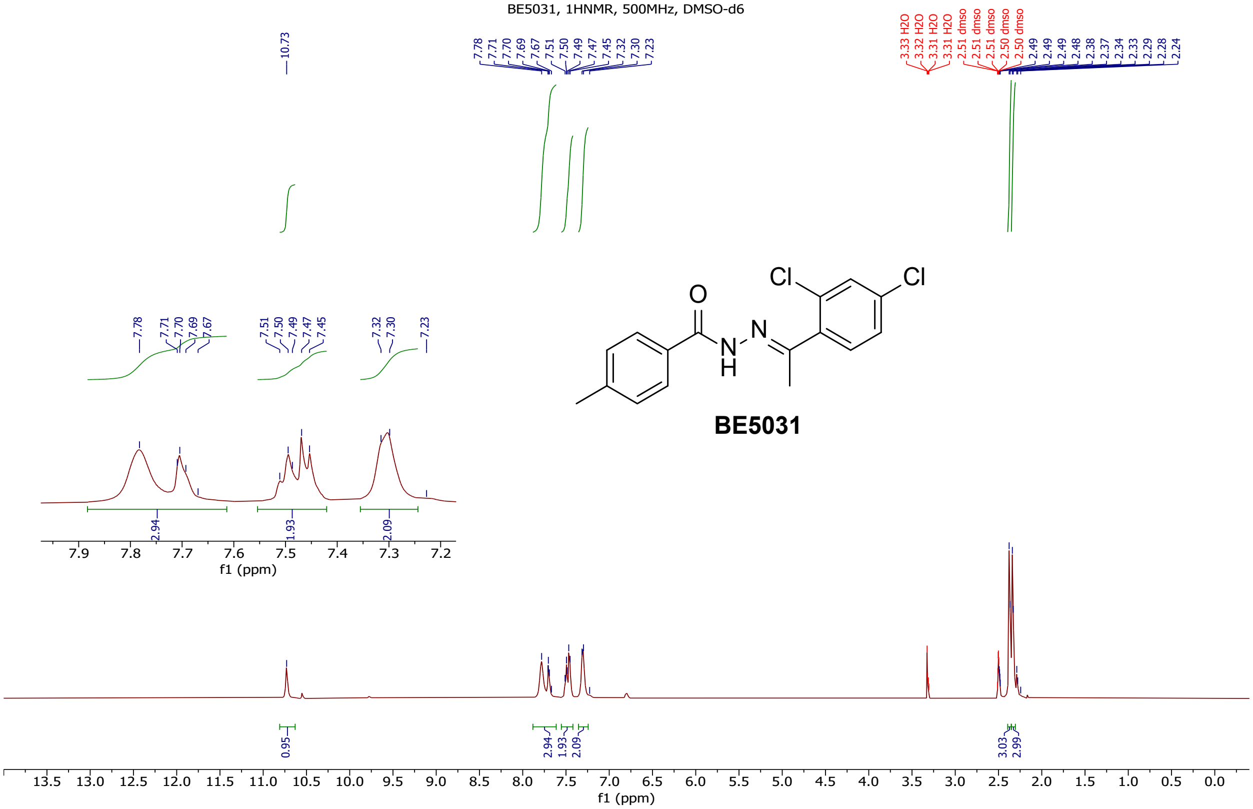

Figure S99. ^{1}H NMR spectra of compound BE5031

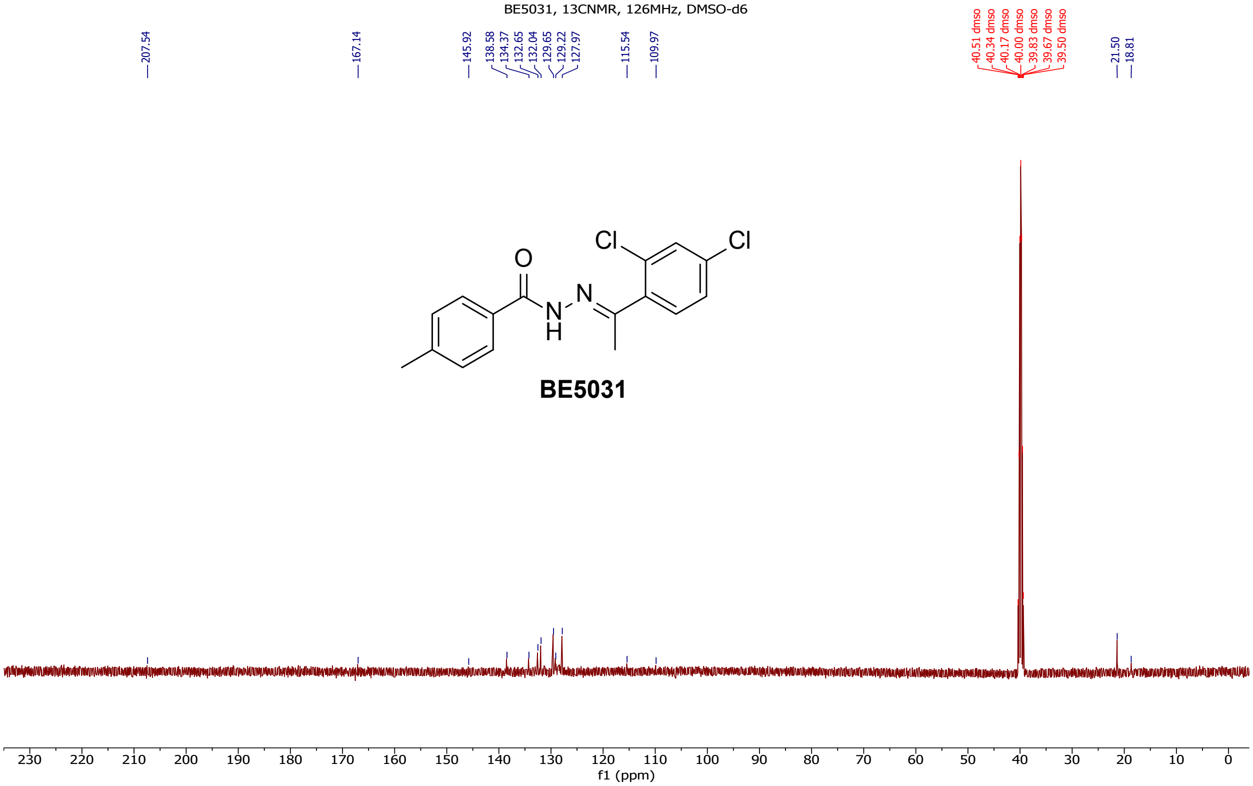

Figure S100. $^{13}C\{^{1}H\}$ NMR spectra of compound BE5031

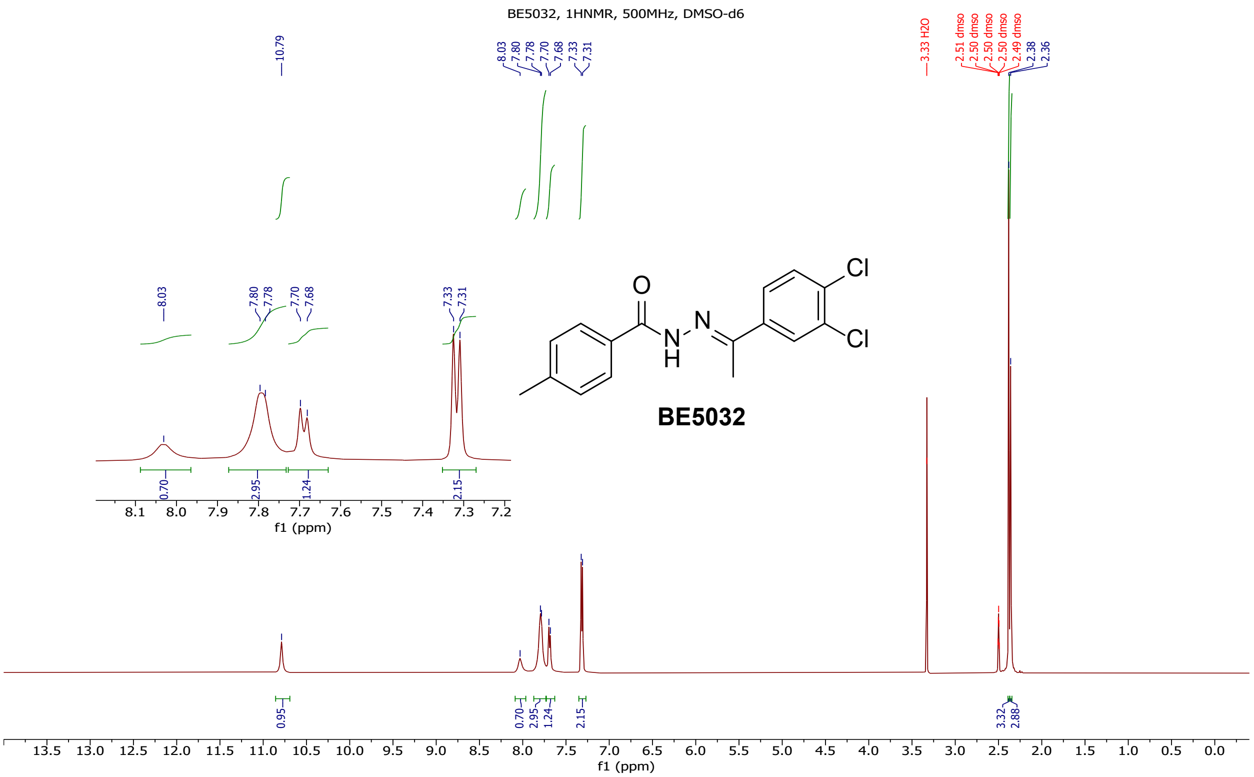

Figure S101. ^{1}H NMR spectra of compound BE5032

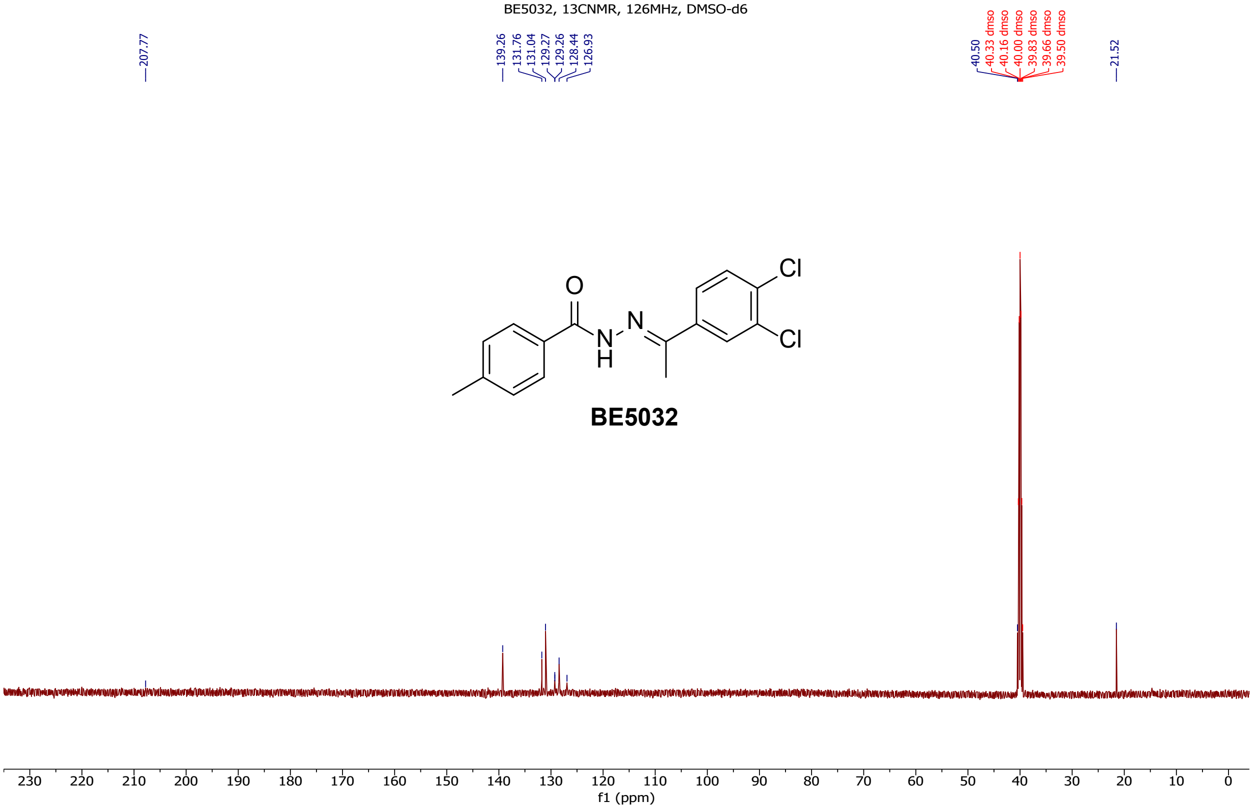

Figure S102. ^{13}C{^{1}H} NMR spectra of compound BE5032

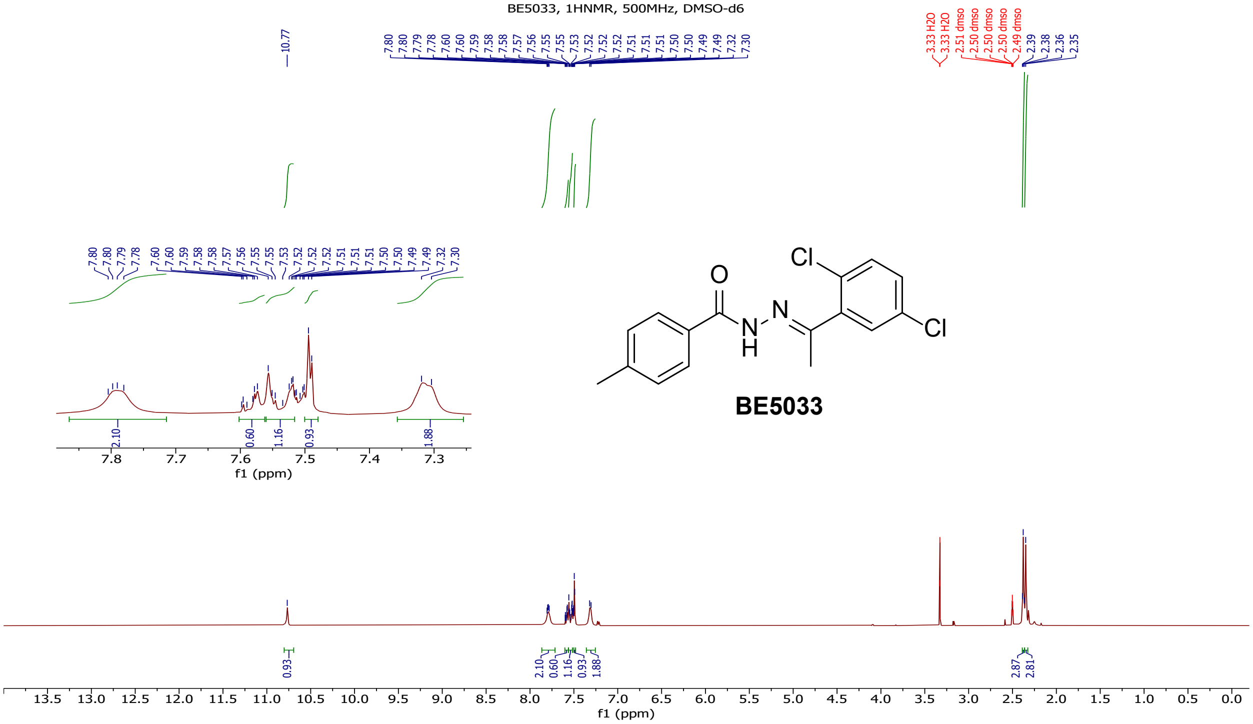

Figure S103. 1H NMR spectra of compound BE5033

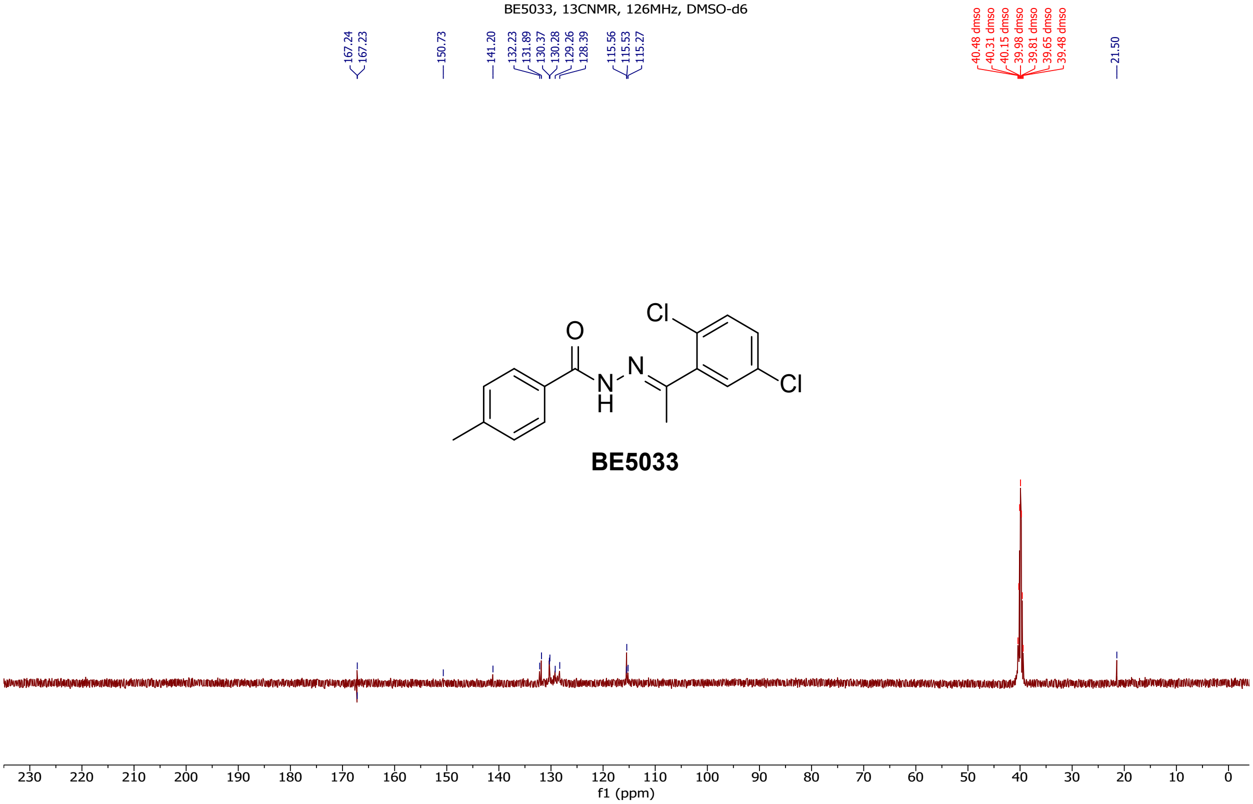

Figure S104. $^{13}C\{^{1}H\}$ NMR spectra of compound BE5033

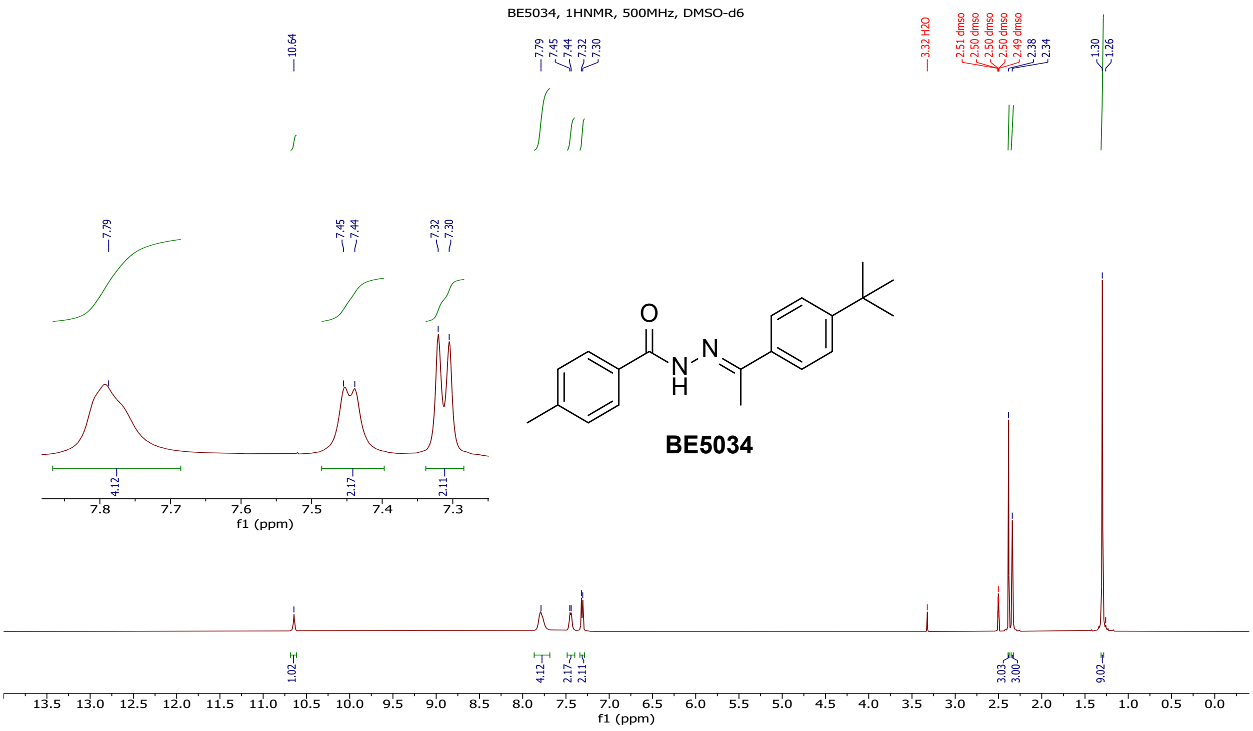

Figure S105. ^{1}H NMR spectra of compound BE5034

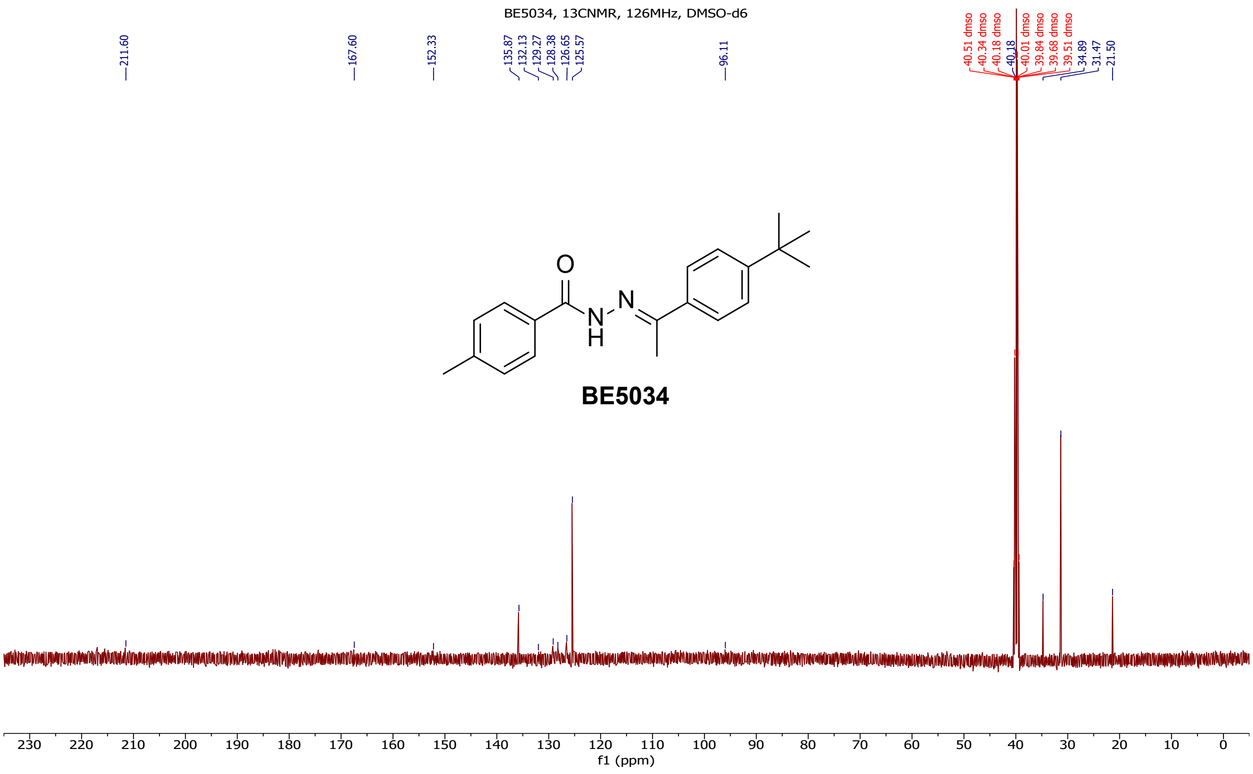

Figure S106. $^{13}C\{^{1}H\}$ NMR spectra of compound BE5034

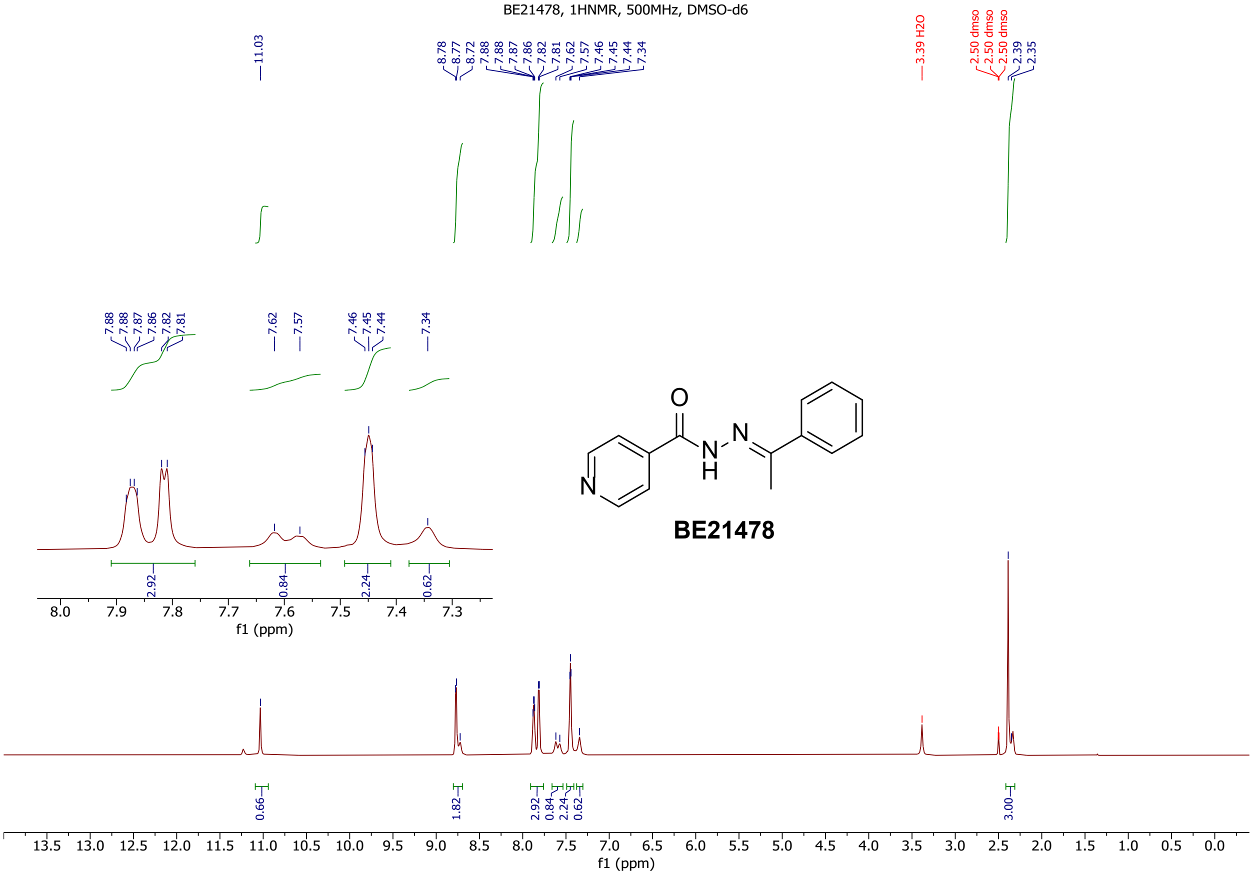

Figure S107. ^{1}H NMR spectra of compound BE21478

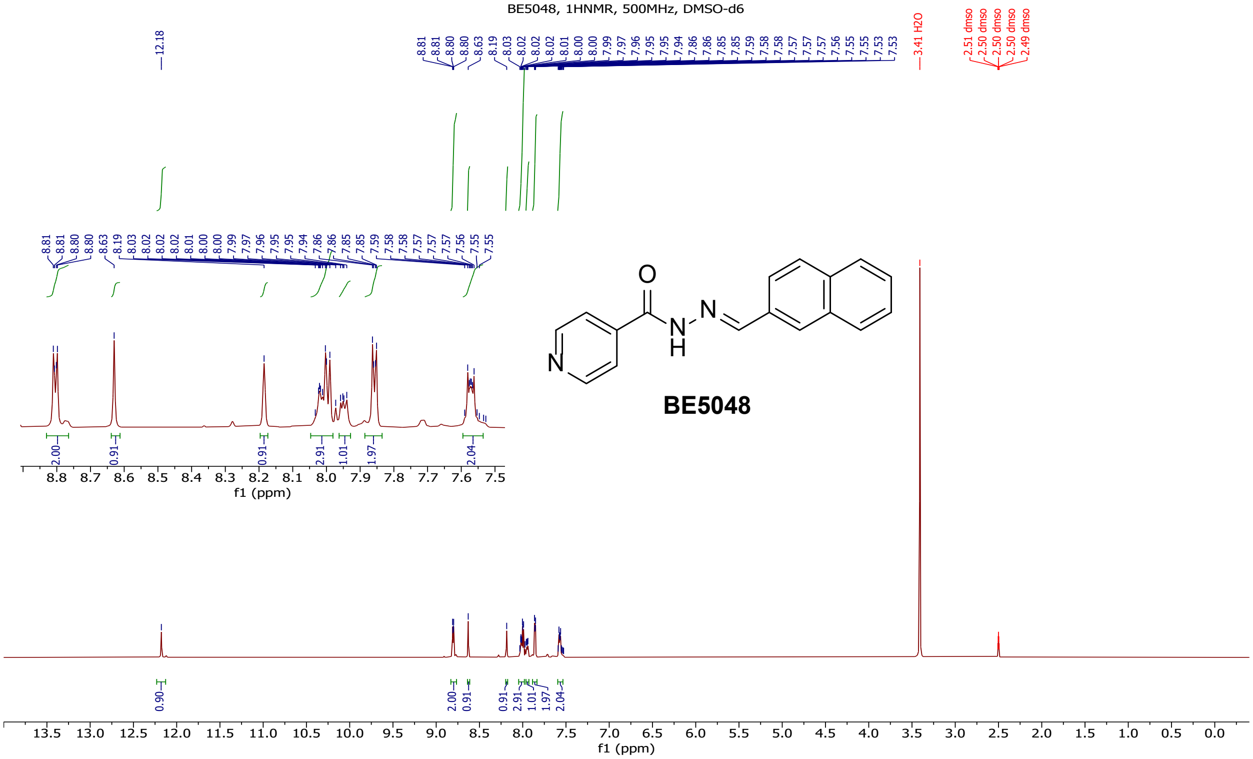

Figure S108. ^{1}H NMR spectra of compound BE5048

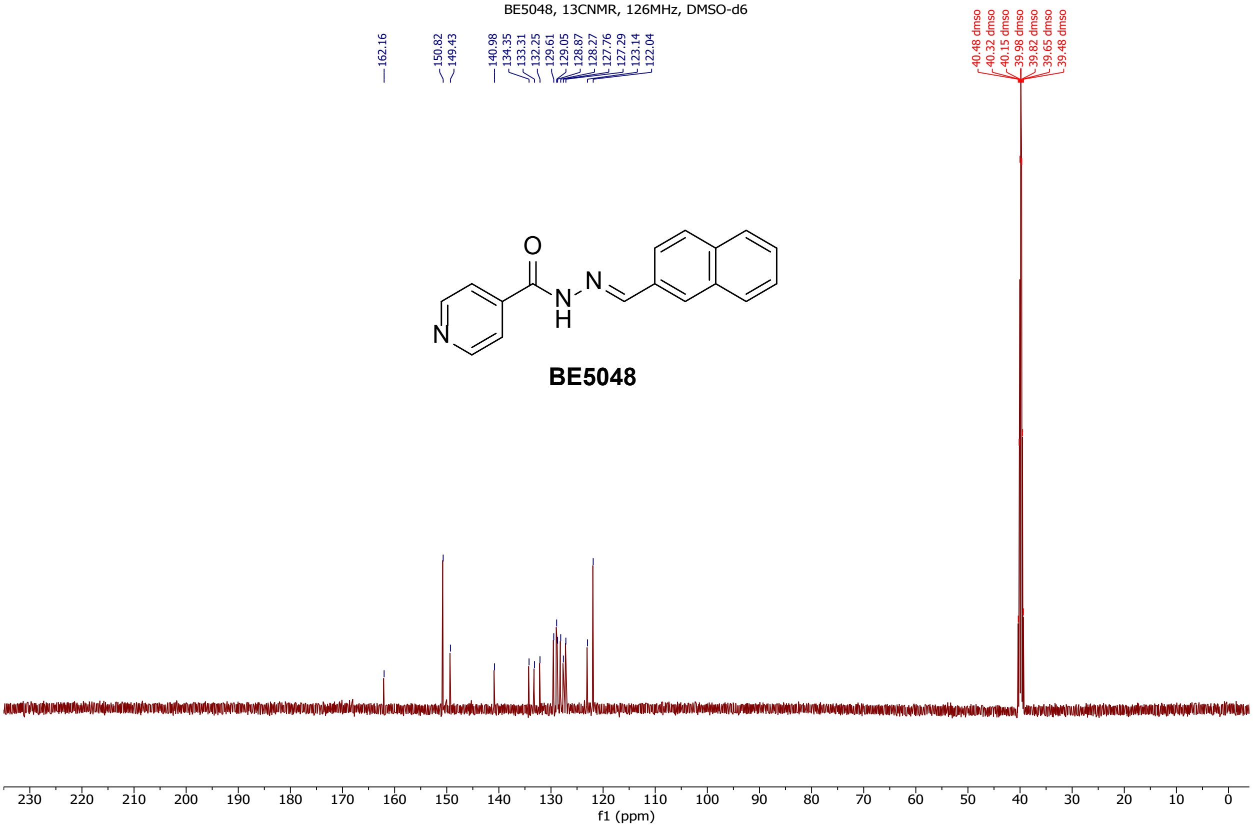

Figure S109. $^{13}C\{^{1}H\}$ NMR spectra of compound BE5048

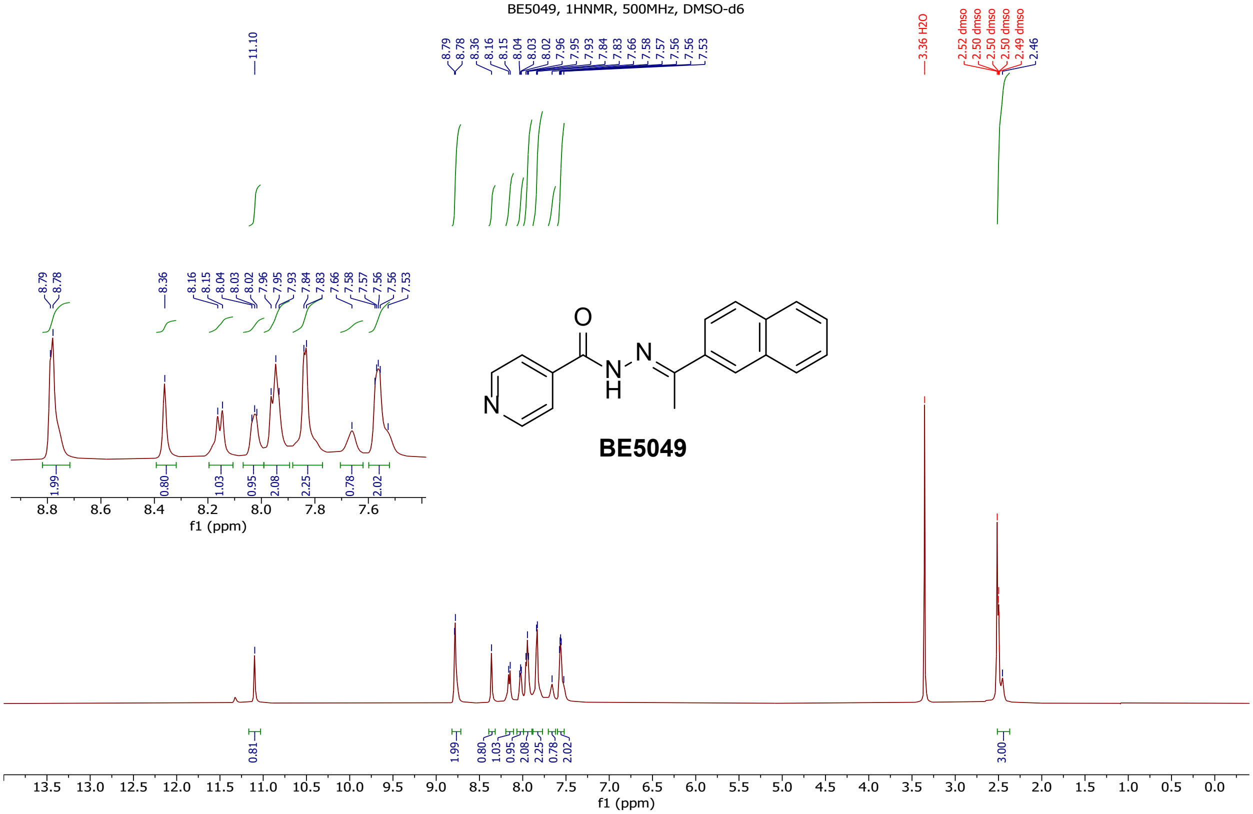

Figure S110. ^{1}H NMR spectra of compound BE5049

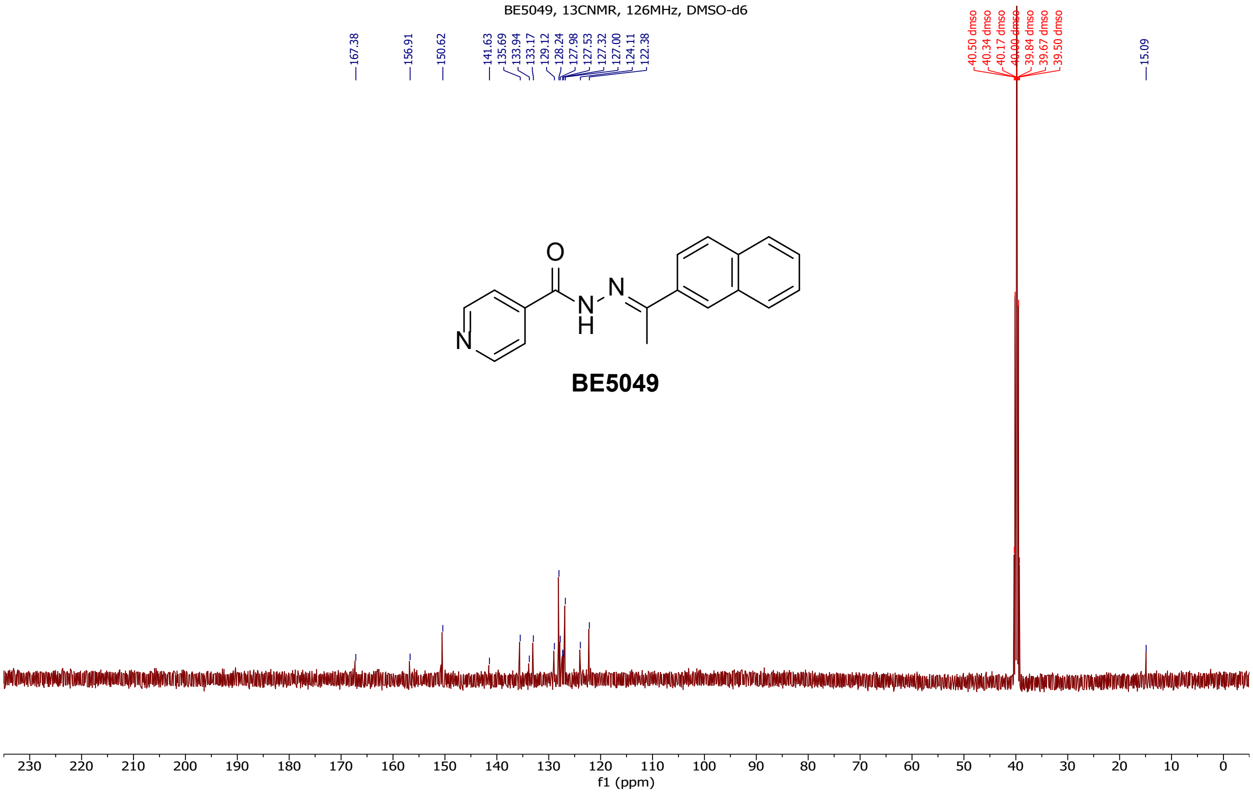

Figure S111. $^{13}C\{^{1}H\}$ NMR spectra of compound BE5049

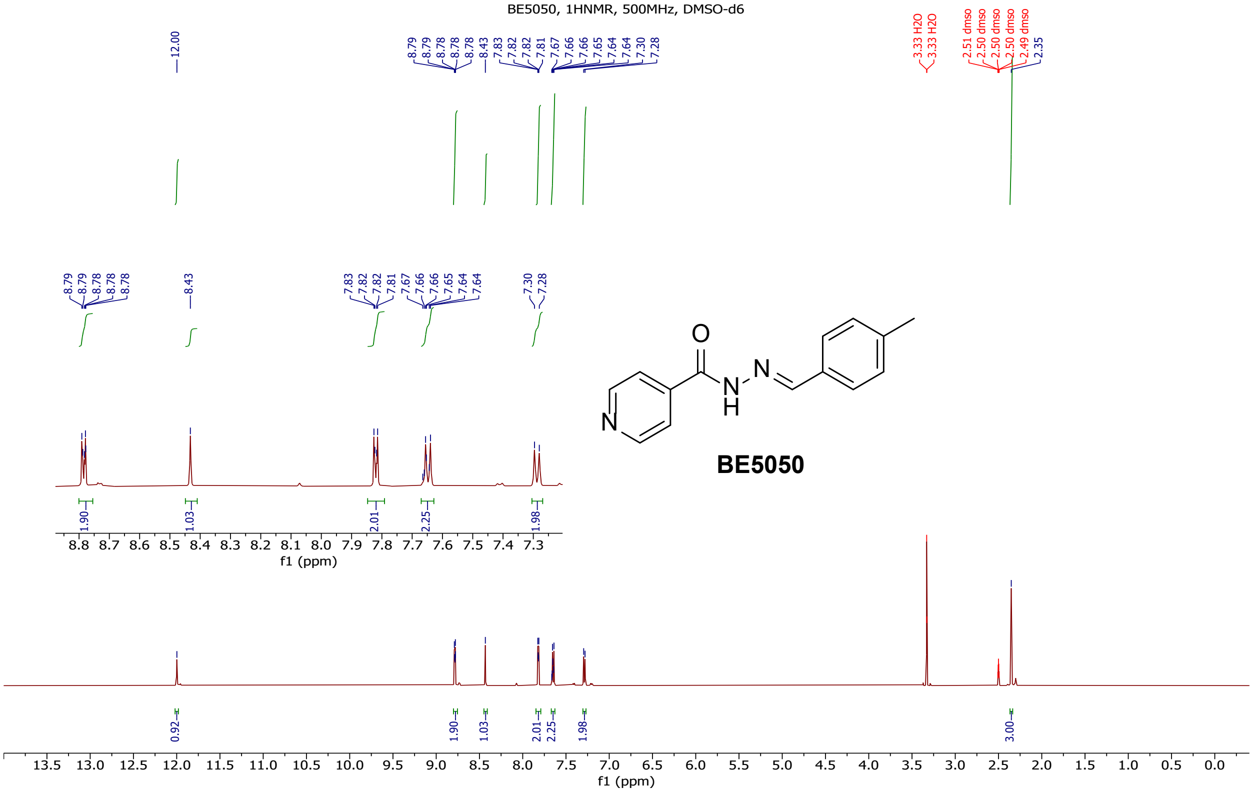

Figure S112. ^{1}H NMR spectra of compound BE5050

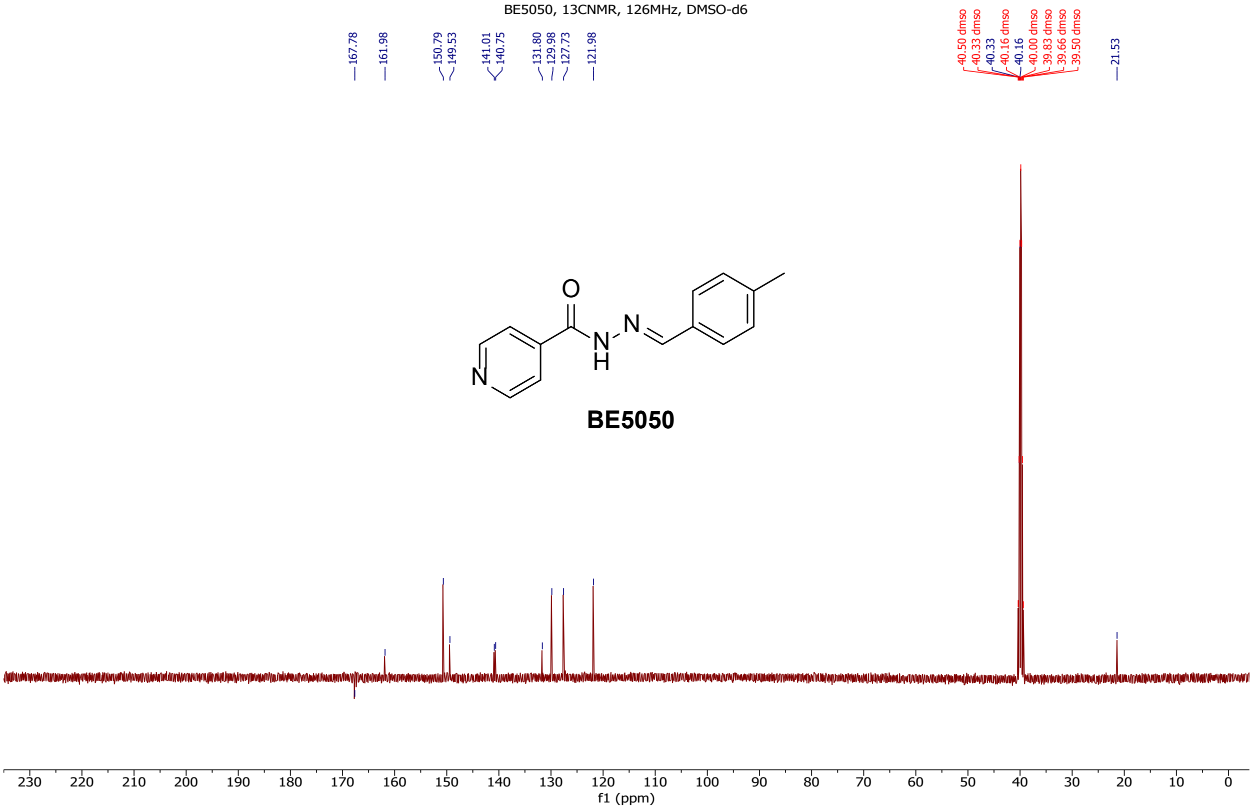

Figure S113. ^{13}C{^{1}H} NMR spectra of compound BE5050

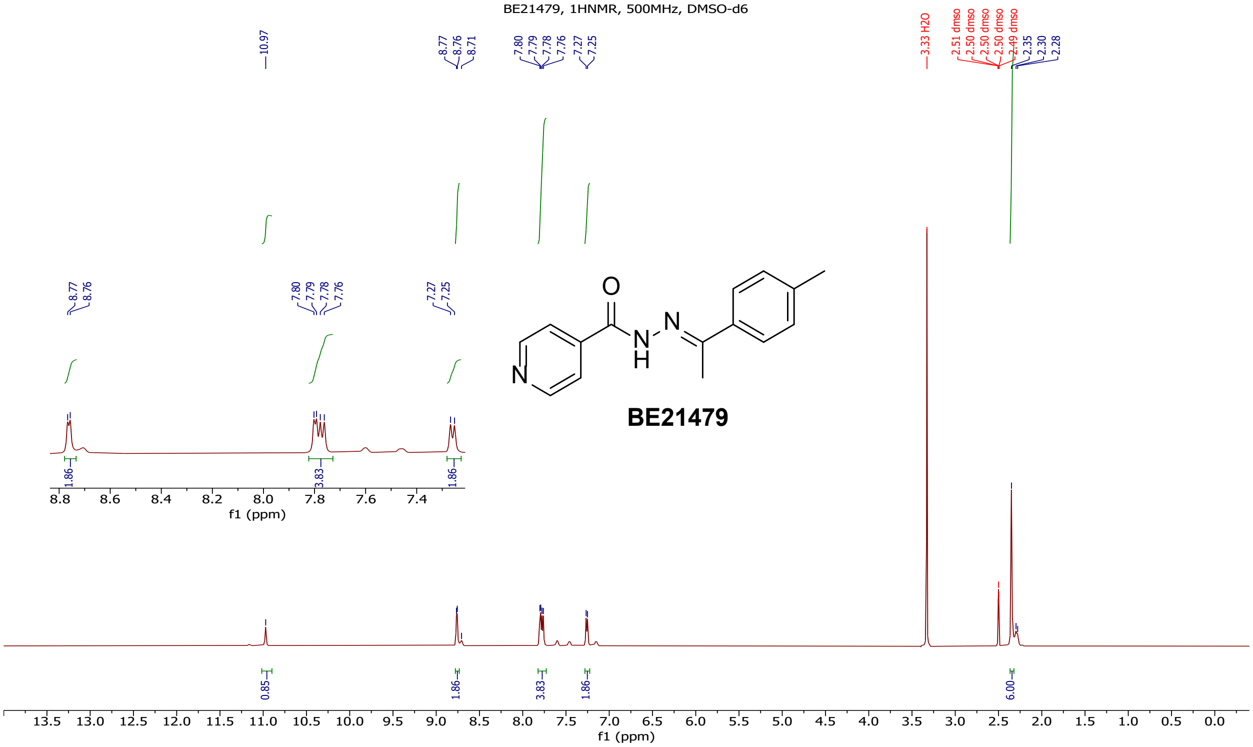

Figure S114. ^{1}H **NMR spectra of compound BE21479**

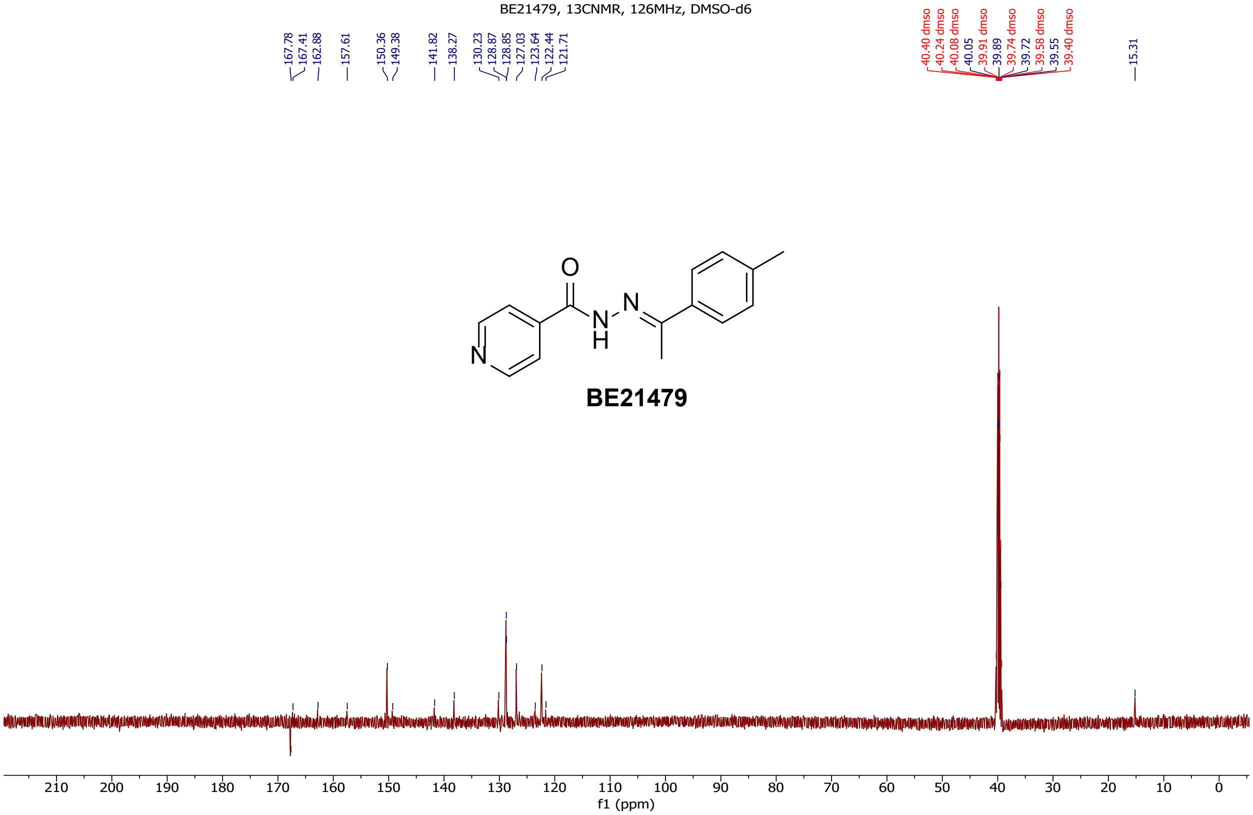

Figure S115. $^{13}C\{^{1}H\}$ **NMR spectra of compound BE21479**

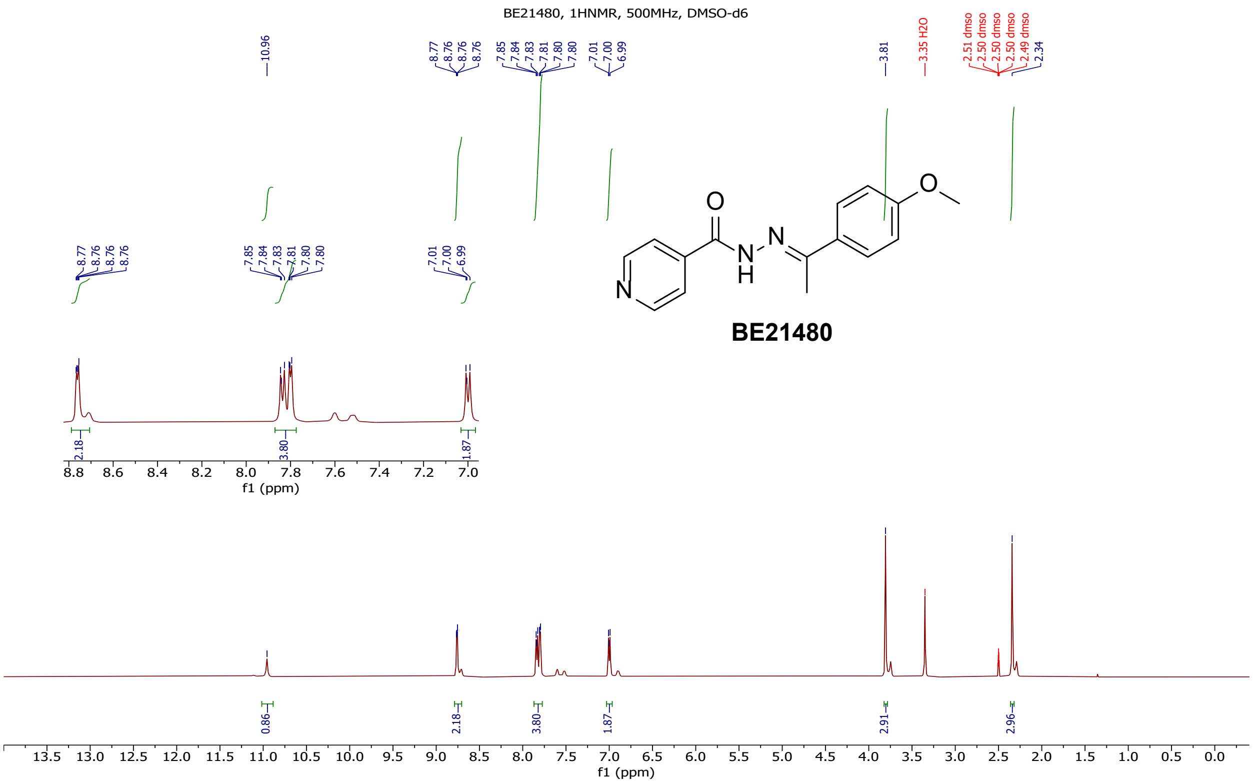

Figure S116. ^{1}H NMR spectra of compound BE21480

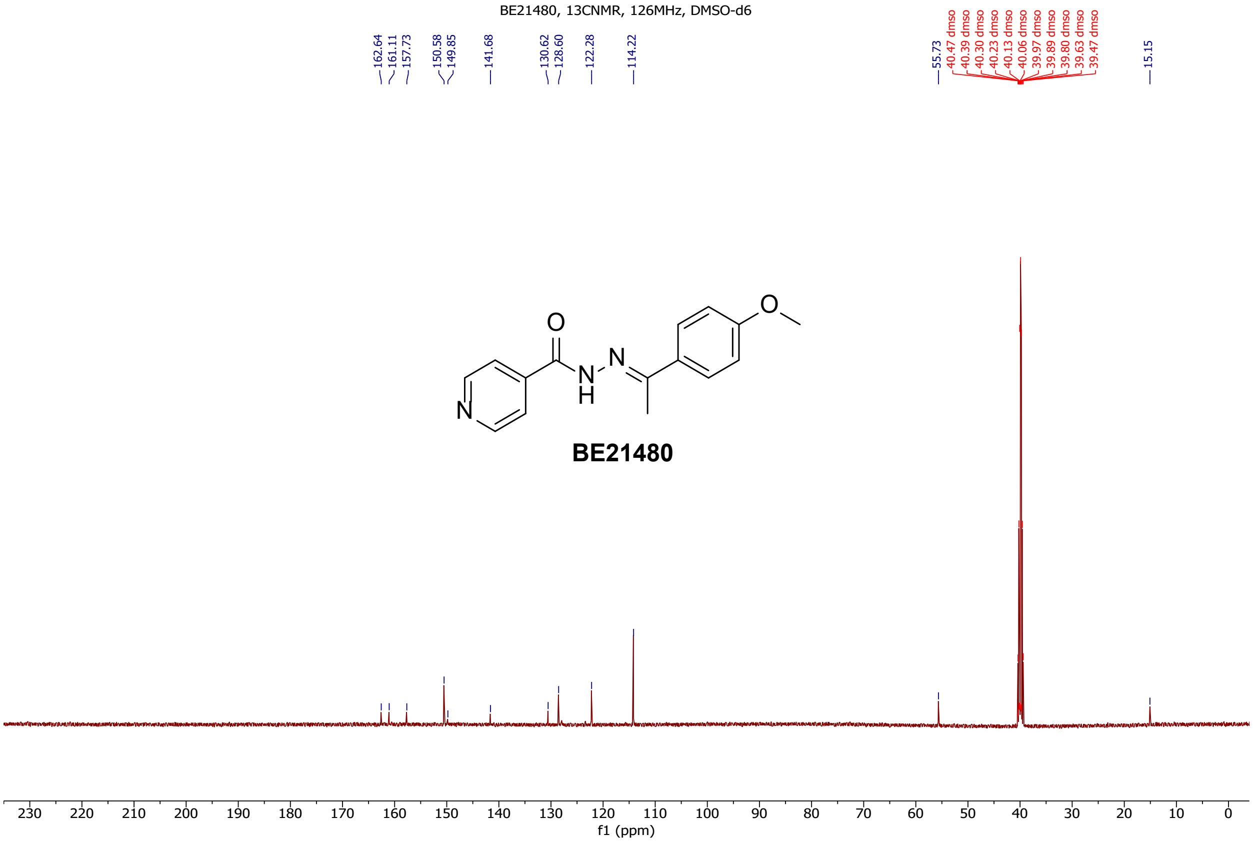

Figure S117. $^{13}C\{^{1}H\}$ NMR spectra of compound BE21480

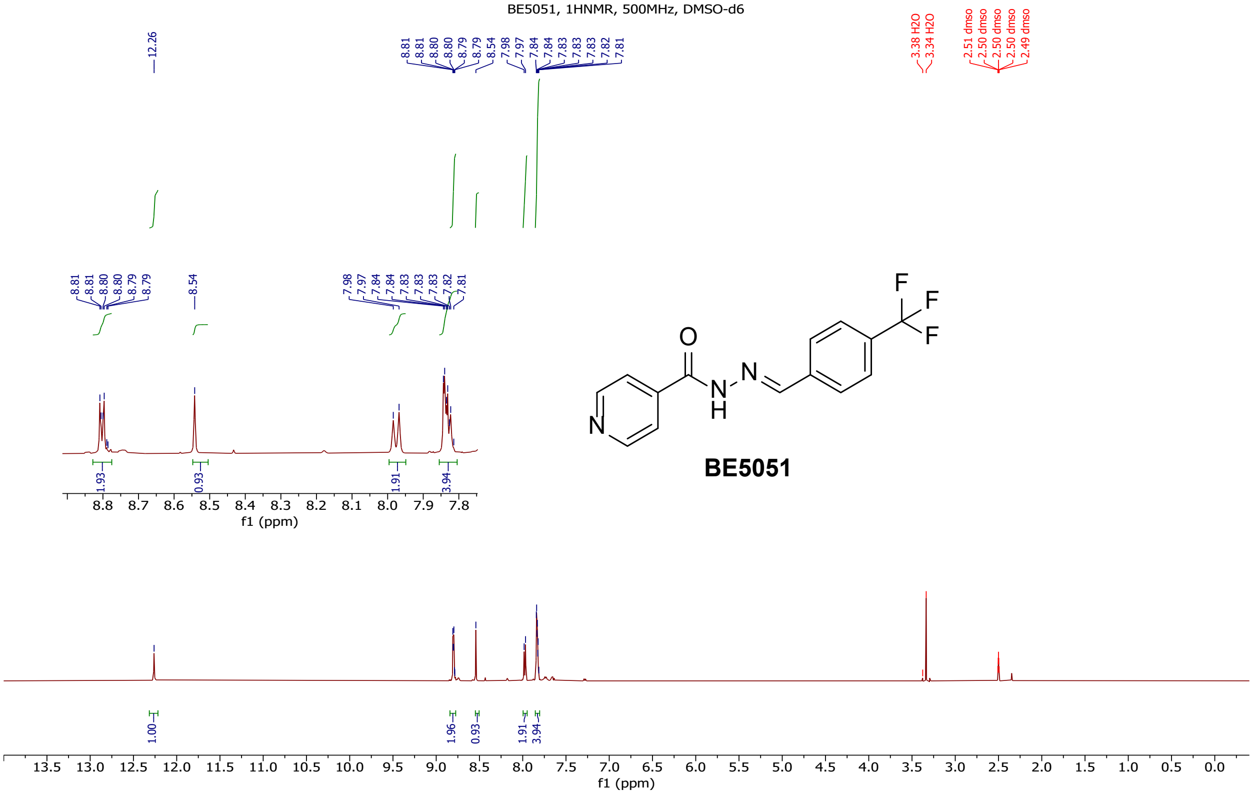

Figure S118. ^{1}H NMR spectra of compound BE5051

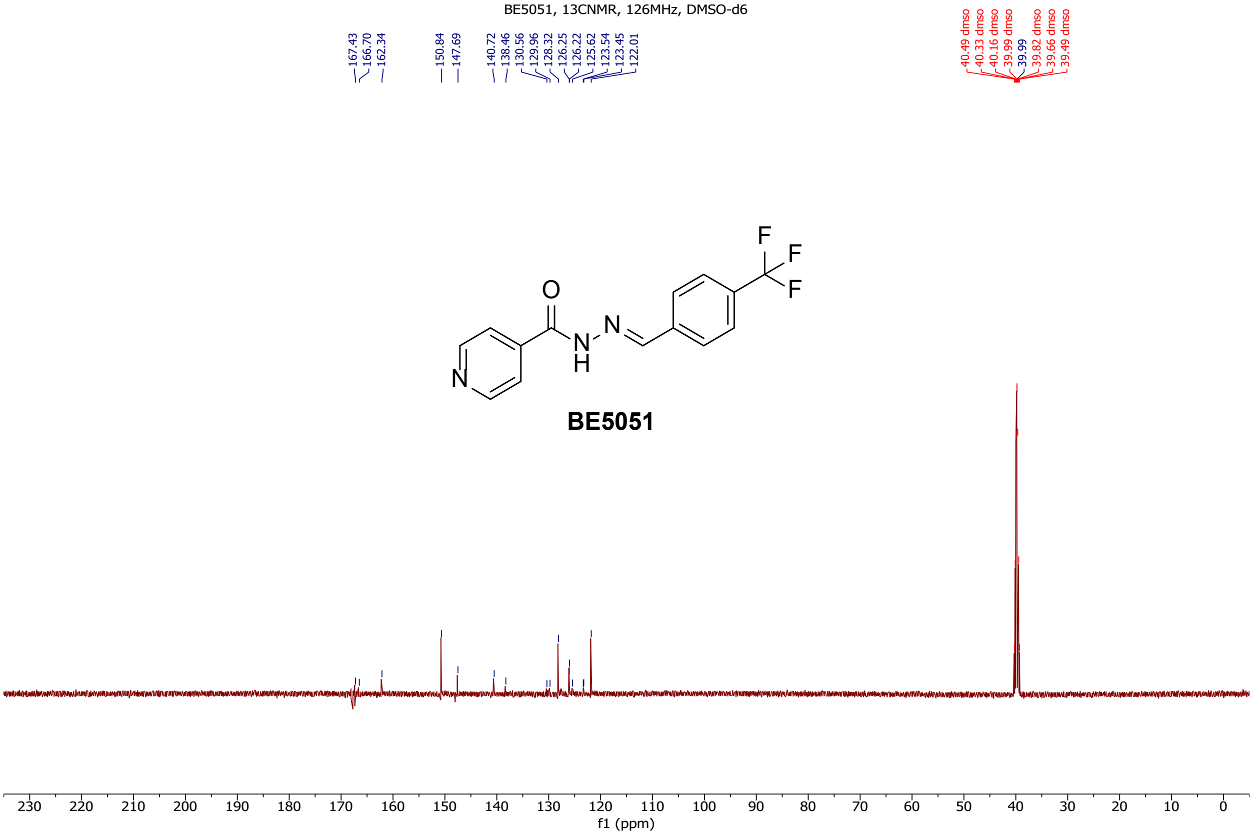

Figure S119. ^{13}C{^{1}H} NMR spectra of compound BE5051

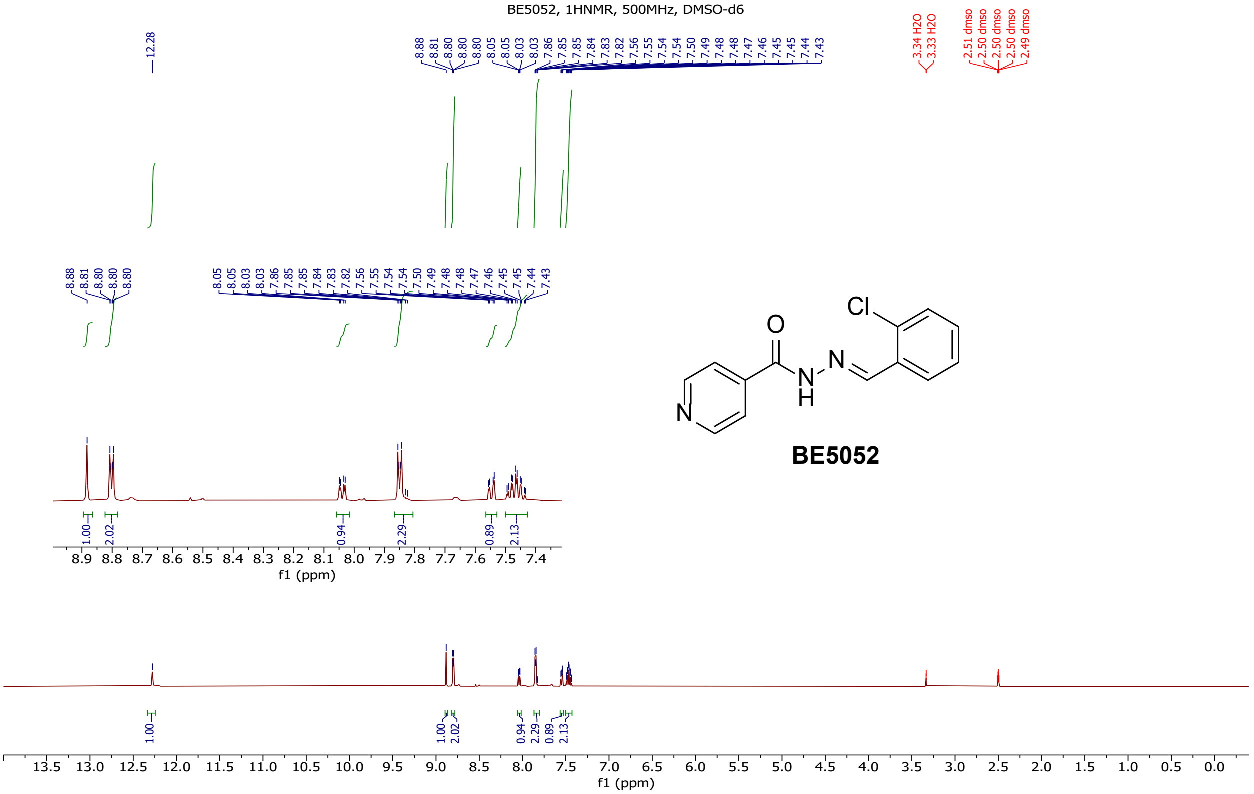

Figure S120. ^{1}H NMR spectra of compound BE5052

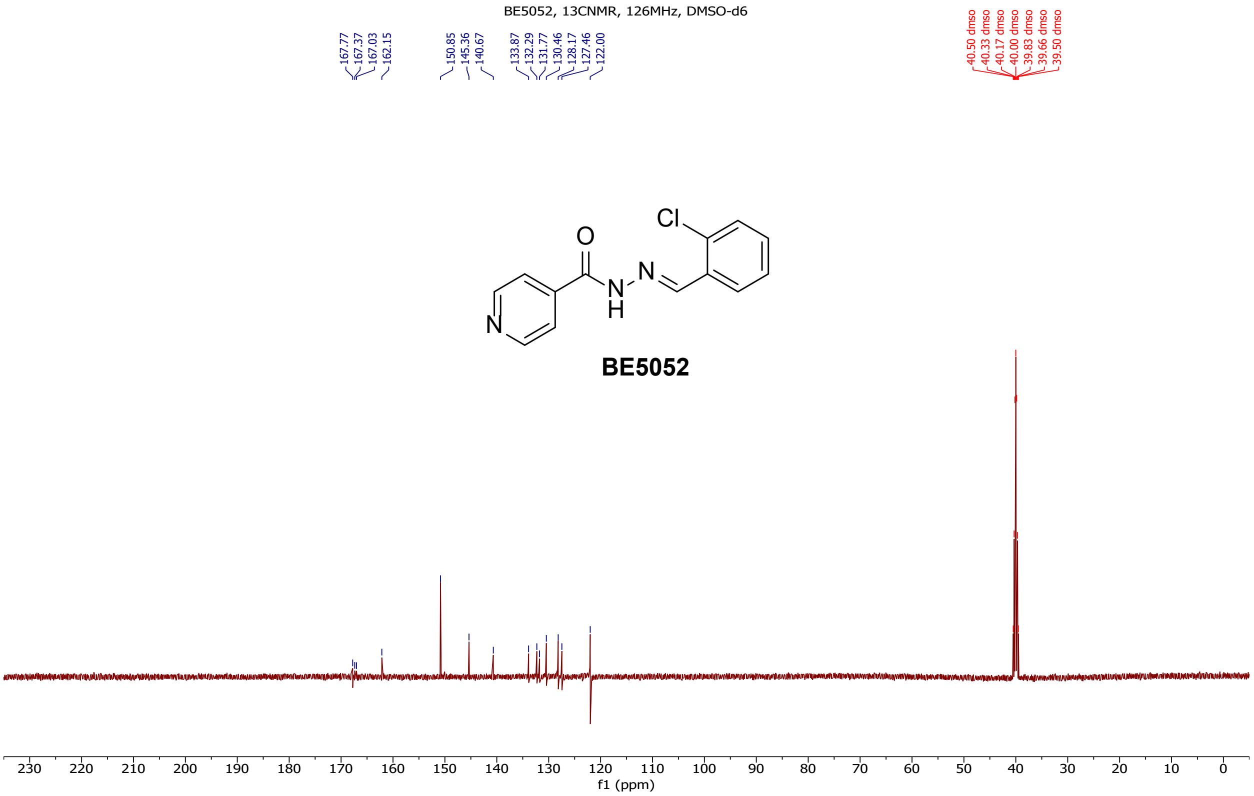

Figure S121. ^{13}C{^{1}H} NMR spectra of compound BE5052

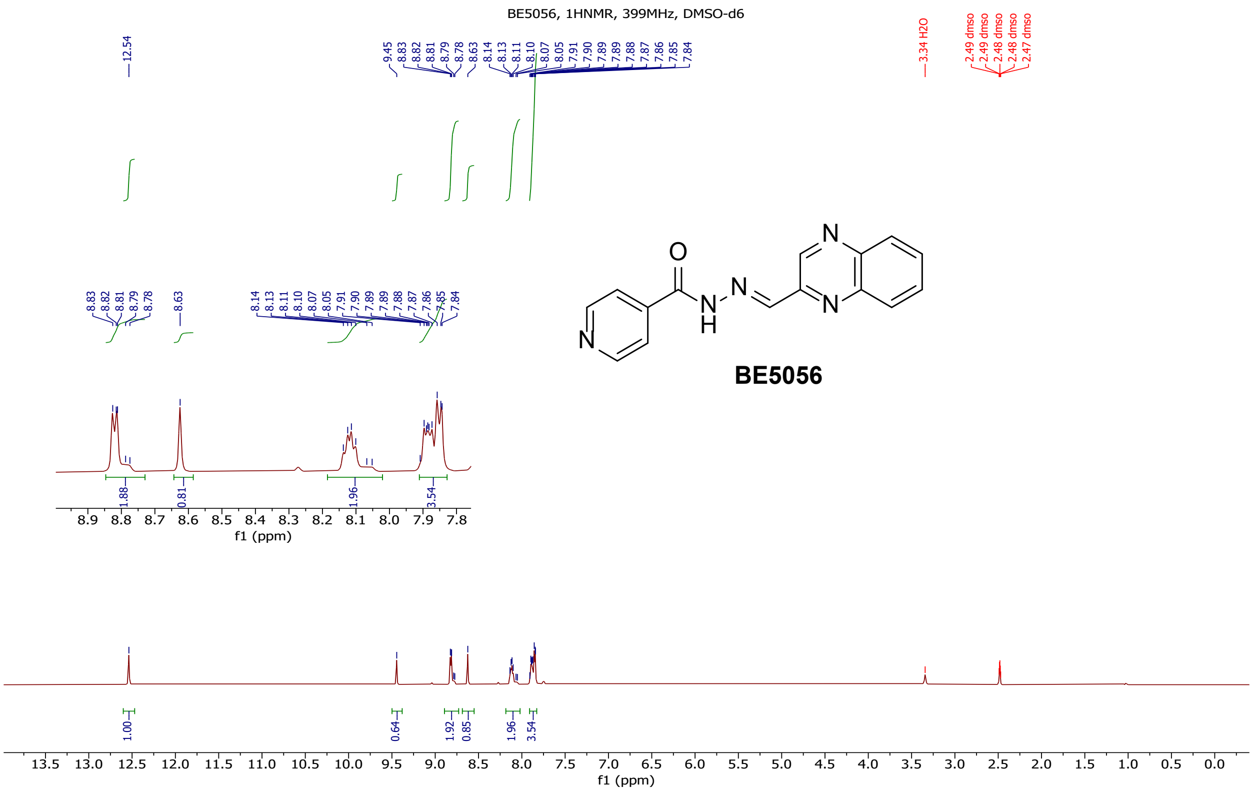

Figure S122. ^{1}H NMR spectra of compound BE5056

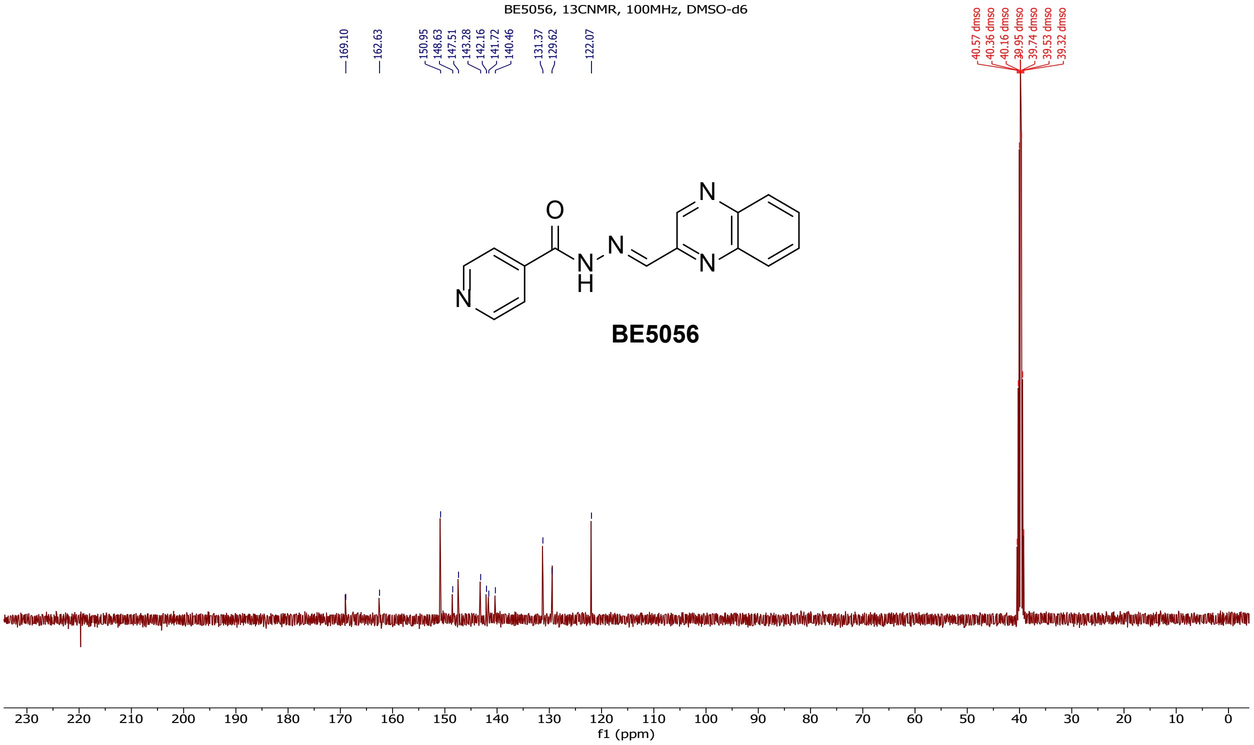

Figure S123. $^{13}C\{^1H\}$ NMR spectra of compound BE5056

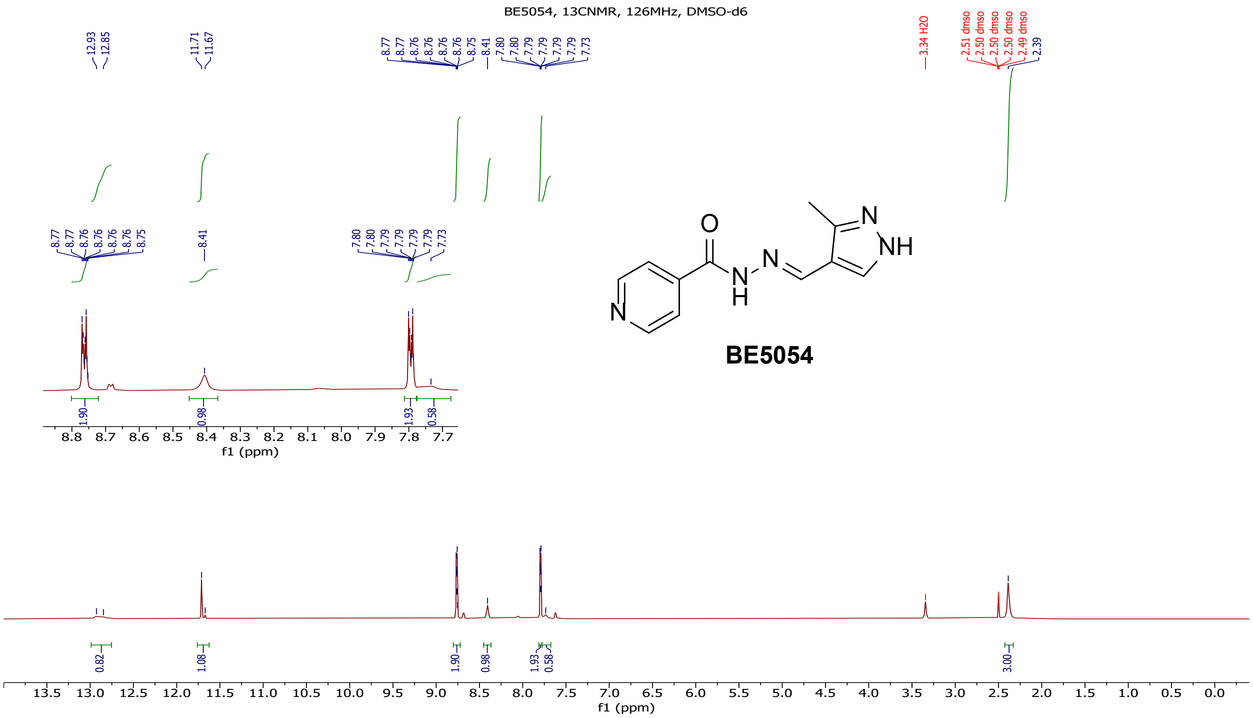

Figure S124. ^{1}H NMR spectra of compound BE5054

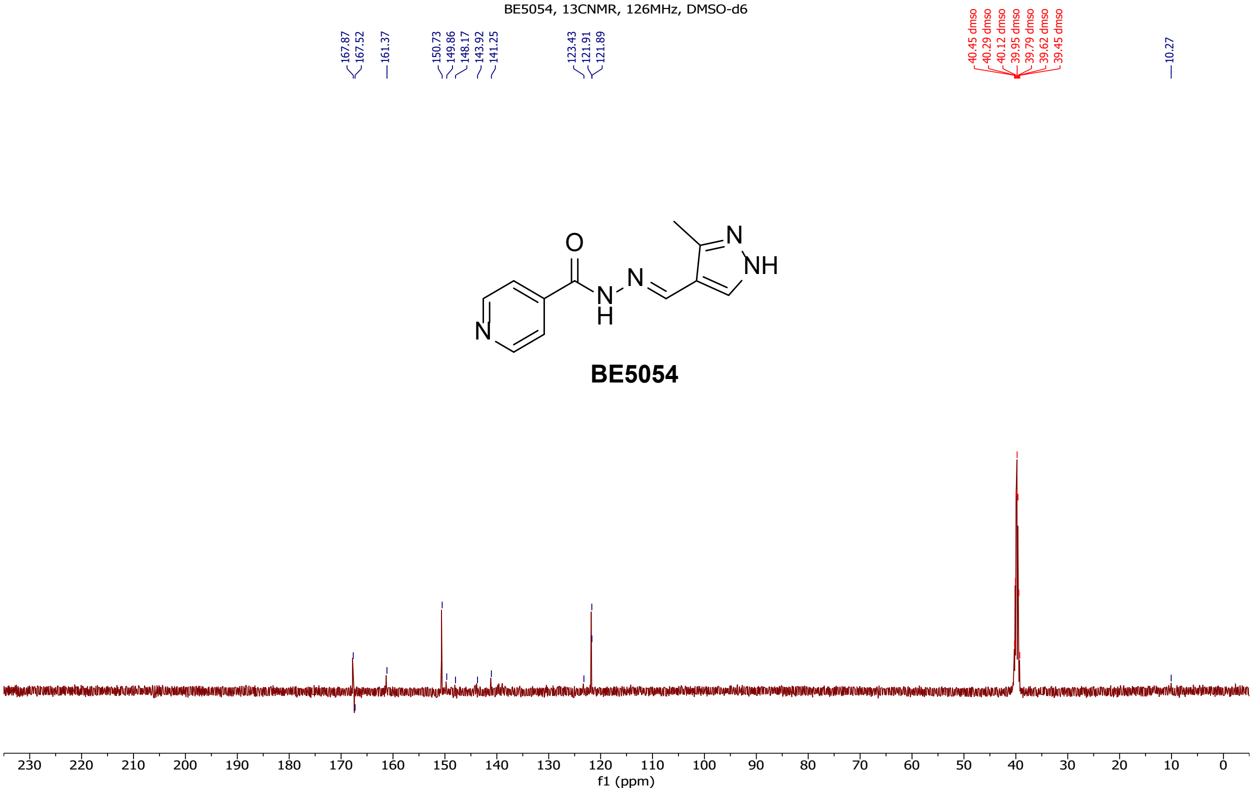

Figure S125. ^{13}C{^{1}H} NMR spectra of compound BE5054

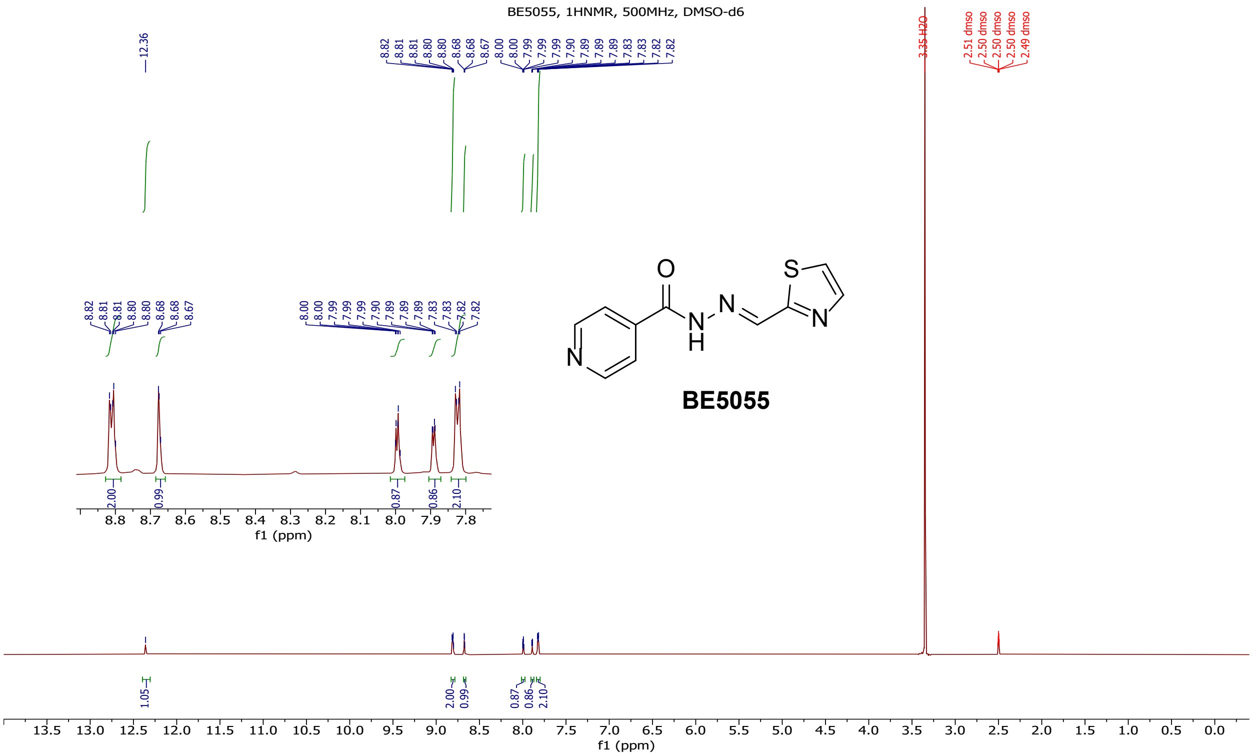

Figure S126. ^{1}H NMR spectra of compound BE5055

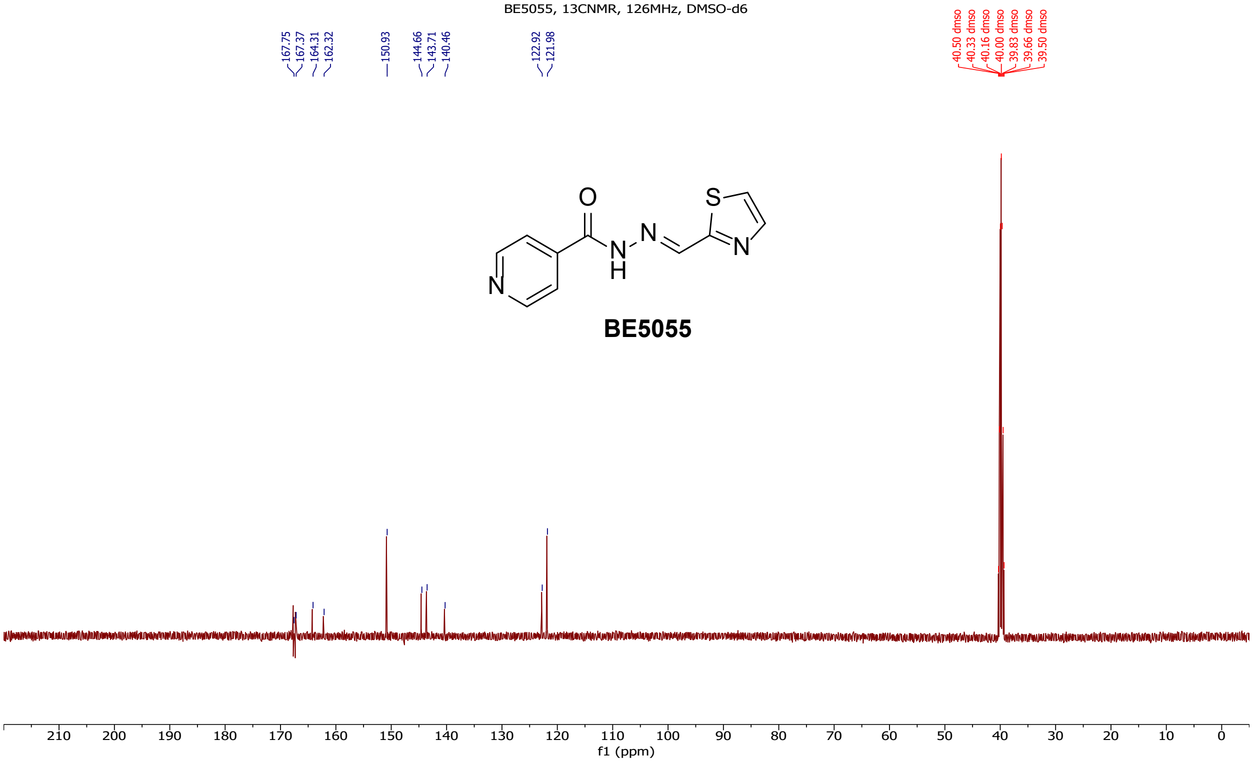

Figure S127. ^{13}C{^{1}H} NMR spectra of compound BE5055

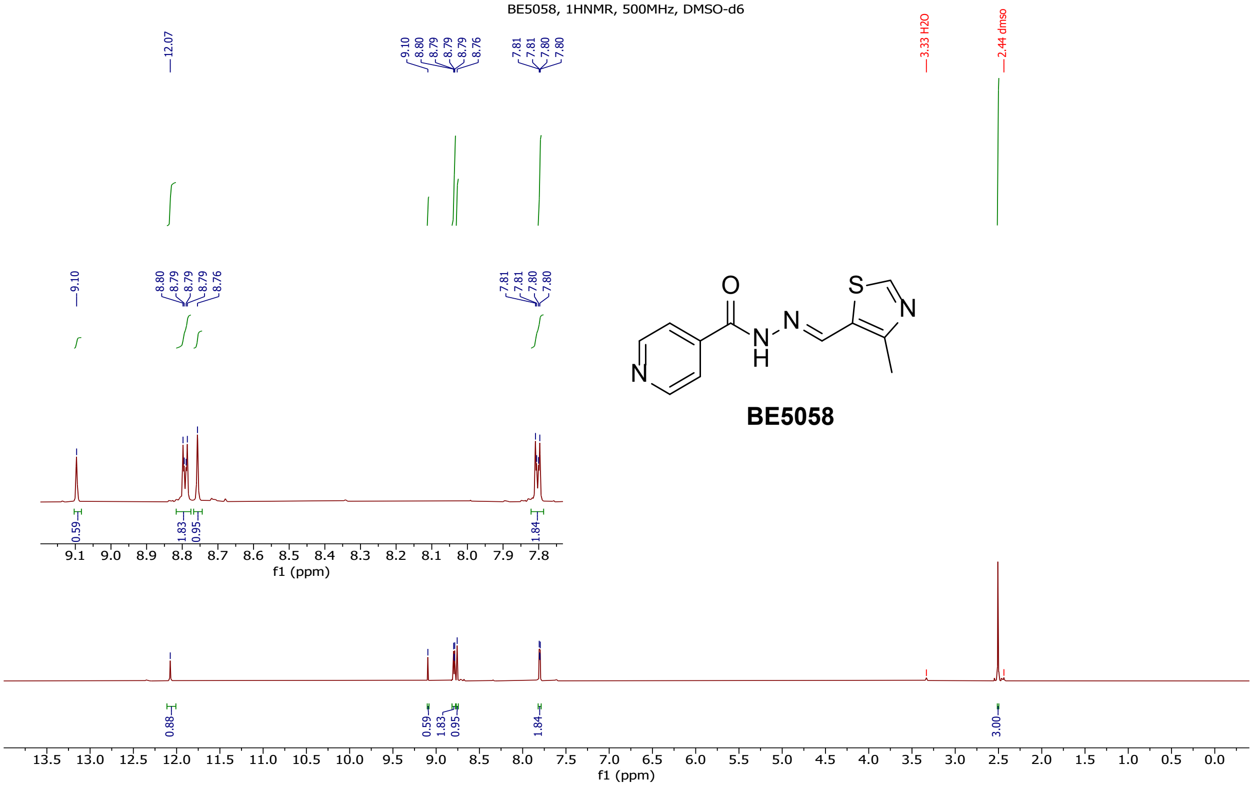

Figure S128. ^{1}H NMR spectra of compound BE5058

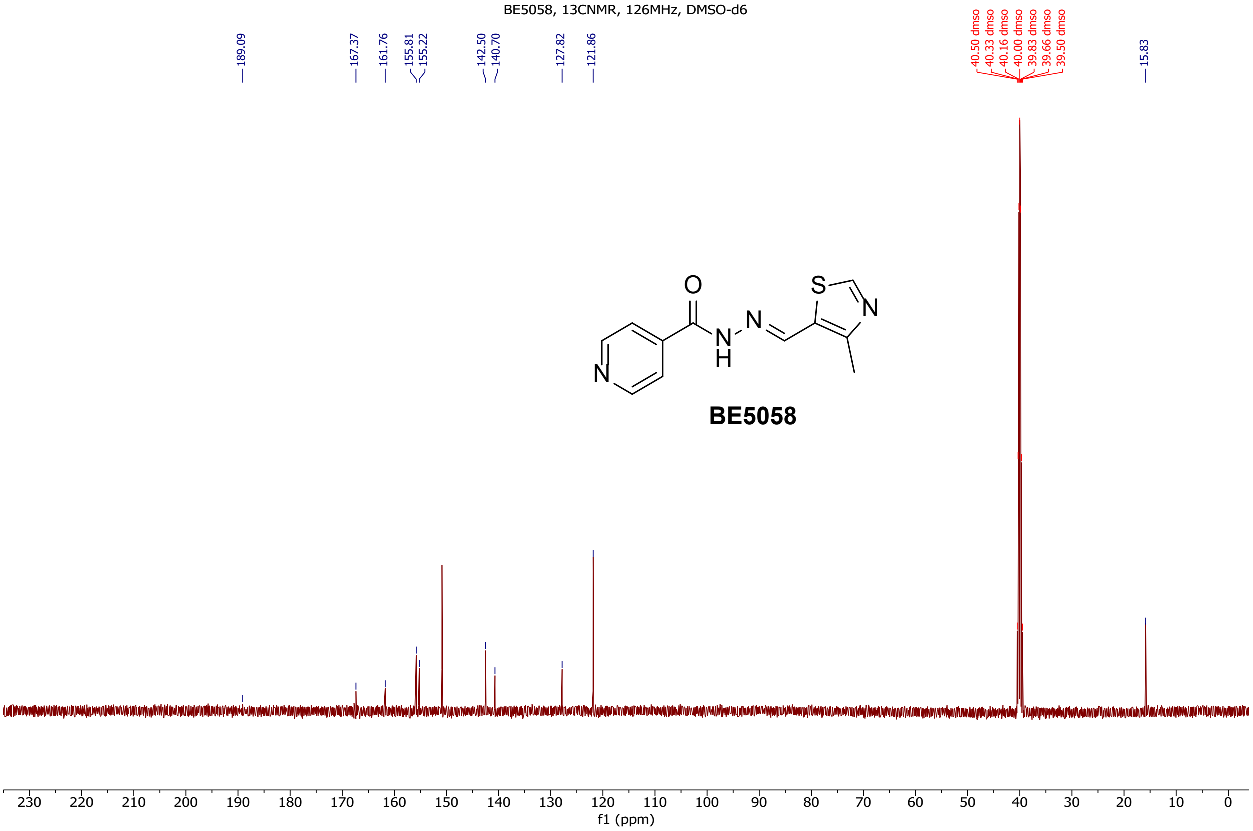

Figure S129. ^{13}C{^{1}H} NMR spectra of compound BE5058

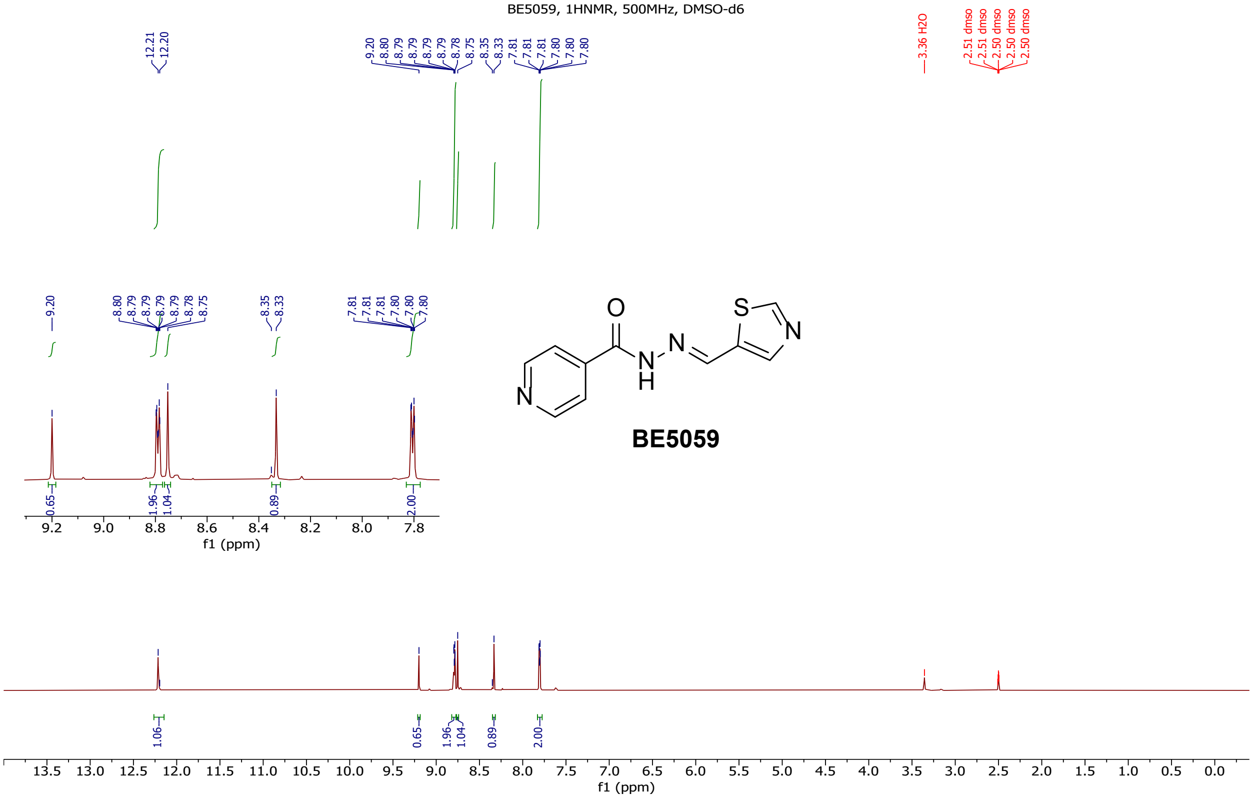

Figure S130. ^{1}H NMR spectra of compound BE5059

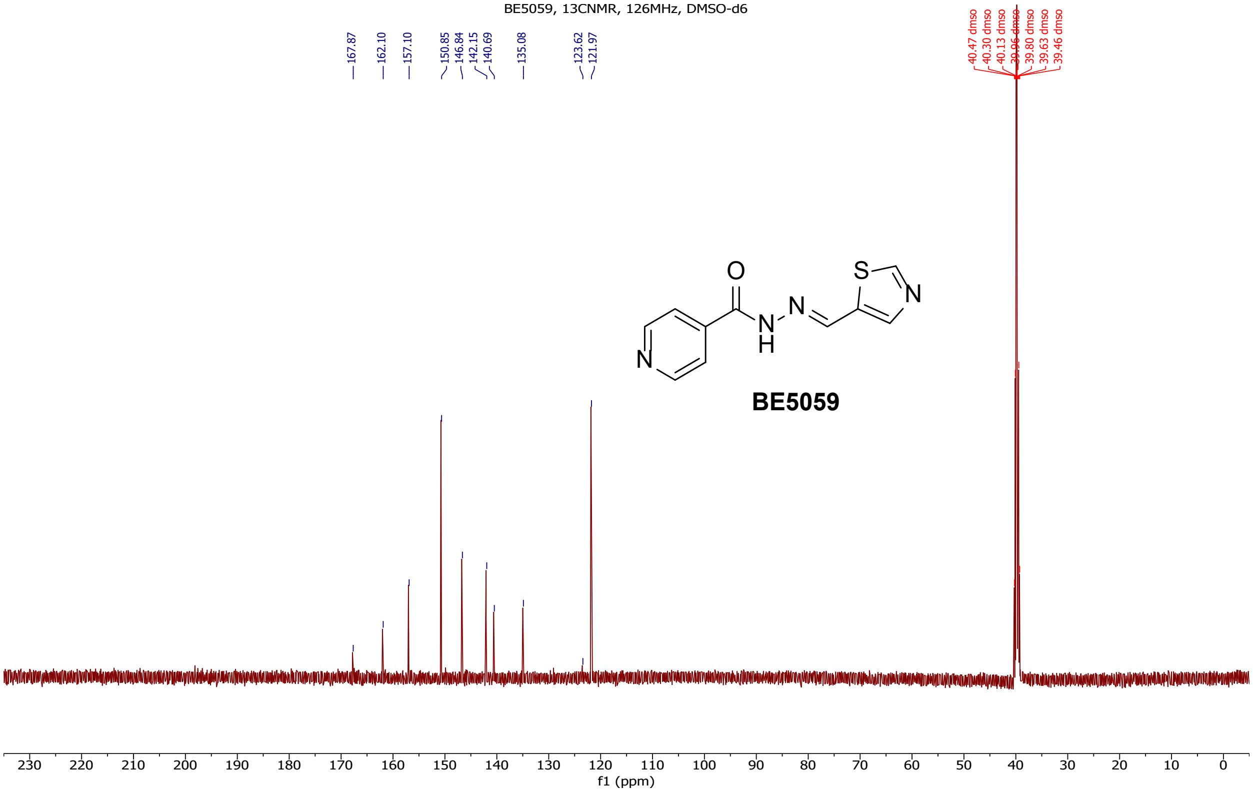

Figure S131. ^{13}C{^{1}H} NMR spectra of compound BE5059

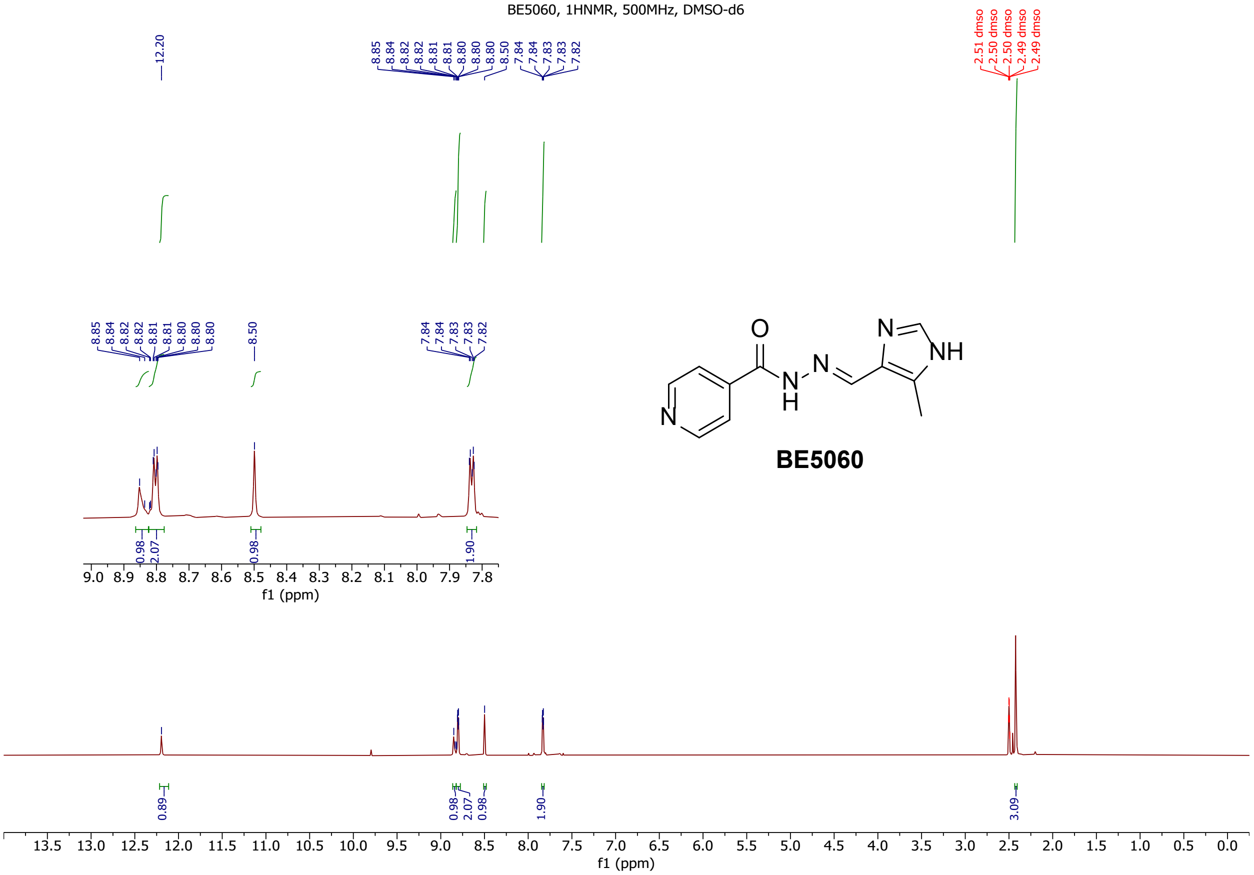

Figure S132. **^{1}H NMR spectra of compound BE5060**

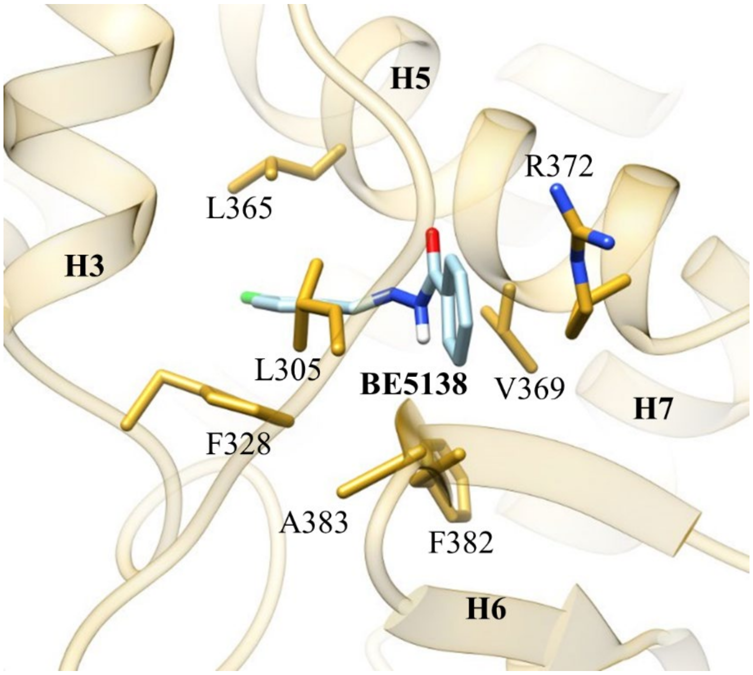

Figure S133. Representative conformations **BE5138** in complex with ERRα obtained from MD simulations.

5. LC–MS Characterization of ERR-Active Compounds

(E)-4-Hydroxy-*N′*-(naphthalen-2-ylmethylene)benzohydrazide (SLU-PP-332)

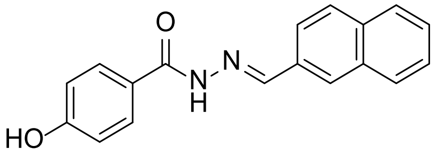

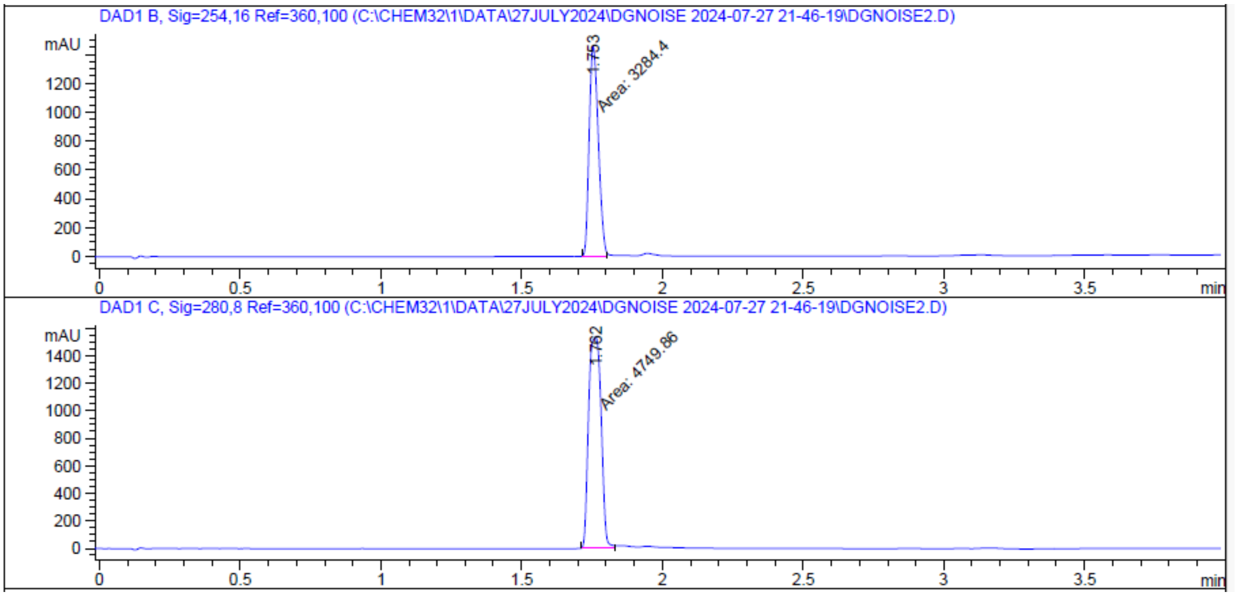

Peak	RT (min)	Area	Area %
1	1.75	3284.4	100.00

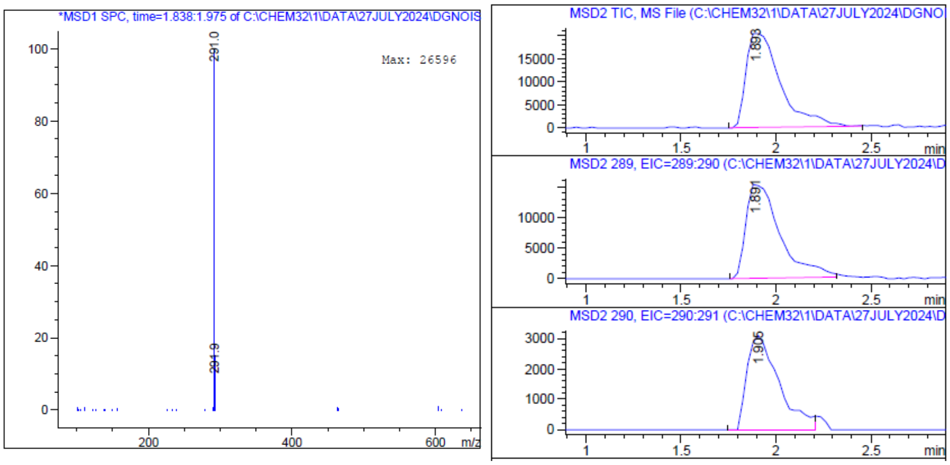

(E)-*N′*-Benzylidene-4-methylbenzohydrazide (BE5082)

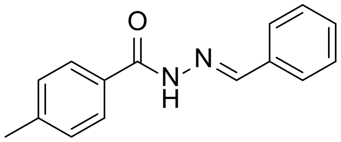

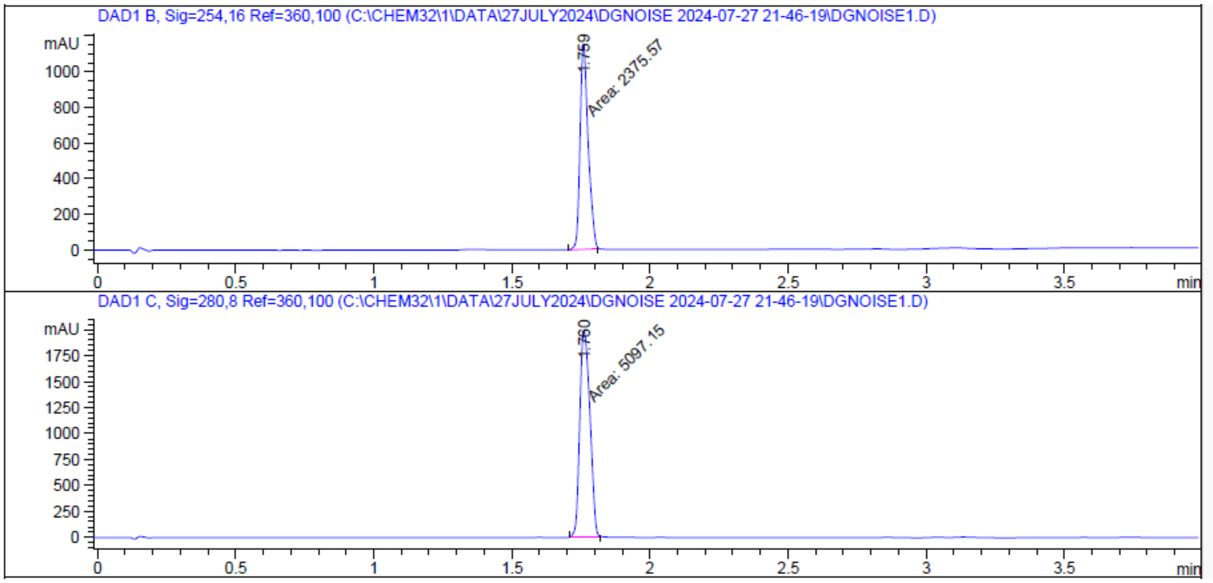

Peak	RT (min)	Area	Area %
1	1.75	2375.57	99.2

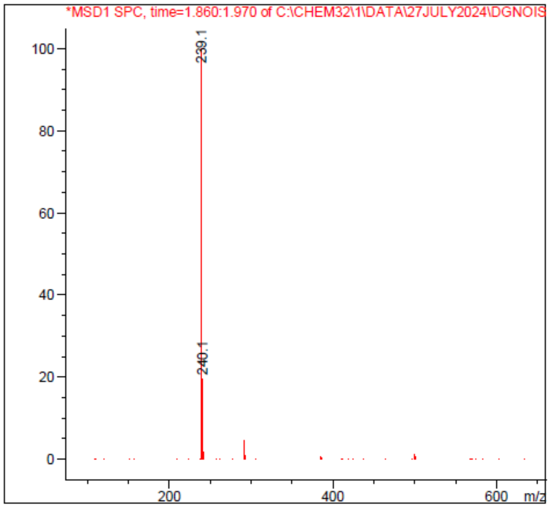

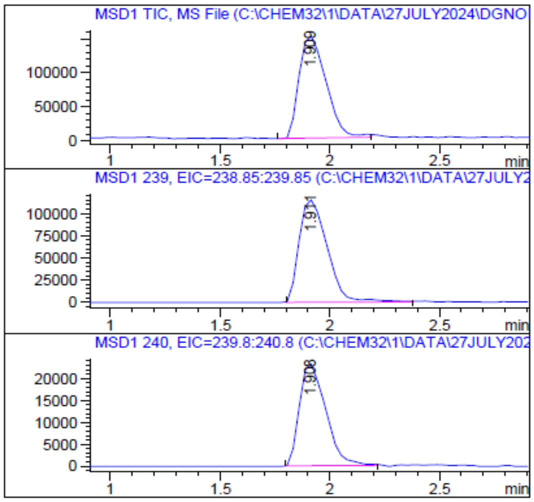

(E)-4-Methyl-N'-(1-phenylethylidene)benzohydrazide (BE5027)

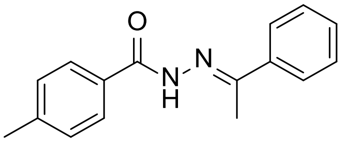

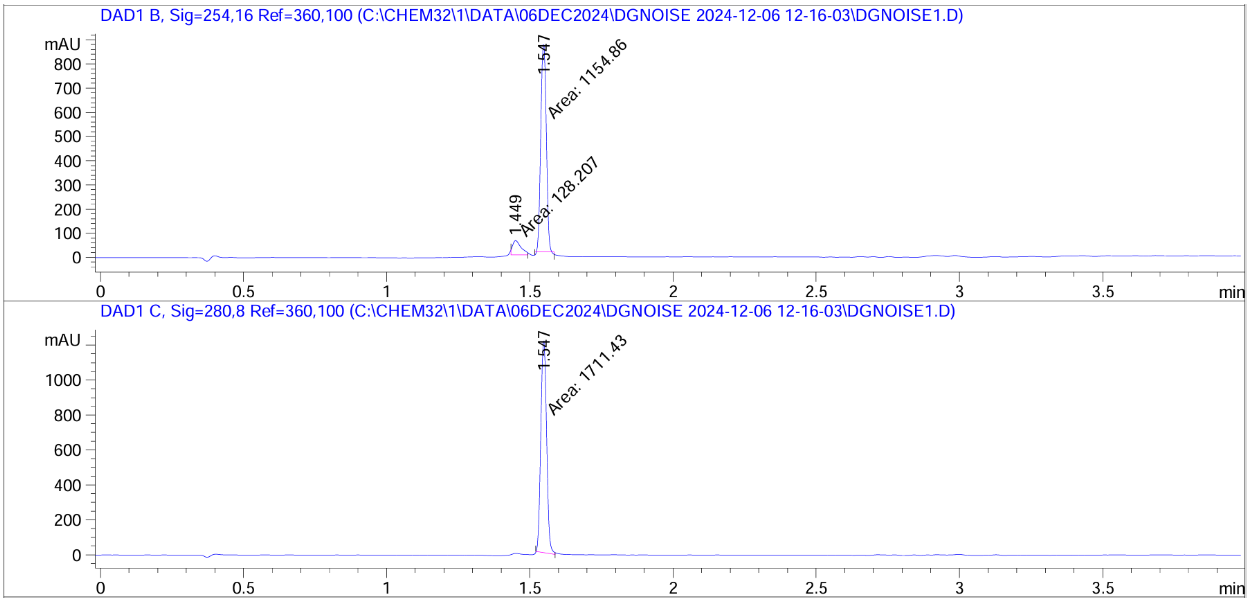

Peak	RT (min)	Area	Area %
1	1.547	1711.43	98.1

(E)-N'-(1-(3,4-Dichlorophenyl)ethylidene)-4-methylbenzohydrazide (BE5032)

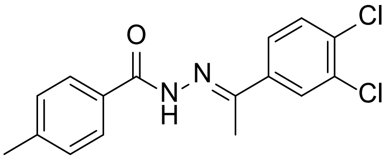

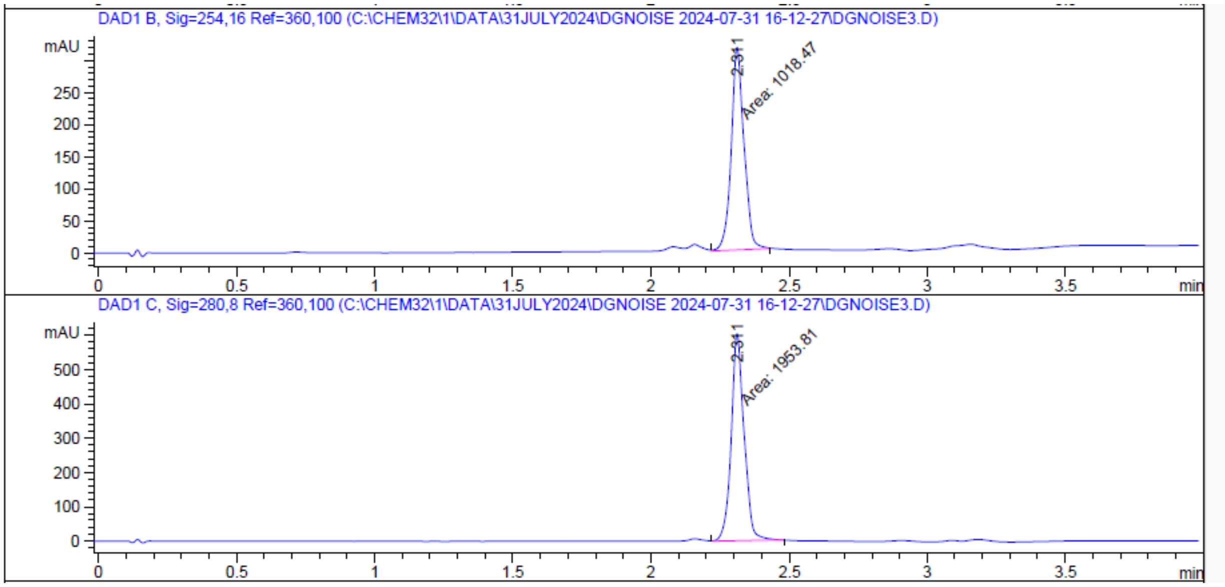

Peak	RT (min)	Area	Area %
1	2.31	1018.47	98.64

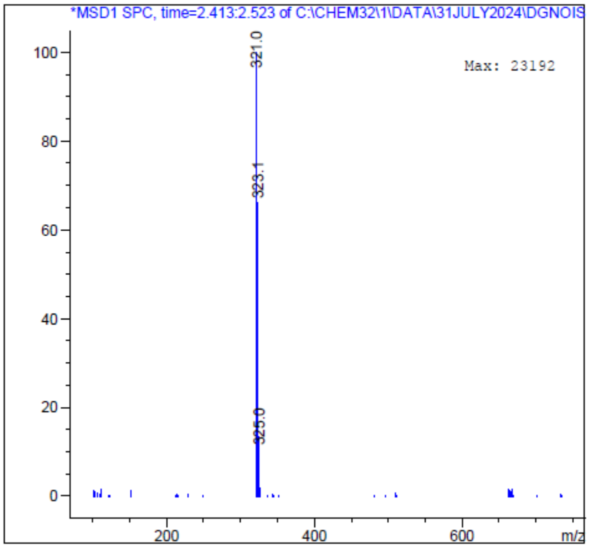

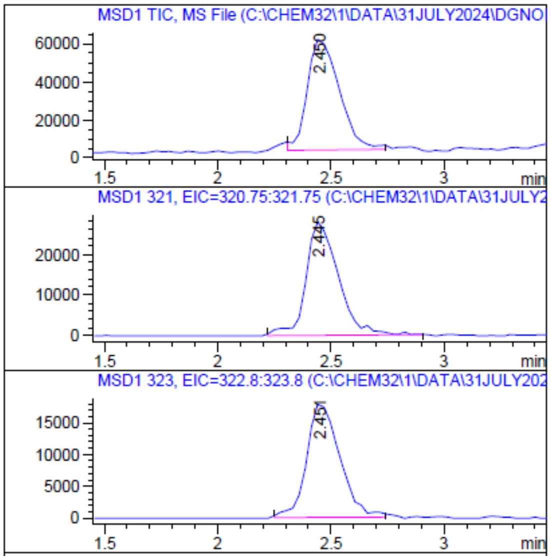

(E)-4-Methyl-*N′*-(naphthalen-1-ylmethylene)benzohydrazide (BE5086)

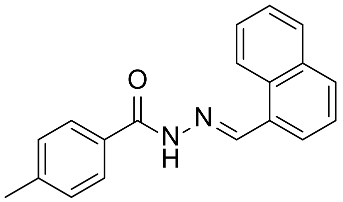

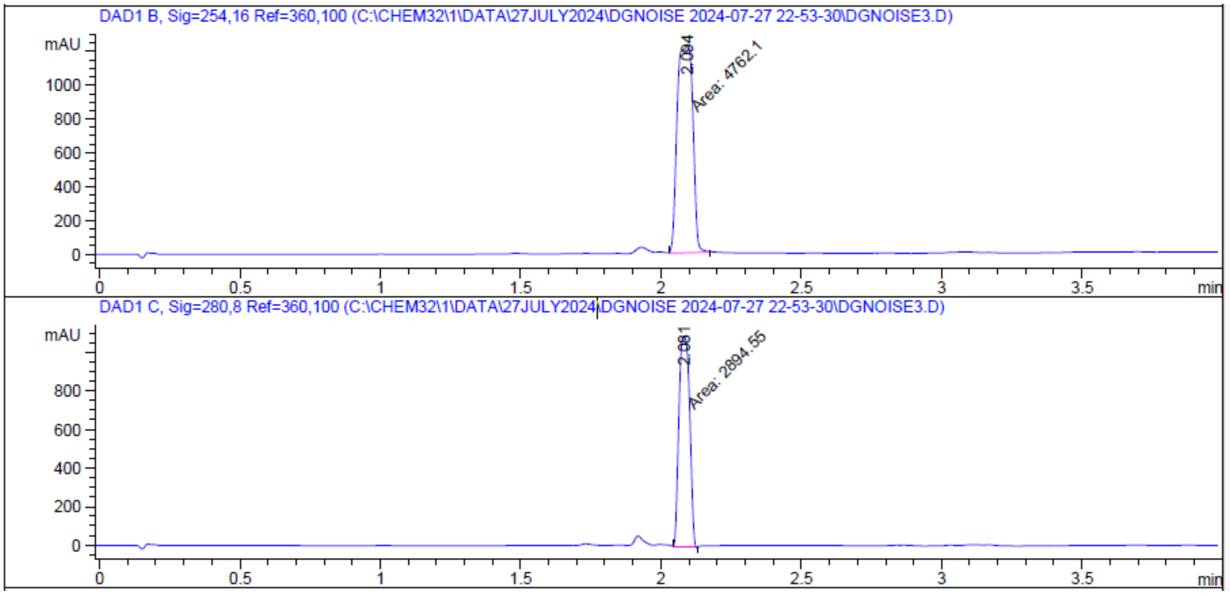

Peak	RT (min)	Area	Area %
1	2.08	4762.1	100

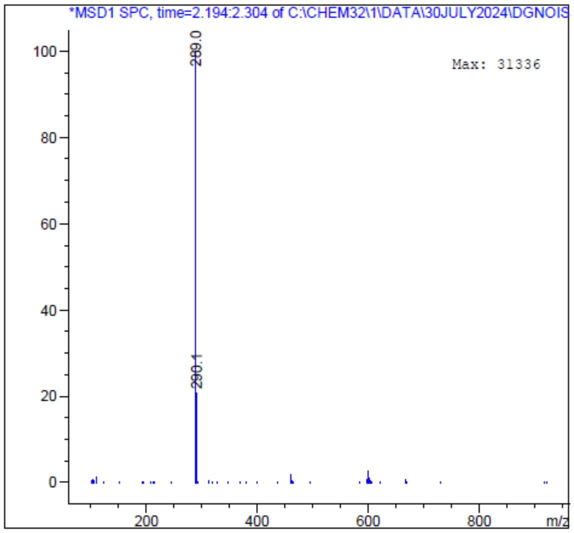

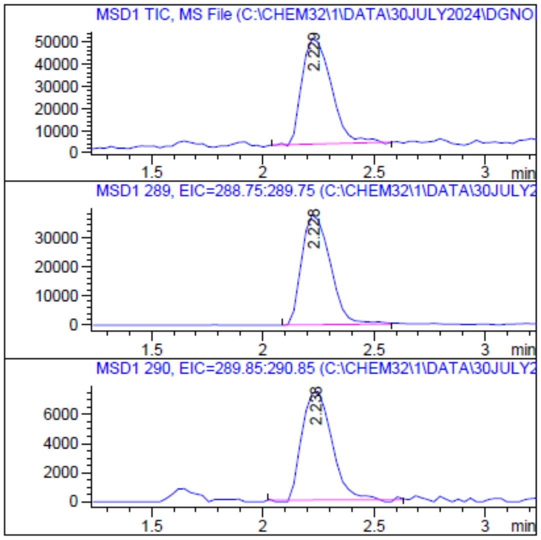

***(E)*-*N'*-(Naphthalen-2-ylmethylene)benzohydrazide (BE5066)**

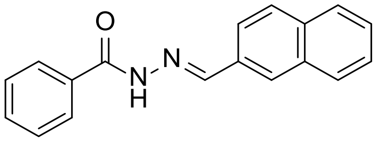

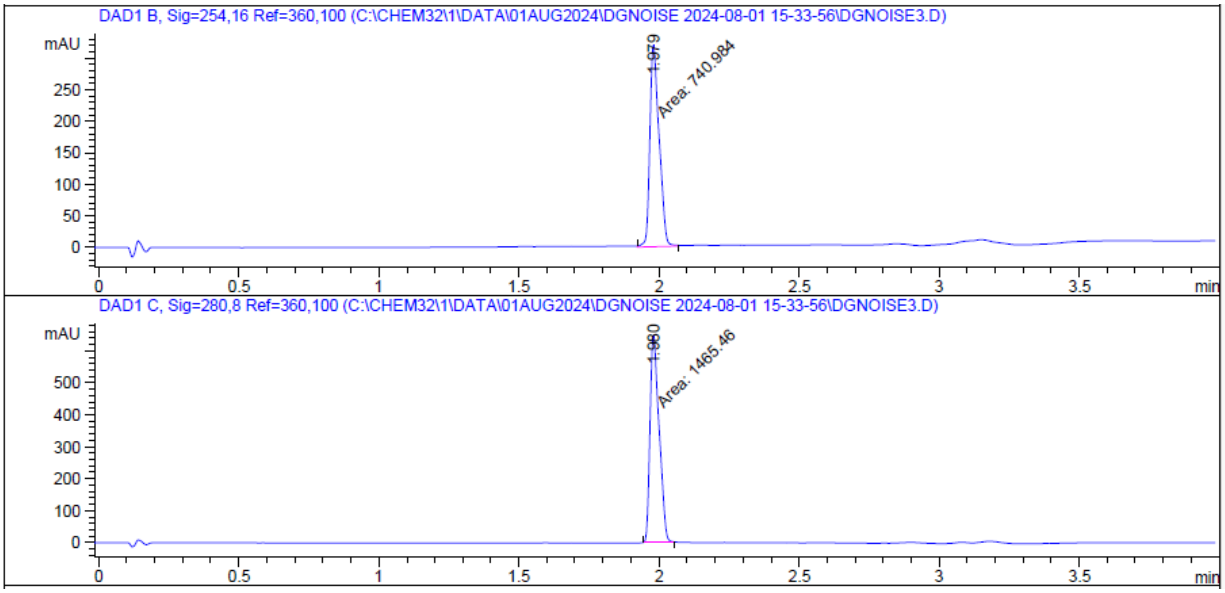

Peak	RT (min)	Area	Area %
1	1.97	740.98	99.8

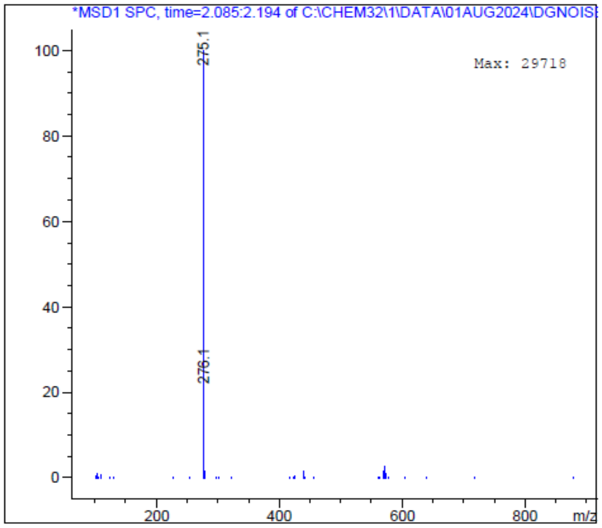

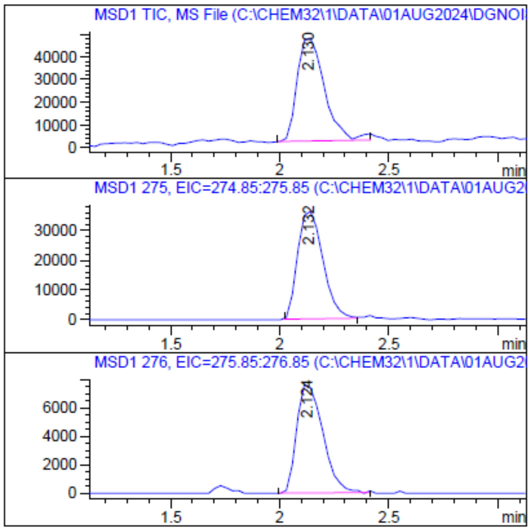

(E)-4-Hydroxy-*N′*-(1-phenylethylidene)benzohydrazide (BE5035)

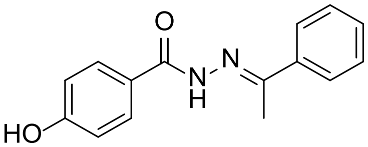

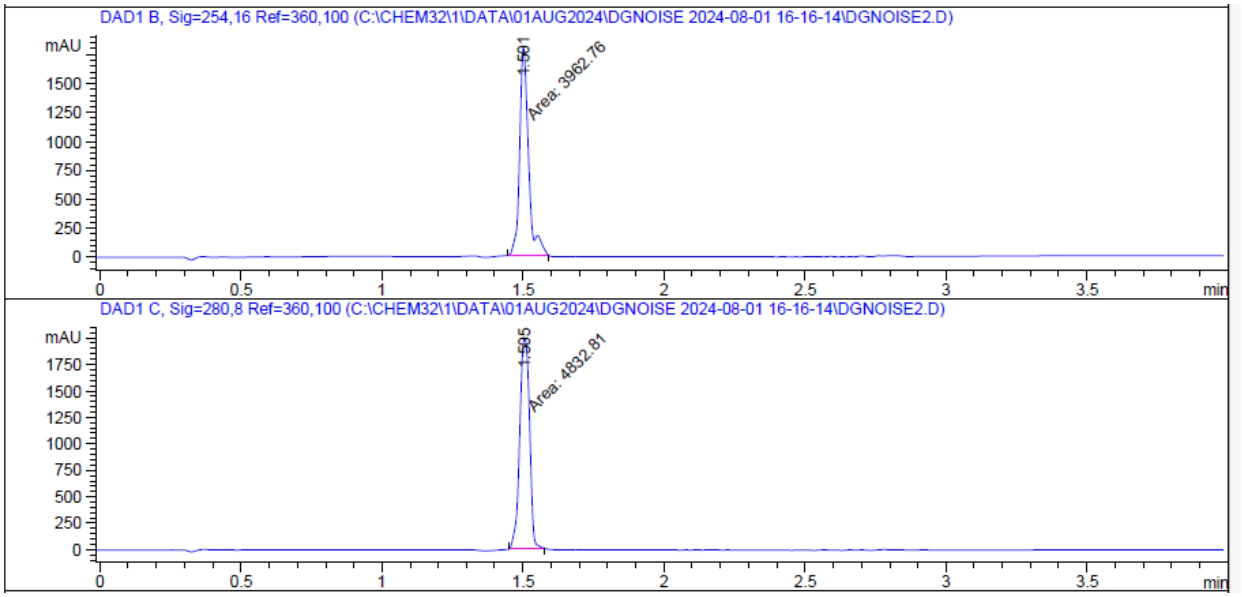

Peak	RT (min)	Area	Area %
1	1.5	3962.76	100

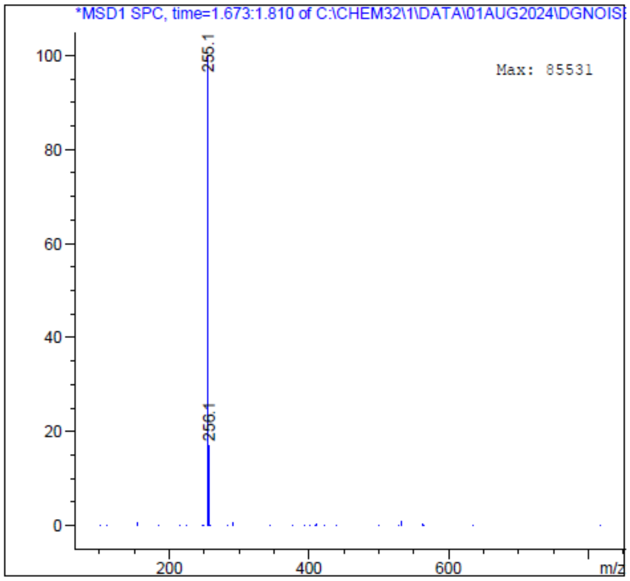

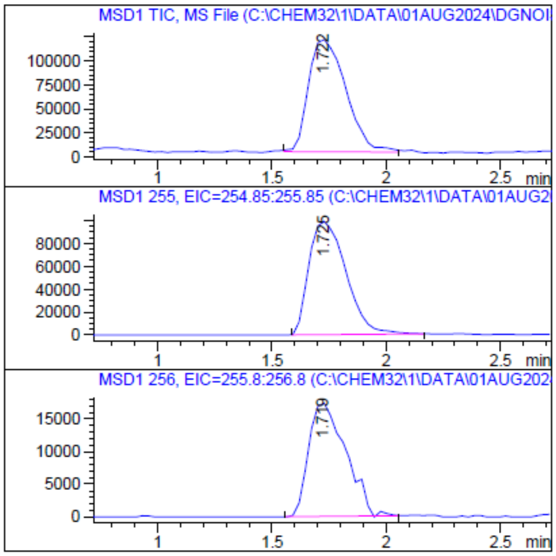

(*E*)-*N′*-(Naphthalen-2-ylmethylene)isonicotinohydrazide (BE5048)

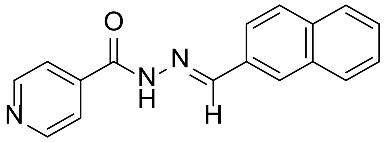

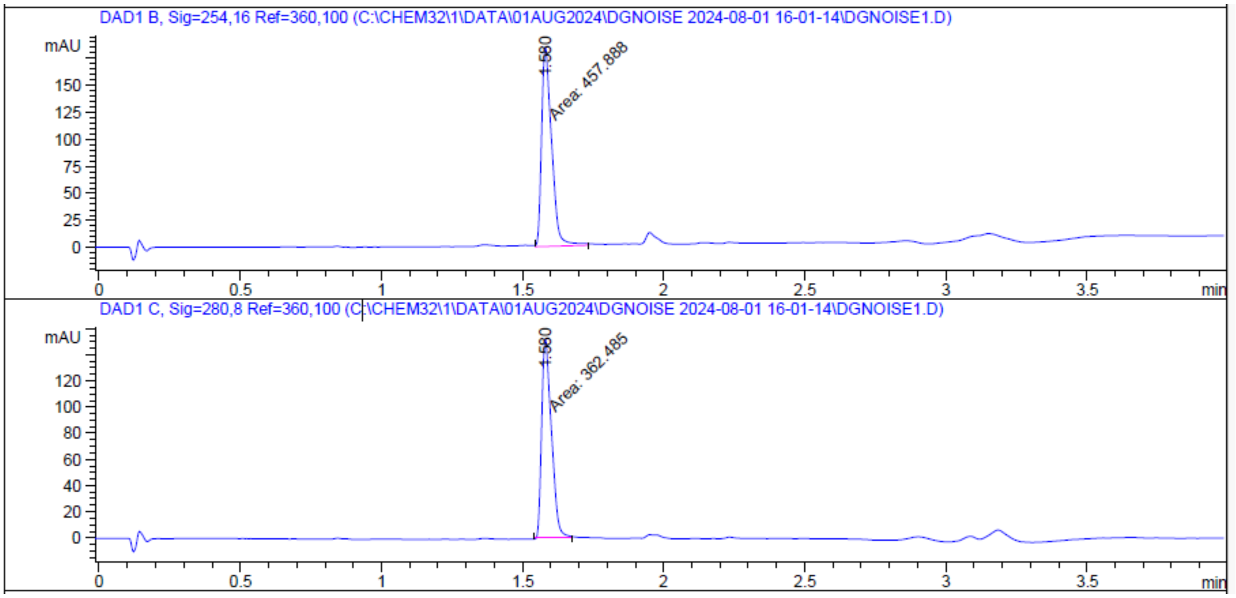

Peak	RT (min)	Area	Area %
1	1.58	457.88	100

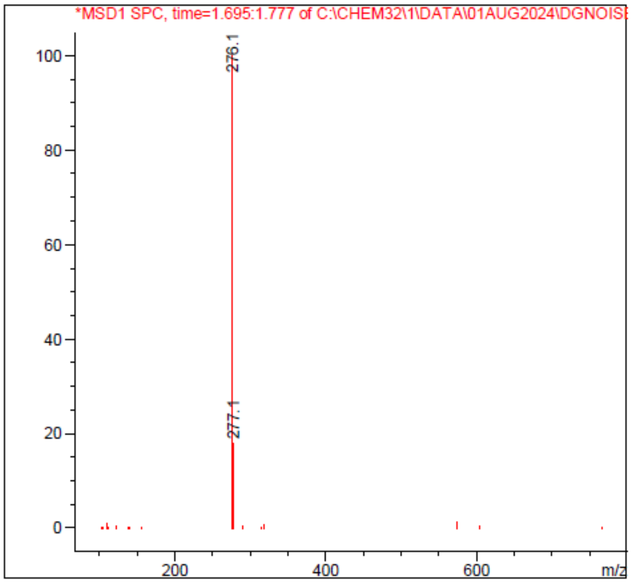

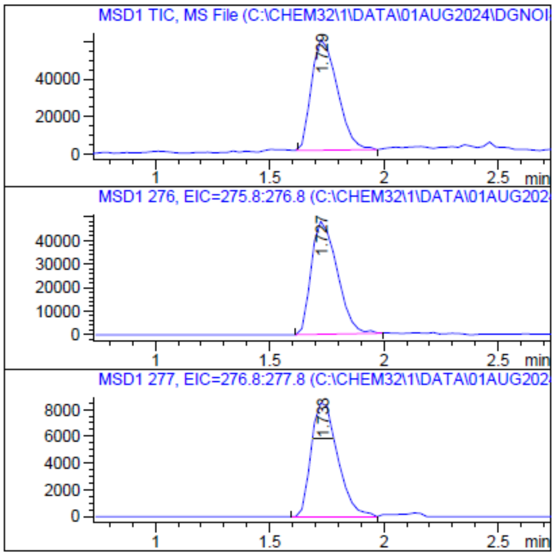

(E)-*N'*-(1-(Naphthalen-2-yl)ethylidene)isonicotinohydrazide (BE5049)

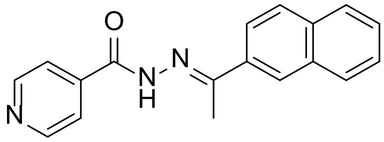

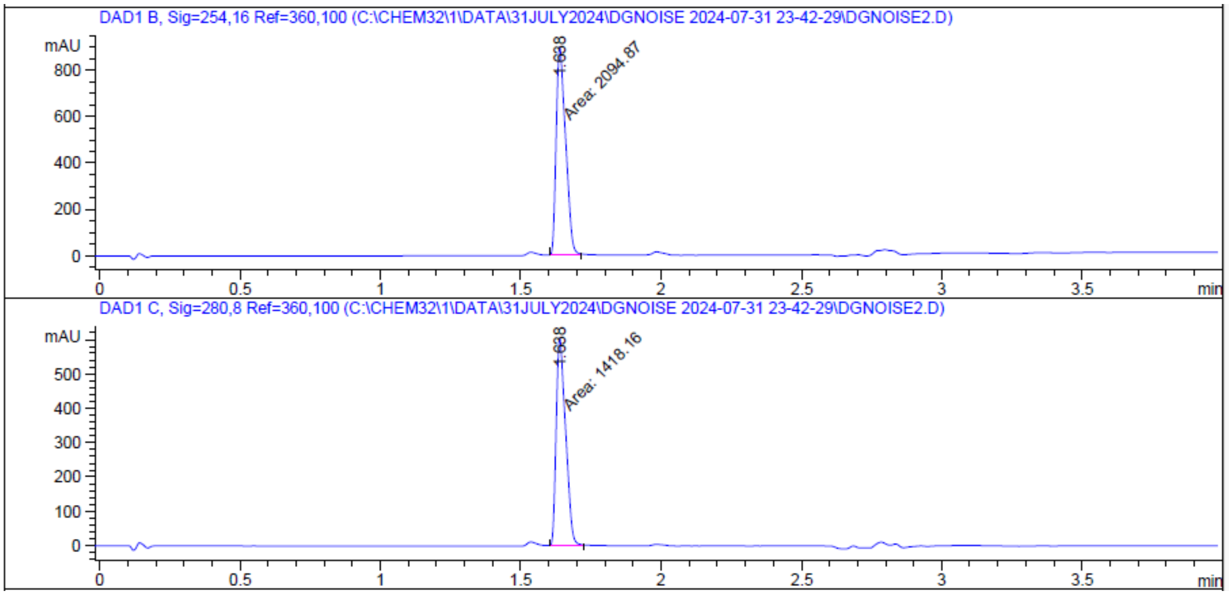

Peak	RT (min)	Area	Area %
1	1.68	2094.87	100

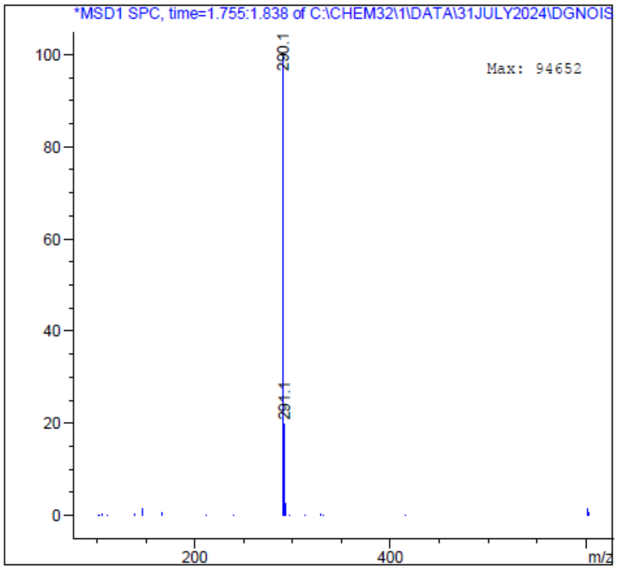

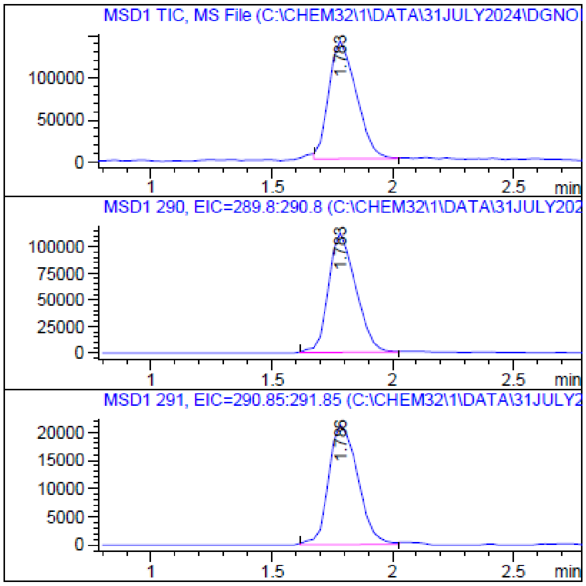

(E)-N′-(Isoquinolin-4-ylmethylene)-4-methylbenzohydrazide (BE5112)

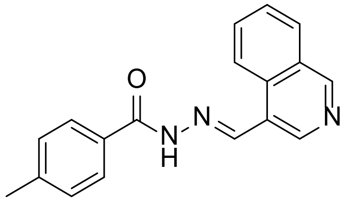

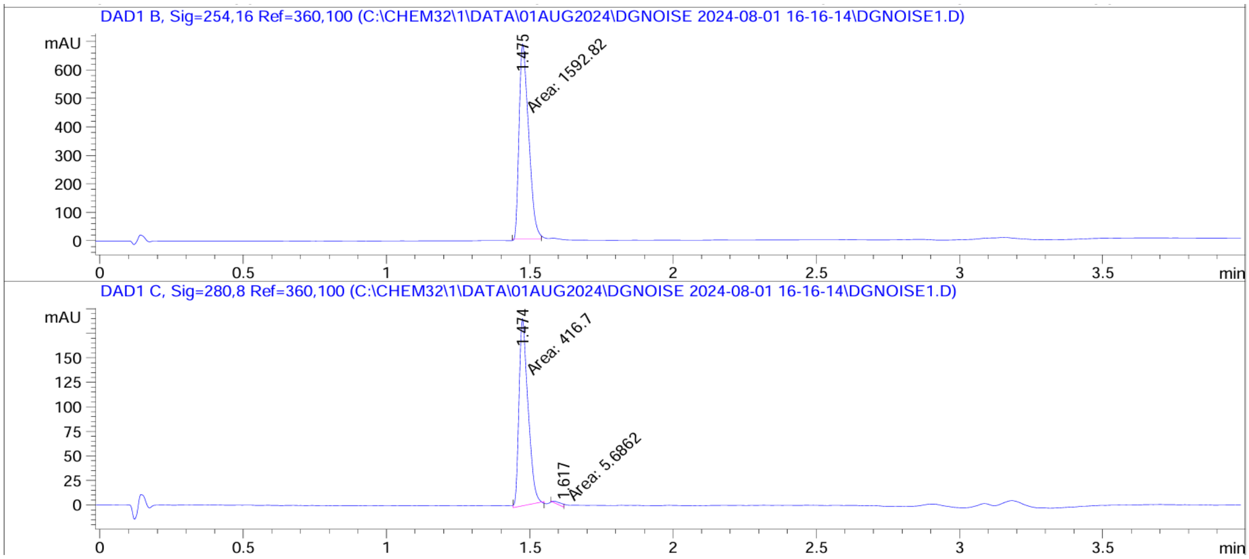

Peak	RT (min)	Area	Area %
1	1.474	416.7	98.65
2	1.614	5.686	1.36

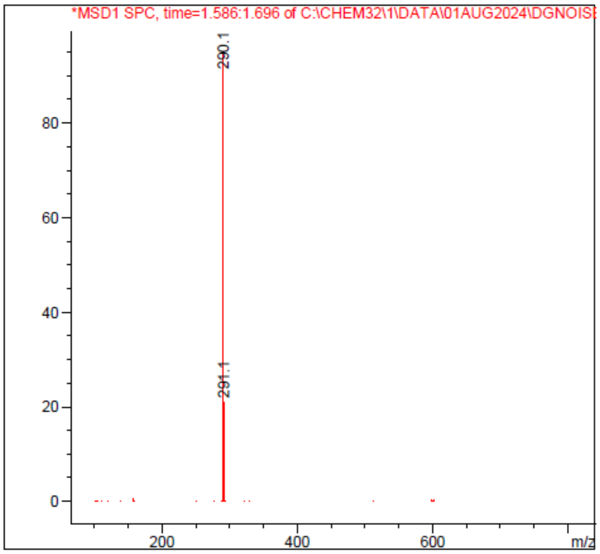

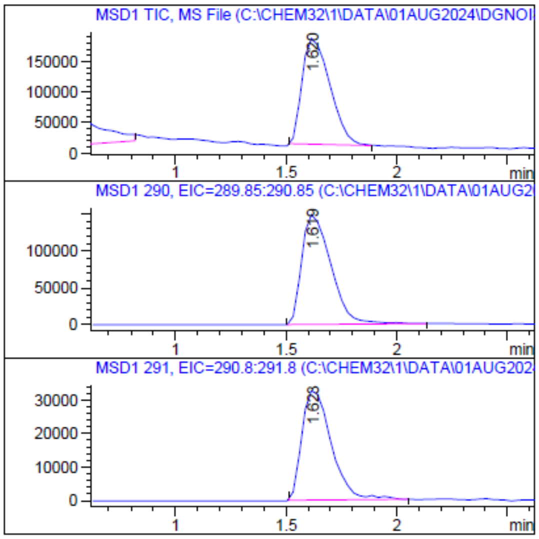

(E)-N′-(3,4-dichlorobenzylidene)-4-methylbenzohydrazide (BE5137)

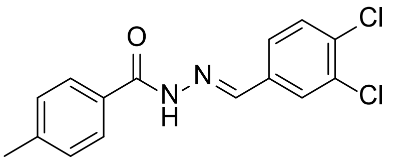

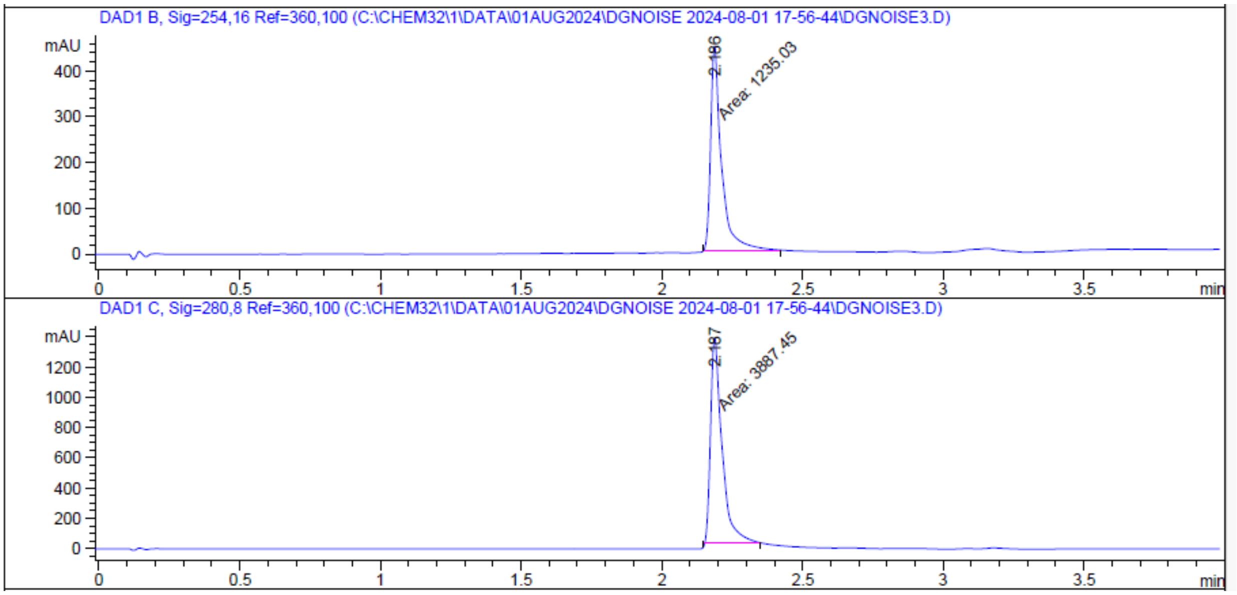

Peak	RT (min)	Area	Area %
1	2.18	3887.45	100

***(E)*-*N′*-(3,4-Dichlorobenzylidene)benzohydrazide (BE5138)**

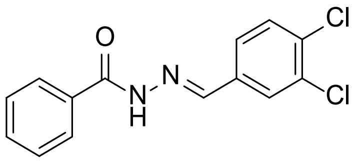

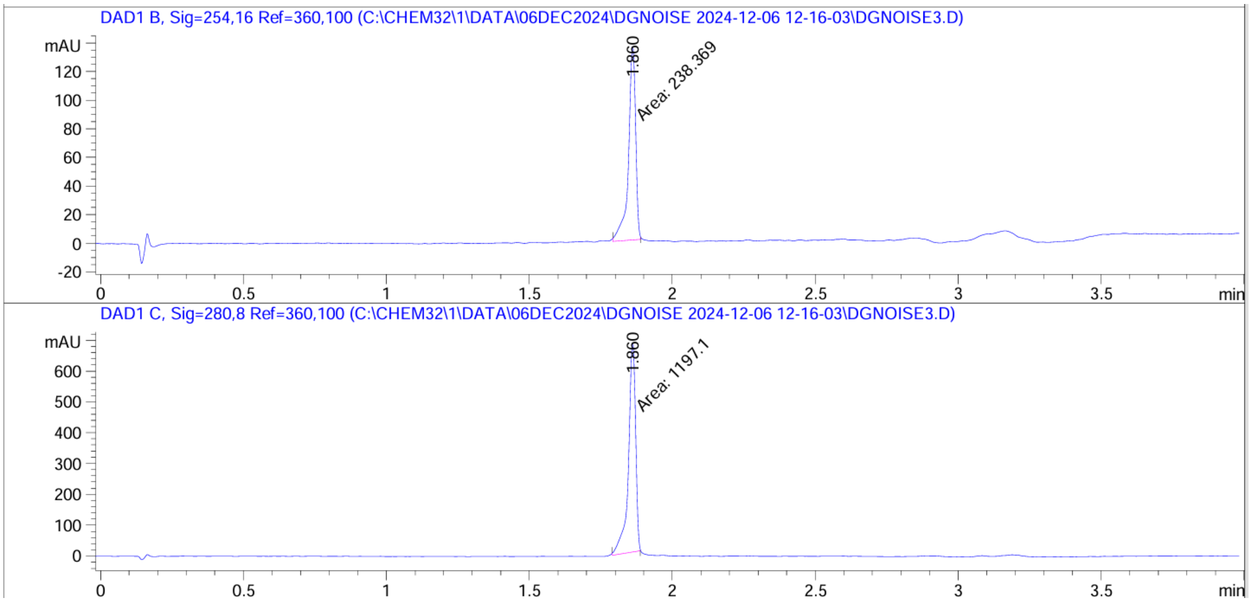

Peak	RT (min)	Area	Area %
1	1.86	1197.1	99.92

(E)-N′-(Quinoxalin-2-ylmethylene)isonicotinohydrazide (BE5056)

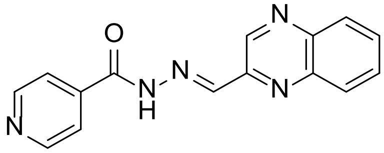

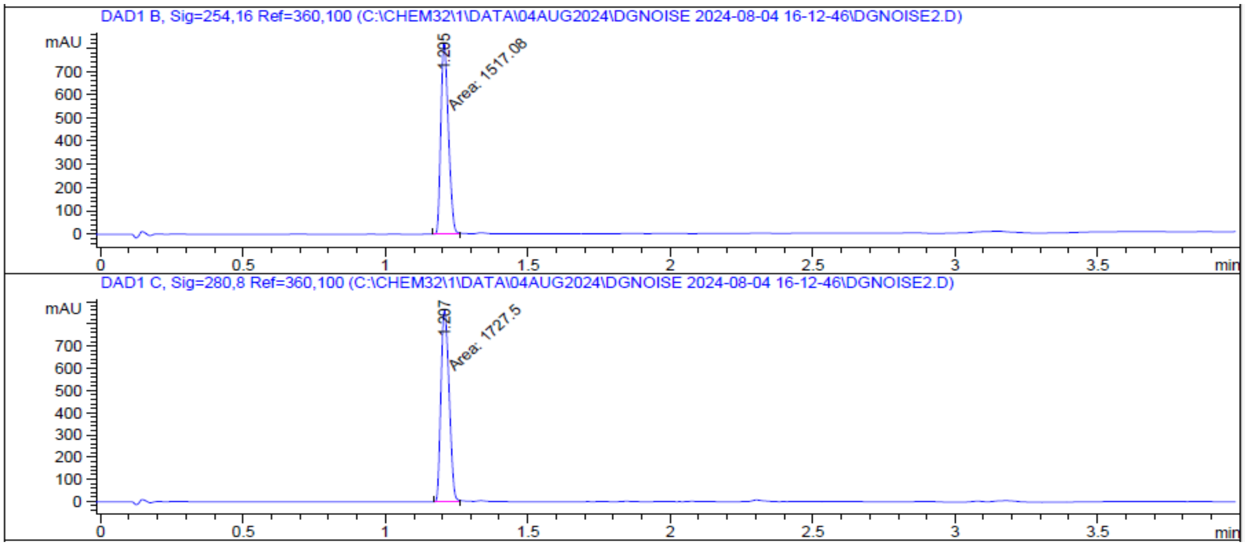

Peak	RT (min)	Area	Area %
1	1.2	1727.5	100

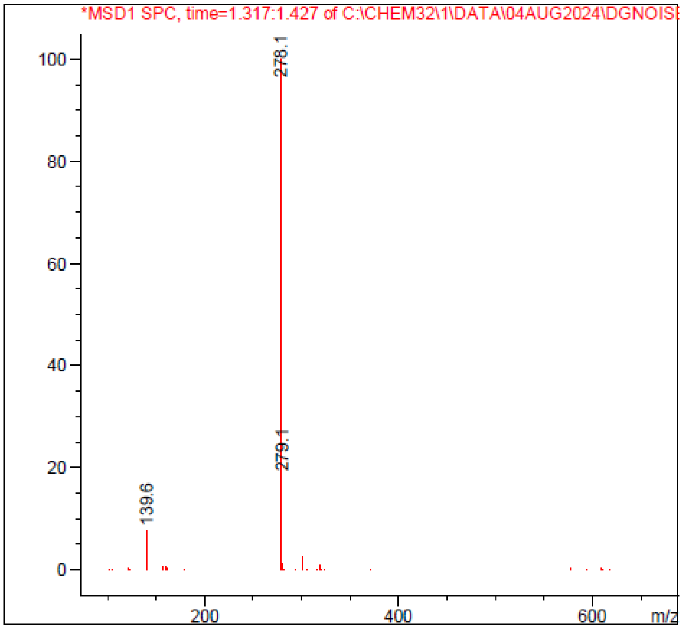

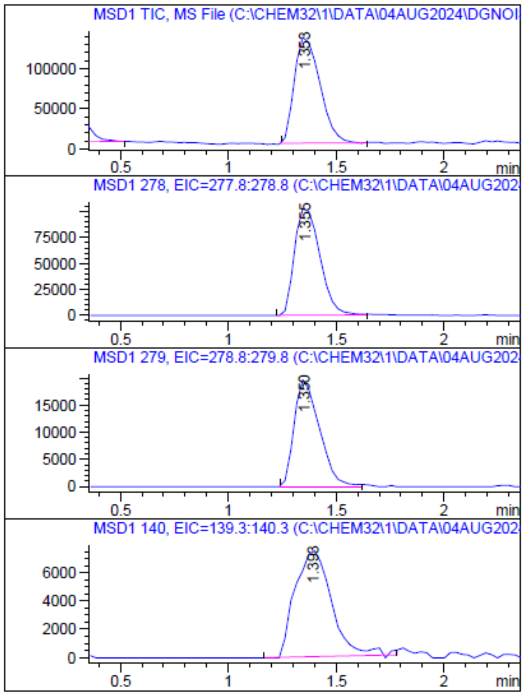

Supplement: 1 [file NIHMS2164575-supplement-1.pdf]
